# Inter- and Transgenerational Effects of In Ovo Stimulation with Bioactive Compounds on Cecal Tonsils and Cecal Mucosa Transcriptomes in a Chicken Model

**DOI:** 10.3390/ijms26031174

**Published:** 2025-01-29

**Authors:** Mariam Ibrahim, Marek Bednarczyk, Katarzyna Stadnicka, Ewa Grochowska

**Affiliations:** 1 Faculty of Health Sciences, Collegium Medicum, Nicolaus Copernicus University, Łukasiewicza 1, 85-821 Bydgoszcz, Poland; miriam.ibrahim@pbs.edu.pl (M.I.); katarzyna.stadnicka@cm.umk.pl (K.S.); 2 PBS Doctoral School, Bydgoszcz University of Science and Technology, Aleje prof. S. Kaliskiego 7, 85-796 Bydgoszcz, Poland; 3 Department of Animal Biotechnology and Genetics, Bydgoszcz University of Science and Technology, Mazowiecka 28, 85-084 Bydgoszcz, Poland

**Keywords:** choline, cecal tonsils, cecal mucosa, in ovo stimulation, intergenerational effect, epigenetic dynamics, transcriptome, transgenerational effect

## Abstract

Exploring how early-life nutritional interventions may impact future generations, this study examines the inter- and transgenerational effects of in ovo injection of bioactive compounds on gene expression in the cecal tonsils and cecal mucosa using a chicken model. Synbiotic PoultryStar^®^ (Biomin) and choline were injected in ovo on the 12th day of egg incubation. Three experimental groups were established in the generation F1: (1) a control group (C) receiving 0.9% physiological saline (NaCl), (2) a synbiotic group (SYN) receiving 2 mg/embryo, and (3) a combined synbiotic and choline group (SYNCH) receiving 2 mg synbiotic and 0.25 mg choline per embryo. For the generations F2 and F3, the SYN and SYNCH groups were each divided into two subgroups: (A) those injected solely in F1 (SYNs and SYNCHs) and (B) those injected in each generation (SYNr and SYNCHr). At 21 weeks posthatching, cecal tonsil and cecal mucosa samples were collected from F1, F2, and F3 birds for transcriptomic analysis. Gene expression profiling revealed distinct intergenerational and transgenerational patterns in both tissues. In cecal tonsils, a significant transgenerational impact on gene expression was noted in the generation F3, following a drop in F2. In contrast, cecal mucosa showed more gene expression changes in F2, indicating intergenerational effects. While some effects carried into F3, they were less pronounced, except in the SYNs group, which experienced an increase compared to F2. The study highlights that transgenerational effects of epigenetic modifications are dynamic and unpredictable, with effects potentially re-emerging in later generations under certain conditions or fading or intensifying over time. This study provides valuable insights into how epigenetic nutritional stimulation during embryonic development may regulate processes in the cecal tonsils and cecal mucosa across multiple generations. Our findings provide evidence supporting the phenomenon of epigenetic dynamics in a chicken model.

## 1. Introduction

A bioactive compound is a substance with biological activity that affects a living organism. The effect of these compounds on organisms can be positive or negative depending on the substance, the dose, and its bioavailability [1]. In the concept of nutrigenetics and nutrigenomics, these substances can transfer information from the external environment and can influence gene expression in the cell, thus modulating metabolic processes and the function of the whole organism [2]. Epigenetic mechanisms can modulate gene expression without altering the underlying DNA sequence. These mechanisms regulate how genes are turned on and off, allowing cells to respond to environmental signals and maintain cell-specific gene expression profiles. Major epigenetic mechanisms include DNA methylation, histone modifications, chromatin remodeling, and non-coding RNAs [3].

Epigenetic inheritance phenomena assume that epigenetic modifications can affect not only the phenotypes of exposed individuals but also their progeny and further subsequent generations through inter- and transgenerational effects occurring either via epigenetic changes during embryonic development or through the inheritance of epigenetic marks from the gametes [4,5]. Epigenetic effects can be classified as inter- or transgenerational. Intergenerational inheritance refers to the transmission of traits or phenotypes between generations that is influenced by environmental factors, often observed in the context of parental experiences affecting offspring [6]. Parental effects are also classified as an example of “context-dependent” epigenetic inheritance [7]. The latter term has a broader meaning. “Context-dependent” epigenetic inheritance is defined as that which results from direct and continuous exposure to an environmental stressor within or across generations [7]. In contrast, transgenerational (so-called “germline-dependent”) inheritance involves the passing of epigenetic changes through the germline, allowing these modifications to affect multiple generations beyond the immediate offspring. As such, only the altered phenotypes occurring in the second (in the case of male transmission) or third (in the case of female transmission) generation after a trigger can truly be described as transgenerational effects [6].

Studies on mammalian models have shown that DNA methylation patterns can be transmitted for generations after exposure to an environmental perturbation (such as toxins, deficient dietary supplements, heat stress, oxidative stress, metabolic disorders, and hormonal exposure) by escaping the transgenerational erasure mechanisms [8]. Importantly, the timing of stress impact has been found to play an important role in determining epigenetic outcomes, with changes occurring early in life potentially having a greater impact than those that occur later [9].

Taking this into consideration, bird models have several advantages over mammalian ones when studying inter- and transgenerational epigenetic inheritance [5]. Chickens are characterized by early sexual maturity, a high rate of egg production (300 eggs/year), and shorter intervals between generations, as well as requiring small floor space and less feed. However, one major advantage is that a bird’s embryo develops outside of the mother, and the maternal influence is reduced only to the egg composition. Other environmental factors, such as the temperature of incubation and humidity, could be strictly controlled to minimize interindividual environmental variability [5]. Moreover, the in ovo technique makes it possible to impact an embryo by direct injection of the studied substance into an egg. Despite these advantages, the chicken model has not been often utilized in inter- and transgenerational studies; therefore, the knowledge in this field needs further exploration.

Currently, synbiotics are widely used to improve health both in humans and animals [10]. Many years of research, including that conducted by our group, have shown that bioactive substances such as prebiotics, probiotics, and synbiotics, administered in ovo to the embryo on day 12 of incubation, may directly affect exposed individuals in the following terms: composition of the microbiota in chickens [11,12], physiological traits [13,14,15], immunological traits [16,17], intestinal development [18,19], performance traits [12,20], and immune-related gene expression in chickens [21,22].

It was observed that epigenetic mechanisms such as DNA methylation and histone modification can be influenced by dietary intake of nutrients like choline and other methyl donors [23]. Prenatal exposure to betaine, a choline metabolite, can modulate hypothalamic cholesterol metabolism in chickens through epigenetic modifications, affecting gene expression and brain function in offspring [24]. Additionally, choline influences the gut microbiome and immune status, promoting beneficial bacteria and improving disease resistance in broiler chickens [25]. Choline supplementation has been shown to alter the gut microbiome composition, increasing the abundance of beneficial bacteria and activating pathways associated with steroid hormone biosynthesis and degradation of environmental pollutants [25].

Taking into consideration the facts mentioned above, for the first time, we stated the hypothesis that a single in ovo injection of bioactive compounds (a synbiotic and its combination with choline) may induce inter- and transgenerational effects on immune-related tissues, altering the transcriptome of both the directly exposed generation and subsequent ones. Therefore, our study aimed to investigate, for the first time, if transcriptome changes that were acquired in one generation, as a result of the prenatal in ovo impact on embryonic and long-term postembryonic development, can be inherited and propagated in the future generations. It should be noted that the novelty of this study is the use of in ovo technology and a chicken model to conduct a three-generational experiment on the effects of bioactive compounds, such as a synbiotic (PoultryStar^®^ solUS, Biomin GmbH, Herzogenburg, Austria) and choline, on immune system tissue transcriptomes, namely cecal tonsils and cecal mucosa. Furthermore, the experimental design was the first of its kind. In parallel, we reproduced birds that received a single in ovo injection in F1 as well as individuals with repeated in ovo injections in each successive generation to investigate both “germline-dependent” and “context-dependent” inheritance.

## 2. Results

In this study, slow-growing local Green-legged partridgelike chickens were used to study inter- and transgenerational effects of bioactive compounds, choline and synbiotic, administered in ovo. Two groups, SYNs and SYNCHs, were designed to investigate the transgenerational impact of the single in ovo synbiotic as well as synbiotic + choline stimulation applied to the eggs laid by F0 hens. In contrast, the SYNr and SYNCHr groups, where chickens received repeated in ovo stimulation in every generation, aimed to explore the cumulative effects of repeated stimulation across generations. We examined the resulting changes in gene expression patterns within immune system tissues, i.e., the cecal mucosa and cecal tonsils, in the generations F1, F2, and F3, following in ovo stimulation at embryonic day 12 with bioactive compounds.

### 2.1. Dose Selection of Synbiotic and Choline

The results of Experiment 1, focused on selecting the choline source and dosage, are presented in Supplementary File S2, Table S1. The highest hatchability rates were observed with choline (Sigma Aldrich, Sain Louis, MA, USA, cat. no. C7527) at both dosages, 0.25 mg and 0.5 mg, achieving 93.3% and 100% hatchability, respectively. A two-way ANOVA was performed to examine the effects of choline source, dose, and their interaction on hatchability (Supplementary File S3, Table S1). None of the factors—choline source, dose, or their interaction—significantly influenced hatchability. Although choline source accounted for 13.8% of the variance in hatchability (η^2^p = 0.138), this effect was not significant. Similarly, dose accounted for only 1.1% of the variance (η^2^p = 0.011), and the interaction term explained 2.2% (η^2^p = 0.022), both of which were also non-significant. For further evaluation, we selected choline (Sigma Aldrich, Sain Louis, MA, USA, cat. no. C7527) and choline (Miavit, Oldenburg, Germany) at both dosages because we observed the highest hatchability for these two products (Supplementary File S2, Table S2).

In Experiment 2, the combination of choline (Sigma Aldrich, Sain Louis, MA, USA, cat. no. C7527) at a dosage of 0.25 mg/embryo and PS synbiotic at a dosage of 2 mg/embryo achieved a 96% hatchability rate across six trials, consistently performing well. Choline (Miavit, Oldenburg, Germany) produced similar results, 96% hatchability, with 0.5 mg choline and 1 mg/embryo synbiotic. A three-way ANOVA was performed to evaluate the effects of choline source, choline dose, synbiotic dose, and their interactions on hatchability (Supplementary File S3, Table S2). None of the main effects (choline source, choline dose, or synbiotic dose) was statistically significant (*p* > 0.05). However, there were significant interactions between choline source and synbiotic dose (*p* = 0.008) and between choline dose and synbiotic dose (*p* = 0.010). Post hoc pairwise comparisons were conducted to investigate the interaction effects of choline source and synbiotic dose, as well as choline dose and synbiotic dose, on hatchability (Supplementary File S3, Tables S3 and S4, respectively). No statistically significant differences were observed between any combinations of choline source and synbiotic dose (Ptukey > 0.05). In contrast, the post hoc analysis for the interaction between choline dose and synbiotic dose revealed a significant difference between 0.25 mg choline with 2 mg synbiotic and 0.5 mg choline with 2 mg synbiotic, with the former showing significantly higher hatchability (*p* = 0.033, mean difference = 7.783%, 95% CI [0.527, 15.040]). Other comparisons within this interaction did not reach statistical significance.

Based on these findings, we selected choline (Sigma Alrich, Sain Louis, MA, USA, cat. no. C7527) at a dosage of 0.25 mg/embryo and PS synbiotic at a dosage of 2 mg/embryo for the three-generational study. While the selected combination of choline and PS synbiotic resulted in the highest hatchability rates, the differences between this combination and others were not statistically significant (*p* > 0.05).

### 2.2. Effect of the In Ovo Stimulation on Body Weights of Adult Chickens

**Figure 1:**
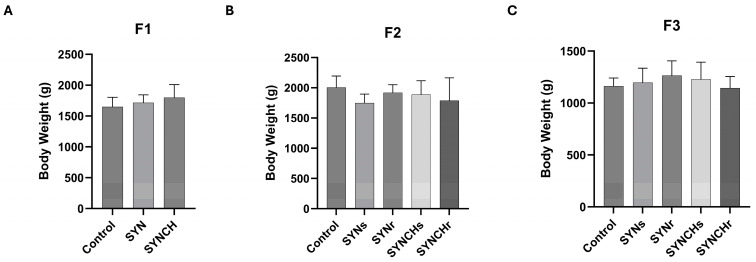
Body weights (in grams) of adult chickens in the different groups of F1 (**A**), F2 (**B**), and F3 (**C**) generations (n = 10 per group in each generation). All data were presented as the mean ± standard deviation (SD). SYN: synbiotic group; SYNCH: synbiotic and choline group; SYNs: single injection (F1) of synbiotic group; SYNr: repeated injections (F1, F2, F3) of synbiotic group; SYNCHs: single injection (F1) of synbiotic + choline group; and SYNCHr: repeated injections (F1, F2, F3) of synbiotic + choline group.

The average body weights of the chickens in each group of each generation are shown in Figure 1. No significant differences in body weights were observed in the experimental groups compared to controls in F1, F2, and F3 (Figure 1). Across all groups, body weights were consistently lower in the generation F3 compared to F2. Although the natural effect of a production season on chicken body weights was observed, the injected bioactive compounds did not affect the body weights of chickens within the same generation.

### 2.3. Gene Expression Changes Induced in Chickens by In Ovo Stimulation with Bioactive Compounds

The input read counts and the uniquely mapped reads to the chicken genome (bGalGal1.mat.broiler. GRCg7b) generated from each group in generations F1, F2, and F3 are summarized in Supplementary File S4. Using datasets derived from these uniquely mapped reads, differential expression analysis was performed, identifying genes with statistically significant changes in expression (adjusted *p*-value of ≤0.05). Differential expression gene (DEG) profiles are presented in Supplementary File S5, showcasing volcano plots and heatmaps.

**Figure 2:**
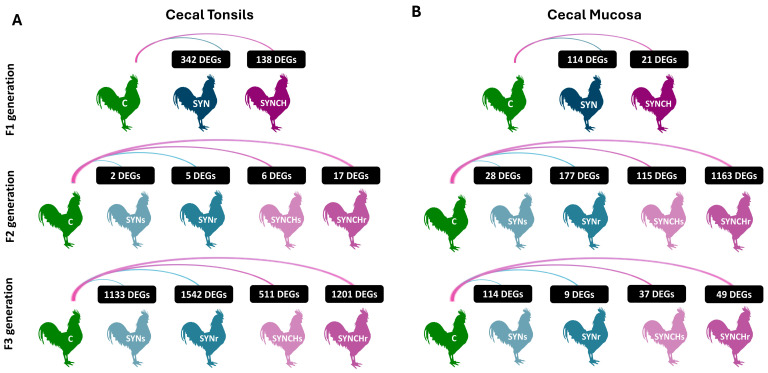
A diagram presenting the number of differentially expressed genes (DEGs) obtained by comparing experimental groups with the control group across three generations: F1, F2, and F3 (n = 3 per group in each generation). The figure is divided into two parts: (**A**) shows the results from the analysis of cecal tonsils, while (**B**) displays the results from the analysis of cecal mucosa. C: control; SYN: synbiotic group; SYNCH: synbiotic and choline group; SYNs: single injection (F1) of synbiotic group; SYNr: repeated injections (F1, F2, F3) of synbiotic group; SYNCHs: single injection (F1) of synbiotic + choline group; and SYNCHr: repeated injections (F1, F2, F3) of synbiotic + choline group.

Figure 2 presents the DEG counts across generations F1, F2, and F3 following in ovo synbiotic and synbiotic + choline stimulation for the cecal tonsils (Figure 2A) and the cecal mucosa (Figure 2B). The identified DEGs across all comparisons in both tissues are provided in Supplementary Files S6 and S7 for cecal tonsils and cecal mucosa, respectively. In generation F1, we observed that both synbiotic and synbiotic + choline administration resulted in notable changes in gene expression compared to the control, with the SYNCH group resulting in fewer DEGs than SYN in both tissues. In the cecal tonsil tissue, by generation F2, the number of DEGs drops across all groups, with the SYNs group showing two DEGs and the SYNr group five DEGs. The SYNCH groups maintained 6 DEGs in SYNCHs and 17 DEGs in SYNCHr. In the cecal mucosa in the generation F2, we observed a much larger increase in DEGs, particularly in the SYNr (177 DEGs) and SYNCHr (1163 DEGs) groups. In comparison, the SYNs and SYNCHs groups maintained 28 and 115 DEGs, respectively. In generation F3, we observed a resurgence of DEGs in the cecal tonsils, particularly in the SYNr group with 1542 DEGs and the SYNs group with 1133 DEGs, followed by the SYNCHr group with 1201 DEGs and the SYNCHs group with 511 DEGs. In the cecal mucosa; however, the number of DEGs decreased in F3, except for that of the SYNs group, which increased to 114 DEGs. The SYNr group exhibited 9 DEGs, while the SYNCHs and SYNCHr groups showed 37 and 49 DEGs, respectively. Overall, the data demonstrate that synbiotic and synbiotic + choline treatments have distinct effects on gene expression in both the cecal tonsils and cecal mucosa. The results suggest a strong transgenerational effect in F3 (SYNs and SYNCHs) on gene expression in the case of cecal tonsils despite the decrease in DEGs in F2 which is linked to the intergenerational effect of the stimulation. Repeated in ovo stimulation amplifies these effects, particularly in generation F3. On the other hand, the results observed in the case of cecal mucosa indicate an intergenerational effect in F2 and a potential transgenerational effect on gene expression in F3 (SYNs and SYNCHs). Repeated injections across generations intensify gene expression changes, particularly in F2, but may stabilize by F3.

**Figure 3:**
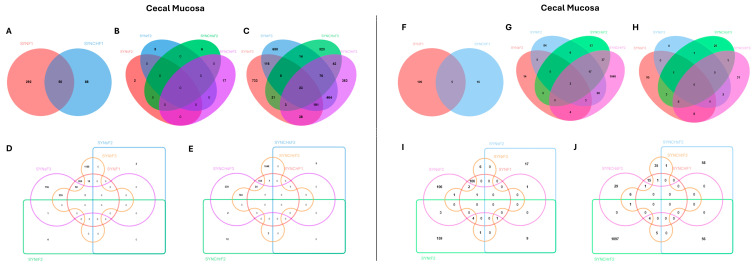
Venn diagrams illustrating the distribution of differentially expressed genes (DEGs) across comparisons in F1 (**A**,**F**), F2 (**B**,**G**), and F3 (**C**,**H**) generations, all synbiotic groups (**D**,**I**), and all synbiotic + choline (**E**,**J**) groups for cecal tonsils and cecal mucosa.

Figure 3 shows the Venn diagrams illustrating the distribution and the overlapping of DEGs across different comparisons in the three generations for cecal tonsils and cecal mucosa, respectively. The overlapping genes are listed in Supplementary Files S8 and S9 for cecal tonsils and cecal mucosa, respectively.

### 2.4. Functional Clustering Based on Gene Ontology (GO) and KEGG Pathways

Functional information was extracted from the DEG datasets using Gene Ontology (GO) enrichment analysis. The enriched GO terms were categorized into three groups: biological process (BP), cellular component (CC), and molecular function (MF). The complete lists of significant GO terms across all comparisons for the two tissues—cecal tonsils and cecal mucosa—are provided in Supplementary Files S10 and S11, respectively. Likewise, the lists of significant KEGG pathways across all comparisons for cecal tonsils and cecal mucosa can be found in Supplementary Files S12 and S13, respectively.

#### 2.4.1. GO Terms and KEGG Pathways Enrichment Related to Cecal Tonsils

**Figure 4:**
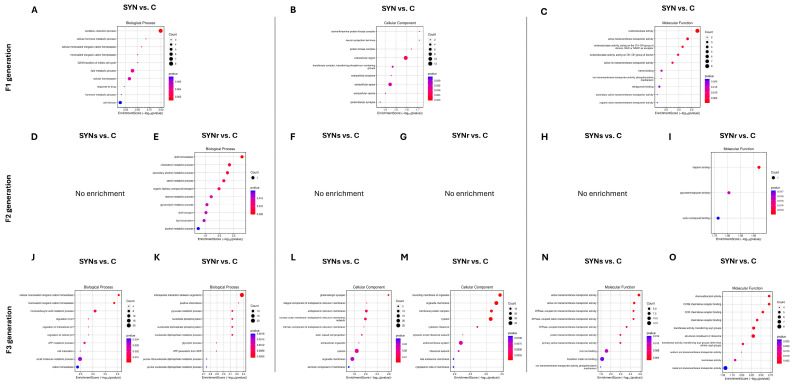
Gene Ontology (GO) enrichment analysis of DEGs in cecal tonsils across F1, F2, and F3 generations. (**A**–**O**) Bubble plots showing top 10 enriched terms for biological processes (**A**,**D**,**E**,**J**,**K**), cellular components (**B**,**F**,**G**,**L**,**M**), and molecular functions (**C**,**H**,**I**,**N**,**O**) in SYN groups. The size of the bubbles represents the number of enriched genes, and the color gradient indicates the enrichment significance. F1 results demonstrate response to direct exposure to synbiotic treatment, F2 shows intergenerational effects, and F3 reveals transgenerational effects.

Figure 4 shows the top ten GO term enrichment analysis in cecal tonsils across three successive generations, comparing the control and synbiotic-injected groups. In the first generation (F1), biological processes were primarily related to cellular homeostasis. The second generation (F2) showed a reduction in gene expression enrichment. Both single (SYNs) and repeated injection (SYNr) groups exhibited minimal functional enrichment across biological processes, cellular components, and molecular functions. In the third generation (F3), gene expression dramatically increased. Biological processes re-emphasized cellular homeostasis and metabolic activities. Molecular functions expanded to include transmembrane transporter activity, chemoattractant activity, and chemokine receptor binding. The repeated injection groups (SYNr) demonstrated additional enrichment in specific cellular transition processes and metabolic pathways, particularly in the third generation.

**Figure 5:**
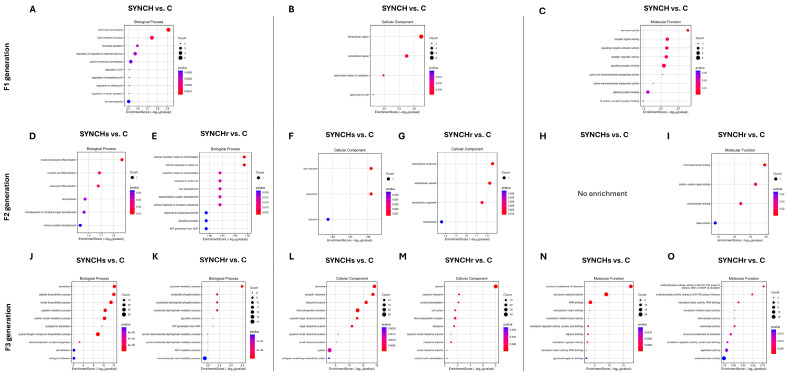
Gene Ontology (GO) enrichment analysis of DEGs in cecal tonsils across F1, F2, and F3 generations. (**A**–**O**) Bubble plots showing top 10 enriched terms for biological processes (**A**,**D**,**E**,**J**,**K**), cellular components (**B**,**F**,**G**,**L**,**M**), and molecular functions (**C**,**H**,**I**,**N**,**O**) in SYNCH groups. The size of the bubbles represents the number of enriched genes, and the color gradient indicates the enrichment significance.

Figure 5 displays the GO term enrichment analysis in cecal tonsils across three successive generations, comparing the control and synbiotic + choline-injected groups. In F1, the SYNCH group showed enrichment in chemical homeostasis, lipid metabolism, and hormone transport (*p* < 0.05). F2 demonstrated reduced enrichment, with the SYNCHs group showing enrichment in immune system development and the SYNCHr group in cellular transitions (*p* < 0.05). F3 exhibited increased enrichment in both SYNCHs (511 terms) and SYNCHr (1201 terms) groups compared to control, with translation and biosynthetic processes dominating in SYNCHs and pyruvate metabolism and ATP generation prominent in the SYNCHr group. For molecular functions, F1 showed enrichment in hormone and receptor activities, while F3 displayed significant enrichment in ribosomal structure and RNA binding (SYNCHs) and oxidoreductase activity (SYNCHr). Both F3 groups showed enrichment in translation regulation compared to control.

**Figure 6:**
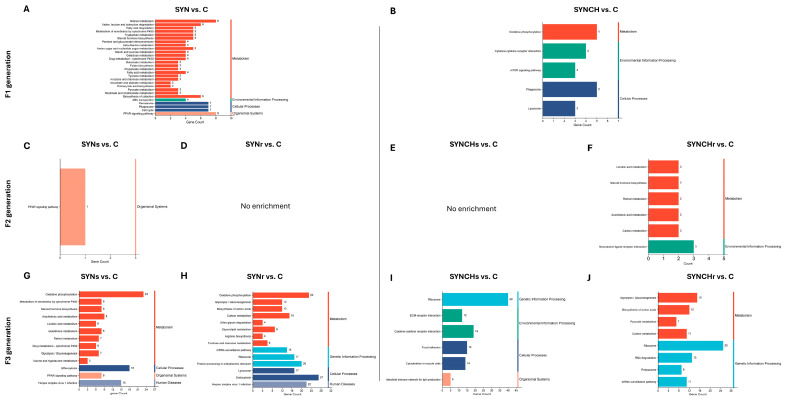
KEGG pathway enrichment analysis of DEGs in cecal tonsils across F1, F2, and F3 generations. (**A**–**J**) Bar plots depict the enriched KEGG pathways in SYN and SYNCH groups. Enrichment is shown for SYN groups in F1 (**A**), F2 (**C**,**D**), and F3 (**G**,**H**) and for SYNCH groups in F1 (**B**), F2 (**E**,**F**), and F3 (**I**,**J**). Each bar represents a pathway, with bar length corresponding to the number of enriched genes.

Figure 6 presents the KEGG pathway enrichment analysis in cecal tonsils across the generations F1, F2, and F3. In the F1 SYN group, metabolic pathways including retinol metabolism and steroid hormone biosynthesis showed significant enrichment (*p* < 0.05). The peroxisome proliferator-activated receptor (PPAR) signaling pathway was enriched in both F1 SYN and F3 SYNs groups compared to control. In F2, pathway enrichment was limited, though PPAR signaling persisted in the SYNs group. F3 SYNs and SYNr groups shared a common enrichment profile in oxidative phosphorylation and glycolysis pathways versus control. For SYNCH groups, F1 showed enrichment in oxidative phosphorylation, phagosome, and lysosome pathways. The cytokine–cytokine receptor interaction pathway was enriched in both F1 SYNCH and F3 SYNCHs groups, while the carbon metabolism pathway appeared in both F2 SYNCHr and F3 SYNCHr groups. In F3, both SYNCHs and SYNCHr groups showed significant enrichment in the ribosome pathway compared to control. Significant KEGG pathways, visualized with Pathview, are shown in Supplementary File S14.

#### 2.4.2. GO Term and KEGG Pathway Enrichment Related to Cecal Mucosa

**Figure 7:**
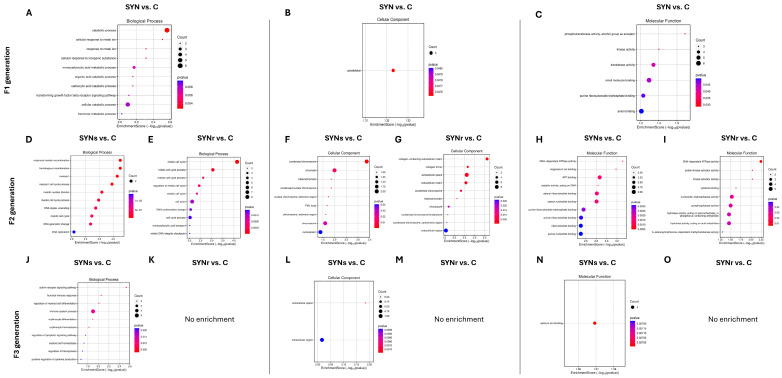
Gene Ontology (GO) enrichment analysis of DEGs in cecal mucosa across F1, F2, and F3 generations. (**A**–**O**) Bubble plots showing top 10 enriched terms for biological processes (**A**,**D**,**E**,**J**,**K**), cellular components (**B**,**F**,**G**,**L**,**M**), and molecular functions (**C**,**H**,**I**,**N**,**O**) in SYN groups. The size of the bubbles represents the number of enriched genes, and the color gradient indicates the enrichment significance.

Figure 7 shows the top ten GO term enrichment analysis in cecal mucosa across three successive generations, comparing the control and synbiotic-injected groups. The F1 SYN treatment demonstrated significant enrichment (*p* < 0.05) in pathways associated with catabolic processes and metal ion response. F2 generation analysis revealed enrichment in cell cycle and genomic regulation pathways in both SYNs and SYNr groups (*p* < 0.05 vs. control). The F3 SYNs treatment group exhibited significant enrichment in immune system-associated processes. Analysis of cellular components identified cytoskeletal element enrichment in F1 SYN, while F2 SYNs and SYNr groups displayed enrichment in chromosomal components and heterochromatin regions. Molecular function assessment demonstrated significant enrichment in kinase and phosphotransferase activity (F1 SYN) and purine ribonucleoside triphosphate binding (F1 SYN, F2 SYNs). Additionally, DNA-dependent ATPase activity showed consistent enrichment in F2 SYNs and SYNr groups relative to control.

**Figure 8:**
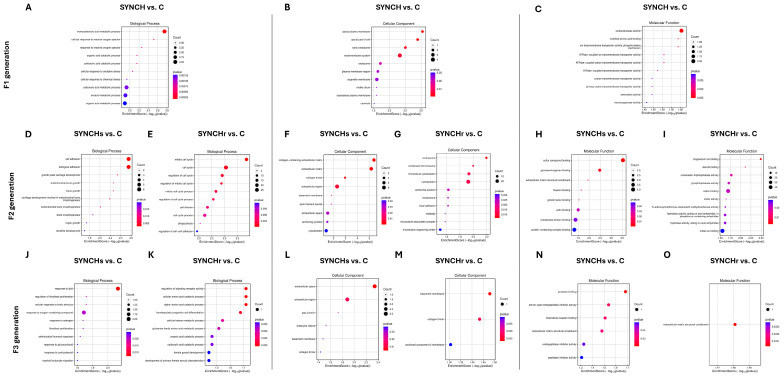
Gene Ontology (GO) enrichment analysis of DEGs in cecal mucosa across F1, F2, and F3 generations. (**A**–**O**) Bubble plots showing top 10 enriched terms for biological processes (**A**,**D**,**E**,**J**,**K**), cellular components (**B**,**F**,**G**,**L**,**M**), and molecular functions (**C**,**H**,**I**,**N**,**O**) in SYNCH groups. The size of the bubbles represents the number of enriched genes, and the color gradient indicates the enrichment significance.

Figure 8 presents the top ten GO term enrichment analysis of the cecal mucosa across three successive generations, comparing control and synbiotic+choline groups. The F1 SYNCH treatment exhibited significant enrichment (*p* < 0.05) in monocarboxylic acid metabolism and reactive oxygen species response pathways. Analysis of the F2 generation revealed enrichment in cell adhesion processes in both SYNCHs and SYNCHr groups, with additional enrichment in cell cycle and phagocytosis pathways specific to F2 SYNCHr (*p* < 0.05 vs. control). Cellular component assessment identified enrichment in apical cell regions and organelle membrane components in F1 SYNCH, while cytoskeletal components showed significant enrichment in both F2 SYNCHs and SYNCHr groups. Molecular function analysis demonstrated enrichment in oxidoreductase and transmembrane transporter activities in F1 SYNCH, sulfur compound and glycosaminoglycan binding in F2 SYNCHs, and ion binding and hydrolase activity in F2 SYNCHr relative to control (*p* < 0.05).

**Figure 9:**
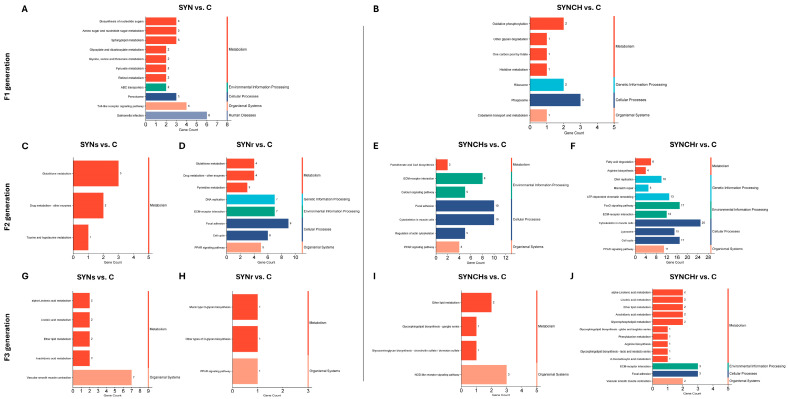
KEGG pathway enrichment analysis of DEGs in cecal mucosa across F1, F2, and F3 generations. (**A**–**J**) Bar plots depict the enriched KEGG pathways in SYN and SYNCH groups. Enrichment is shown for SYN groups in F1 (**A**), F2 (**C**,**D**), and F3 (**G**,**H**) and for SYNCH groups in F1 (**B**), F2 (**E**,**F**), and F3 (**I**,**J**). Each bar represents a pathway, with bar length corresponding to the number of enriched genes.

Figure 9 presents the KEGG pathway enrichment analysis in cecal mucosa across the generations F1, F2, and F3. In the synbiotic groups, the KEGG pathway enrichment analysis revealed a strong focus on metabolism across generations. The F1 SYN treatment demonstrated significant enrichment (*p* < 0.05) in nucleotide sugar biosynthesis, amino sugar metabolism, sphingolipid metabolism, and retinol metabolism pathways. Toll-like receptor signaling pathways showed concurrent enrichment. F2 analysis identified enrichment in glutathione metabolism and drug metabolism pathways in both SYNs and SYNr groups, with the PPAR signaling pathway specifically enriched in F2 SYNr and persisting in F3 SYNr (*p* < 0.05 vs. control). The F3 SYNs group exhibited significant enrichment in lipid-associated pathways, notably linoleic acid and arachidonic acid metabolism. In SYNCH groups, F1 treatment showed enrichment in oxidative phosphorylation pathways, while F2 SYNCHs demonstrated enrichment in extracellular matrix (ECM)–receptor interaction and cytoskeletal components. F2 SYNCHr maintained similar pathway enrichment with additional PPAR signaling pathway activation. F3 analysis revealed significant enrichment in ether lipid metabolism and glycosphingolipid biosynthesis pathways in both SYNCHs and SYNCHr groups relative to control (*p* < 0.05).

Significant KEGG pathways were visualized using Pathview, highlighting potentially affected genes (Supplementary File S15).

### 2.5. Validation of Sequencing Data by RT-qPCR

**Figure 10:**
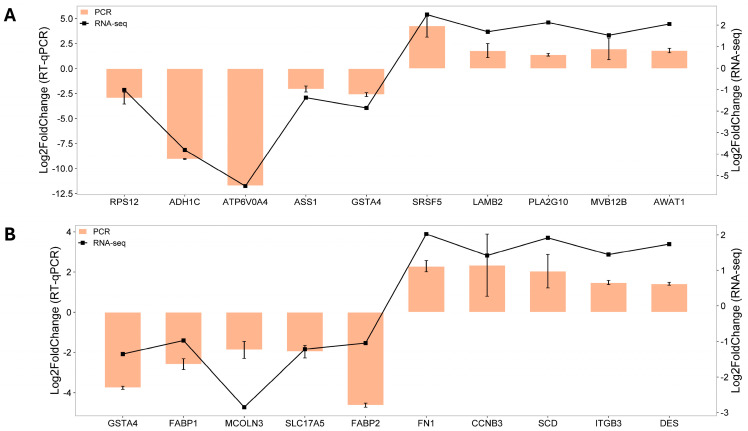
RT-qPCR validation of 10 selected genes for each tissue. PCR vs. RNA-seq dual y-axis plot for the genes differentially expressed in the (**A**) cecal tonsils and (**B**) cecal mucosa. All data from RT-qPCR analyses were presented as the mean ± standard error of the mean (SEM).

Figure 10 presents the log2 fold change of the ten selected DEGs in each tissue, analyzed using both RT-qPCR and RNA sequencing. In the cecal tonsils (Figure 10A), RT-qPCR showed upregulation of *SRSF5*, *LAMB2*, *PLA2G10*, *MVB12B*, and *AWAT1*, along with downregulation of *RPS12*, *ADH1C*, *ATP6V0A4*, *ASS1*, and *GSTA4*. These results align with the RNA-sequencing data, demonstrating the reliability of the sequencing approach. Similarly, in the cecal mucosa (Figure 10B), RT-qPCR indicated upregulation of *FN1*, *CCNB3*, *SCD*, *ITGB3*, and *DES* and downregulation of *GSTA4*, *FABP1*, *MCOLN3*, *SLC17A5*, and *FABP2*. The strong concordance in gene expression patterns and log2 fold change values between RT-qPCR and RNA sequencing further supports the accuracy and reliability of the RNA-seq data.

## 3. Materials and Methods

### 3.1. Ethical Consideration

The animals were handled following the decision of the Local Ethical Committee for Animal Experiments in Bydgoszcz, Poland (Approval No. 15/2022 on 20 April 2022), Directive 2010/63/EU and Regulation (EU) 2019/1010. Welfare monitoring was applied. Birds were kept in standard environmental conditions on a poultry farm. Qualified personnel carried out the rearing of birds. A veterinarian at the facility provided oversight of animal welfare. The study complies with the 3Rs principles and ethical standards. No suitable in vitro alternatives exist for studying transgenerational epigenetic effects in avian models.

### 3.2. Animals

The study involved Green-legged partridgelike chickens, a local Polish slow-growing breed known for its minimal environmental and nutritional demands, hardiness, resistance to harsh conditions, and well-developed maternal traits [26]. This breed has not undergone extensive selective breeding [26], maintaining a wider range of genetic traits.

### 3.3. Selection and Dosage Testing of Choline and Synbiotic

Fertilized eggs obtained from the F0 hens were incubated in standard conditions in a commercial hatchery, Wagrowiec, Poland (37.5 °C, 55% relative humidity, turned every 2 h, for 18 days, then in the hatcher for 3 days at 36.9 °C, 65% relative humidity). On the 12th day of embryonic development, after candling, bioactive compounds suspended in 0.2 mL of NaCl were manually injected into the air chamber of 10–15 eggs (Experiment 1) or 19–22 eggs (Experiment 2) with viable embryos per replicate. After injection, the hole was sealed with non-toxic glue to avoid embryo contamination and prevent moisture loss. The eggs were then returned to incubation under the same standard conditions. The in ovo injection protocol, using 0.2 mL of 0.9% NaCl, was adapted from the method optimized by Bednarczyk et al. [11,12] to ensure effective compound delivery without harming embryonic development.

#### 3.3.1. Experiment 1

Experiment 1 aimed to select a proper choline source and dosage. Four different choline sources were tested: (1) choline chloride (Sigma Aldrich, Saint Louis, MA, USA, cat. no. PHR1251); (2) choline chloride (Sigma Aldrich, Sain Louis, MA, USA, cat. no. 26978); (3), choline chloride (Sigma-Aldrich, Saint Louis, MA, USA, cat. no. C7527), and (4) choline chloride (Miavit, Oldenburg, Germany). Two dosages, 0.5 mg/embryo and 0.25 mg/embryo, were evaluated for their effects on the eggs’ hatchability. For each group three repetitions were tested separately. A control group received 0.9% NaCl. The results from the three repetitions were summed up. Hatchability was calculated for each group from the following formula: total number of hatched chicks to the number of viable eggs, candled and injected at day 12 of incubation multiplied by 100. Two choline sources from the groups with the highest hatchability were selected for the second experiment.

#### 3.3.2. Experiment 2

The aim of the second experiment was to select the proper combination of choline and synbiotic for further study in the project. Two choline sources that showed the best results in Experiment 1 were combined with the synbiotic (PoultryStar^®^ sol^US^, Biomin GmbH, Herzogenburg, Austria). The two choline products were administered at dosages of 0.25 mg/embryo and 0.5 mg/embryo, and each dosage was cross-combined with two dosages of the synbiotic, 1 mg/embryo and 2 mg/embryo. Each combination was tested in six repetitions, with 19–22 eggs per repetition. A control group receiving 0.9% NaCl was also included in this phase. After injection, eggs were further incubated under the standard conditions as described before. The results from the six repetitions were summed up. Hatchability was calculated according to the formula described in Experiment 1. Based on the hatchability of the eggs, the optimal combination of choline and synbiotic doses was selected for further experiments in the project. The combined solution of synbiotic and choline was administered manually into the air chamber of fertilized viable eggs on embryonic day 12. The synbiotic preparation used for in ovo administration, PoultryStar^®^ sol^US^ (PS; Biomin GmbH, Herzogenburg, Austria), consisted of a prebiotic (inulin) and a probiotic mixture of four microbial strains (5.0 × 10^9^ CFU/g): *Pediococcus acidilactici* from the cecum, *Bifidobacterium animalis* from the ileum, *Enterococcus faecium* from the jejunum, and *Lactobacillus reuteri* from the crop. The PS synbiotic is a commercial, well-defined, poultry-specific, multi-species synbiotic product that promotes a beneficial gut microbiota through the combined action of carefully selected probiotic microorganisms and prebiotic fructooligosaccharides [27]. It is also easily soluble in water, so it can be used for in ovo injections.

Statistical analyses were performed using JASP (version 0.19.3, JASP Team (2025), Amsterdam, The Netherlands). Hatchability data were analyzed using both two-way and three-way analyses of variance (ANOVAs) to examine the effects of choline source, choline dose, synbiotic dose, and their interactions on hatchability rates. For the two-way ANOVA, we assessed the effects of choline source and dose on hatchability. The three-way ANOVA included choline source, choline dose, and synbiotic dose as independent factors to evaluate potential interaction effects among these variables. Post hoc pairwise comparisons were conducted using Tukey’s HSD test to identify specific differences within significant interactions. Effect sizes were reported as partial eta-squared (η^2^p) and confidence intervals for mean differences were adjusted for multiple comparisons. Significance was determined at *p* < 0.05.

### 3.4. Experimental Design

Fertilized eggs from F0 green-legged partridgelike hens were incubated under standard conditions as described before at a commercial hatchery in Wagrowiec, Poland. On the 12th day of embryonic development, viable embryos identified by candling were randomly assigned to one of three experimental groups: (1) the synbiotic group (SYN), which received an injection of 2 mg PS synbiotic suspended in 0.2 mL NaCl; (2) the synbiotic and choline group (SYNCH), which received an injection of 2 mg PS synbiotic and 0.25 mg choline (Sigma Aldrich, Sain Louis, MA, USA, cat. no. C7527) suspended in 0.2 mL NaCl; and (3) the control group (C), which received an injection of 0.2 mL NaCl (0.9%). This rearing scheme was continued through three generations (F2 and F3). In F2 and F3, treatment groups were split into four subgroups: two groups continued with the single injection (without repeated injection in F2 and F3), one with synbiotic alone (SYNs) and the other with synbiotic and choline (SYNCHs). The other two groups received repeated injections of synbiotic alone (SYNr) and synbiotic with choline (SYNCHr) in F2 and F3.

After hatching, all chickens of each generation were raised in the same local poultry farm under semi-intensive conditions in floor pens with a bedding made of chopped wheat straw, enriched with perches, with 30 birds per experimental and control group (allowing natural behaviors) in two rearing replicates per experimental group and generation. Indoor parameters were maintained according to breed-specific requirements, with ambient temperature stabilized in cold seasons at 16–18 °C. Photoperiod management combined natural light exposure through facility windows with supplementary artificial lighting. During the growth phase, a 12:12 light:dark cycle was implemented. Upon reaching reproductive maturity, the photoperiod was gradually extended to maximally 16–17 h of light (20–36 weeks of age), initiated at dawn, to optimize egg production for generational progression.

All birds of each generation were fed the same commercial diet free from antibiotics, probiotics, and prebiotics, purchased from a feed company (Golpasz, De Heus, Golub-Dobrzyń, Poland). Laying hens were fed a diet prepared on the farm consisting of 75% winter wheat and 25% concentrate for laying hens from De Heus (manufacturer’s code: 1957—HD660X00S-W00). Birds had free access to fresh water. Individual body weights of 10 randomly selected adult chickens (after a fasting period of 12 h) per group were measured in week 21 of life across the five groups in each generation. GraphPad Prism (version 10.0.1) software (GraphPad Software, La Jolla, CA, USA) was employed for data analysis using one-way ANOVA.

### 3.5. Tissue Collection and RNA Isolation

Samples of cecal tonsils and cecal mucosa were collected from randomly selected 21-week-old chickens (n = 6 per group per generation). Samples were preserved in RNAlater buffer (ThermoFisher, Waltham, MA, USA) and then stored at −80 °C until use. To homogenize the tissue samples, metal beads (2.4 mm, cat. no. 10032-370, OMNI International, Tulsa, OK, USA) were employed. RNA isolation was performed using the GeneMATRIX Universal RNA Purification Kit (EURx, Gdańsk, Poland, cat. no. E3598), following the manufacturer’s protocol for animal tissues with RNA Extracol reagent (EURx, Gdańsk, Poland, cat. no. E3700). RNA quantity and purity were assessed on a NanoDrop 2000 spectrophotometer (Thermo Scientific, Waltham, MA, USA). The integrity of the isolated RNA was assessed using an Agilent Bioanalyzer 2100 system (Agilent Technologies, Santa Clara, CA, USA) with an RNA Nano 6000 Assay Kit (Agilent Technologies, Santa Clara, CA, USA). Furthermore, RNA degradation and contamination were monitored on 1% agarose gel. All the extracted RNA samples passed the quality control requirements (RNA integrity number (RIN) ≥ 7.5) and were processed for downstream applications.

### 3.6. RNA-Sequencing and Bioinformatic Analysis

In total, 78 RNA-seq libraries (n = 39 per tissue) were prepared using Novogene NGS Stranded RNA Library Prep Set (PT044, Novogene, Cambridge, UK). All cDNA libraries were sequenced using a paired-end strategy with a reading length of 150 bps on an Illumina NovaSeq 6000 sequencing platform (Illumina, San Diego, CA, USA) at a depth of 20 million reads per sample by Novogene (Novogene, Cambridge, UK). FastQC v0.12.1 was used to perform the raw sequencing data’s quality control [28]. Next, the raw data were processed using fastp tool v0.23.4 [29] to remove adapter sequences and trim low-quality reads to obtain clean data for downstream analyses. Simultaneously, the Q20, Q30, and GC contents of the clean data were calculated. All the paired-end reads (n = 3 per group and per generation in each tissue) passed the quality control and were mapped to the chicken reference genome (bGalGal1.mat.broiler.GRCg7b) using STAR v.2.7.11b aligner [30]. The DESeq2 v.1.42.0 program in RStudio v.2024.09.0+375.pro3 was used to perform the differential expression analysis [31]. DESeq2 was used to normalize the raw counts. A fold change criterion of less than (for downregulated genes) or greater than 0 (for upregulated genes) and an adjusted *p*-value less than or equal to 0.05 were used to define differentially expressed genes. The Kyoto Encyclopedia of Genes and Genomes (KEGG) and Gene Ontology (GO) pathway enrichment analysis was carried out with the Scientific and Research plot tool (SRplot, http://www.bioinformatics.com.cn/SRplot, accessed on 7 October 2024) [32], which utilizes clusterProfiler [33]. Significantly enriched KEGG pathways were visualized using Pathview [34]. Jvenn, https://jvenn.toulouse.inrae.fr/app/index.html, accessed on 7 October 2024, was used to construct the Venn diagrams [35].

### 3.7. Validation of Sequencing Data by Reverse Transcription–Quantitative Polymerase Chain Reaction (RT-qPCR)

Five up- and five downregulated significantly differentially expressed genes involved with different KEGG pathways were chosen for RT-qPCR assessment to validate the RNA sequencing output (Supplementary File S15). The smART First strand cDNA Synthesis kit (Eurx, Gdańsk, Poland, cat. no. E0804) was used to prepare the cDNA. Primers for the selected genes were designed using Primer Blast [36]. Supplementary File S1 shows the list of primers used for the real-time qPCR amplification of the cDNA. Reference genes were selected according to the results of the reference gene stability experiment [37]. First, 50 ng of cDNA, 0.25U of uracil-*N*-glycosylase (UNG), and 15 pmol of each forward and reverse amplification primer were added to a 1 × SG qPCR master mix (Eurx, Gdańsk, Poland, E0401) in a 20 μL volume for each reaction. Thermocycling conditions for RT-qPCR were as follows: 1 cycle for UNG pretreatment at 50 °C for 2 min, 1 cycle for initial denaturation at 95 °C for 10 min, and 40 cycles of 94 °C for 15 s, 60 °C for 30 s, and 72 °C for 30 s. All amplicons’ melting curve profiles were examined under the following thermal conditions: 95 °C for 5 s, 70 °C for 5 s, and then a gradual rise in temperature to 95 °C at a ramp rate of 0.5 °C/5 s. The CFX Opus 96 real-time PCR equipment (BIO-RAD, Hercules, CA, USA) was used for the amplification. The relative expression levels of the studied genes were examined using the Pffafl (or standard curve) approach [38]. The double y-axis plot of PCR expression versus RNA-seq expression was visualized using the SRplot tool [32].

## 4. Discussion

To the best of our knowledge, we are the first to utilize a chicken model in this study to conduct a comprehensive inter- and transgenerational experiment investigating the effects of bioactive compounds, i.e., PS synbiotic and choline on immune system tissue transcriptomes.

Many studies on pre-, pro-, and synbiotics have focused on their effect on exposed individuals and/or their immediate offspring [39,40,41]. However, little is known about the effects of pre-, pro-, and synbiotic supplementation on further generations. Taking into account the potential of pre-, pro-, and synbiotics in building the body’s immunity, we found it interesting to study if the alterations introduced by a synbiotic as well as a synbiotic combined with choline in the transcriptome of immune system tissues can be observed in further generations, i.e., F2 and F3. The changes in tissue gene expression or transcriptome often act as precursors or direct contributors to phenotypic changes. These alterations in gene expression can arise from a variety of factors, broadly categorized as genetic, epigenetic, and environmental influences [42,43]. Epigenetic factors, in particular, involve modifications that affect gene expression without altering the DNA sequence itself. Such mechanisms include DNA methylation, histone modification, and regulation by non-coding RNAs. These epigenetic changes can influence chromatin structure and gene accessibility, potentially altering gene expression. Importantly, epigenetic modifications are reversible and can be influenced by environmental conditions, lifestyle, and other external factors [42]. Herein, we decided to study immune system transcriptomes as a link between epigenetic alterations and an individual’s phenotype due to the fact that it is generally accepted that changes in the epigenetic mechanism can alter phenotypic characteristics [44]. In our study, we established treatment groups which received single injections in eggs laid by generation F0 hens to study the phenomenon of transgenerational (germline-dependent) epigenetic inheritance in successive generations. In parallel, we also reproduced the treatment groups that received repeated injections in each generation to investigate the multigenerational effects of introduced bioactive compounds directly on the exposed generation as well as their cumulative effects in the successive generations.

We observed that the whole-genome gene expression profiles showed distinct intergenerational and transgenerational patterns in cecal tonsils and cecal mucosa stimulated in ovo with a synbiotic and a synbiotic combined with choline. In cecal tonsils, we revealed a very high increase in the DEG number in F3 between treated groups and the control, suggesting a transgenerational effect of synbiotic and choline injection. Interestingly, the effects were less pronounced in the generation F2, showing a sharp reduction in DEGs before the spike in F3. However, in cecal mucosa, the gene expression effects were more prominent in the generation F2, indicating intergenerational effects. In F3, some of these effects carried over, suggesting the potential for transgenerational influence, although the DEGs did not reach the same levels as in F2. An exception was seen in the SYNs group, where the effect increased in F3 compared to F2. Hence, cecal tonsils demonstrated robust transgenerational effects by F3, while cecal mucosa had intergenerational changes in F2 with the potential for continued, though less pronounced, transgenerational effects in F3. This observation can be supported by considering the specialized immune functions and intricate architecture of cecal tonsils, which likely render them more susceptible to transgenerational programming due to their continuous exposure to diverse antigens and their crucial role in shaping the immune system [45,46]. In contrast, the cecal mucosa, primarily involved in nutrient absorption and barrier function [47], may exhibit less pronounced and persistent transgenerational effects. This difference could be attributed to the transient nature of mucosal changes compared to the role of cecal tonsils in establishing long-lasting immune memory. Additionally, the differences in gene expression profiles in both tissues, despite the same epigenetic stimulation, may be due to the tissue-specific nature of epigenetic regulation [48]. For instance, in mice, developmental exposure to diethylstilbestrol (DES) induces distinct, tissue-specific patterns of DNA methylation and histone modifications in seminal vesicles and uterine tissues, driving differential gene expression and resulting in unique phenotypic outcomes [49]. This reflects the crucial role of tissue-specific epigenetic regulation in driving the observed intergenerational and transgenerational gene expression patterns.

Our findings demonstrate that the in ovo stimulation of F1 embryos with bioactive compounds can induce dynamic, non-linear intergenerational and transgenerational shifts in both cecal tonsil and cecal mucosal tissues [4].

### 4.1. Cecal Tonsils

The results of DEGs and enrichment analysis in the cecal tonsils seem to support our hypothesis that even a single in ovo injection of synbiotic or synbiotic + choline is able to induce potential epigenetic effects on immune-related tissues, which impacts not only the exposed individual’s transcriptome but has the potential to modulate gene expression in generation F3. It is well established that embryos containing primordial germ cells (PGCs)—the precursor cells that give rise to the germline cells— are sensitive to external factors, which can introduce epigenetic marks resulting in altered gene expression of selected genes [5].

Interestingly, the whole-genome gene expression did not differ between single-injection groups (SYNs and SYNCHs) and control in F2. However, the effect in F3 was well observed. This interesting observation may be explained by “generational skipping”, a phenomenon in which epigenetic modifications regulating gene expression are inherited across generations but may not manifest consistently in each. A study by Weber-Stadlbauer et al. [50] provides evidence for generational skipping in the context of transgenerational inheritance in mice. The research found that increased behavioral despair emerged in the F2 and F3 offspring of immune-challenged ancestors but not in the direct F1 descendants. This suggests that the generation F1 may act as a “silent carrier” of certain traits, which do not manifest until later generations. This also suggests that certain effects of prenatal immune activation may skip a generation, becoming latent and potentially re-emerging under specific environmental conditions or in later generations. In our study, the generation F2 may similarly act as a “silent carrier”. This pattern of inheritance is similar to other studies where a “silent carrier” phenomenon has been observed in response to chronic stress exposure [51,52]. Furthermore, the observed decrease in the number of DEGs from generation F1 to F2 could also be attributed to a “washout” effect [4]. On the other hand, the DEG increase in the generation F3 could be due to additive effects or shifts in environmental conditions (e.g., season) that reintroduce or amplify the initial epigenetic signals. In our study, generations F1 and F3 experienced similar conditions, being reared in autumn–winter season, while the F2 birds were raised during the spring–summer season. We suppose that this shift back to autumn–winter in generation F3 may potentially trigger a resurgence in gene expression effects.

Our hypothesis is further supported by the enrichment of GO terms and KEGG pathways in the cecal tonsils, which correspond to the specific treatments administered in each group. In the synbiotic groups, these enrichments are attributed to the effects of synbiotics alone, while in the synbiotic + choline groups, they reflect the combined influence of synbiotics and choline. While some enriched terms and pathways were consistently affected across generations in each group, other pathways and terms appeared uniquely in specific generations. For instance, within the SYNCH group, in KEGG pathway analysis, the phagosome pathway was enriched only in the F1 generation, while the ribosome pathway was exclusively affected in the F3 generation. In the SYN group, the KEGG pathway of ABC transporters was enriched in F1 but not in subsequent generations. Conversely, in the SYNr group, the KEGG pathway of endocytosis was uniquely enriched in the F3 generation and absent in earlier generations. This observation aligns with findings from other studies. For instance, Beck et al. demonstrated that while certain epigenetic marks, such as differentially methylated regions (DMRs), are transmitted across generations, distinct epimutations were observed in each generation in response to the epigenetic stimulation [53]. In their study, generation F3 exhibited a more integrated and overlapping epigenetic profile compared to the earlier generations. This included a higher overlap of DMRs with differentially hydroxymethylated regions (DHRs) and non-coding RNAs (ncRNA), suggesting a cumulative effect of epigenetic alterations over generations. Their findings indicate that the epigenetic landscape of generation F3 may be more complex and impactful for transgenerational inheritance.

Among the top ten enriched BPs in the synbiotic-injected groups are those related to cation homeostasis, which was seen in F1 SYN and then in F3 SYNs. Indeed, probiotics within synbiotics stabilize intestinal microbiota, which is essential for maintaining cation homeostasis [54]. This stabilization helps reduce toxic metabolites, protect the gut lining, and improve the absorption and regulation of ions like calcium and magnesium [54]. Additionally, synbiotics may influence the host’s ionic balance by affecting cation transport and homeostasis mechanisms [55]. We also observed effects of the F3 SYNs group on BPs related to monocarboxylic acid metabolism, ATP metabolism, and small-molecule metabolism. This is probably related to synbiotics’ ability to increase the production of short-chain fatty acids like acetate, butyrate, and propionate, which are crucial for energy metabolism and gut health [56]. By modulating the gut microbiota, synbiotics enhance the biosynthesis of small molecules, contributing to better metabolic health and a reduced risk of metabolic disorders [57]. Moreover, in our study in the F3 SYNr group, pathways related to pyruvate metabolism, nucleotide diphosphate metabolism, and purine nucleoside diphosphate metabolism were enriched. Synbiotics have been shown to increase bacterial-derived metabolites, including pyruvate, enhancing metabolic pathways [58]. Synbiotic modulation of gut microbiota also upregulates key pathways involved in carbohydrate, nucleotide, and amino acid metabolism, essential for growth and immune responses [59]. The enriched CCs in SYN groups included the endoplasmic reticulum membrane, extracellular organ, cytosol, organelle membrane, and extrinsic component of the membrane. Synbiotics modulate the gut microbiome, influencing cellular compartments and improving nutrient absorption and immune responses [60]. They enhance intestinal barrier function by modulating cytoskeletal and tight junctional protein phosphorylation [60]. In our study, the glutamatergic synapse was enriched in both F1 and F3 SYN groups, with synbiotics affecting glutamatergic neurotransmission and potentially influencing mood, behavior, and stress responses [61]. Prebiotics like galacto-oligosaccharides also enhance glutamatergic signaling, with long-term benefits of early-life prebiotic supplementation [62]. In terms of MFs, we observed enrichment of synbiotic-injected groups in transmembrane transporter activity, which is important for nutrient absorption [63]. Moreover, synbiotics can enhance membrane fluidity and transporter function [64]. In the F3 SYNr group, we observed that enriched functions included chemoattractant activity and chemokine receptor binding, influenced by synbiotics modulating gut microbiota and short-chain fatty acid production [56]. Synbiotics may also regulate the CCR6 receptor, important for mucosal immunity, through microbiota modulation [65].

In the F3 SYNCHs group, synbiotics affected gene expression in cecal tonsils, influencing BPs related to protein synthesis, peptide metabolism, and cellular amide processes. Choline, a key component of cell membranes, plays a role in maintaining cellular homeostasis and protein synthesis through methylation processes [66]. Synbiotics also alter gut microbiota composition, improving nutrient absorption, including amino acids, and biosynthetic processes [39]. We observed that, in the F3 SYNCHr group, PS synbiotic influenced cellular metabolism, including pyruvate metabolism, nucleoside phosphorylation, and ATP generation. Choline plays a key role in lipid metabolism, energy balance, and nucleotide metabolism, supporting processes like ATP generation and nucleotide phosphorylation through its involvement in phosphatidylcholine synthesis and as a precursor for S-adenosylmethionine [67]. In both SYNCHs and SYNCHr groups of F3, MFs related to translation regulation and initiation factor activity were observed. Choline is essential for ribosomal integrity, particularly in the intestinal mucosa, and its deficiency impairs ribosomal function [68]. Choline supplementation restores polysome profiles and enhances protein synthesis by supporting ribosomal membrane binding and aggregation [68].

In synbiotic-injected groups, particularly in F1 and F3, we observed significant enrichment in metabolic pathways including retinol and steroid hormone metabolism, drug metabolism, and cytochrome P450 pathways. Synbiotics influence gut microbiota, aiding in the conversion of vitamin A [69] and steroid hormone metabolism [70]. Probiotics have also been shown to alter the expression of cytochrome P450 (CYP) enzymes throughout the gastrointestinal tract [71]. Moreover, our analysis revealed enrichment of the PPAR signaling pathway in F1, F2, and F3 SYNs groups. Indeed, synbiotics were shown to activate the PPAR signaling pathway, reducing neuroinflammation [72]. Fructose and mannose metabolism pathways were enriched in F1 SYN and F3 SYNr, as synbiotics can modulate the host’s biochemistry, lipid, carbohydrate, and amino acid metabolism [73,74]. The F3 SYNs and SYNr groups also shared common metabolic pathways such as oxidative phosphorylation and glycolysis/gluconeogenesis. Probiotics have been shown to alter carbon metabolism through phosphorylation and glycolysis [75]. Synbiotics also reduce oxidative stress markers and increase antioxidant levels, enhancing oxidative phosphorylation efficiency by protecting mitochondria from oxidative damage [76].

In the SYNCH groups, we observed enrichment of pathways such as phagosome and lysosome pathways in F1. Lysophosphatidylcholine (LPC), a choline derivative, enhances phagosome maturation and bactericidal activity, indicating a role for choline metabolites in immune responses [77]. Moreover, in our study, F1 and F3 SYNCHs groups revealed enrichment in KEGG pathways related to cytokine–cytokine receptor interactions. Synbiotics have been shown to reduce inflammatory markers in intestinal models, which could affect cytokine signaling pathways [78]. They may also boost the gut microbiota’s ability to process choline, potentially altering inflammatory metabolite production through cytokine modulation [57,79]. Additionally, carbon metabolism pathways were enriched in our study in F2 SYNCHr and F3 SYNCHr groups. Choline plays a role in one-carbon metabolism, serving as a precursor to betaine, which is involved in the methylation of homocysteine to methionine, a key process in one-carbon metabolism [80]. In F3, both SYNCH and SYNCHr groups showed a resurgence of enriched pathways, particularly the ribosome pathway. The gut microbiome impacts protein synthesis, cellular homeostasis, and stress responses [81]. Choline is essential for phospholipid synthesis, which maintains cell membrane integrity and supports ribosome function for efficient protein synthesis [79].

Our findings are based on the whole-genome gene expression. Genome-wide studies have an advantage over single-gene expression because they allow the study of multiple genes and pathways. They are also a good tool in exploratory studies like the one we present in this work. In our study, a genome-wide approach allowed us to observe complex effects of injected substances on the transcriptome of the cecal tonsil tissue in a three-generational context. We found several proofs for the impact of in ovo synbiotic and choline stimulation on the cecal tonsil transcriptome. The effective action of the injected substances was also observed through their influence on the specific GO terms and KEGG pathways, which are related to previously observed biochemical and physiological effects of these substances on the organism. Our results indicate the potential of in ovo synbiotic and choline injections to modulate the transcriptome of adult chicken cecal tonsils, as well as their potential to influence the tissue transcriptome in subsequent generations.

### 4.2. Cecal Mucosa

In the cecal mucosa, another scenario of transgenerational dynamics was observed, where an initial increase is followed by a “washout” effect [4]. The change becomes more pronounced in F2 but then starts to recede, highlighting the non-linear nature of epigenetic effects across generations [4]. Except for the SYNs group, the number of DEGs decreased in F2 then increased in F3 to the same level as in F1. Research on the transgenerational effect of glyphosate exposure demonstrated negligible impacts on the generations F0 and F1, but a significant effect emerged in the generation F2 [82]. By the generation F3, some of these effects persisted, though with variations; certain effects seen in the generation F2 decreased or no longer appeared in the generation F3, while others continued to manifest [82]. These findings collectively underscore the complexity and non-linear nature of epigenetic inheritance. While the pattern observed in the glyphosate study differs from ours, it supports the overarching idea that transgenerational effects are dynamic and may emerge, diminish, or reappear in subsequent generations. This aligns with our findings, which show that environmental exposures can trigger epigenetic modifications with variable impacts across generations, highlighting their unpredictable and evolving nature.

Similar to cecal tonsils, we observed enriched GO terms and KEGG pathways which were related to the synbiotic and choline. In the F1 SYN group, synbiotics primarily enhance catabolic and metabolic processes. Probiotics break down complex carbohydrates into simpler sugars, which are then fermented into short-chain fatty acids [83,84]. Using specialized transport systems and enzymes, these bacteria metabolize prebiotics, supporting overall gut catabolic activity [84]. In the F2 SYNs and F2 SYNr groups, we observed a notable shift towards cell-cycle-related processes, as synbiotics improve gut barrier function by decreasing gut permeability and reinforcing intestinal wall integrity [85]. This enhancement reduces the likelihood of pathogen translocation and inflammation, supporting regulated cell proliferation and potentially reducing disease risk [86]. Additionally, we showed that immune-system-related processes were enriched in the F3 SYNs group. Indeed, synbiotics are well known to increase both innate and adaptive immunity by stimulating natural killer cells, macrophages, antibody production, and T-cell responses [87]. The interaction of probiotics with intestinal cells induces cytokine production, helping to balance pro- and anti-inflammatory responses in the gut [87]. Moreover, we found that F2 SYNs and SYNr groups exhibited enrichment in chromosomal components like chromosomes and heterochromatin among other CC terms. This is probably due to synbiotics’ ability to support gut health, which may improve chromosomal stability by reducing inflammation and oxidative stress, thus helping to prevent DNA damage [88]. This protective effect suggests synbiotics could play a role in maintaining DNA integrity and managing conditions like colorectal cancer [88]. Regarding MFs in our study, the F1 SYN and F3 SYNr groups showed enriched kinase and phosphotransferase activities. Previously, probiotics have been shown to influence adenosine-monophosphate-activated protein kinase (AMPK) activity [89]. Prebiotics can enhance intestinal barrier integrity through protein kinase C (PKC)-dependent mechanisms [90]. Moreover, phosphotransferase enzyme activity can be affected by substrate availability and specific bacterial strains, both of which synbiotic supplementation can modulate [91]. We also observed that both F1 SYN and F2 SYNs groups show purine-ribonucleoside-triphosphate-binding enrichment, which can be influenced by probiotics’ effects on purine metabolism, affecting the availability and binding of purine ribonucleoside triphosphates [92].

In synbiotic + choline groups, we showed a slightly different profile of enriched GO terms in comparison with the SYN group. For instance, the F1 SYNCH group was enriched in BPs related to responses to reactive oxygen species (ROS). This is in line with results of other studies in which synbiotics were found to enhance antioxidant enzyme activity, which helps mitigate oxidative stress [93,94]. We also observed that BPs related to cell adhesion were enriched in both F2 SYNCHs and SYNCHr. Choline phosphate was reported to promote cell adhesion [95], and synbiotics are also known to improve bacterial adhesion to host cells [96]. Moreover, the F1 SYNCH group showed enrichment in CCs related to the apical cell region and organelle membranes. Synbiotics may improve gastrointestinal barrier integrity by influencing tight junctions between epithelial cells, which helps maintain the apical environment and prevent pathogen translocation [83]. In poultry, choline-enriched probiotics have been shown to enhance intestinal histological parameters, such as villus length and crypt depth, indicating better nutrient absorption and gut health [84]. In our study, the F2 SYNCHs group revealed enrichment in MFs such as glycosaminoglycan binding. Choline is essential for lipid metabolism and DNA methylation, influencing cellular interactions with glycosaminoglycans [97]. Additionally, synbiotics were found to influence lipid profiles, which can indirectly affect glycosaminoglycan interactions [98]. We also showed that the F2 SYNCHr group was enriched in ion binding and hydrolase activity functions. Choline transport in the intestine involves a carrier-mediated system that may interact with cation-binding sites [99]. Synbiotics can affect hydrolase activity, such as the bile salt hydrolase activity, which plays a key role in cholesterol metabolism [100].

In our study, KEGG pathway enrichment analysis in synbiotic groups across generations F1, F2, and F3 highlighted a strong focus on metabolism. This result is in agreement with the findings of other authors. For instance, synbiotic interventions have been shown to reverse high-fat-diet-induced changes in microbial populations, enhancing beneficial species while reducing harmful ones, which improves metabolic parameters like reduced body weight gain and glucose and lipid metabolism [101,102]. A study on diet-induced obese mice has demonstrated that synbiotics can regulate glucose metabolism by modulating the insulin–IGF-1 signaling pathway through the overexpression of glucose transporters GLUT-1 and GLUT-4, which are essential for glucose uptake and metabolism [103]. Additionally, synbiotic supplementation in obese individuals has resulted in significant improvements in obesity-related biomarkers, including reductions in cholesterol and cytokines, highlighting their positive effects on metabolic pathways linked to lipid metabolism [104].

In the synbiotic + choline-injected groups, we observed a more diverse set of enriched pathways across generations. KEGG pathways related to metabolism were enriched in each group and each generation. In addition to the above-described effect of synbiotics on gut tissue metabolism, choline plays a critical role in lipid metabolism, particularly in lipoprotein synthesis and secretion [105]. Choline deficiency impairs intestinal lipid metabolism, leading to reduced plasma triacylglycerol and cholesterol levels and altered intestinal morphology, affecting fat absorption [105]. Besides metabolic pathways, the F2 SYNCHs group showed enrichment in ECM–receptor interaction and muscle cell cytoskeleton, which was also seen in the SYNCHr group. Probiotics can modulate immune responses in cecal tonsils, potentially affecting ECM–receptor interactions through changes in cytokine expression and immune cell activity [106]. The bioavailability of choline and its conversion to trimethylamine-*N*-oxide can influence intestinal health and disease, impacting ECM–receptor interactions via changes in cellular communication and immune responses [79]. Additionally, synbiotics can enhance intestinal villi height and surface area, which could indirectly affect muscle cell cytoskeletons [107]. Synbiotic supplementation also increased tight junction protein expression, such as Claudin-1 and Occludin, critical for the intestinal barrier and cytoskeletal dynamics [108]. In broiler chickens, choline combined with probiotics can improve intestinal histological parameters, potentially enhancing the structural integrity of intestinal and muscle cells in the cecal tonsils [109].

In both tissues, we observed a higher number of implicated genes within the potentially affected GO terms and KEGG pathways in the repeated injection groups. This finding aligns with our expectations, suggesting a cumulative effect of synbiotic injections across successive generations.

While this study involving bioactive compounds’ effects on immune tissue transcriptomes was conducted using a chicken model, the findings provide valuable insights into epigenetic mechanisms and their transgenerational effects that are broadly applicable across vertebrate species, including humans. Epigenetic regulatory processes, such as DNA methylation, histone modification, and non-coding RNA activity, are conserved across vertebrates [110]. These mechanisms underpin the ability of environmental factors, including nutrition, to modulate gene expression [111]. The controlled nature of the chicken model allows for precise examination of these processes, offering a foundational understanding that can inform studies in humans [112]. Similar to the in ovo injections used in this study, early-life nutritional interventions in humans—such as maternal dietary supplementation during pregnancy—are known to influence offspring health [42]. For instance, studies have demonstrated how maternal intake of methyl-group donors, including folate and choline, can modulate epigenetic markers associated with immune and metabolic functions [113]. These parallels suggest that the bioactive compounds used in our study could have analogous effects in humans, warranting further investigation. Human studies, such as the Dutch Hunger Winter cohort, have shown that prenatal exposure to environmental factors can result in epigenetic modifications that persist across generations [114]. Our findings align with this phenomenon, demonstrating that nutritional stimulation during embryonic development can lead to both inter- and transgenerational effects on gene expression.

## 5. Conclusions

To the best of our knowledge, this study is the first to use a chicken model for a transgenerational experiment on the impact of bioactive compounds on immune system tissues transcriptomes. Our findings contribute to the growing body of evidence suggesting that dietary and environmental factors can influence gene expression across multiple generations. We observed that PS synbiotic and choline supplementation affected gene expression in both the cecal tonsils and cecal mucosa, with distinct effects on each tissue. The synbiotic- and synbiotic + choline-injected groups demonstrated transgenerational influences on gene expression, although the patterns varied. In the cecal tonsils, the reappearance of effects in the generation F3, after a skipped effect in F2, highlights the complex interplay between epigenetic mechanisms and environmental factors. This underscores the importance of considering the potential for latent effects to be reactivated under changing conditions. In the cecal mucosa, the results suggest that induced epigenetic modifications can trigger transgenerational effects that are not uniform or predictable, with some impacts emerging or diminishing in subsequent generations. These findings emphasize the need for further research into the complex epigenetic mechanisms through which epigenetic factors influence gene expression across generations.

## Data Availability

All data are available in the text and in Supplementary Files. The raw sequencing data, formatted as fastq files, have been deposited into the National Center for Biotechnology Information (NCBI). Accession numbers for the Sequence Read Archive (SRA) data: PRJNA1180491 (for cecal mucosa) and PRJNA1179951 (for cecal tonsils).

## Supplementary Materials

The following supporting information can be downloaded at: https://www.mdpi.com/article/10.3390/ijms26031174/s1, References [115,116] are cited in the supplementary materials.

## Abbreviations

PS	Synbiotic PoultryStar^®^ (Biomin)
NaCl	Physiological saline
F0	Parental generation
F1	First generation
F2	Second generation
F3	Third generation
C	Control group
SYN	Bird group receiving in ovo injection of 2 mg synbiotic/embryo
SYNCH	Bird group receiving in ovo injection of synbiotic (2 mg) combined with choline (0.25 mg) per embryo
SYNs	Bird group receiving a single in ovo injection of 2 mg synbiotic/embryo in F1
SYNCHs	Bird group receiving a single in ovo injection of synbiotic (2 mg) combined with choline (0.25 mg) per embryo in F1
SYNr	Bird group receiving repeated in ovo injections of 2 mg synbiotic/embryo in F1, F3, and F3
SYNCHr	Bird group receiving repeated in ovo injections of synbiotic (2 mg) combined with choline (0.25 mg) per embryo in F1, F2, and F3
CD4+	Cluster of differentiation 4 positive (marker for helper T cells)
CD8+	Cluster of differentiation 8 positive (marker for cytotoxic T cells)
CD20+	Cluster of differentiation 20 positive (marker for B cells)
miRNA	MicroRNA
DNA	Deoxyribonucleic acid
RNA	Ribonucleic acid
KEGG	Kyoto Encyclopedia of Genes and Genomes
GO	Gene Ontology
BP	Biological process (Gene Ontology term)
CC	Cellular component (Gene Ontology term)
MF	Molecular function (Gene Ontology term)
RT-qPCR	Reverse transcription– quantitative polymerase chain reaction
cDNA	Complementary DNA
DEG	Differentially expressed gene
ATP	Adenosine triphosphate
PPAR	Peroxisome proliferator-activated receptor
ECM	Extracellular matrix
SRSF5	Serine- and arginine-rich splicing factor 5
LAMB2	Laminin subunit beta 2
PLA2G10	Phospholipase A2 group X
MVB12B	Multivesicular body subunit 12B
AWAT1	Acyl-CoA wax alcohol acyltransferase 1
RPS12	Ribosomal protein S12
ADH1C	Alcohol dehydrogenase 1C
ATP6V0A4	ATPase H+ transporting V0 subunit A4
ASS1	Argininosuccinate synthase 1
GSTA4	Glutathione S-transferase A4
FN1	Fibronectin 1
CCNB3	Cyclin B3
SCD	Stearoyl-CoA desaturase
ITGB3	Integrin beta 3
DES	Desmin
FABP1	Fatty-acid-binding protein 1
MCOLN3	Mucolipin TRP cation channel 3
SLC17A5	Solute carrier family 17 member 5
FABP2	Fatty-acid-binding protein 2
5azaC	5-azacytidine
PGC	Primordial germ cell
DMRs	Differentially methylated regions
DHRs	Differentially hydroxymethylated regions
ncRNA	Non-coding RNA
CCR6 receptor	C-C chemokine receptor type 6
CYP enzymes	Cytochrome P450 enzymes
AMPK	Adenosine-monophosphate-activated protein kinase
PKC	Protein kinase C
ROS	Reactive oxygen species
GLUT-1	Glucose transporter type 1
GLUT-4	Glucose transporter type 4
