# Supplementary material for: Inter- and Transgenerational Effects of In Ovo Stimulation with Bioactive Compounds on Cecal Tonsils and Cecal Mucosa Transcriptomes in a Chicken Model
Source: Int J Mol Sci. 2025 Jan 29;26(3):1174. doi: 10.3390/ijms26031174 (PMC11817890; doi:10.3390/ijms26031174)
Supplement: Supplementary file 1 [file ijms-26-01174-s001.zip › Supplementary file S14.pdf]

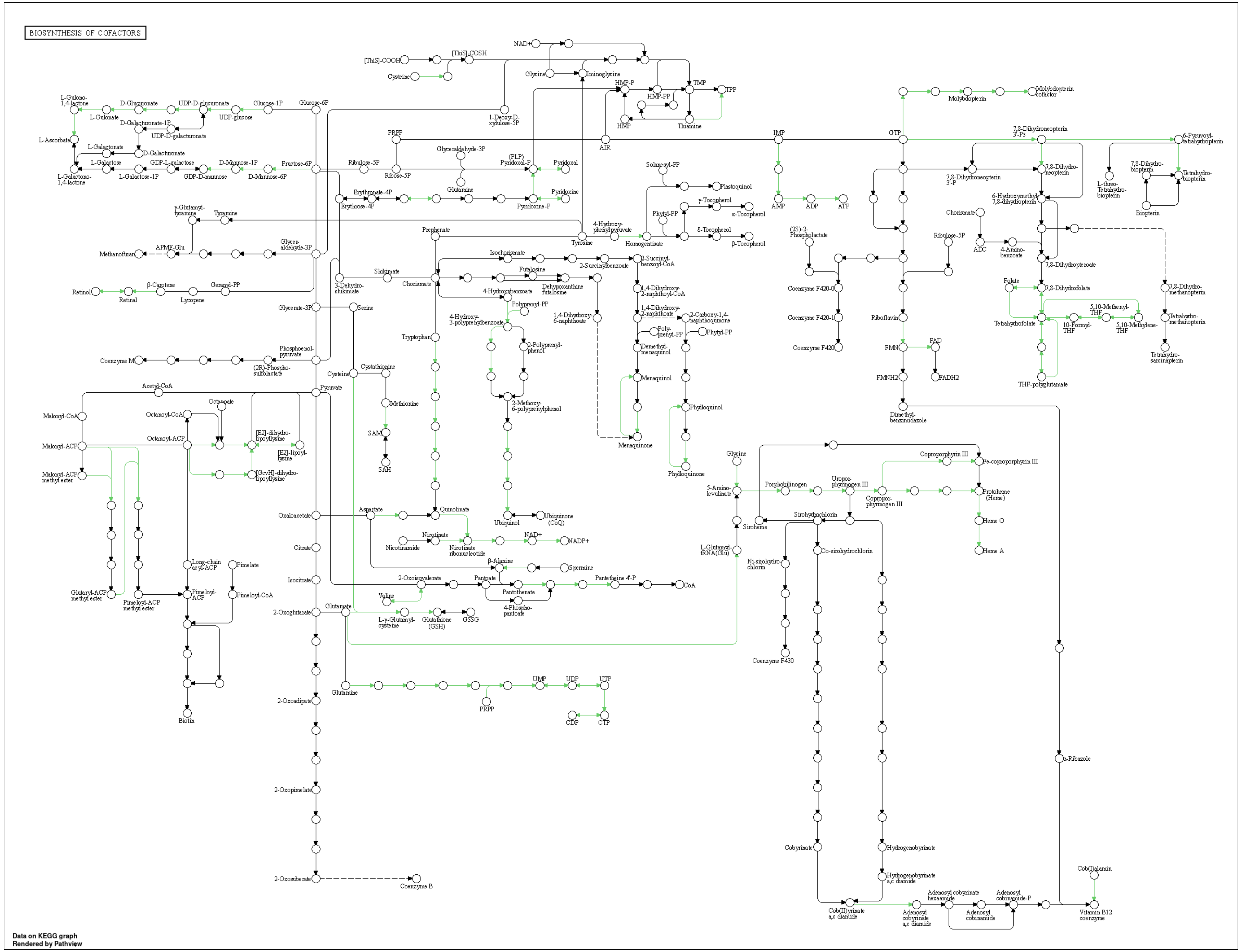

Figure S1. Biosynthesis of cofactors pathway in SYN group in F1 (Cecal tonsils)

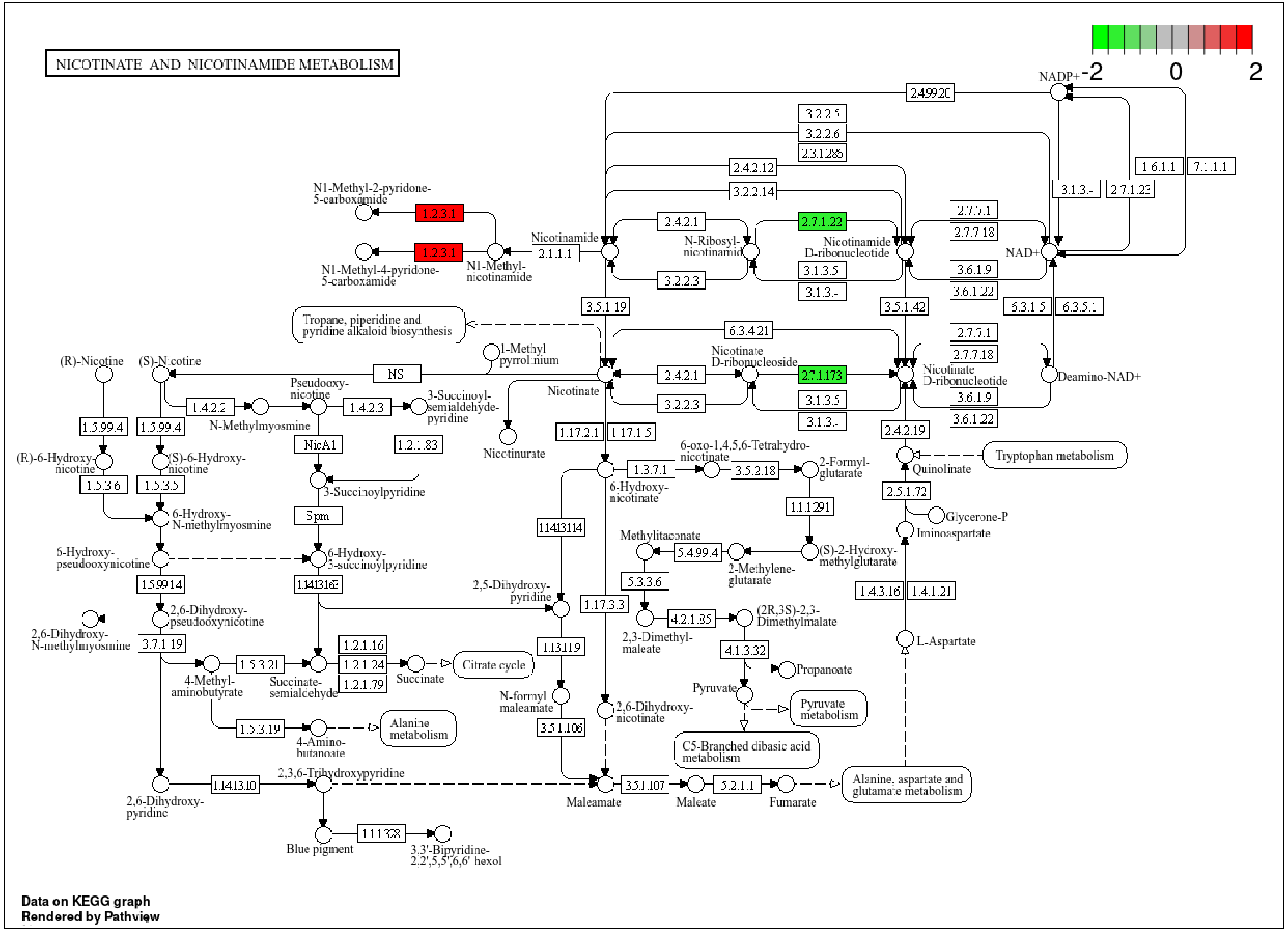

Figure S2. Nicotinate and nicotinamide pathway in SYN group in F1 (Cecal tonsils).

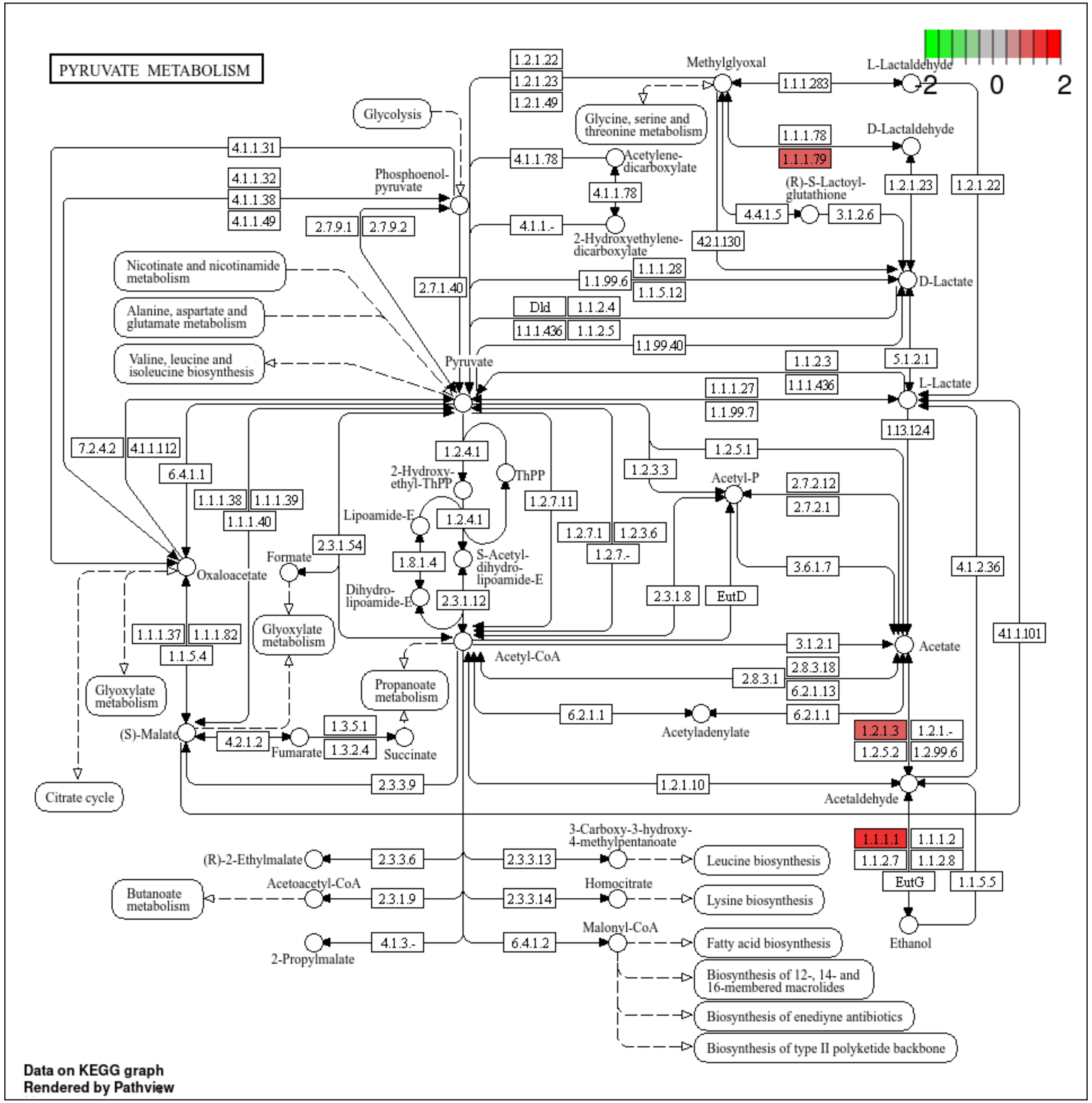

Figure S3. Pyruvate metabolism pathway in SYN group in F1 (Cecal tonsils).

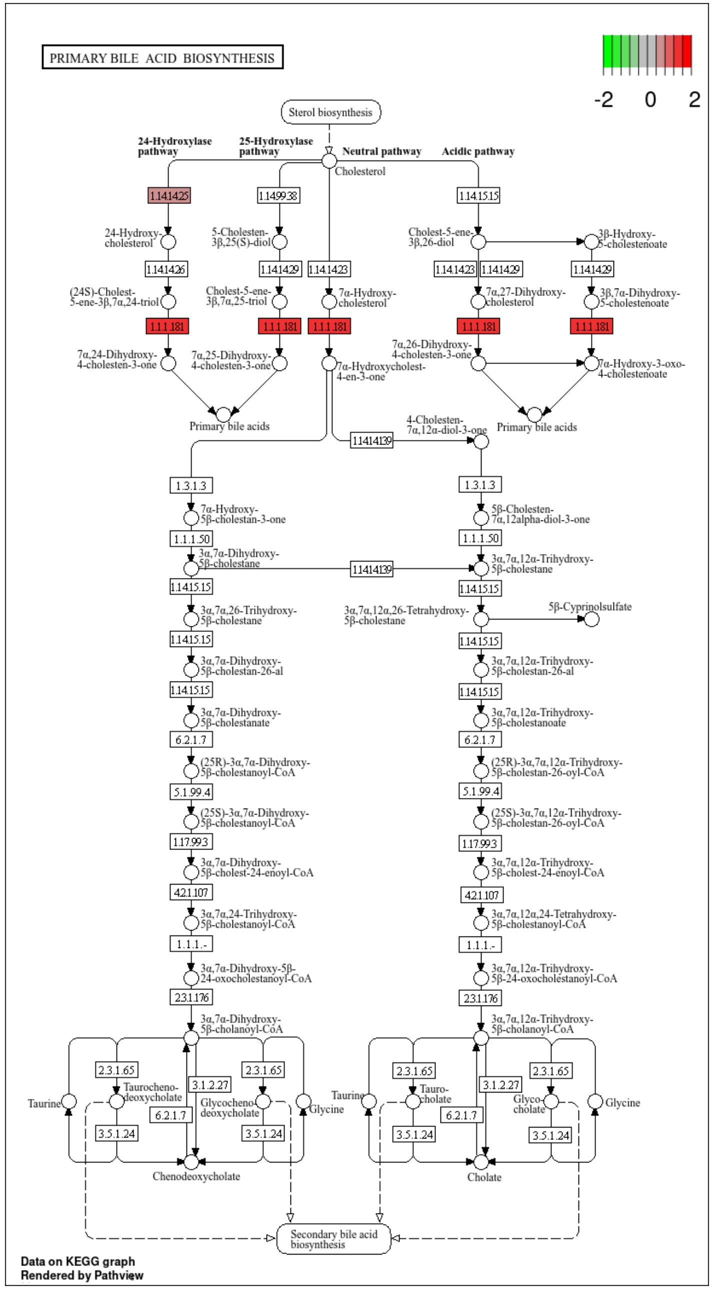

Figure S4. Primary bile acid biosynthesis pathway in SYN group in F1 (Cecal tonsils).

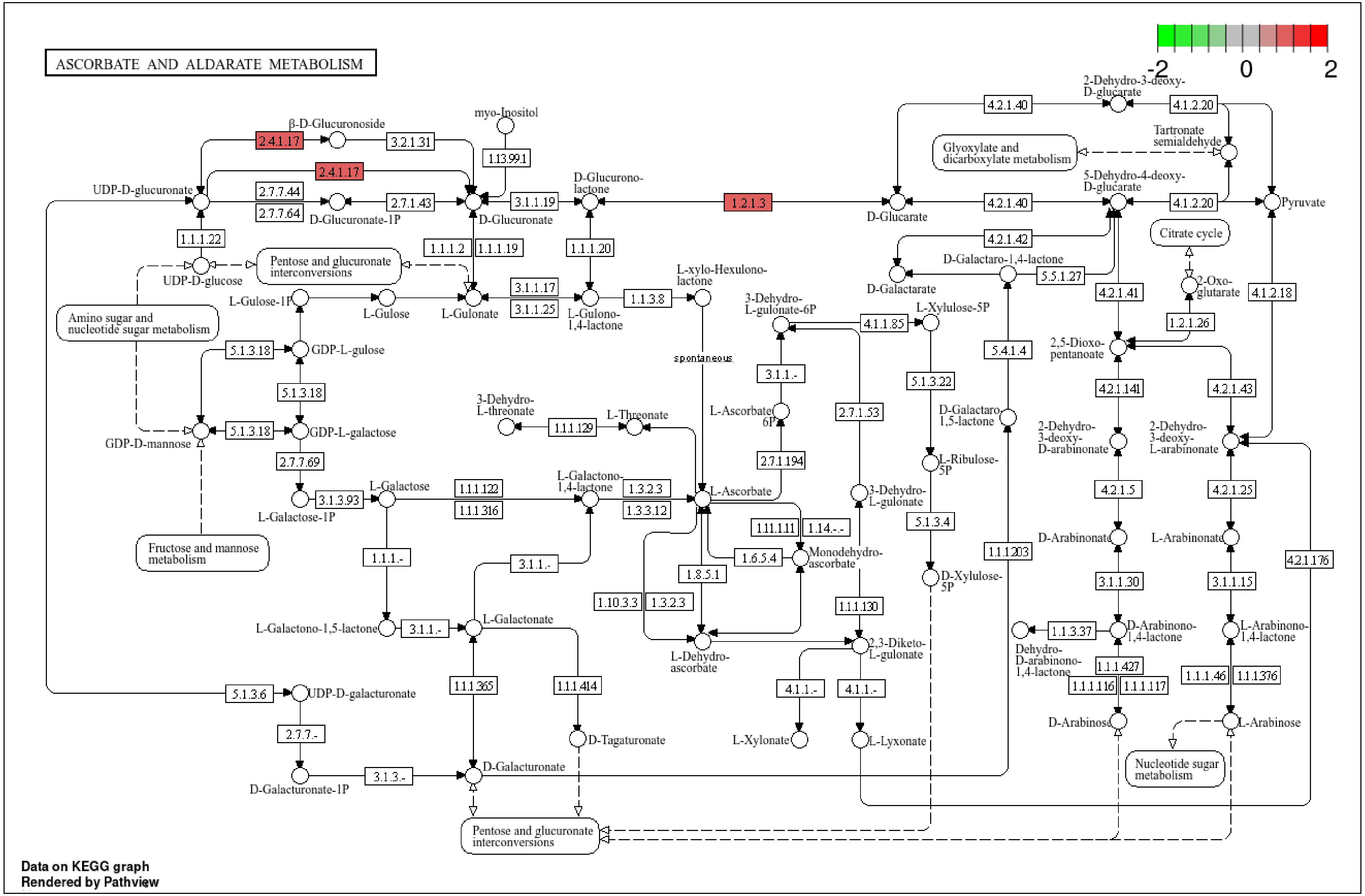

Figure S5. Ascorbate and aldarate metabolism pathway in SYN group in F1 (Cecal tonsils).

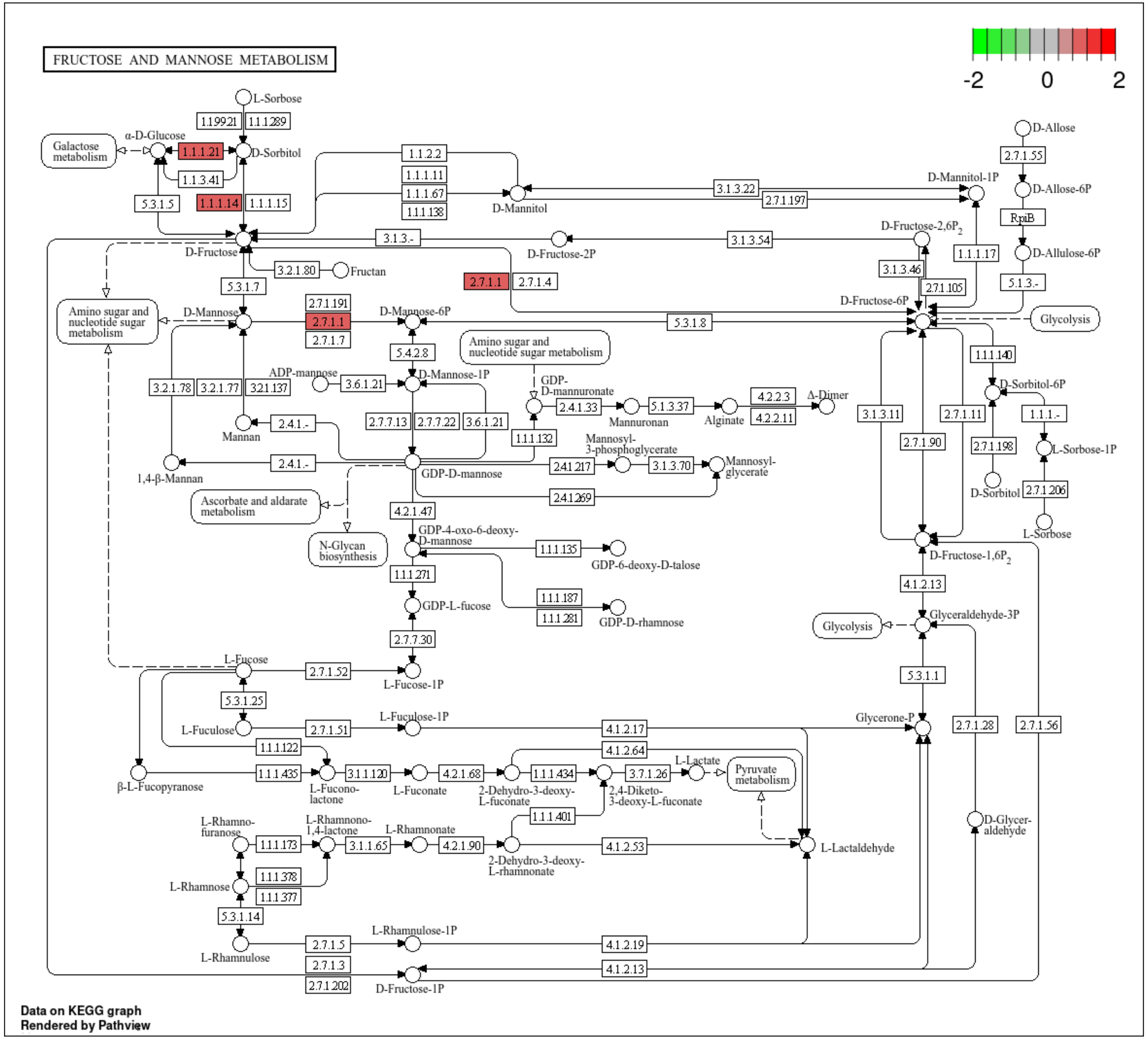

Figure S6. Fructose and mannose metabolism pathway in SYN group in F1 (Cecal tonsils)

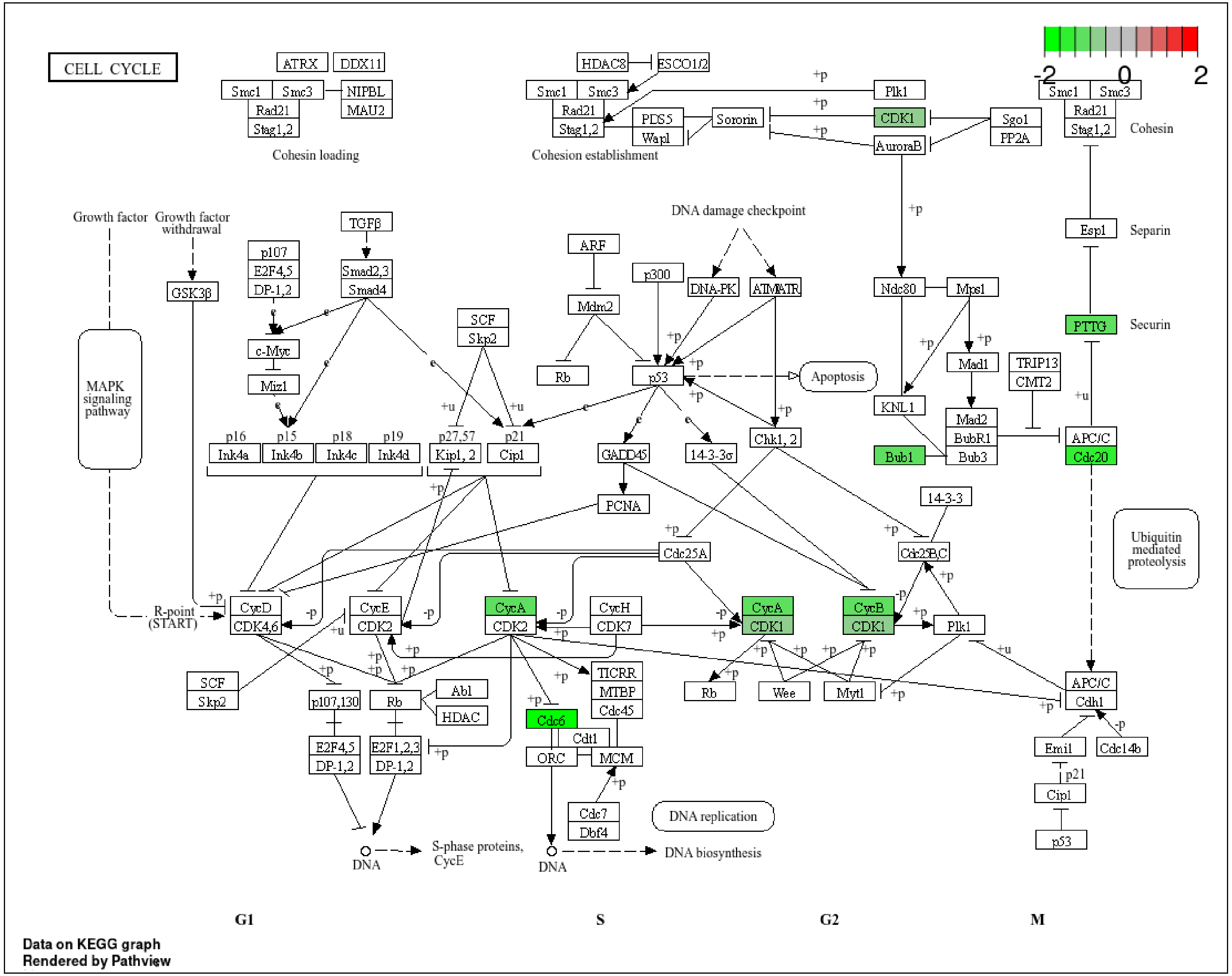

Figure S7. Cell cycle pathway in SYN group in F1 (Cecal tonsils).

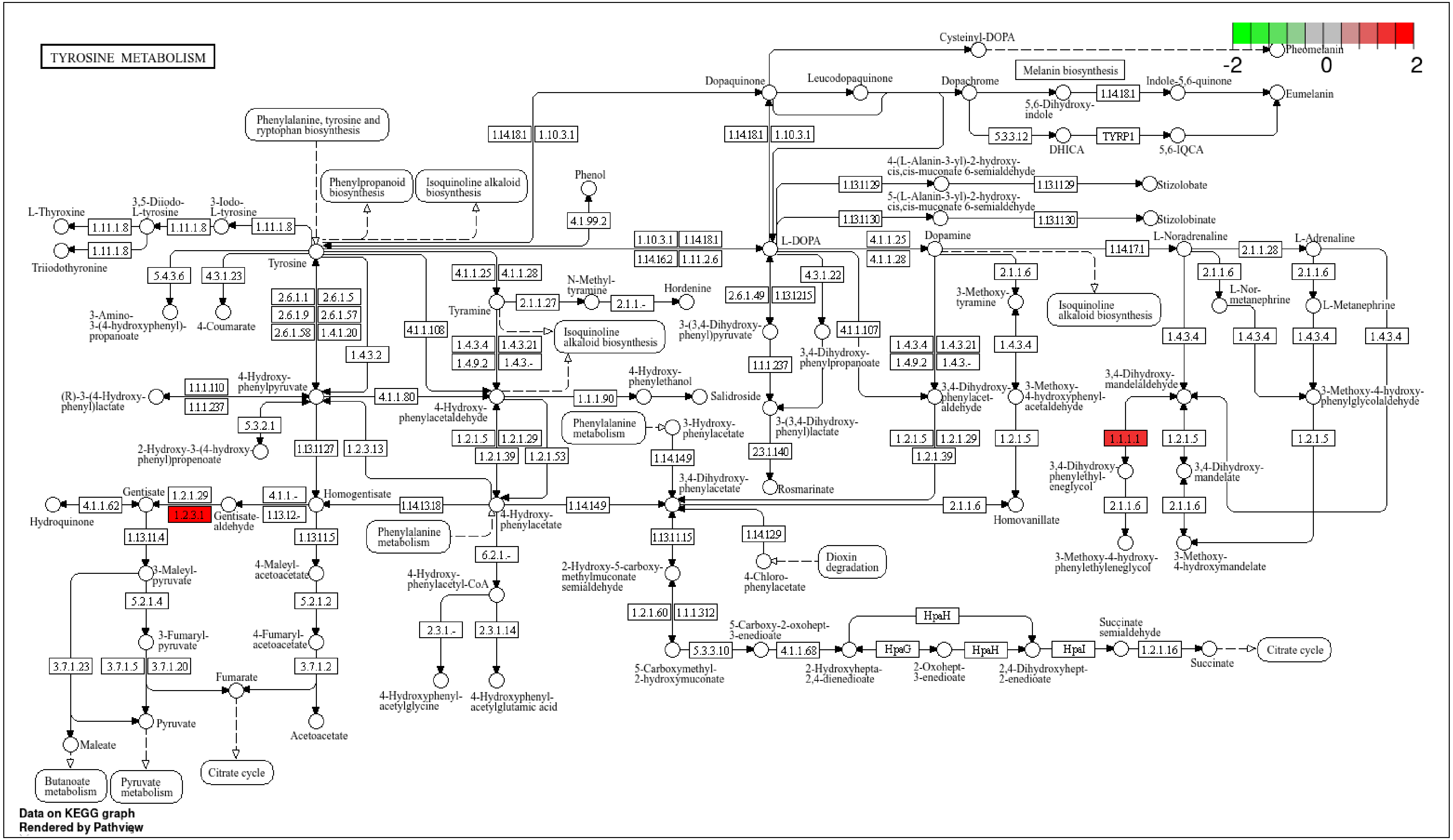

Figure S8. Tyrosine metabolism in SYN group in F1 (Cecal tonsils).

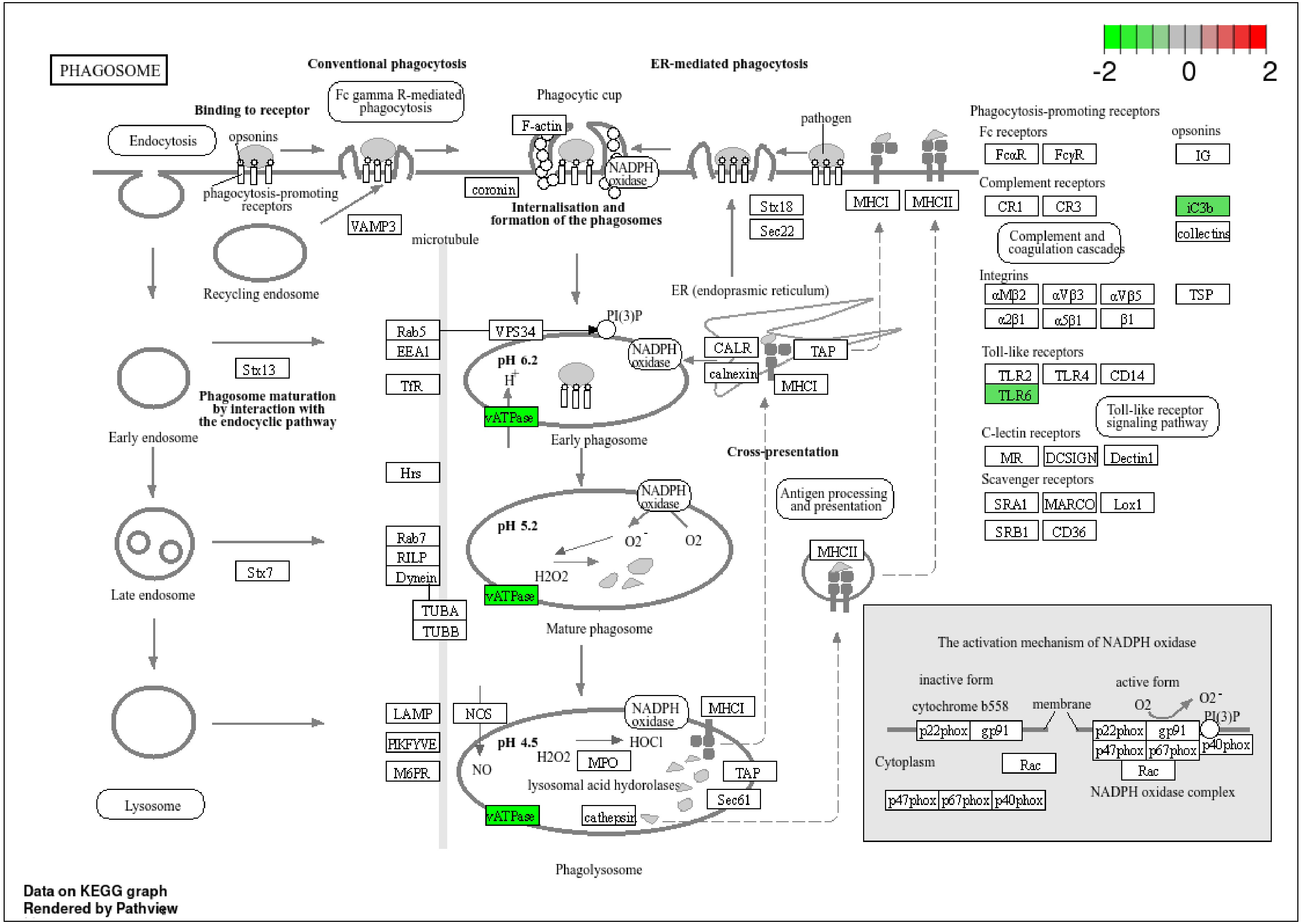

Figure S9. Phagosome pathway in SYN group in F1 (Cecal tonsils).

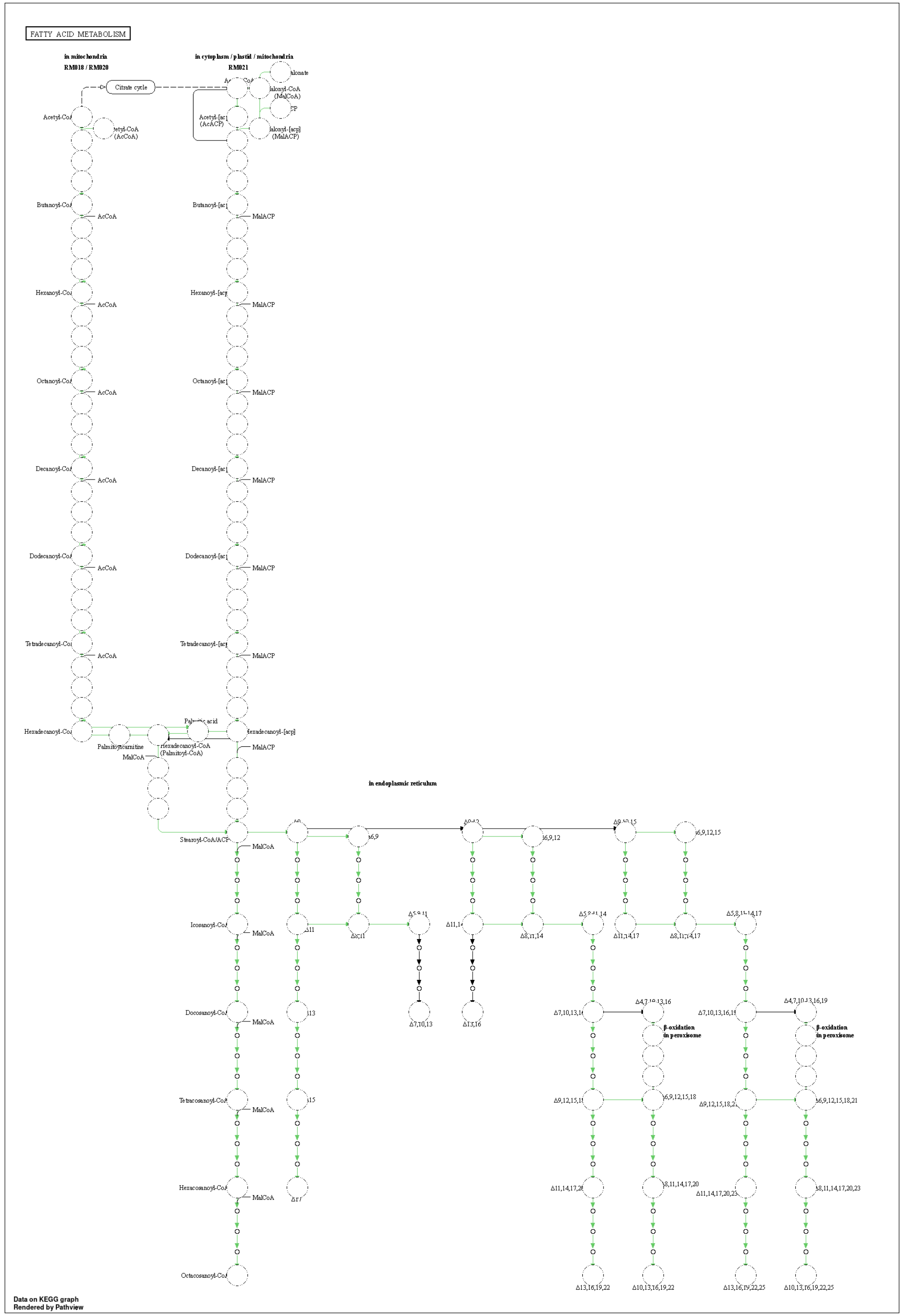

Figure S10. Fatty acid metabolism pathway in SYN group in F1 (Cecal tonsils).

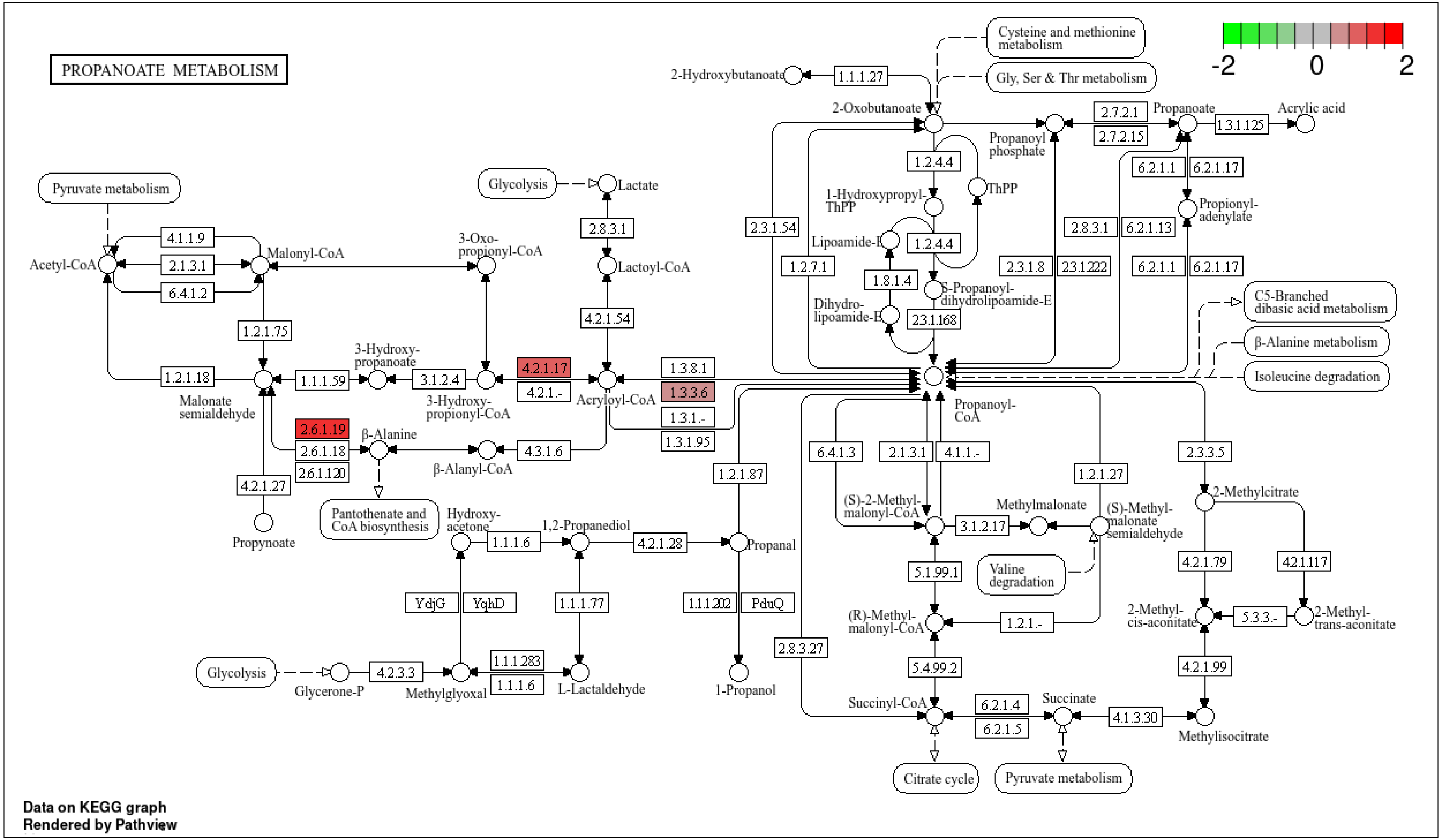

Figure S11. Propanoate metabolism pathway in SYN group in F1 (Cecal tonsils).

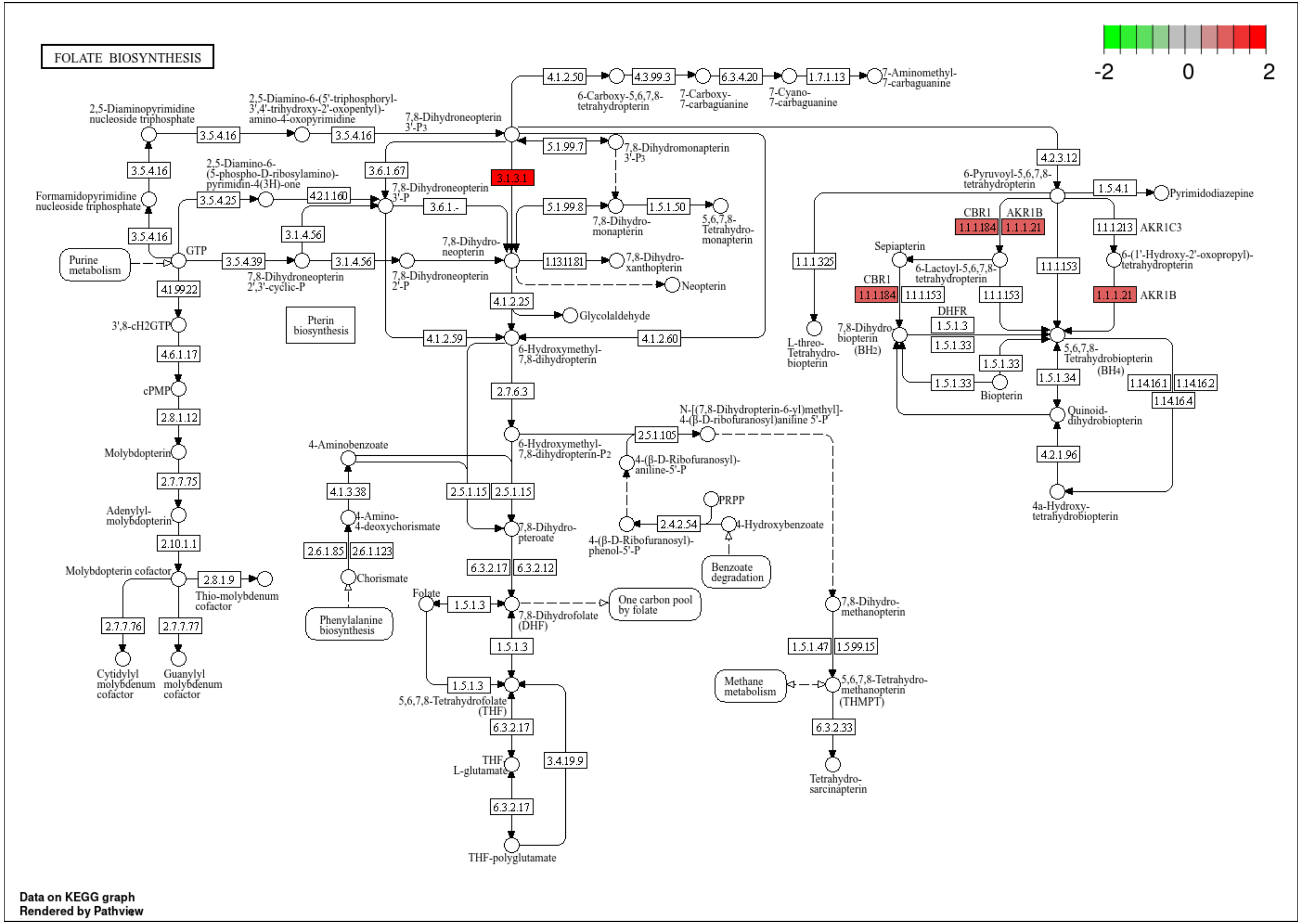

Figure S12. Folate biosynthesis pathway in SYN group in F1 (Cecal tonsils).

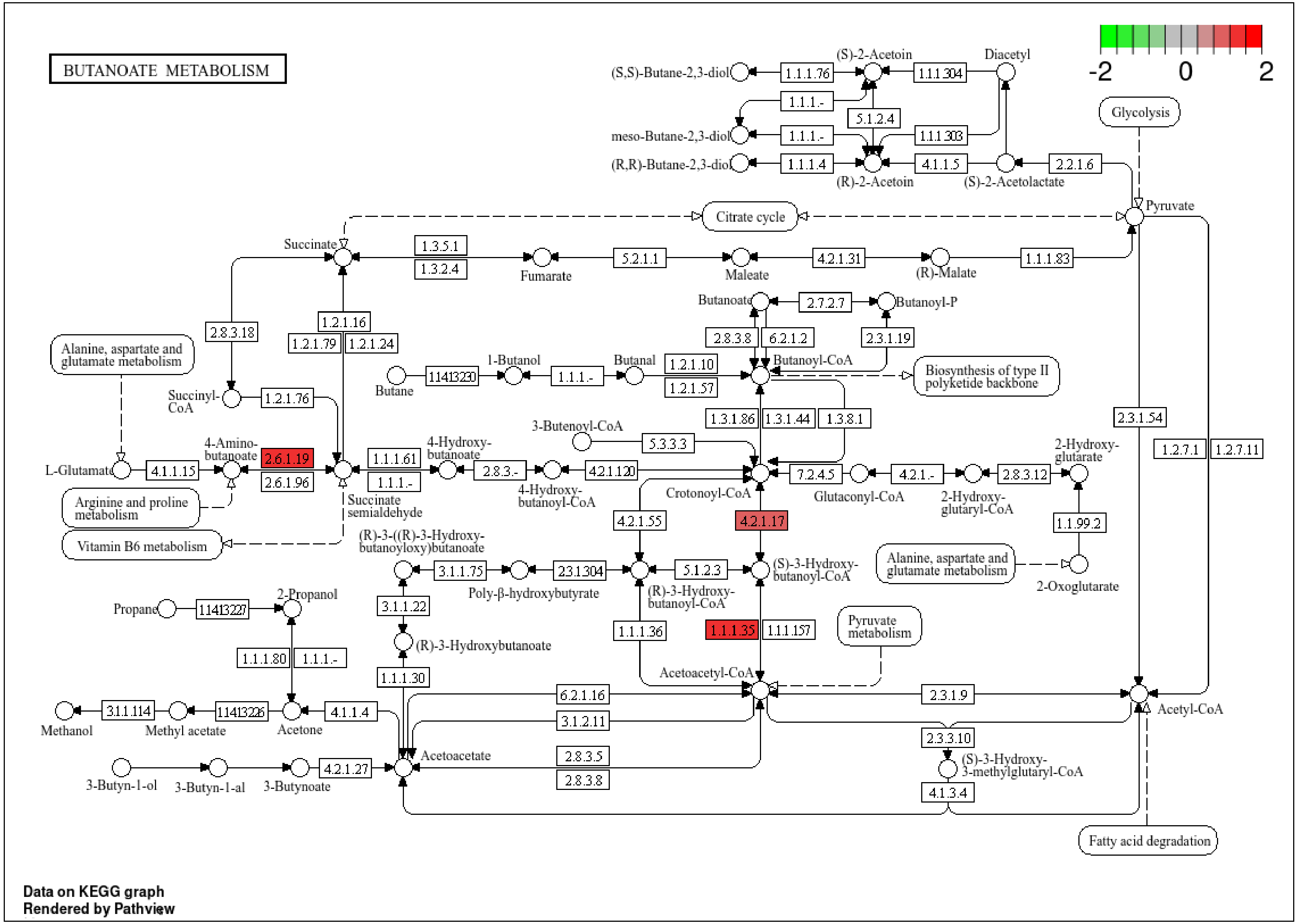

Figure S13. Butanoate metabolism pathway in SYN group in F1 (Cecal tonsils).

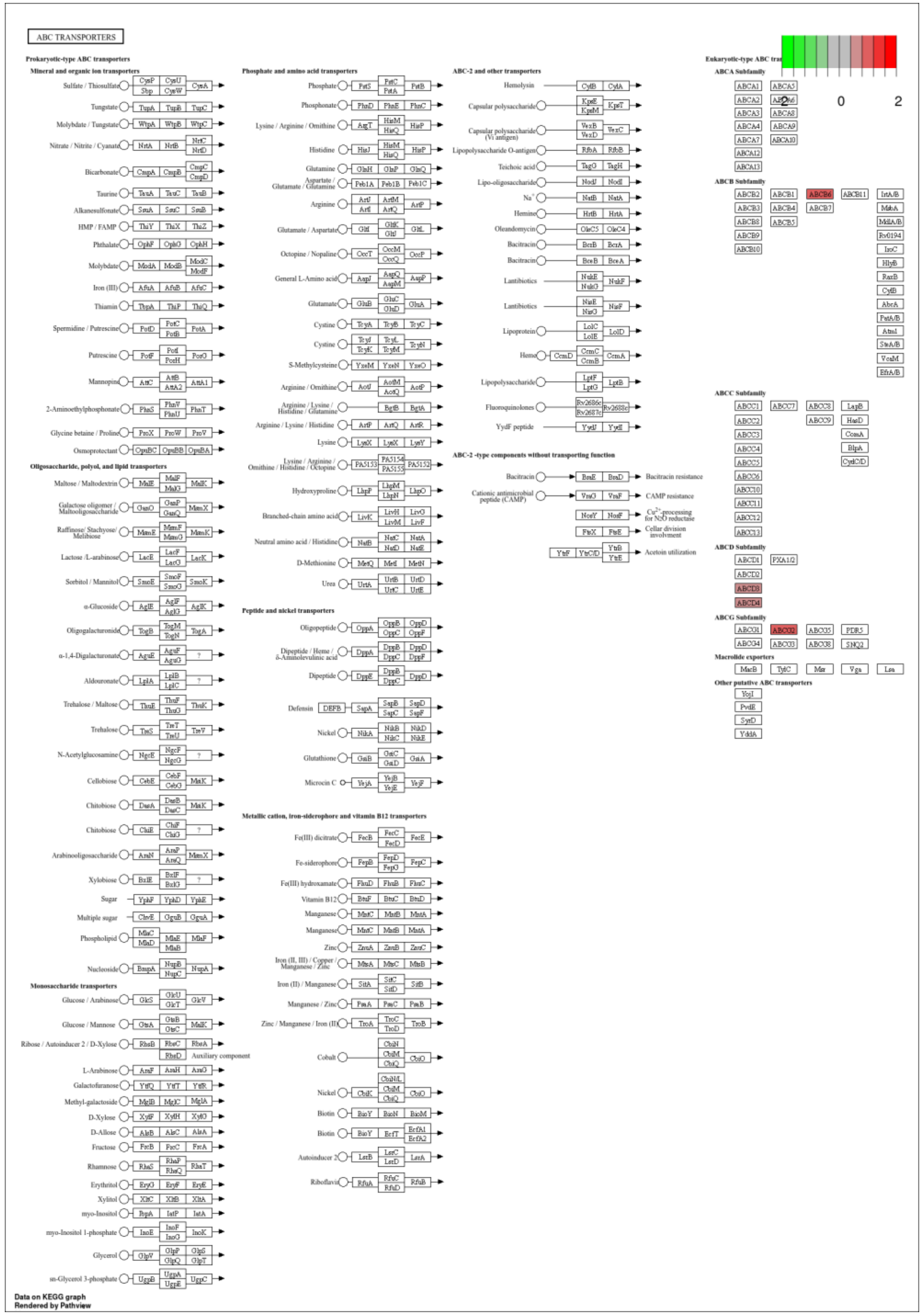

Figure S14. ABC transporters pathway in SYN group in F1 (Cecal tonsils).

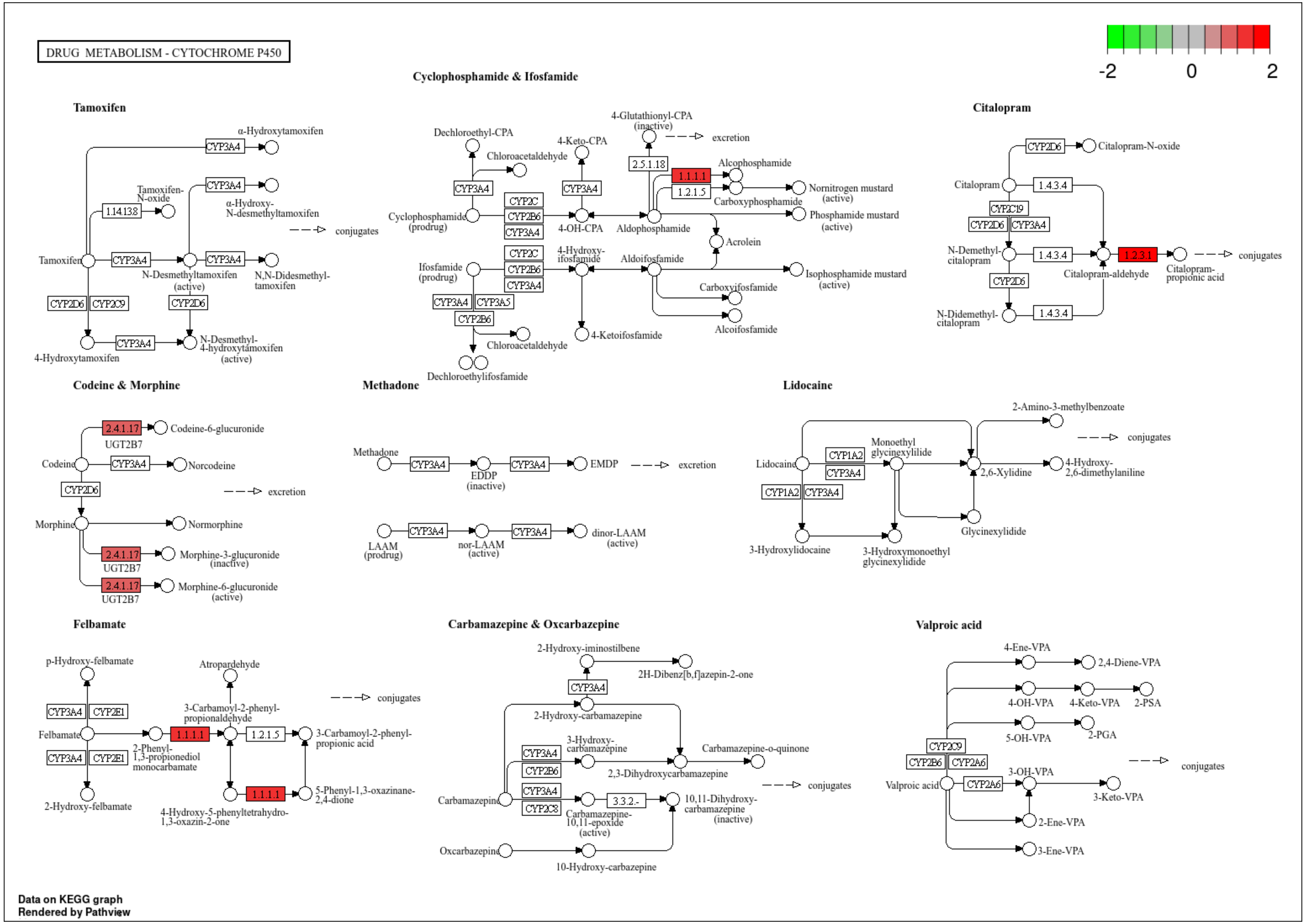

Figure S15. Drug metabolism – cytochrome P450 pathway in SYN group in F1 (Cecal tonsils).

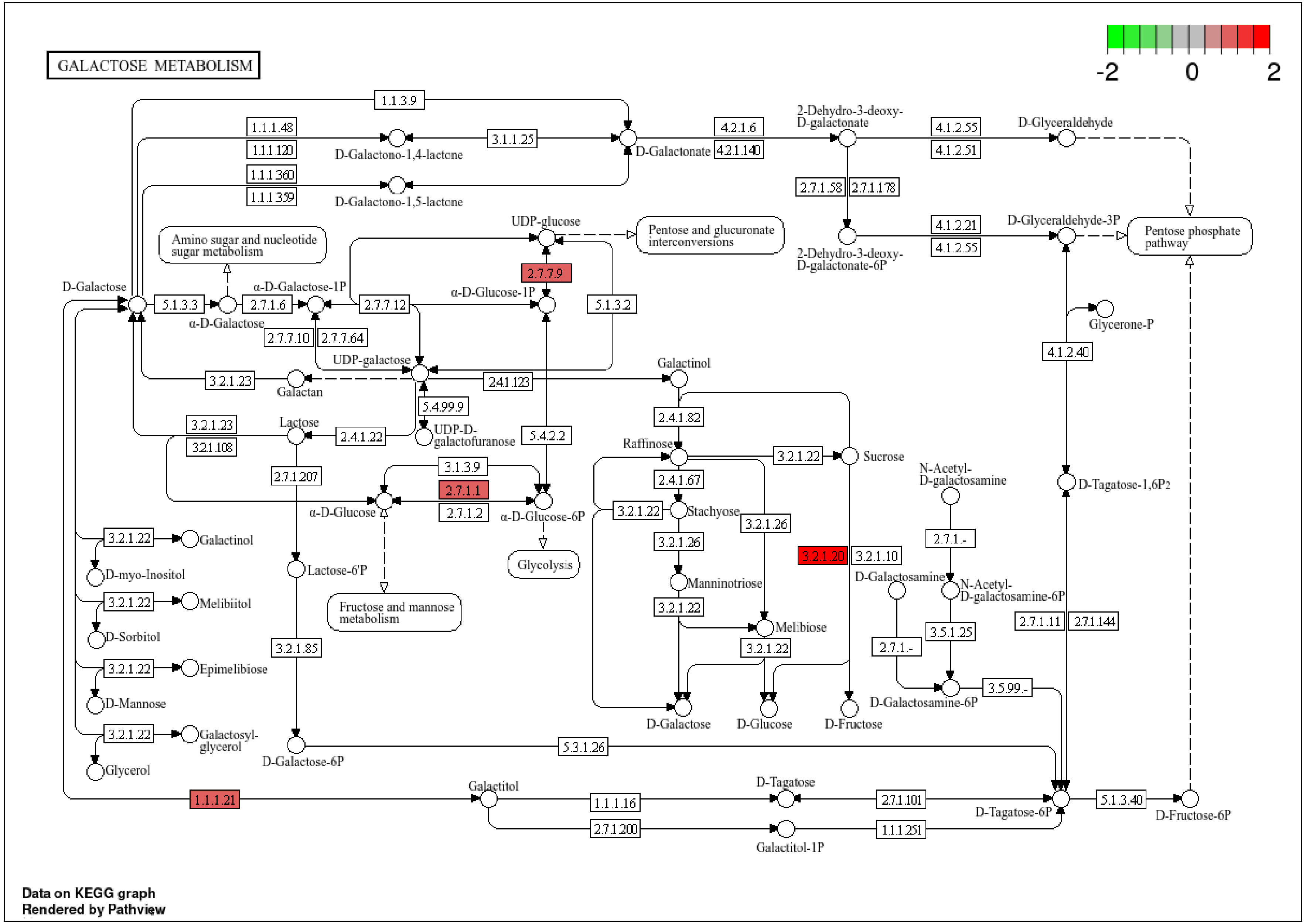

Figure S16. Galactose metabolism pathway in SYN group in F1 (Cecal tonsils).

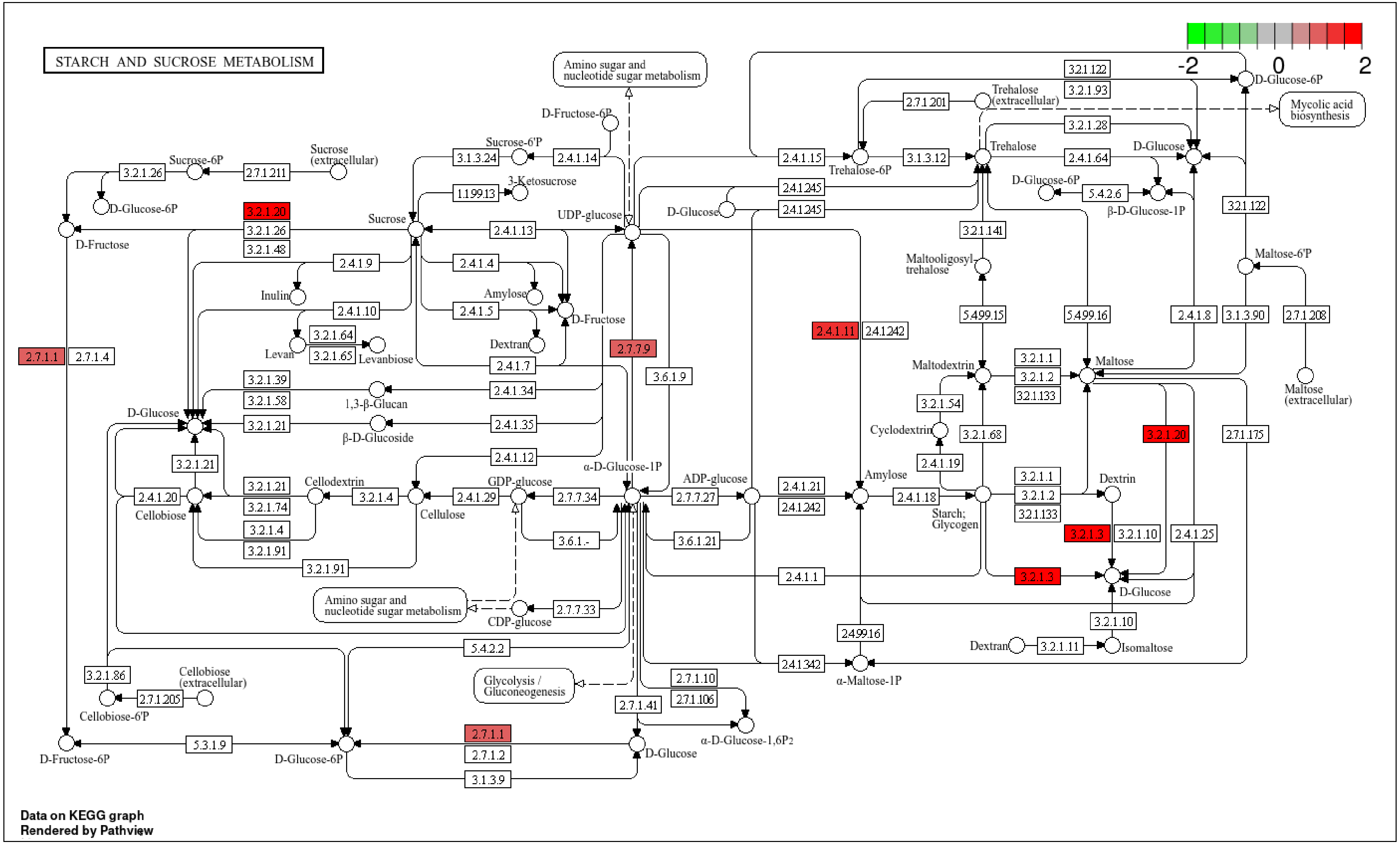

Figure S17. Starch and sucrose metabolism pathway in SYN group in F1 (Cecal tonsils).

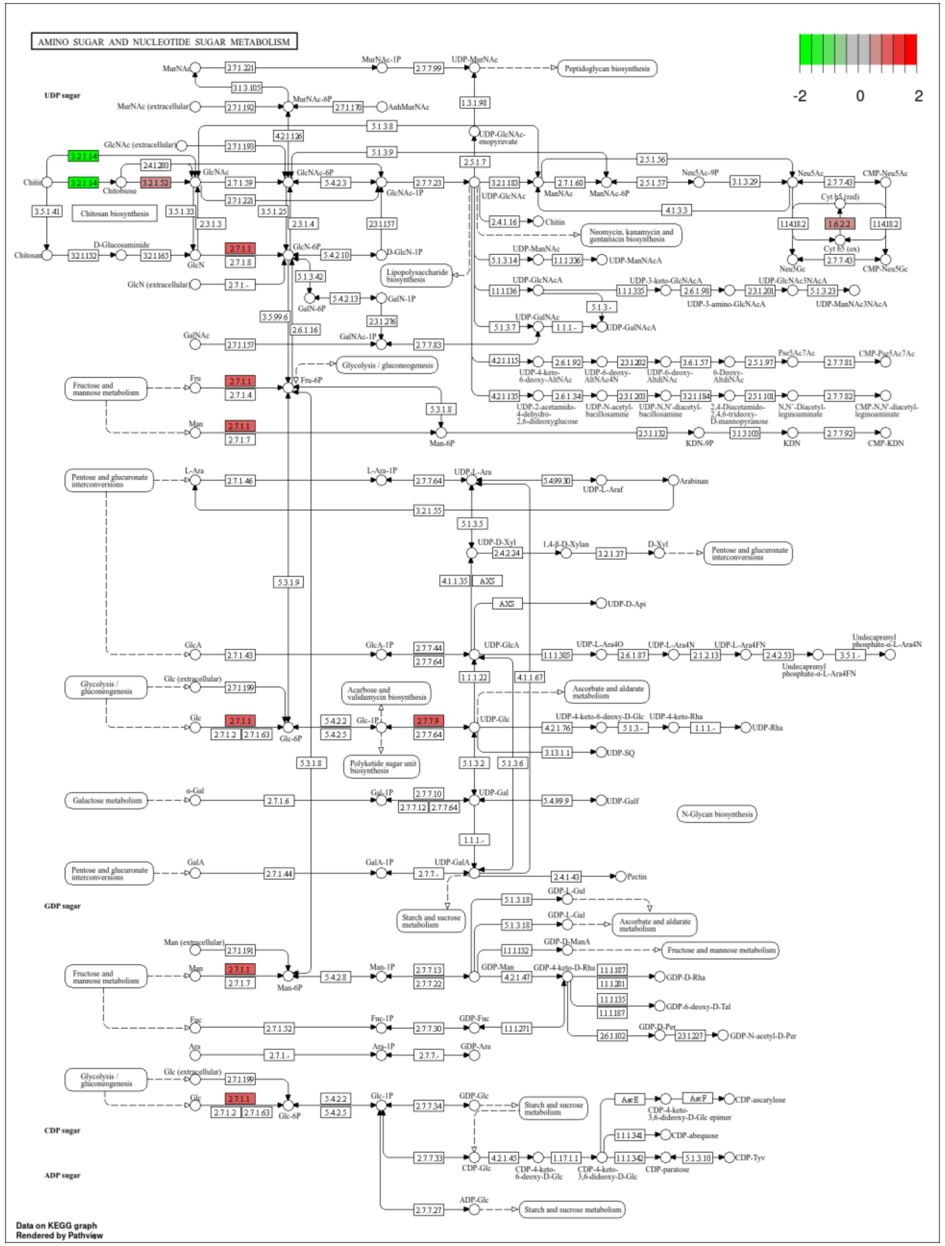

Figure S18. Amino sugar and nucleotide sugar metabolism pathway in SYN group in F1 (Cecal tonsils).

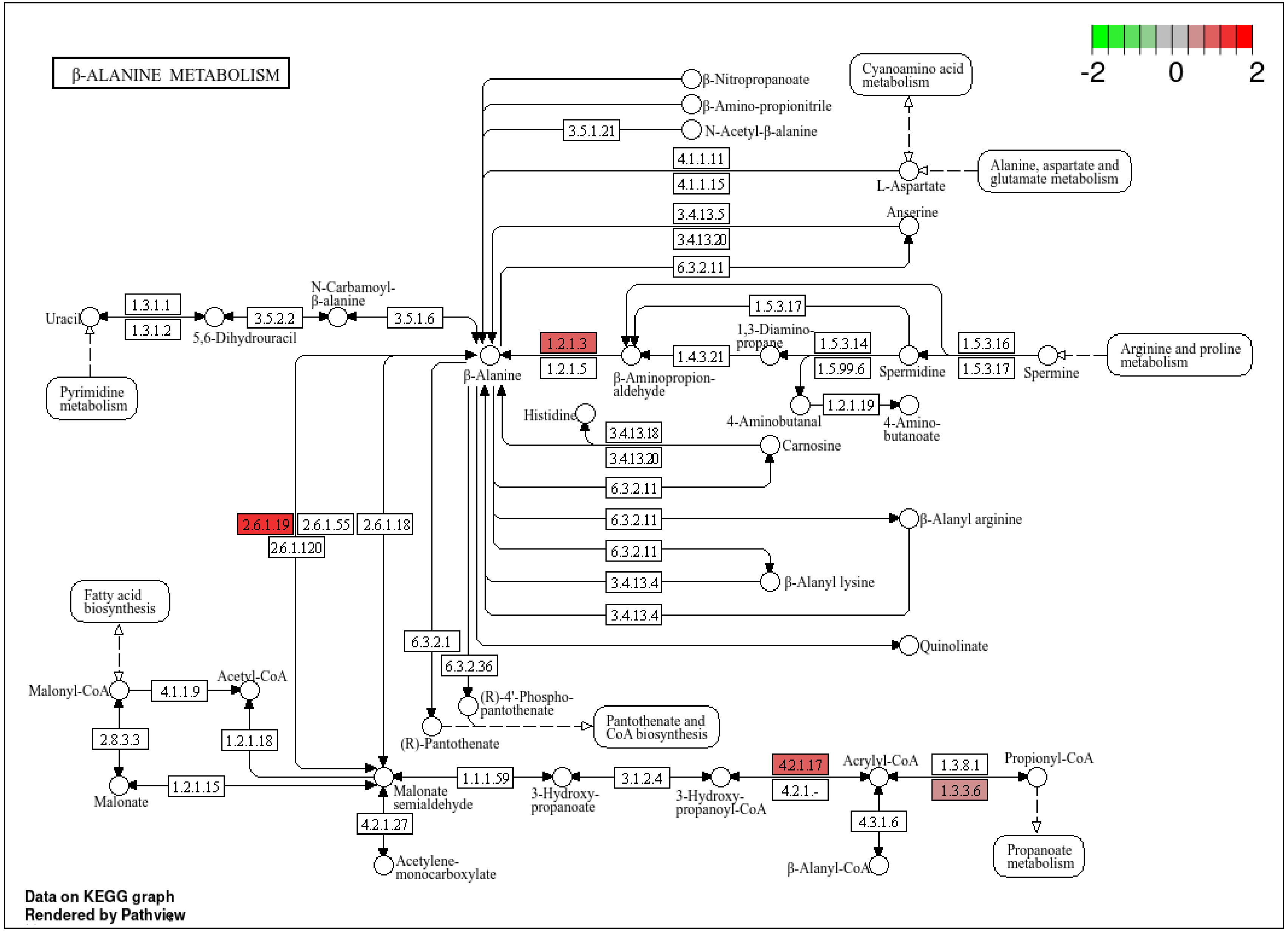

Figure S19. Betta- alanine metabolism pathway in SYN group in F1 (Cecal tonsils).

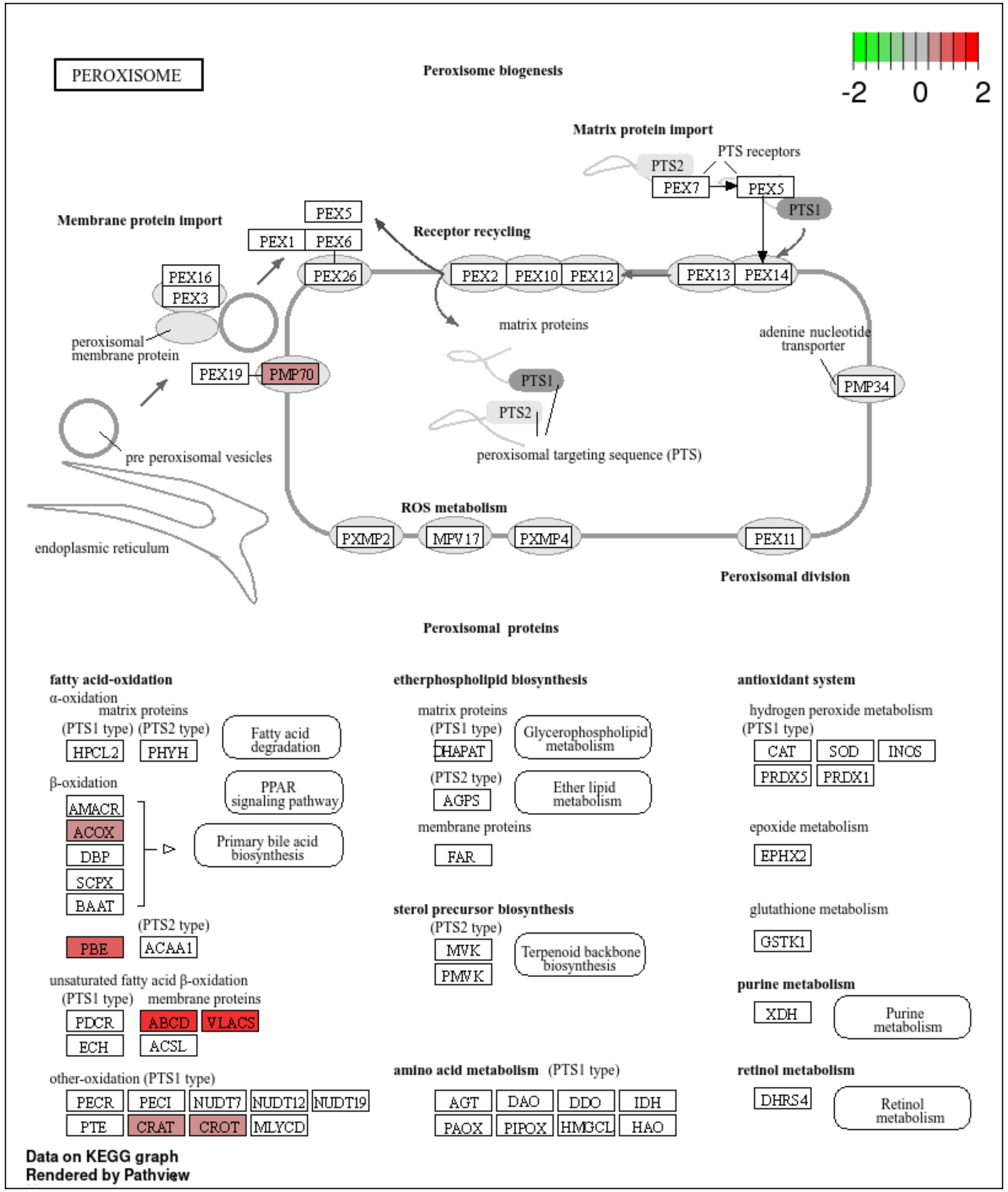

Figure S20. Peroxisome pathway in SYN group in F1 (Cecal tonsils).

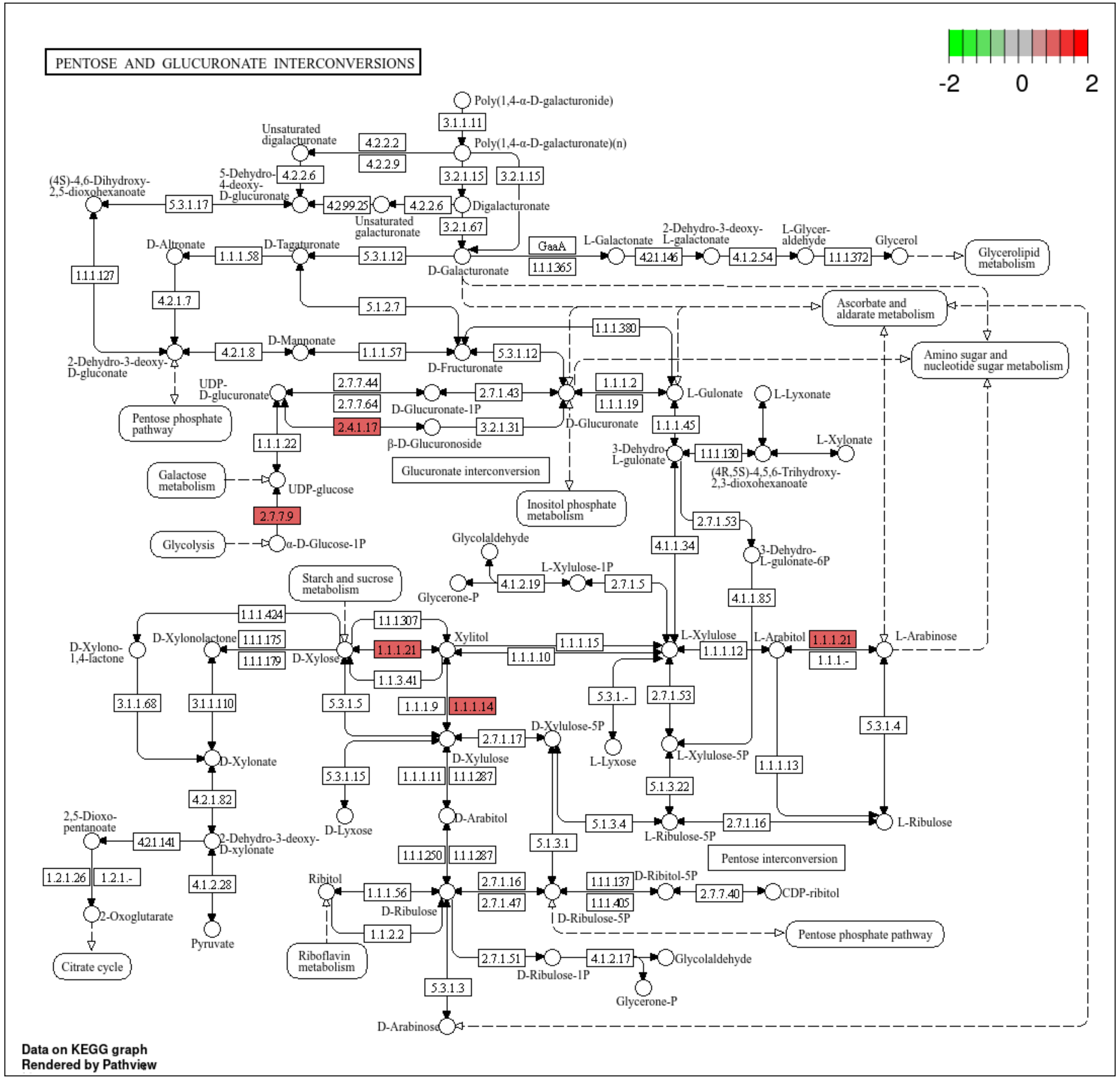

Figure S21. Pentose and glucuronate interconversions pathway in SYN group in F1 (Cecal tonsils).

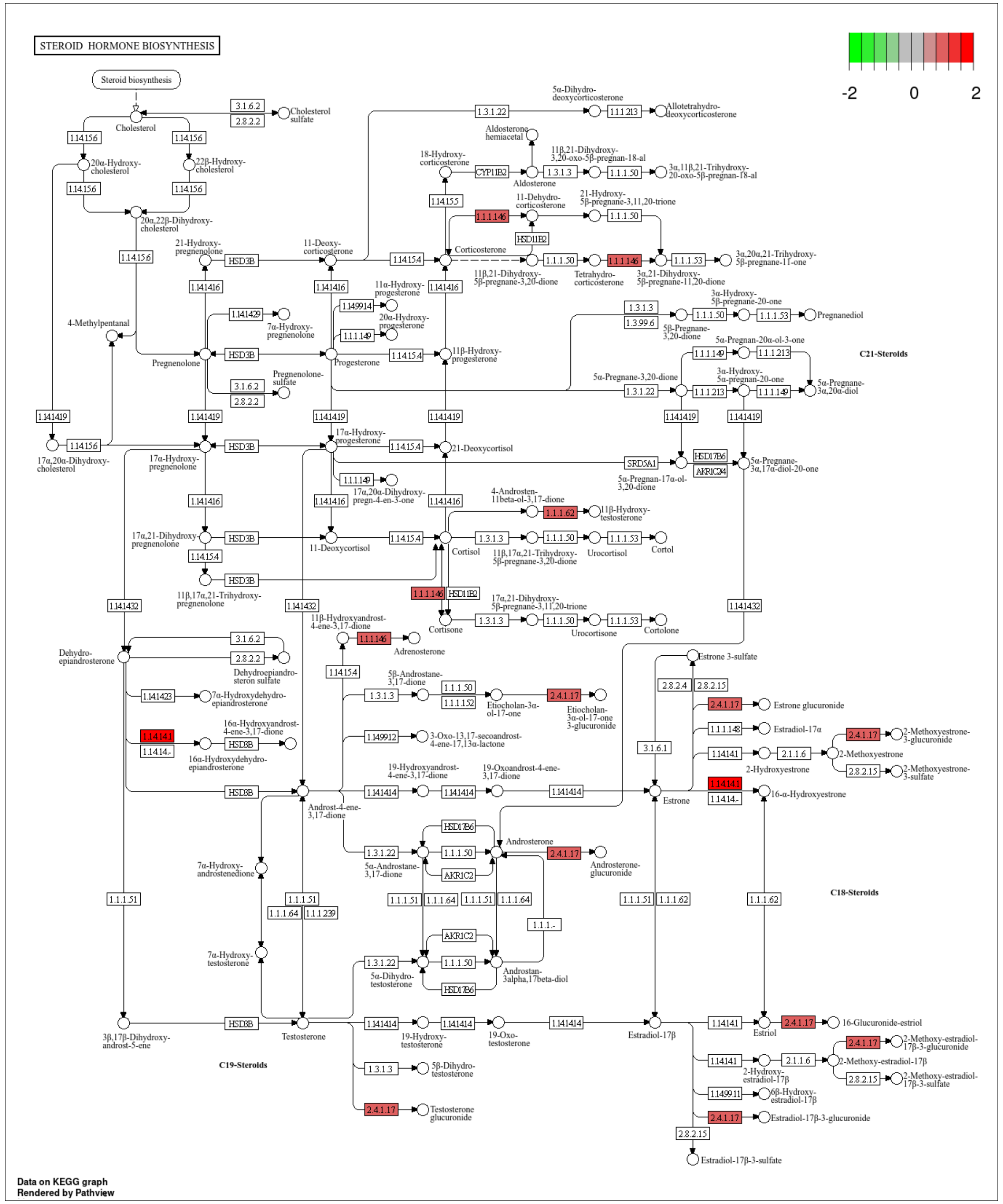

Figure S22. Steroid hormone biosynthesis pathway in SYN group in F1 (Cecal tonsils).

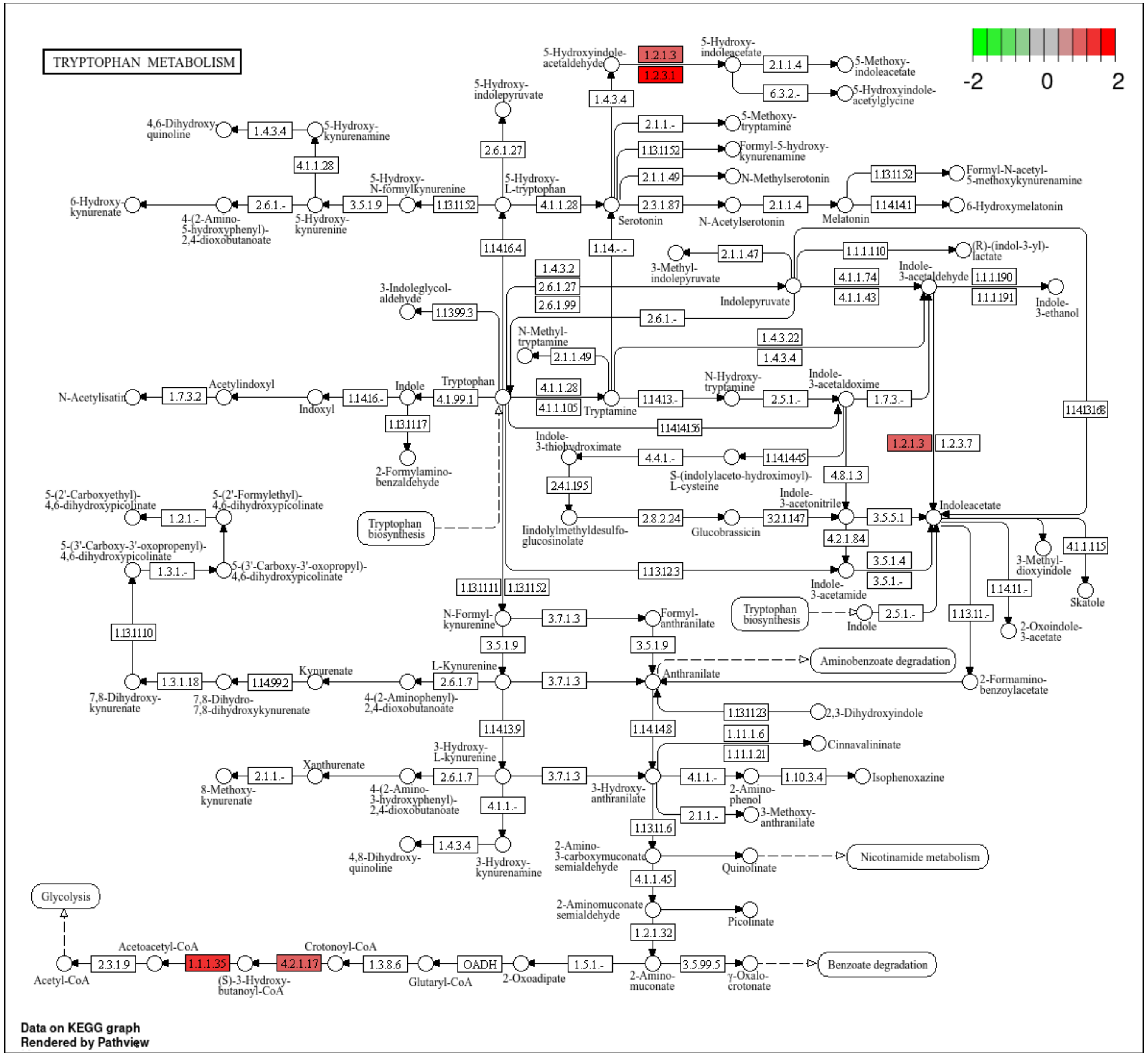

Figure S23. Tryptophan metabolism pathway in SYN group in F1 (Cecal tonsils).

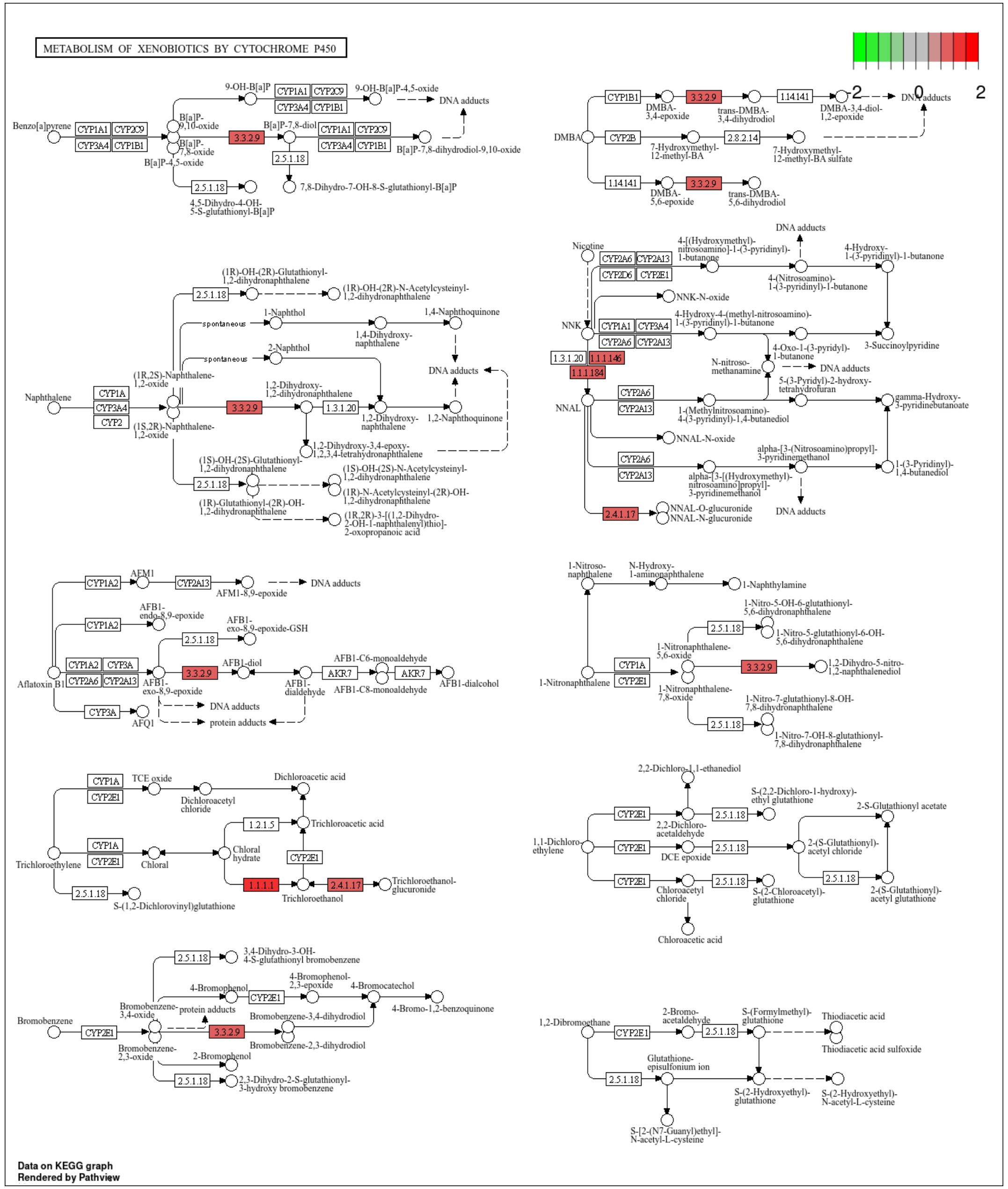

Figure S24. Metabolism of xenobiotics by cytochrome P450 pathway in SYN group in F1 (Cecal tonsils).

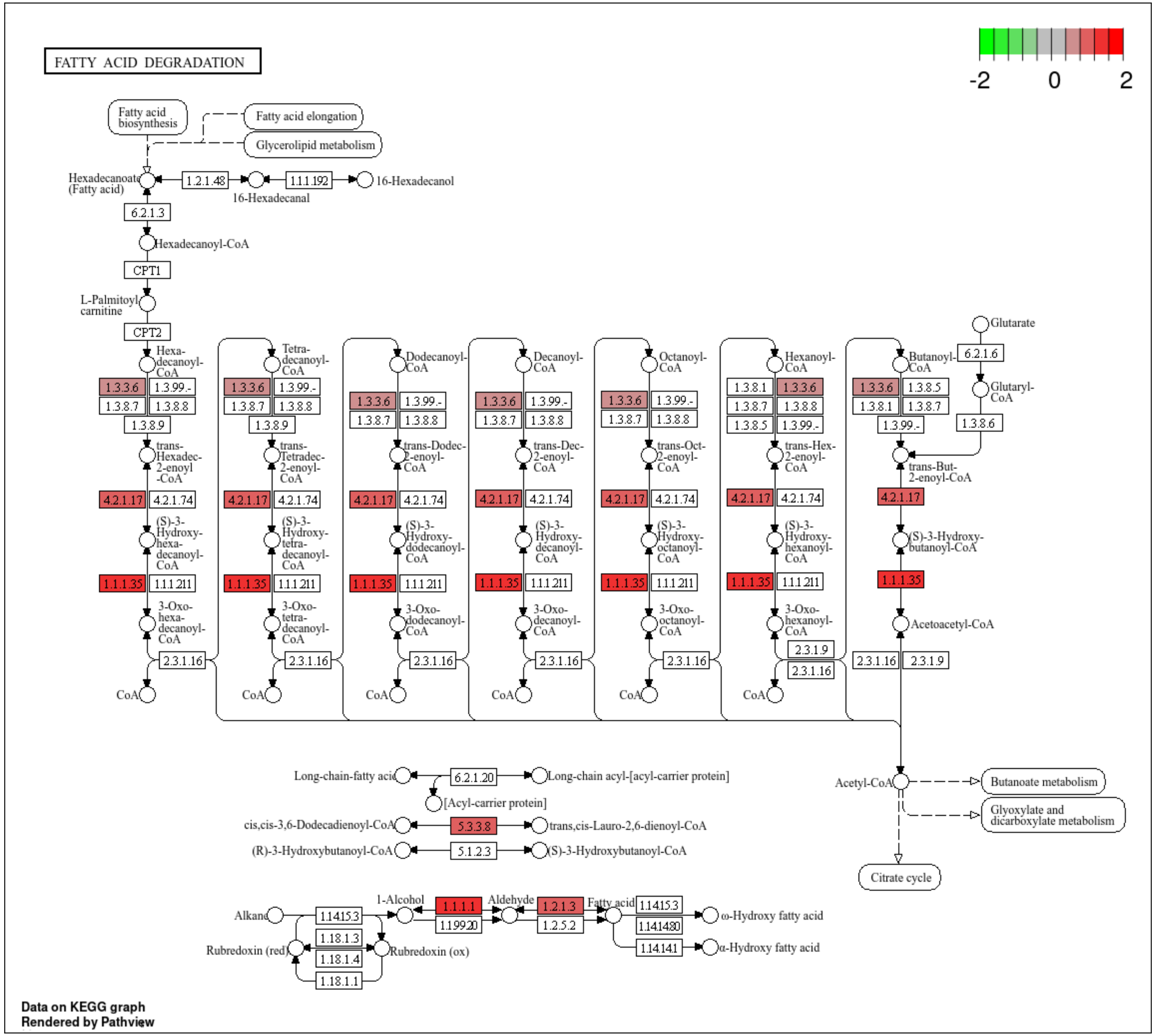

Figure S25. Fatty acid degradation pathway in SYN group in F1 (Cecal tonsils).

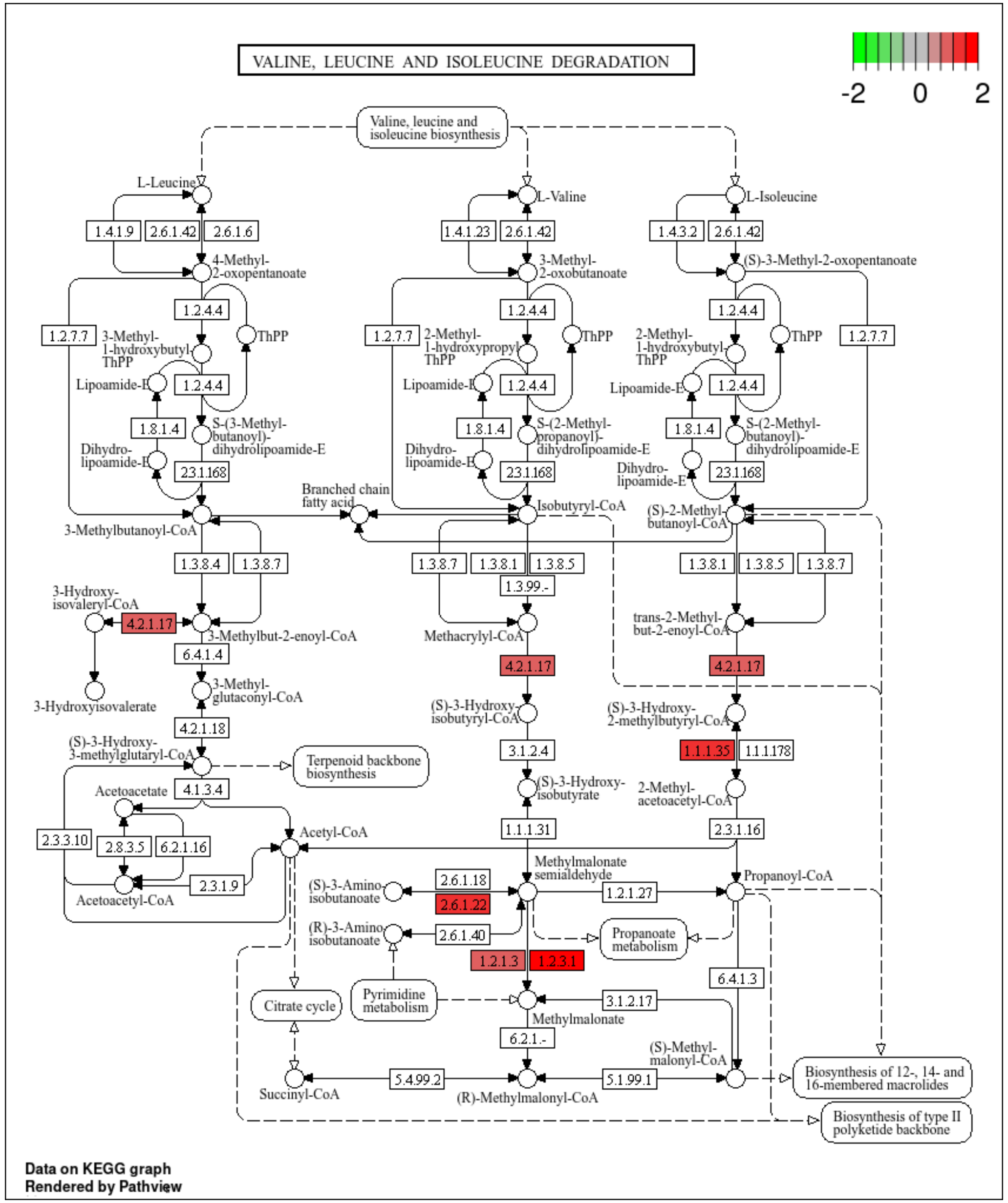

Figure S26. Valine, leucine and isoleucine degradation pathway in SYN group in F1 (Cecal tonsils).

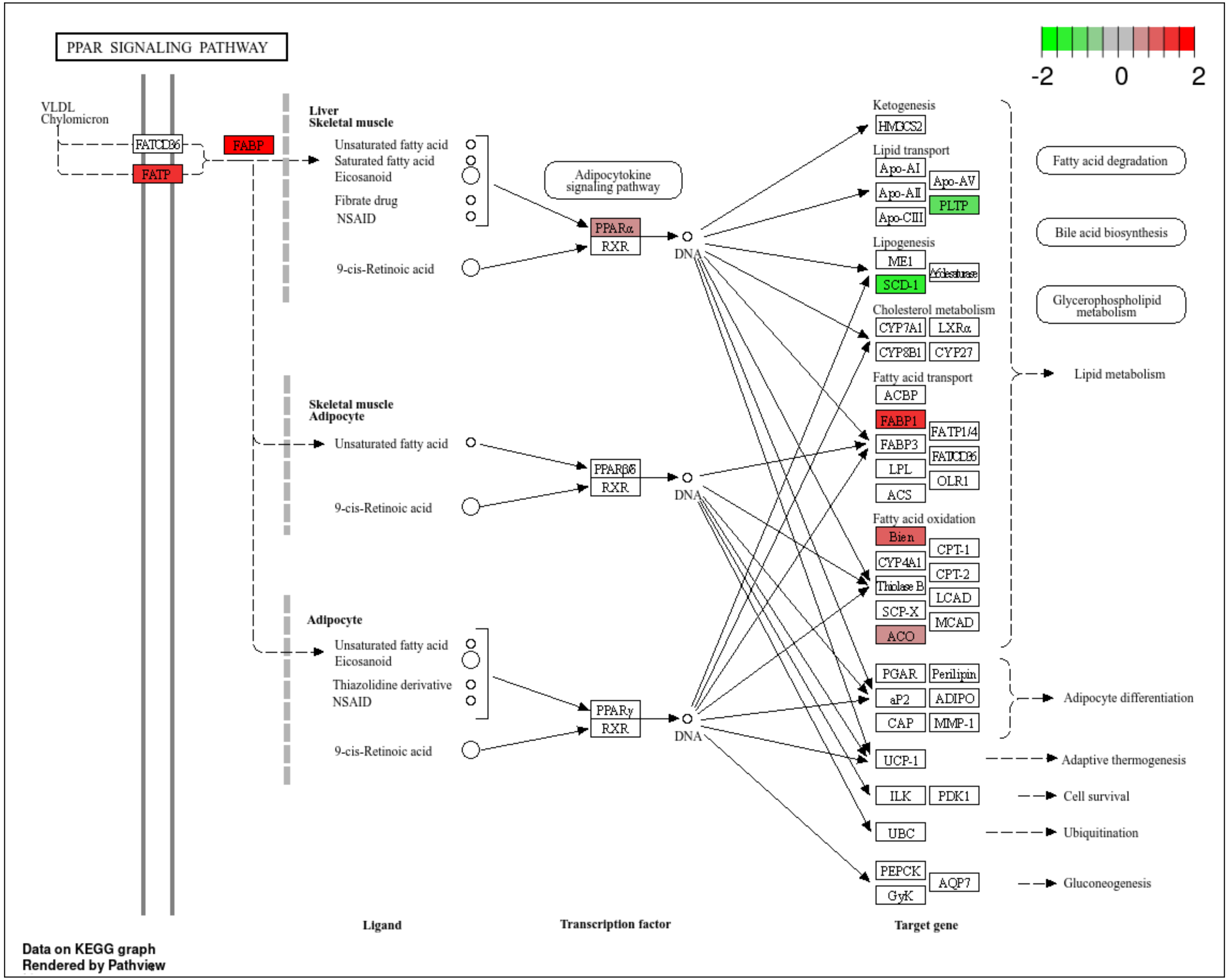

Figure S27. PPAR Signaling pathway in SYN group in F1 (Cecal tonsils).

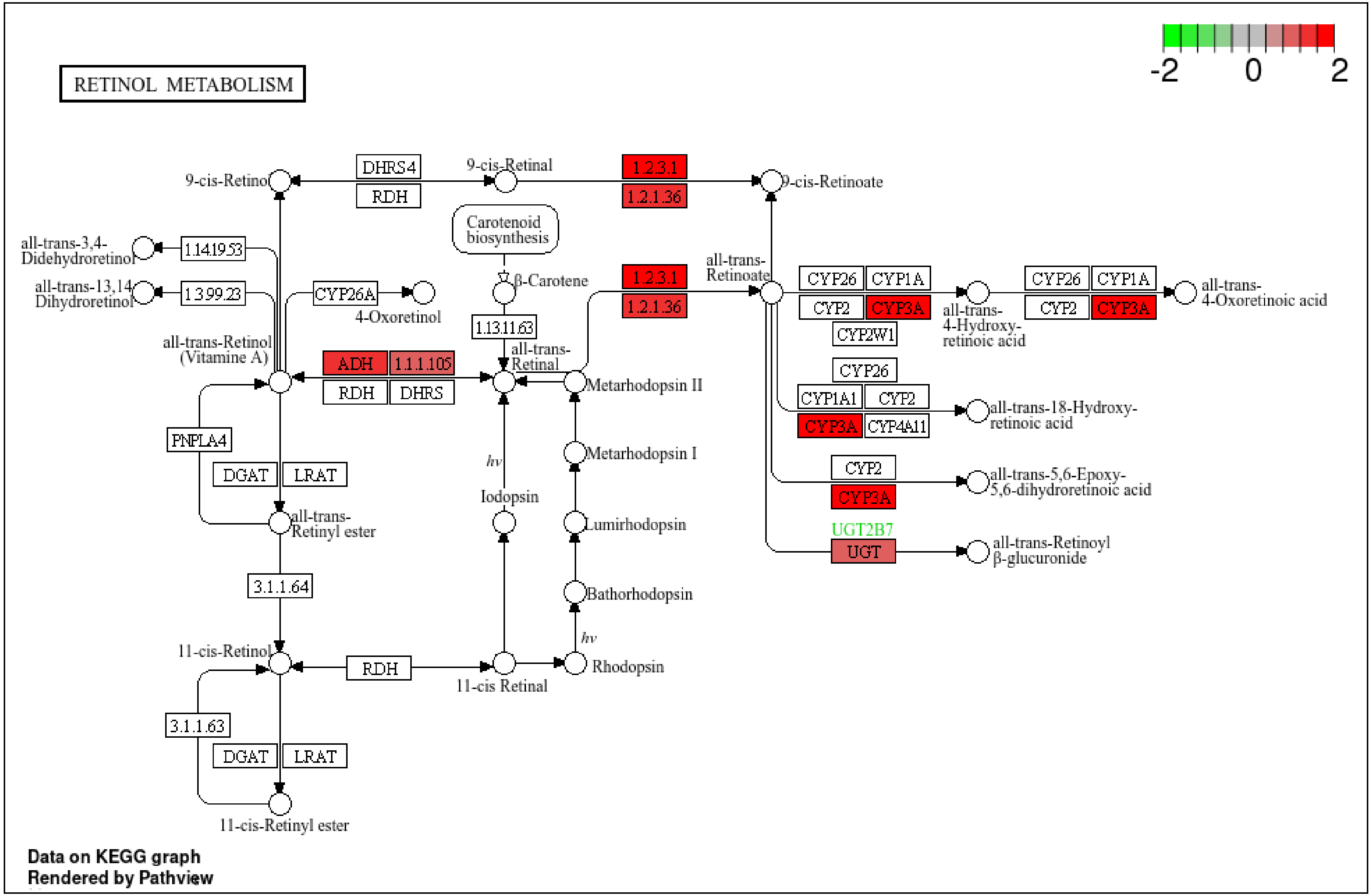

Figure S28. Retinol metabolism pathway in SYN group in F1 (Cecal tonsils).

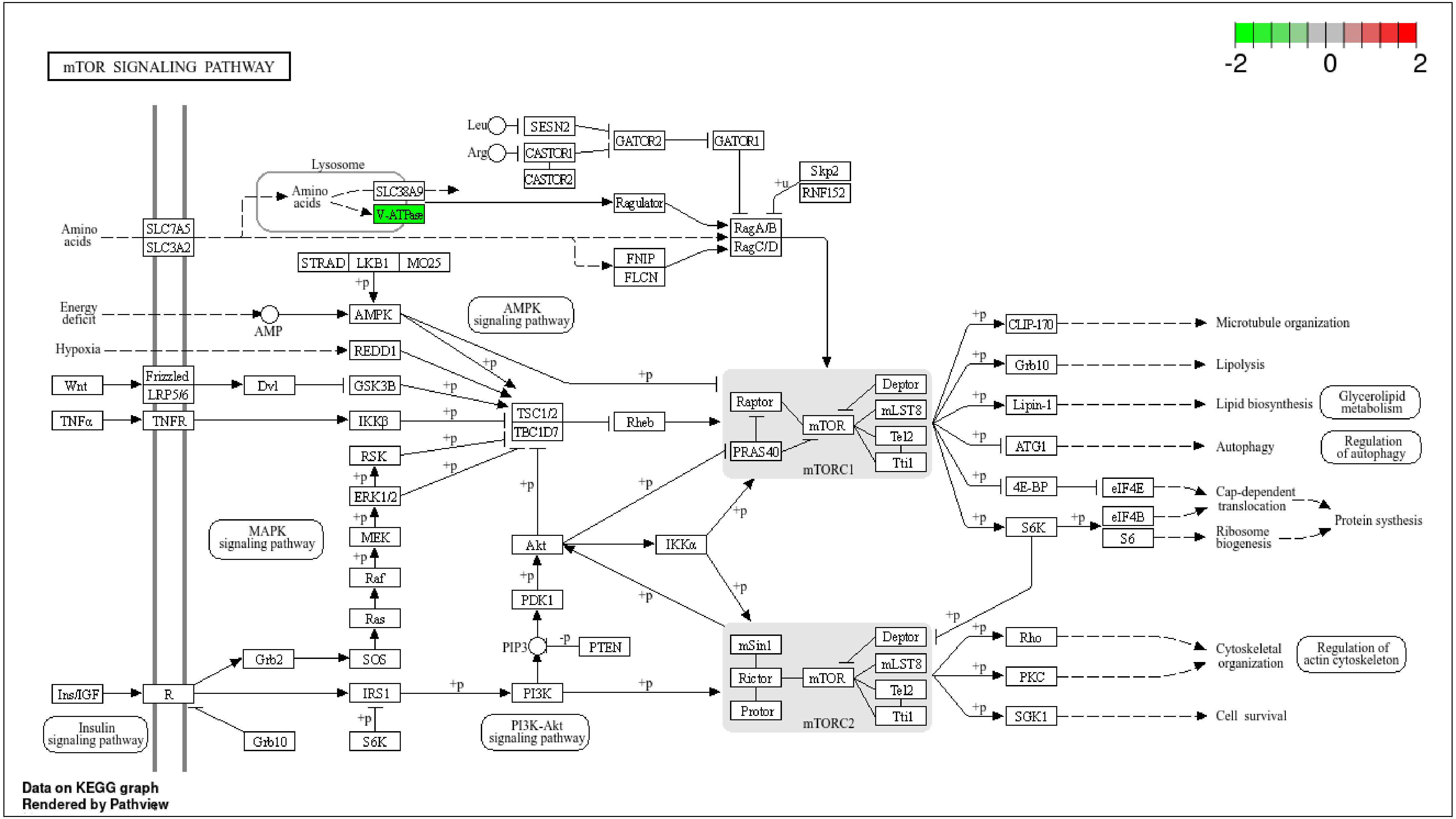

Figure S29. mTOR signaling pathway in SYNCH group in F1 (Cecal tonsils).

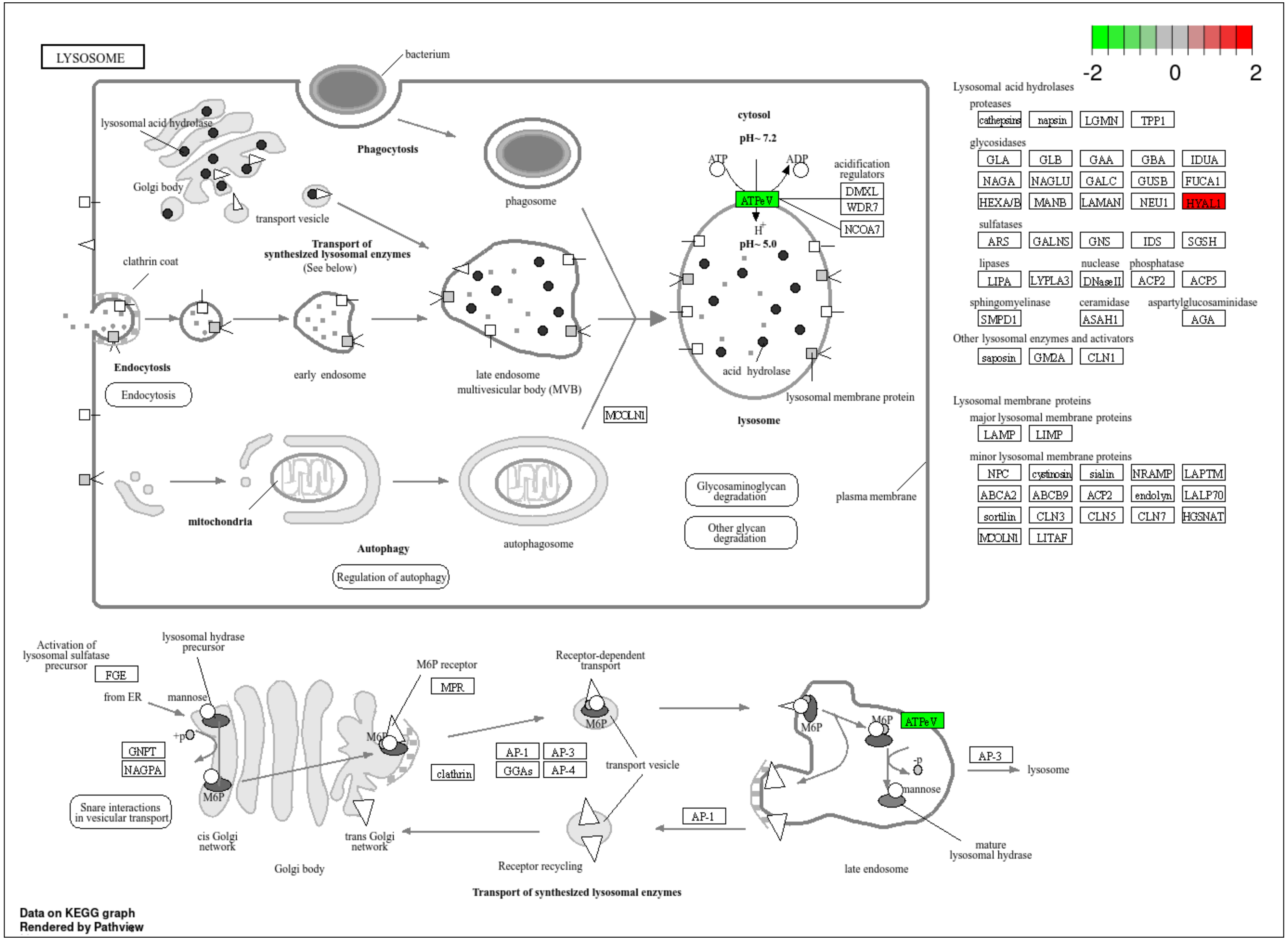

Figure S30. Lysosome pathway in SYNCH group in F1 (Cecal tonsils).

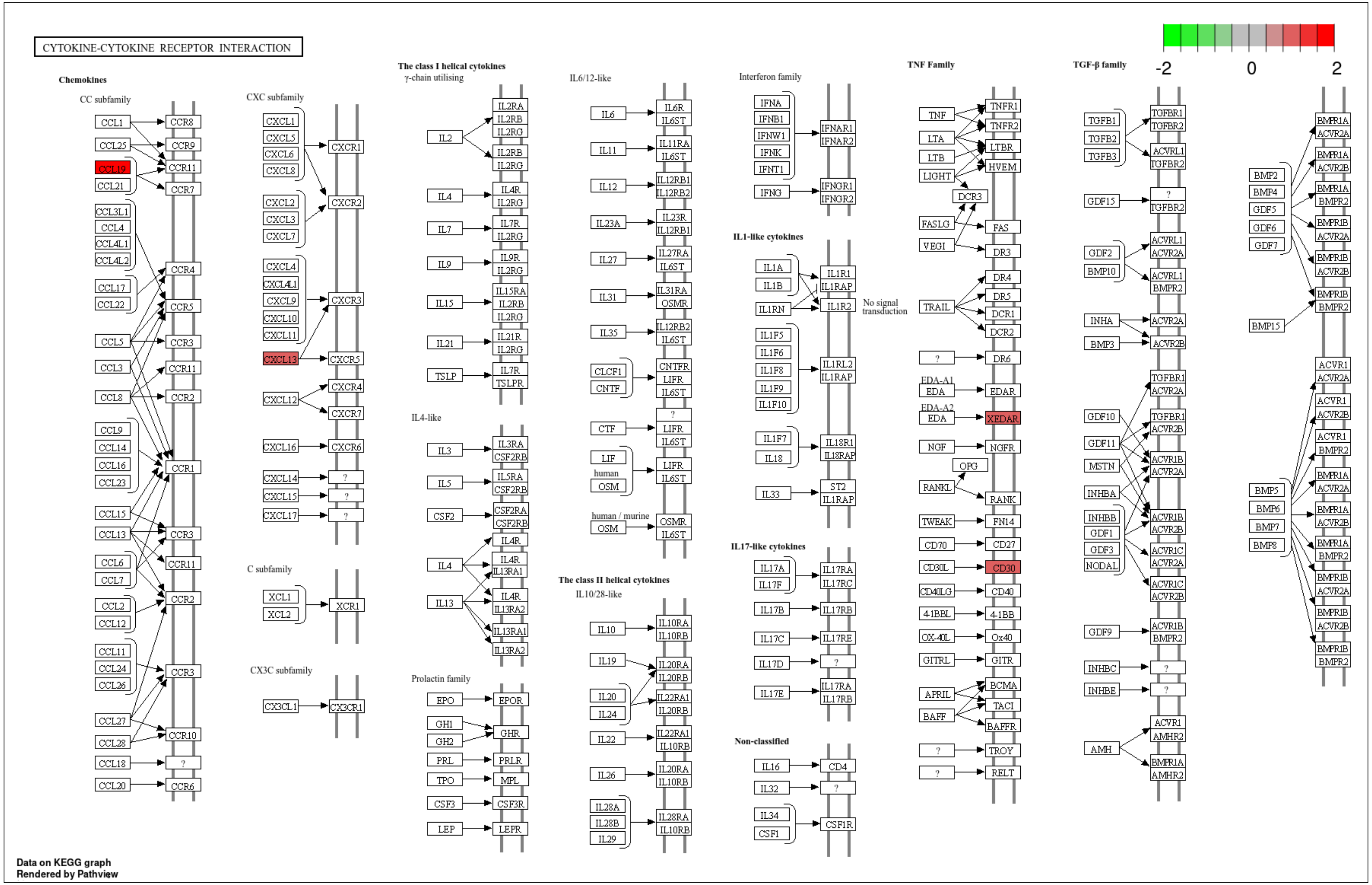

Figure S31. Cytokine- cytokine receptor interaction pathway in SYNCH group in F1 (Cecal tonsils).

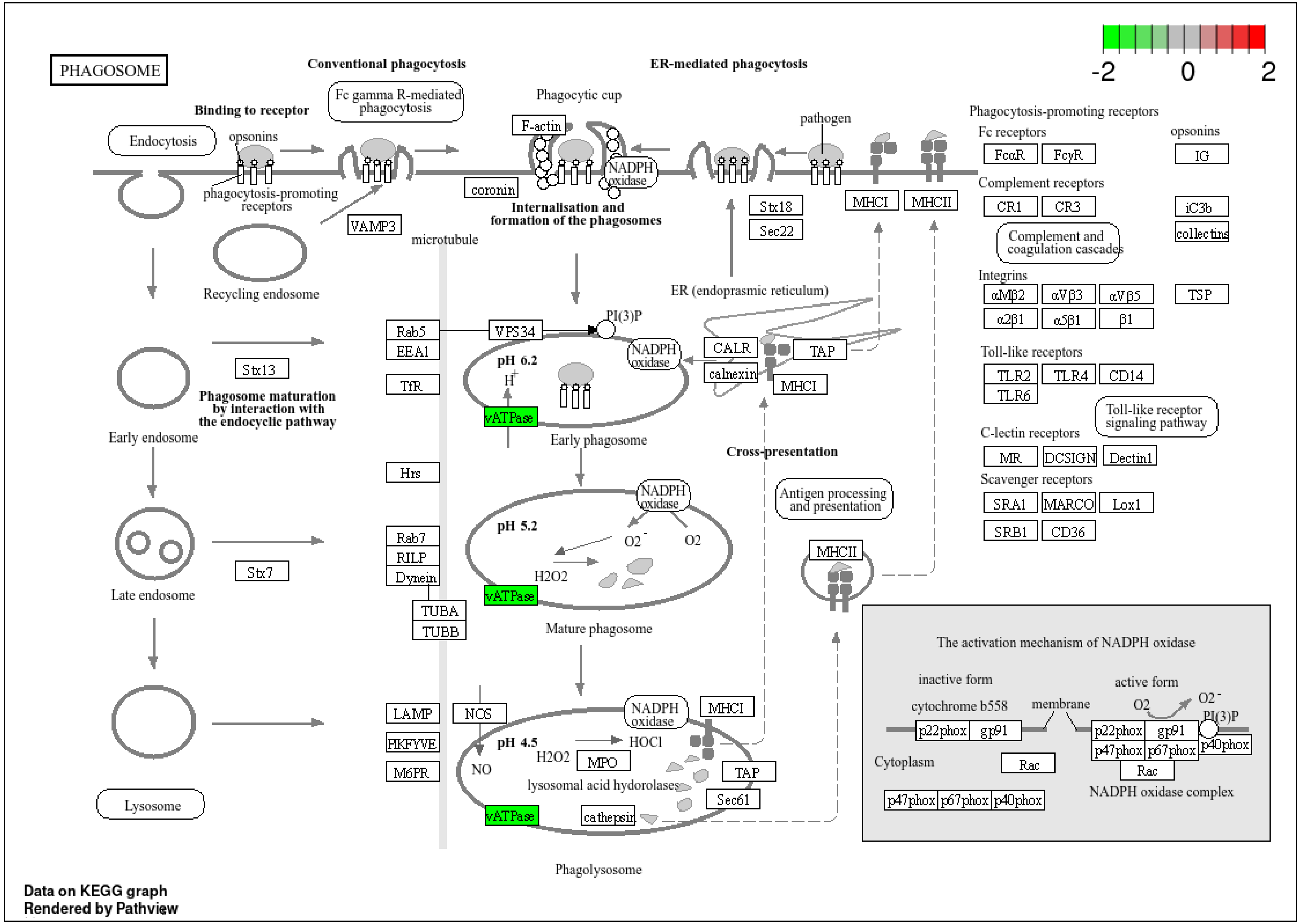

Figure S32. Phagosome pathway in SYNCH group in F1 (Cecal tonsils).

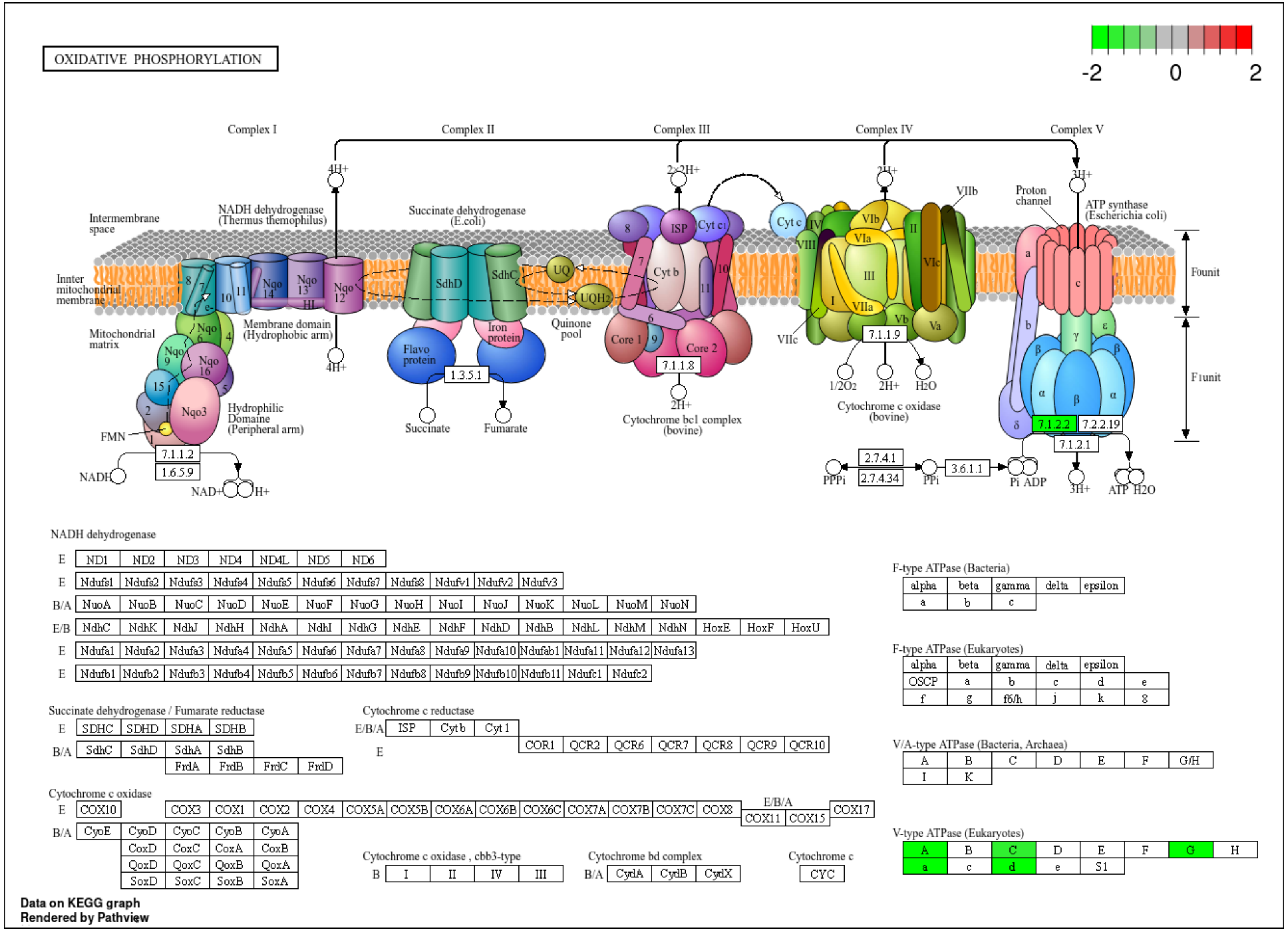

Figure S33. Oxidative phosphorylation pathway in SYNCH group in F1 (Cecal tonsils).

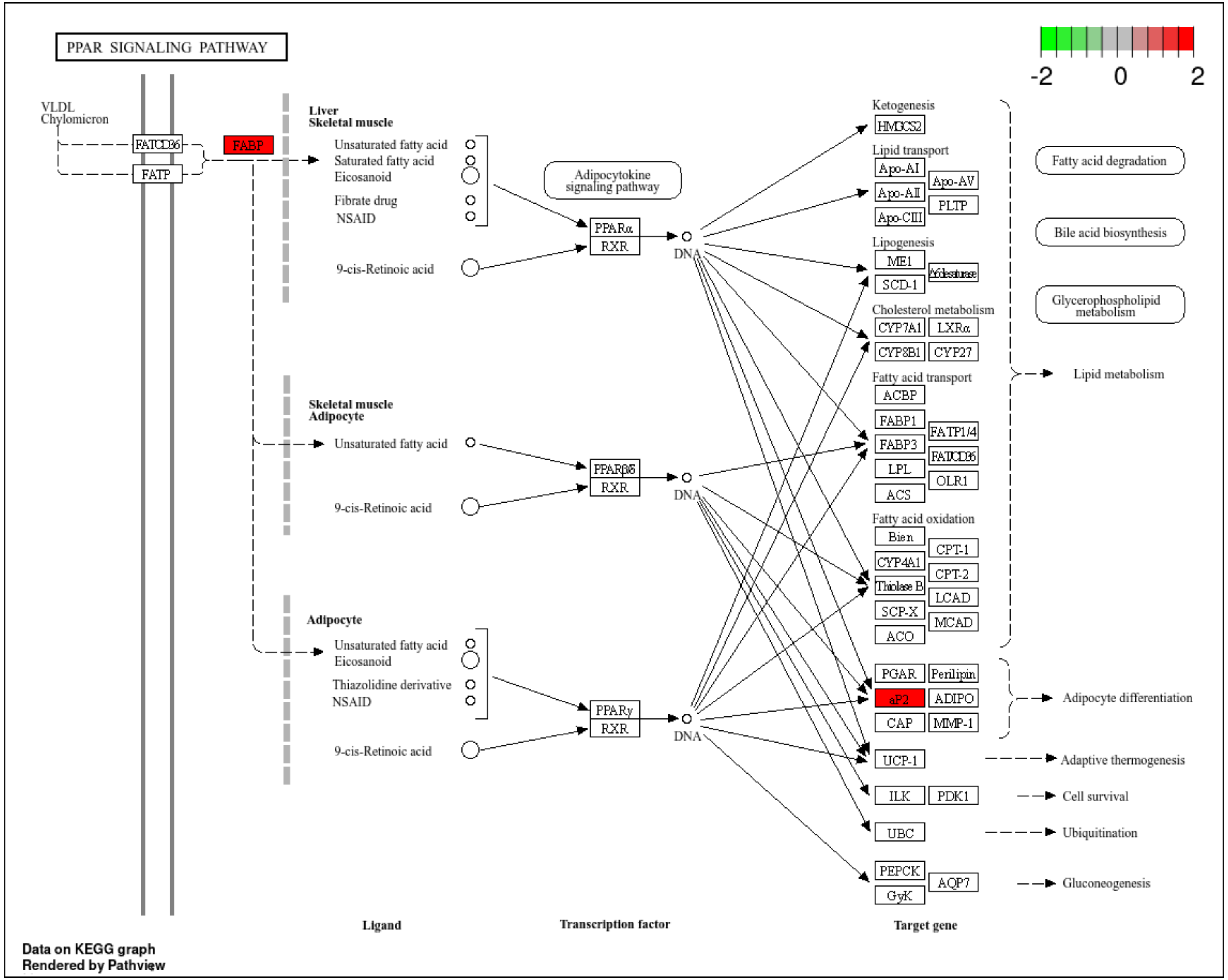

Figure S34. PPAR signaling pathway in SYNs group in F2 (Cecal tonsils).

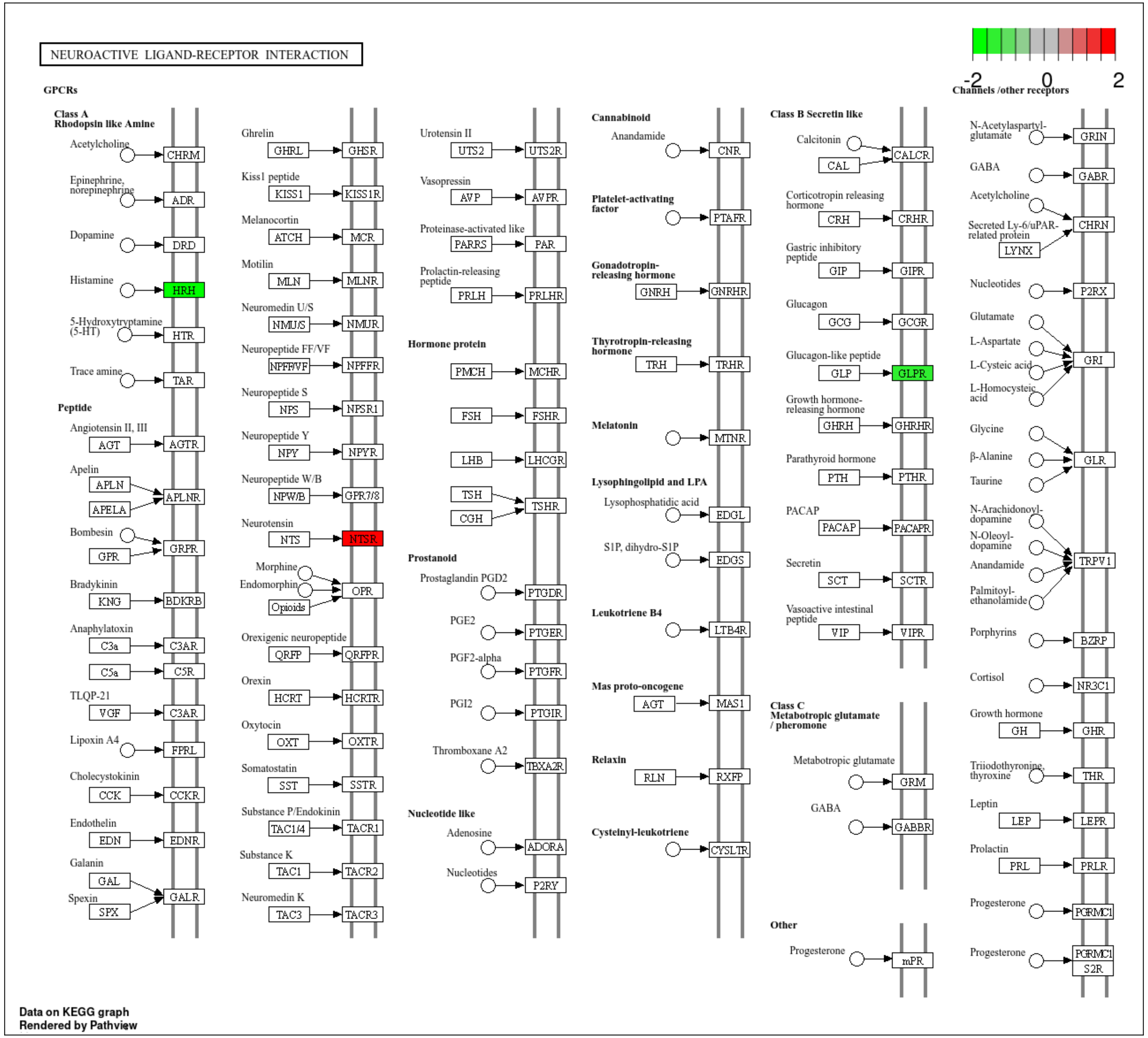

Figure S35. Neuroactive ligand – receptor interaction pathway in SYNCHr group in F2 (Cecal tonsils).

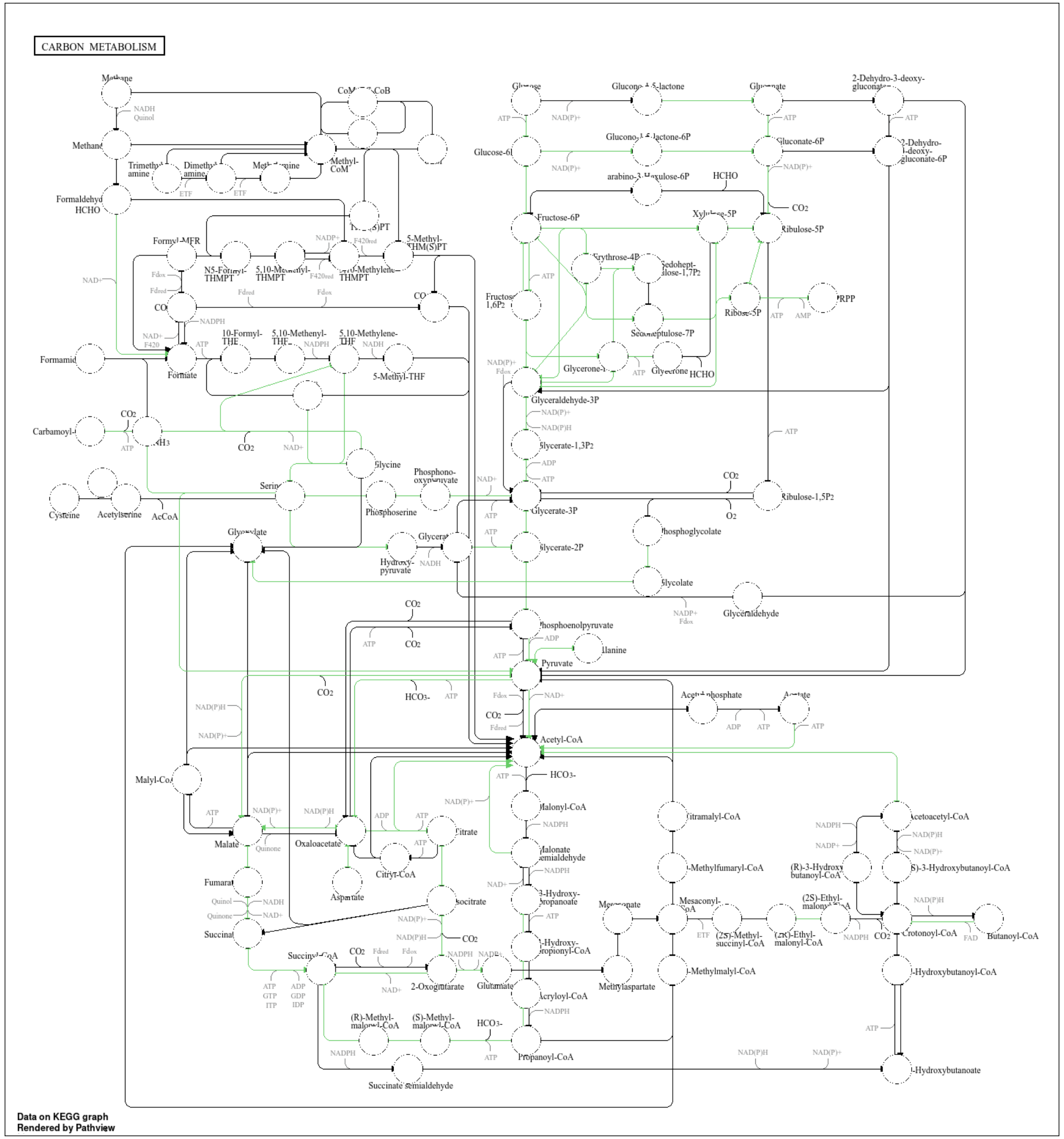

Figure S36. Carbon metabolism pathway in SYNCHr group in F2 (Cecal tonsils).

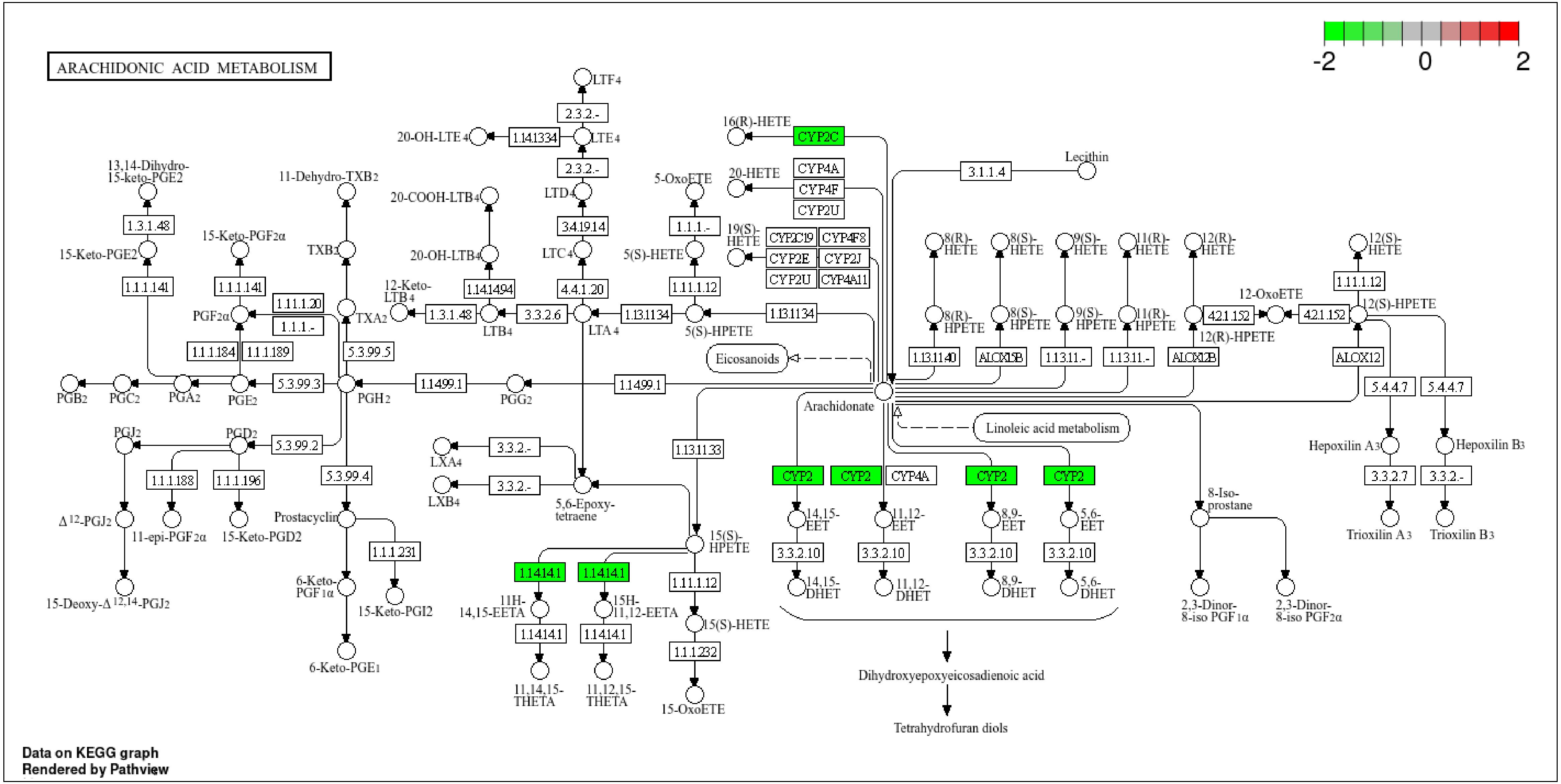

Figure S37. Arachidonic acid metabolism pathway in SYNCHr group in F2 (Cecal tonsils).

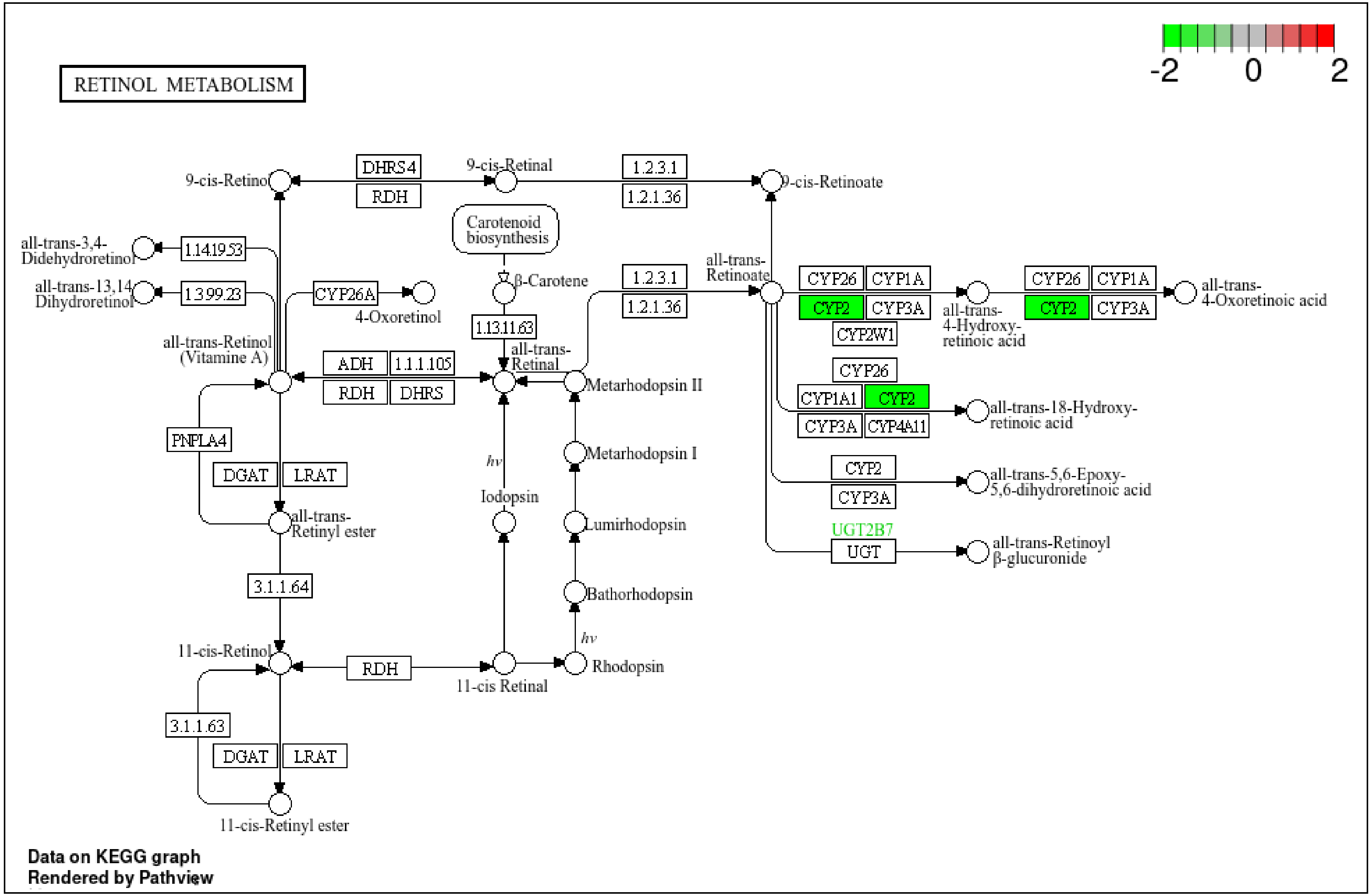

Figure S38. Retinol metabolism pathway in SYNCHr group in F2 (Cecal tonsils).

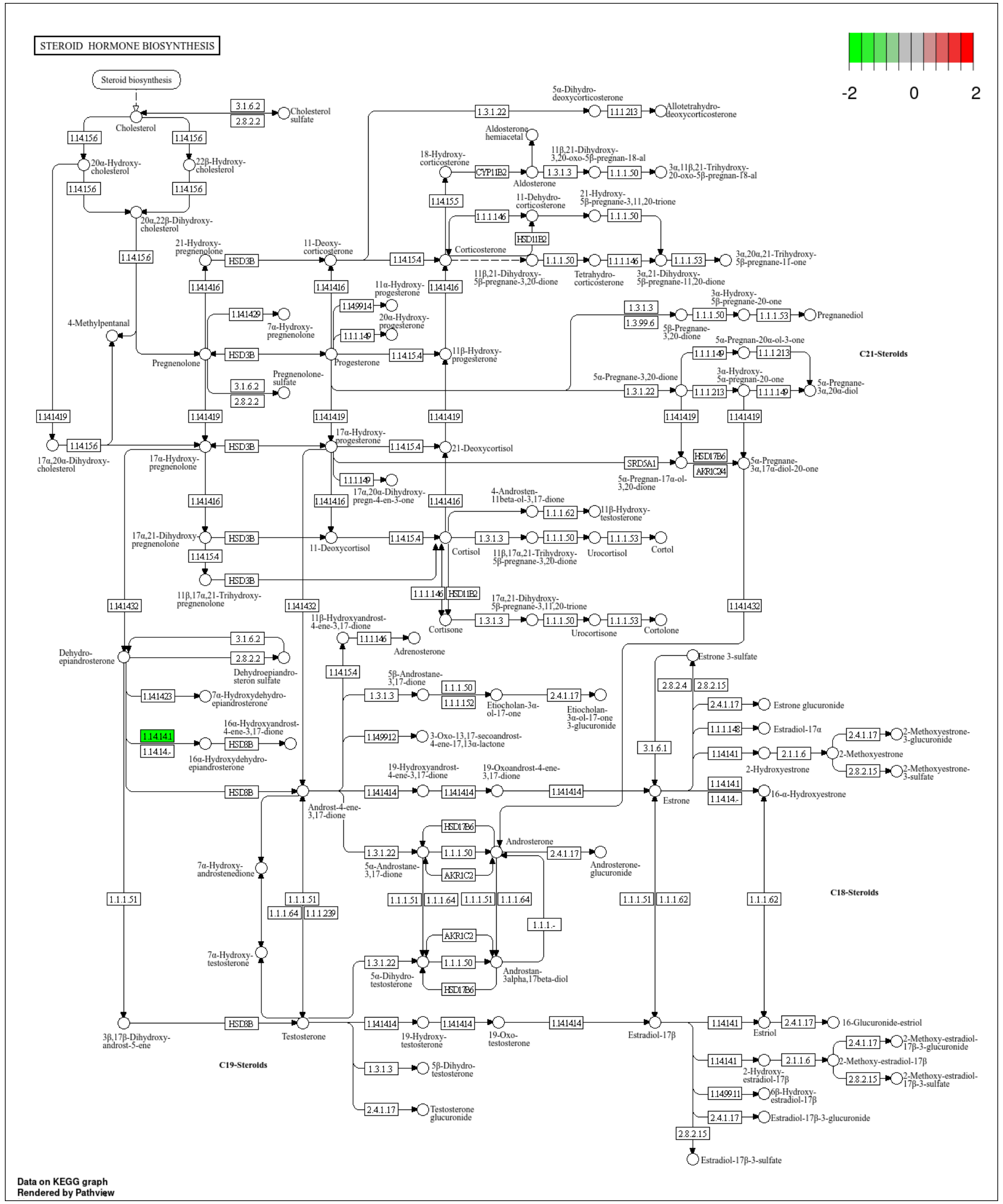

Figure S39. Steroid hormone biosynthesis pathway in SYNCHr group in F2 (Cecal tonsils).

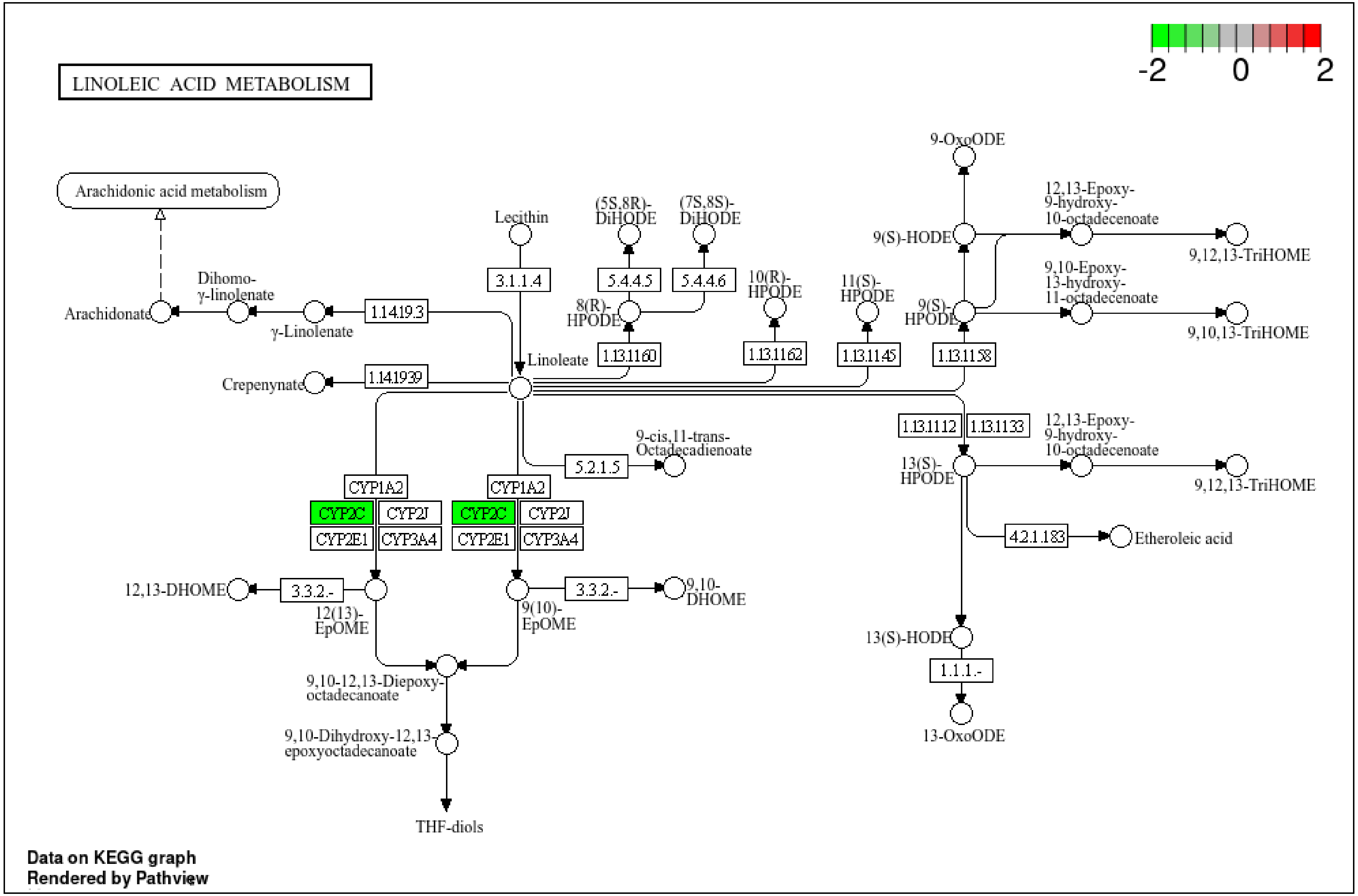

Figure S40. Linoleic acid metabolism pathway in SYNCHr group in F2 (Cecal tonsils).

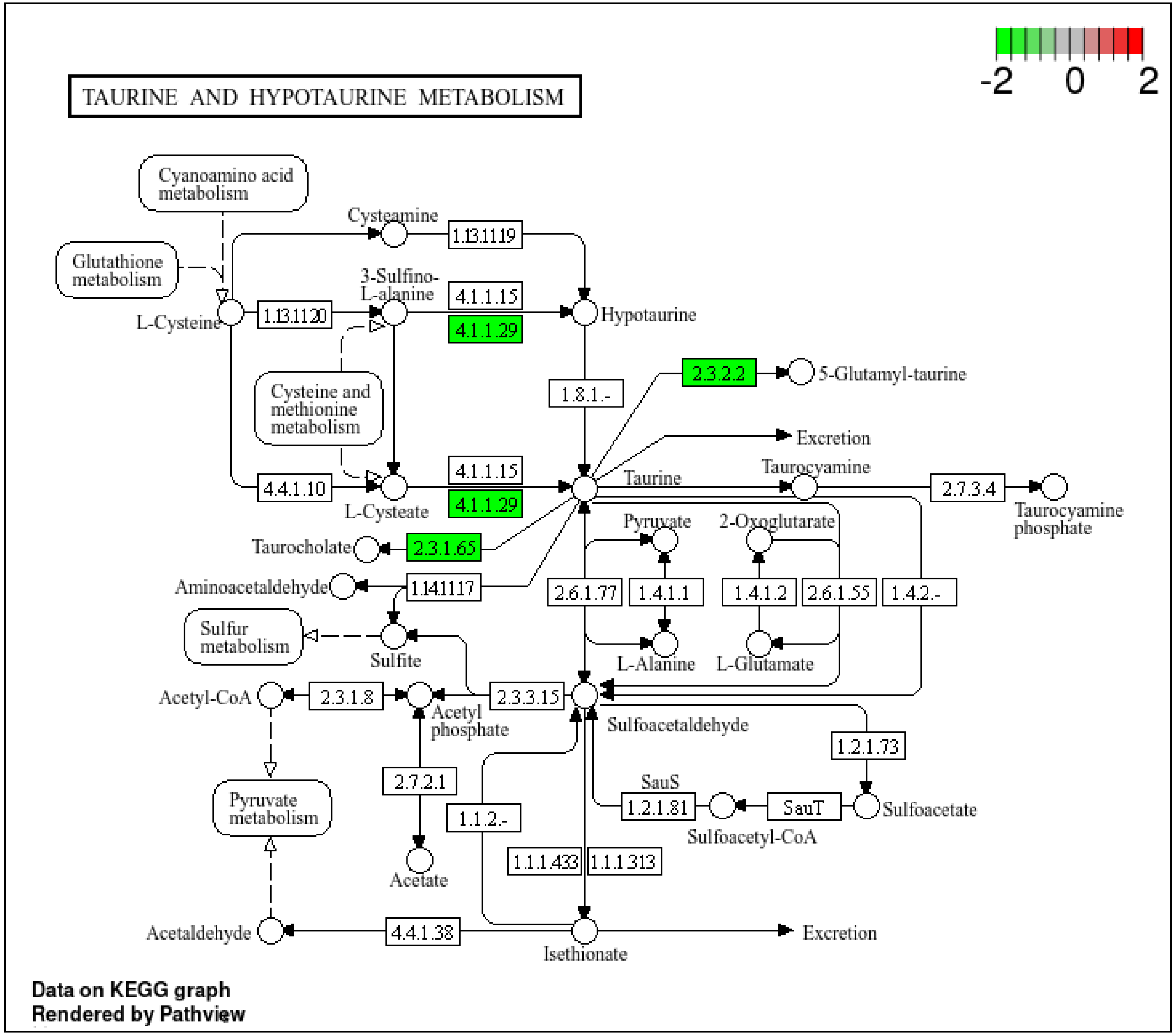

Figure S41. Taurine and hypotaurine metabolism pathway in SYNs group in F3 (Cecal tonsils).

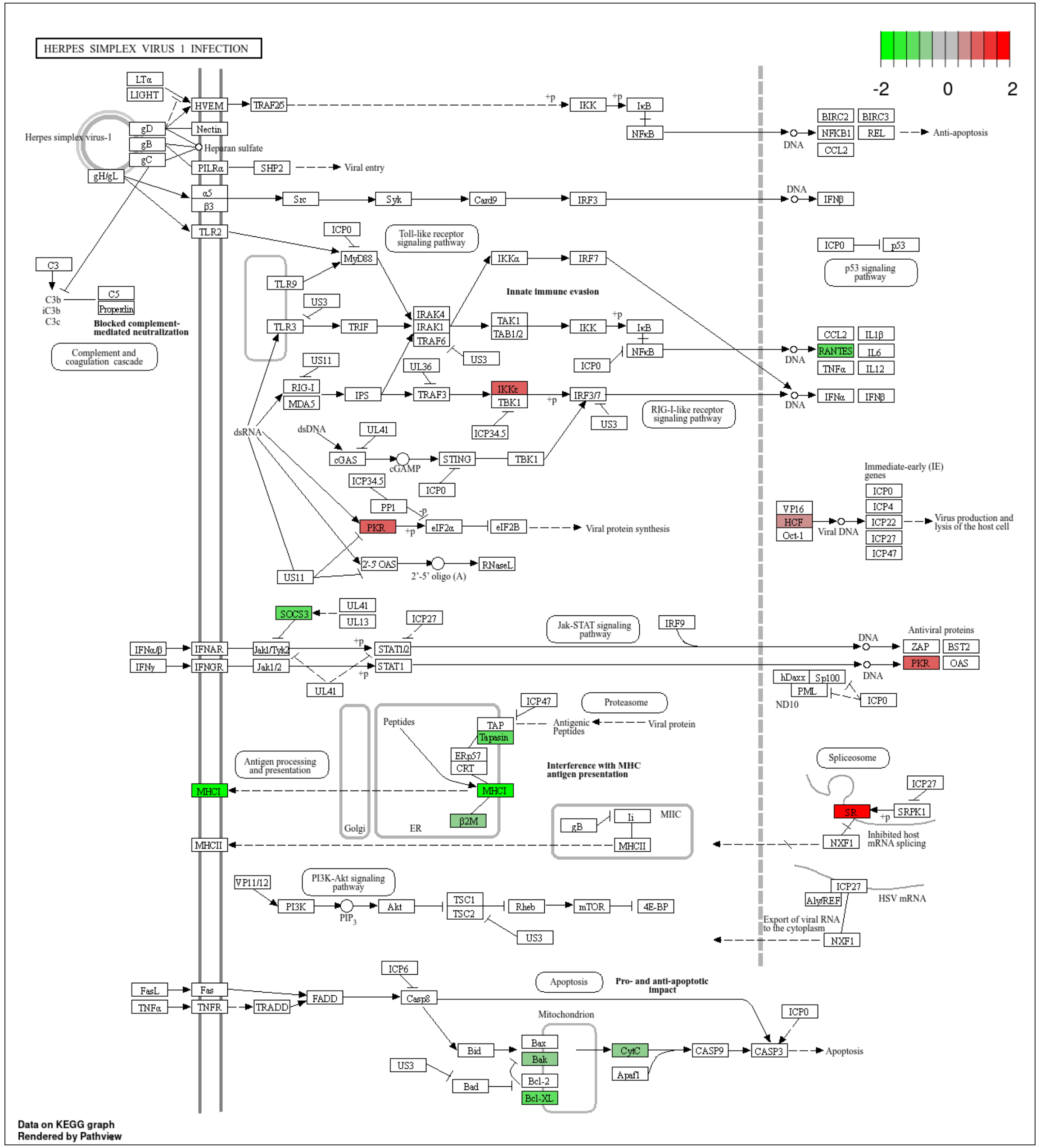

Figure S42. Herpes simplex virus 1 infection pathway in SYNs group in F3 (Cecal tonsils).

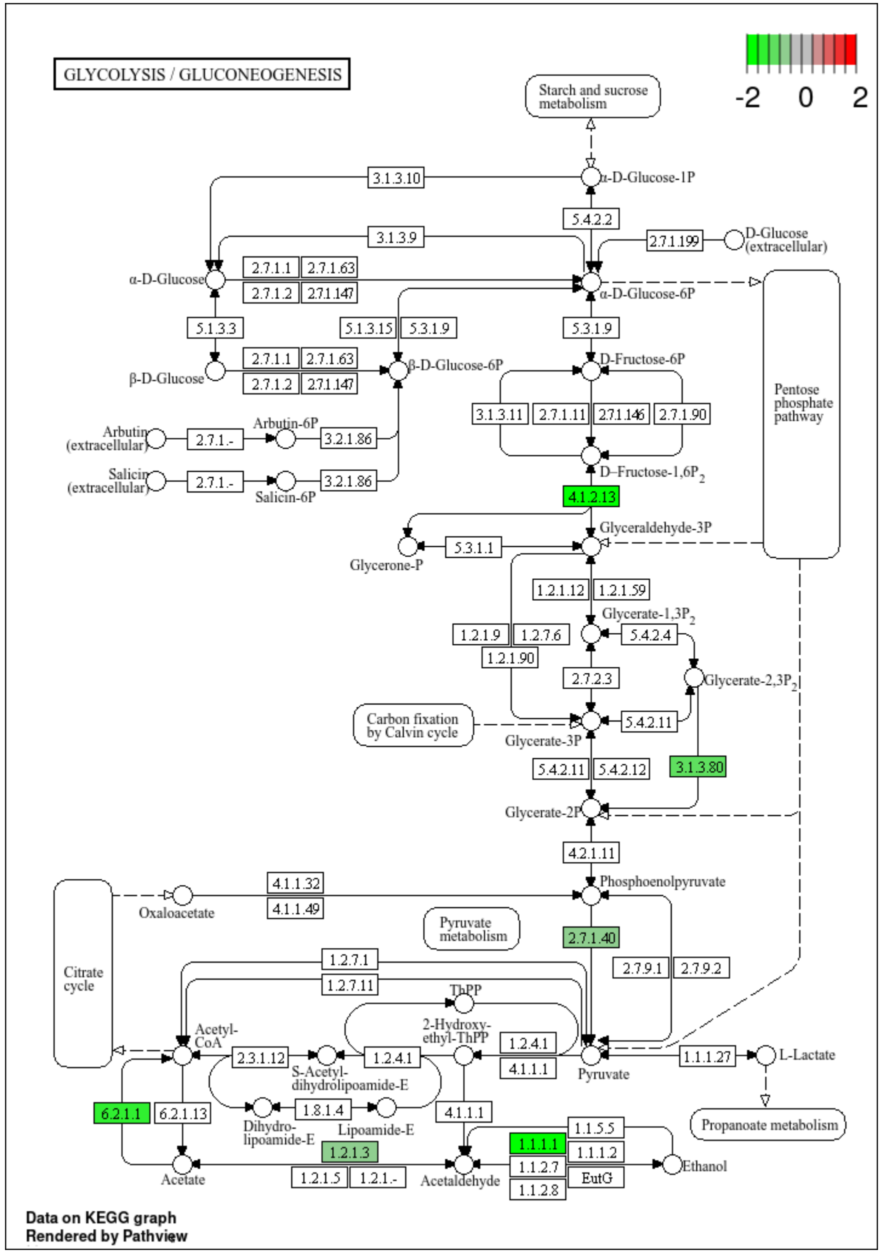

Figure S43. Glycolysis/ gluconeogenesis pathway in SYNs group in F3 (Cecal tonsils).

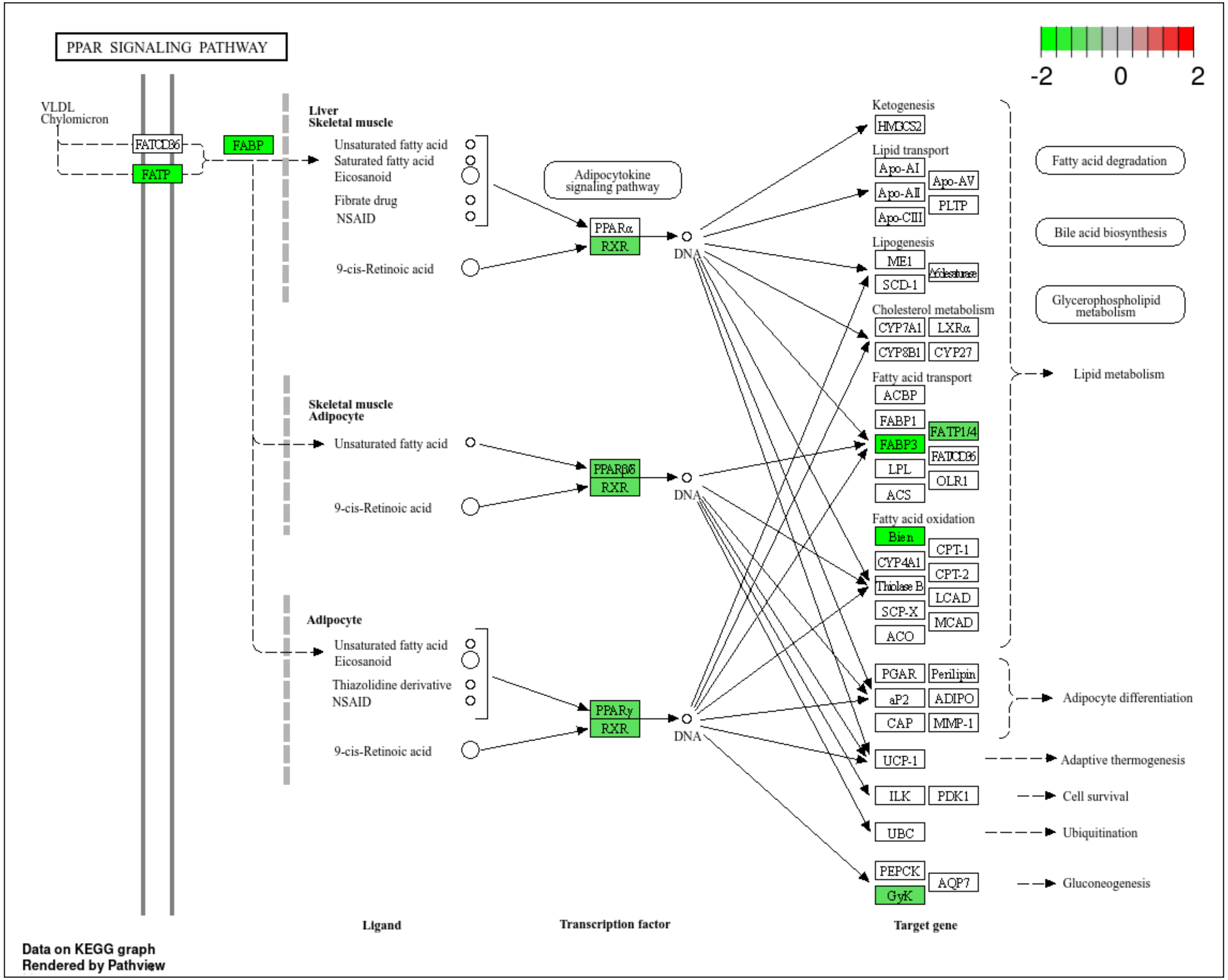

Figure S44. PPAR signaling pathway in SYNs group in F3 (Cecal tonsils).

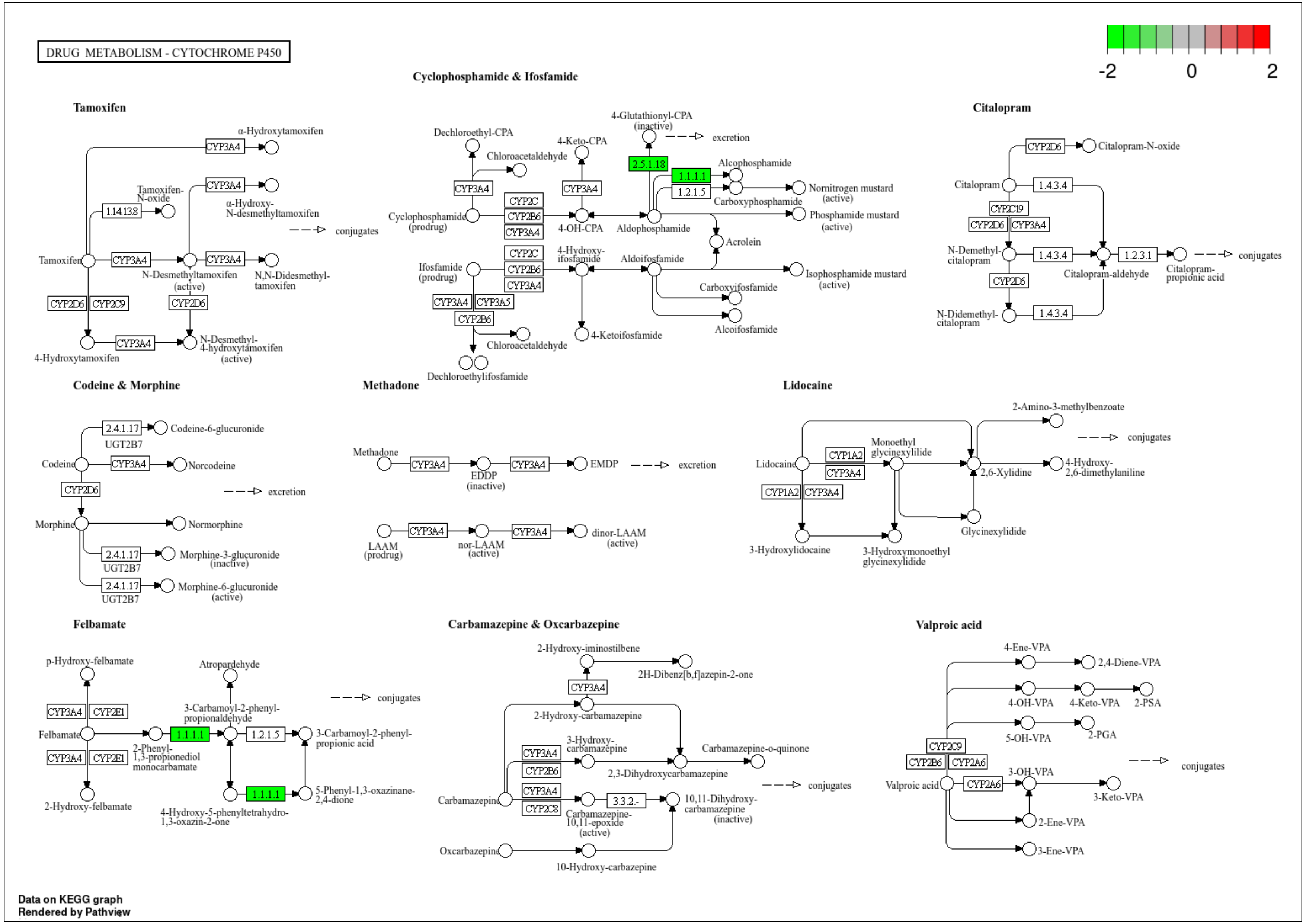

Figure S45. Drug metabolism – cytochrome P450 pathway in SYNs group in F3 (Cecal tonsils).

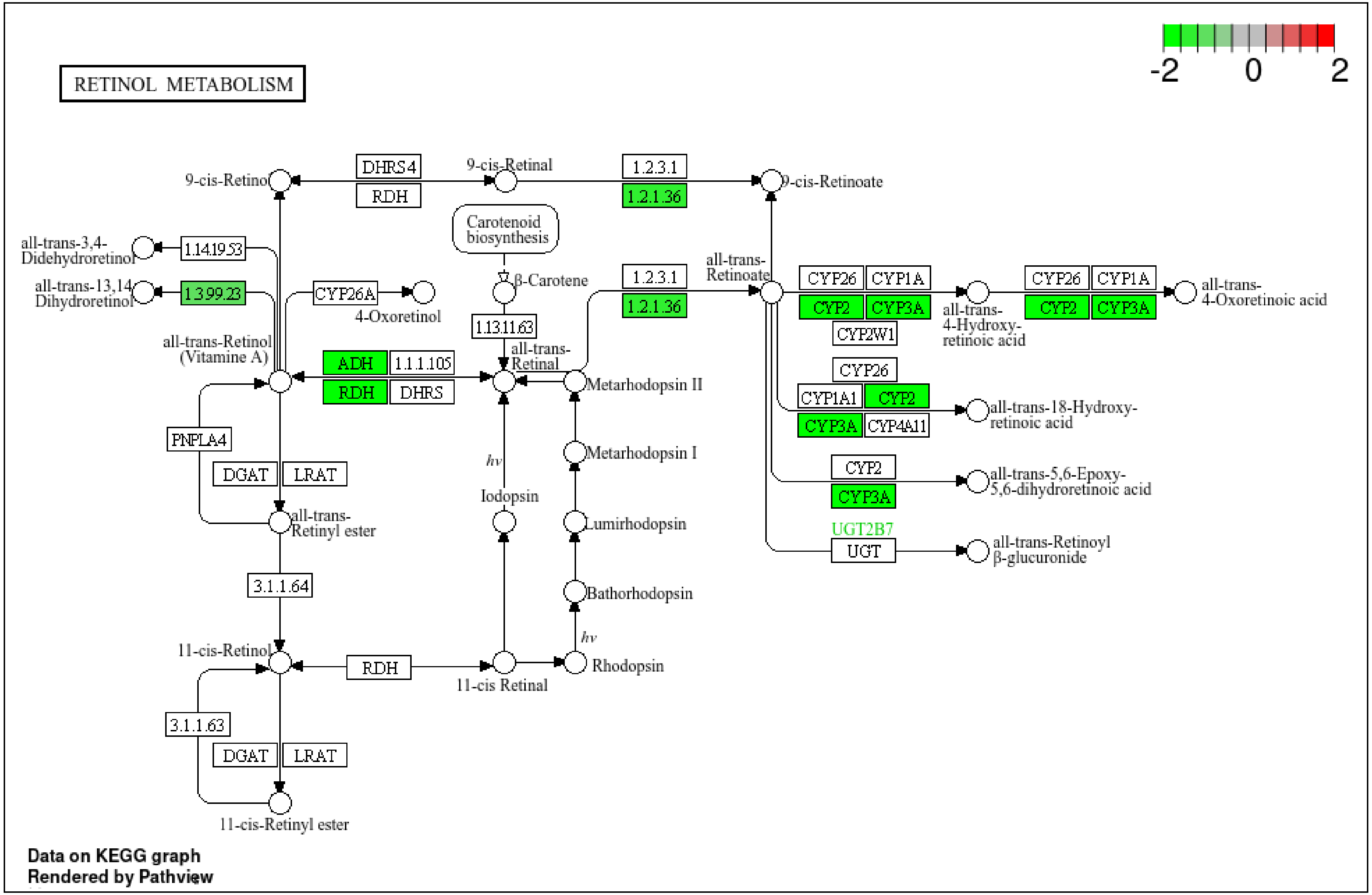

Figure S46. Retinol metabolism pathway in SYNs group in F3 (Cecal tonsils).

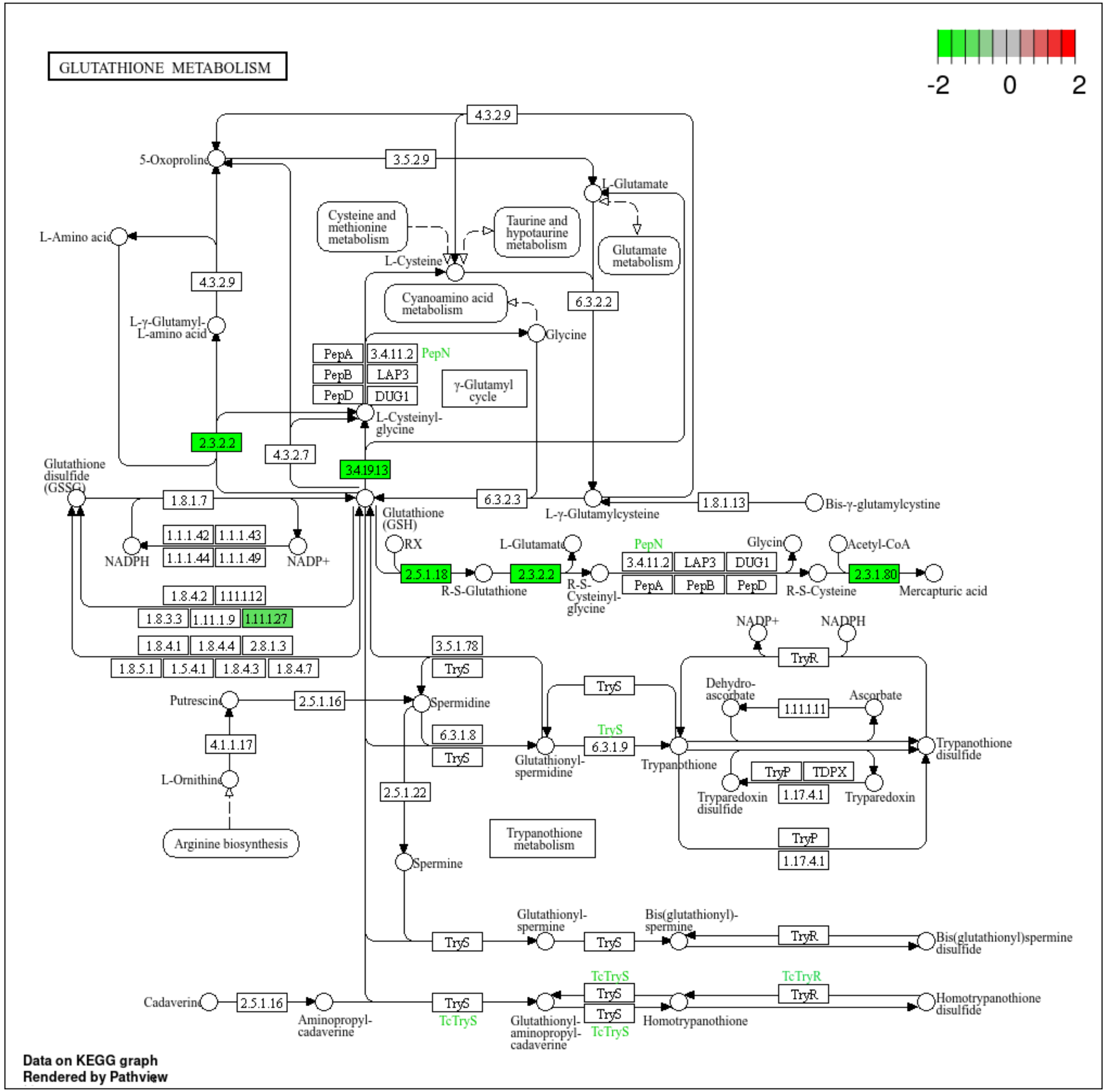

Figure S47. Glutathione metabolism pathway in SYNs group in F3 (Cecal tonsils).

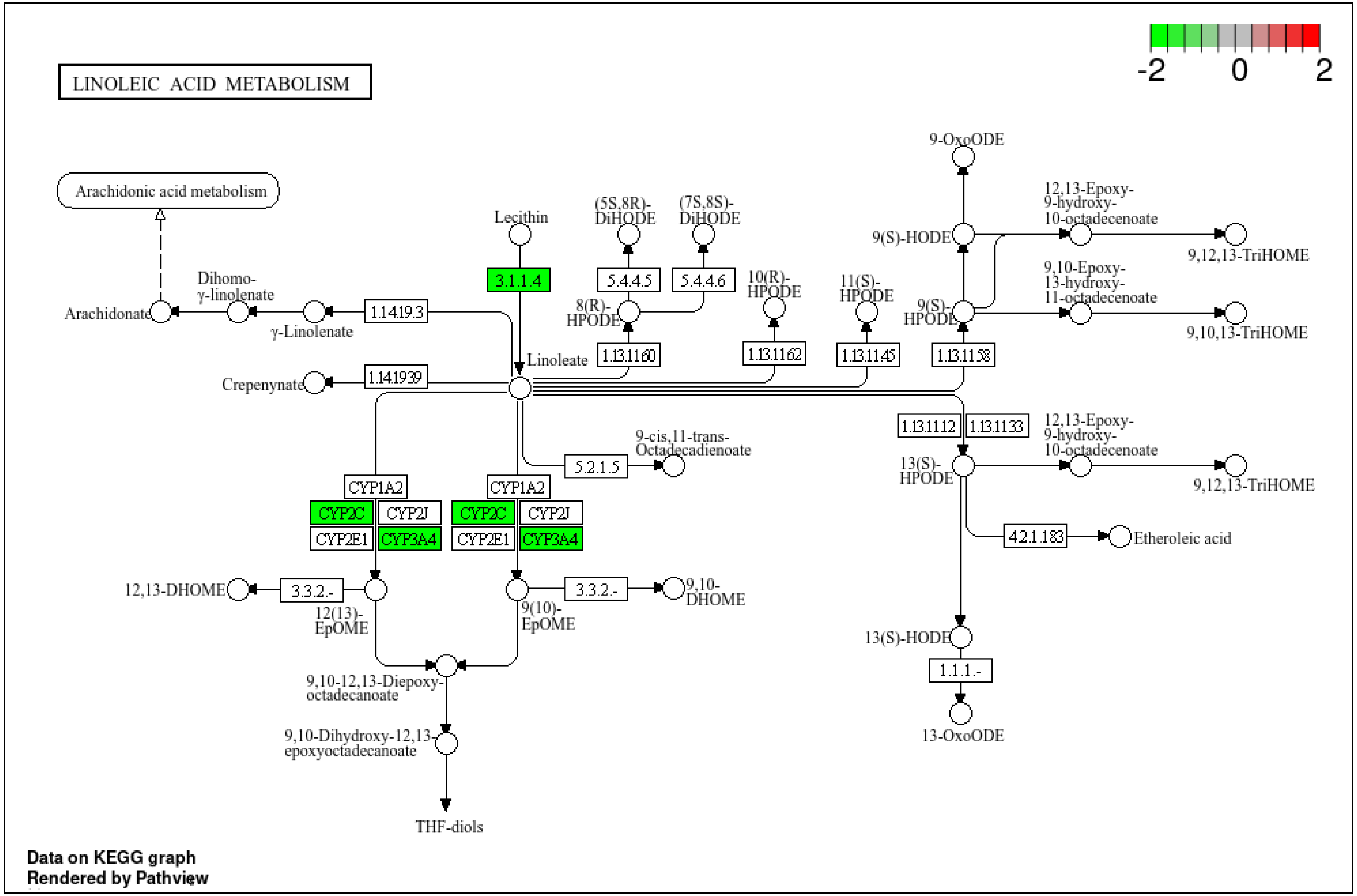

Figure S48. Linoleic acid metabolism pathway in SYNs group in F3 (Cecal tonsils).

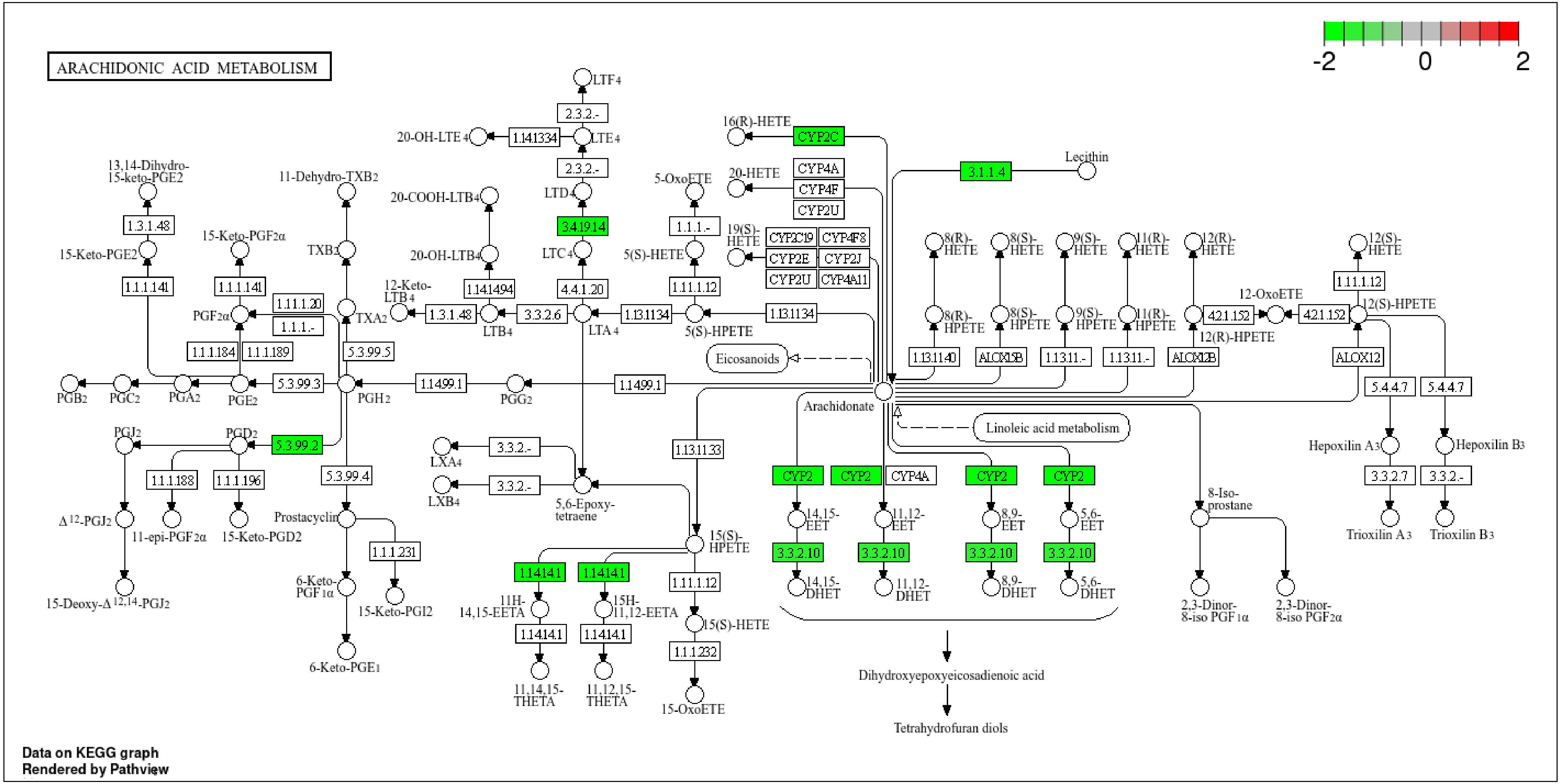

Figure S49. Arachidonic acid metabolism pathway in SYNs group in F3 (Cecal tonsils).

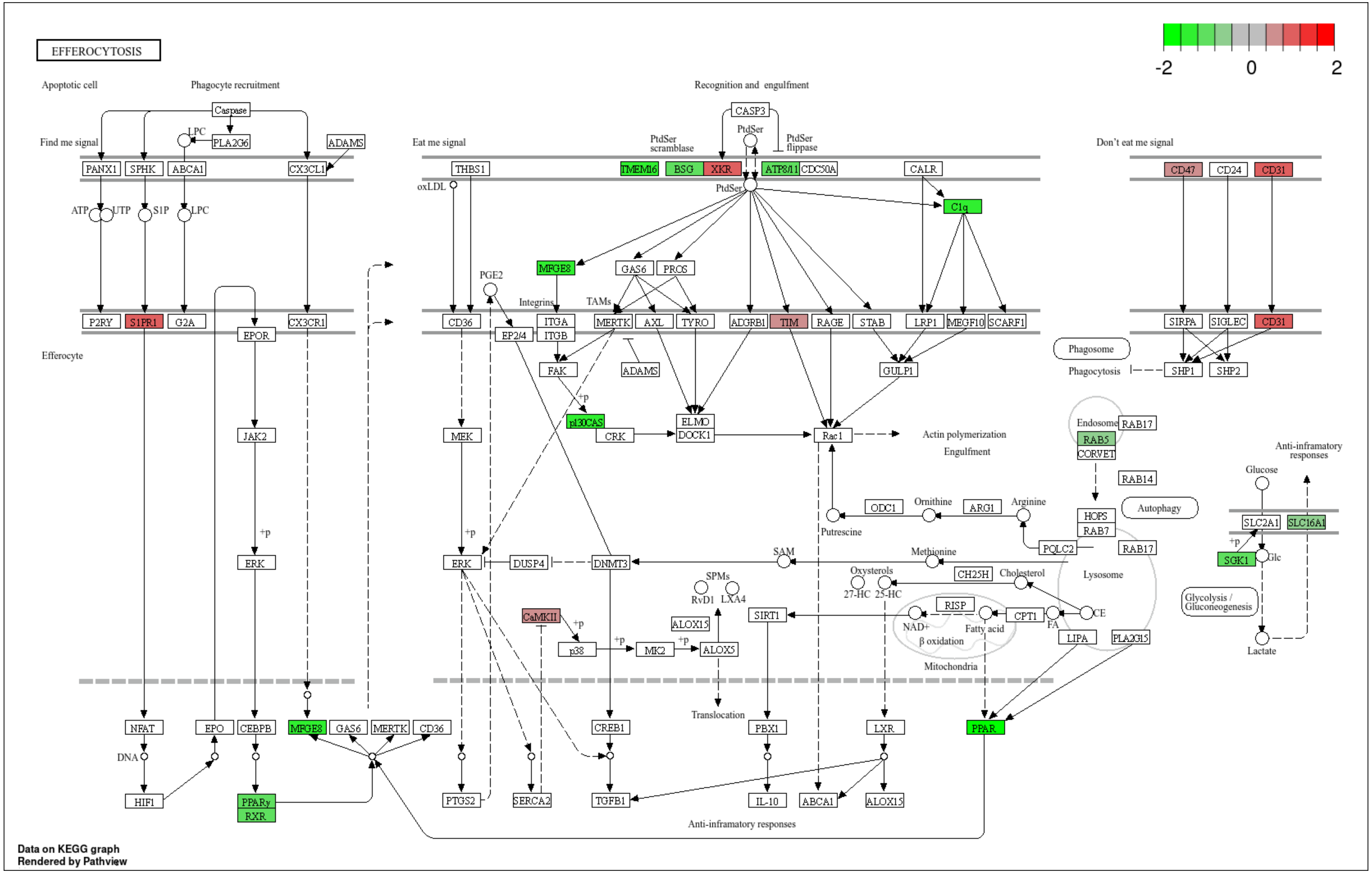

Figure S50. Efferocytosis pathway in SYNs group in F3 (Cecal tonsils).

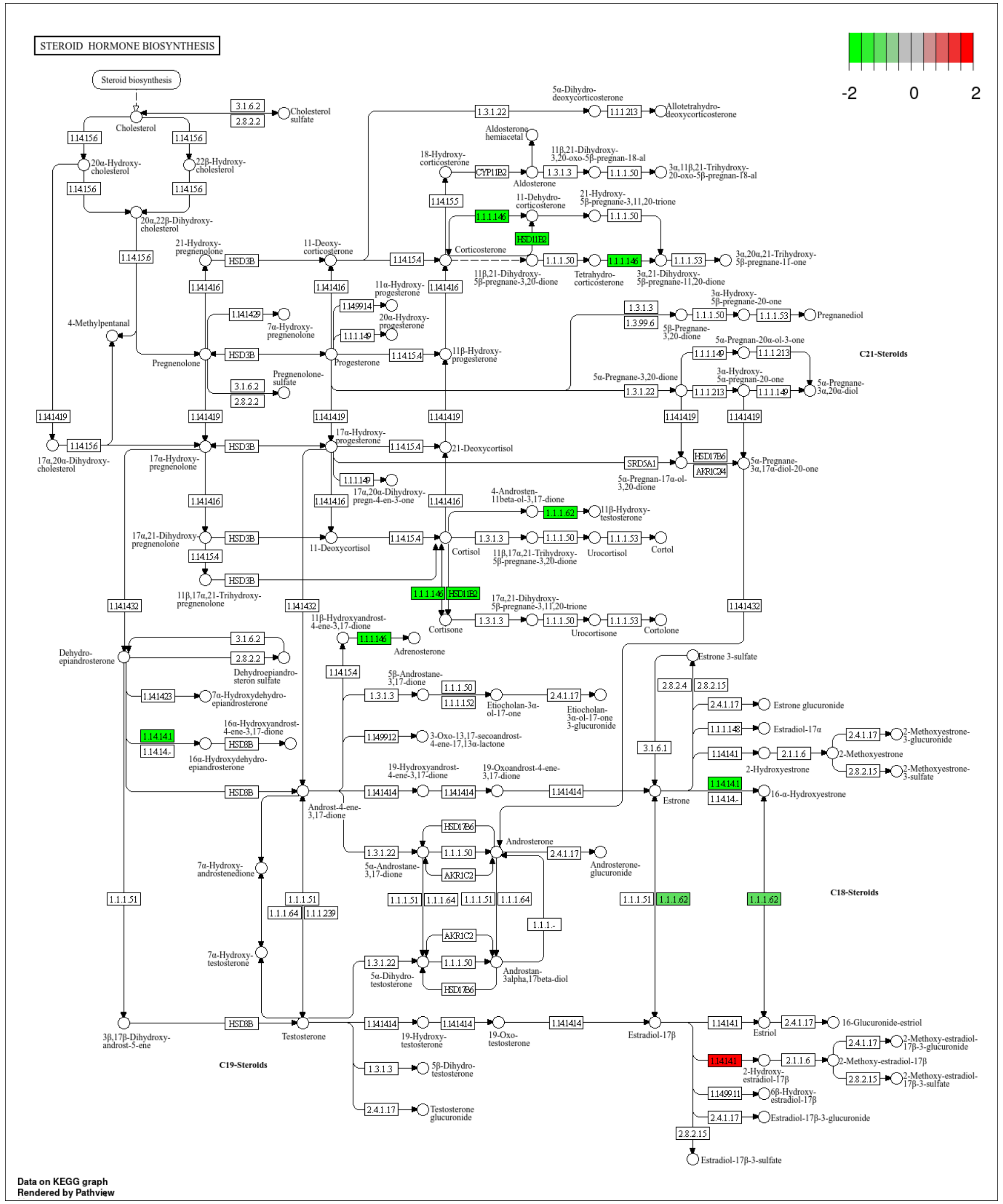

Figure S51. Steroid hormone biosynthesis pathway in SYNs group in F3 (Cecal tonsils).

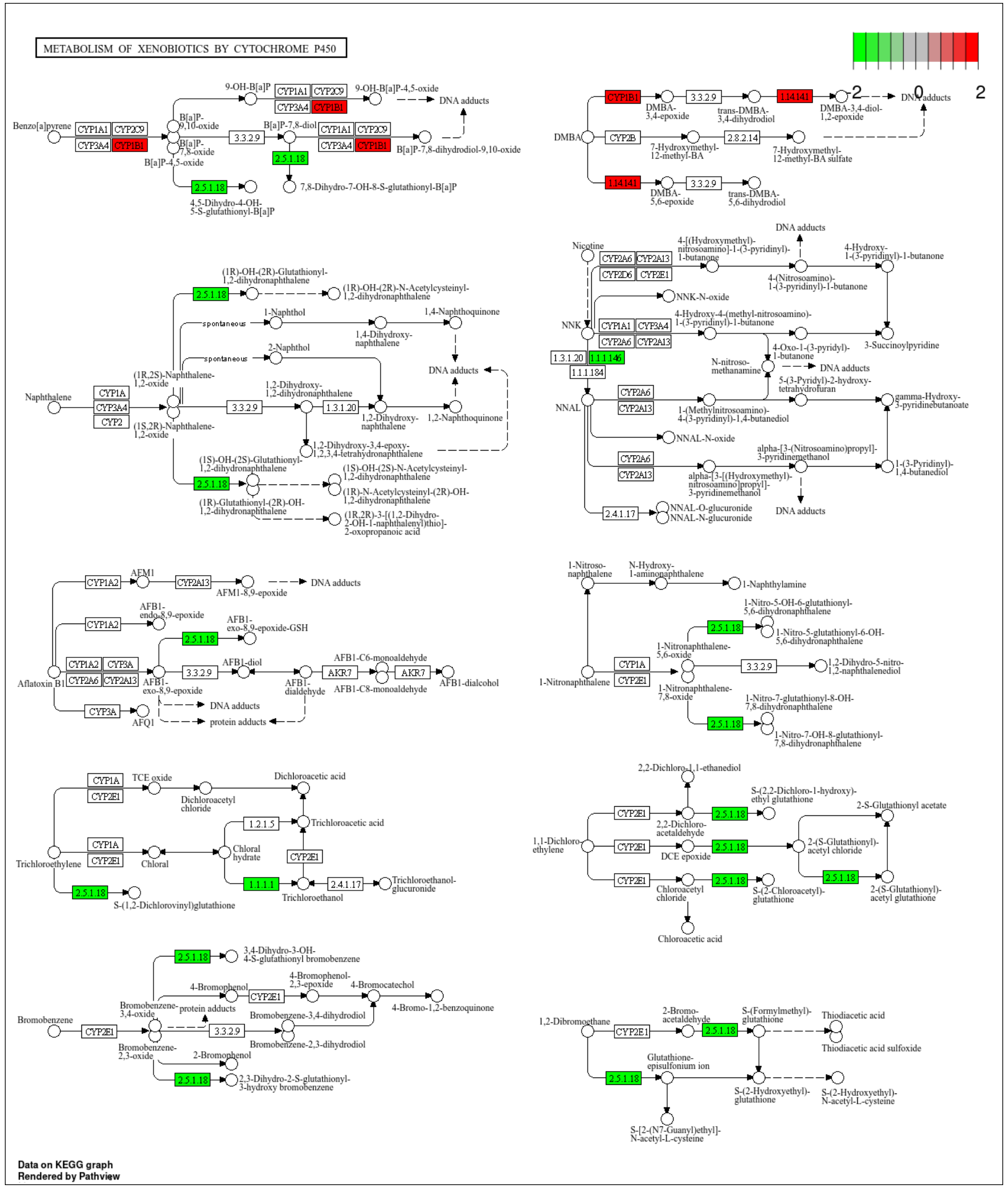

Figure S52. Metabolism of xenobiotics by cytochrome P450 pathway in SYNs group in F3 (Cecal tonsils).

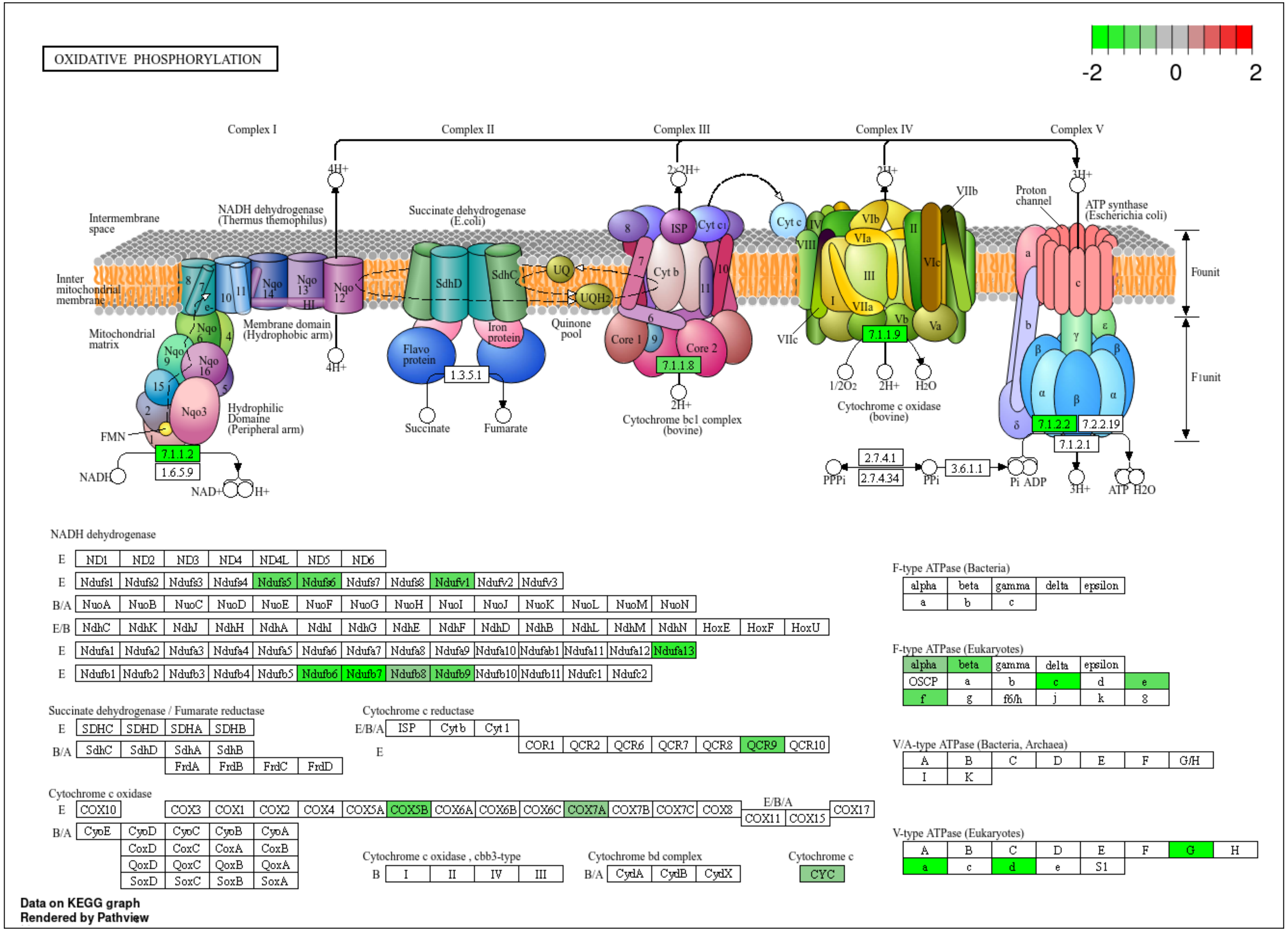

Figure S53. Oxidative phosphorylation pathway in SYNs group in F3 (Cecal tonsils).

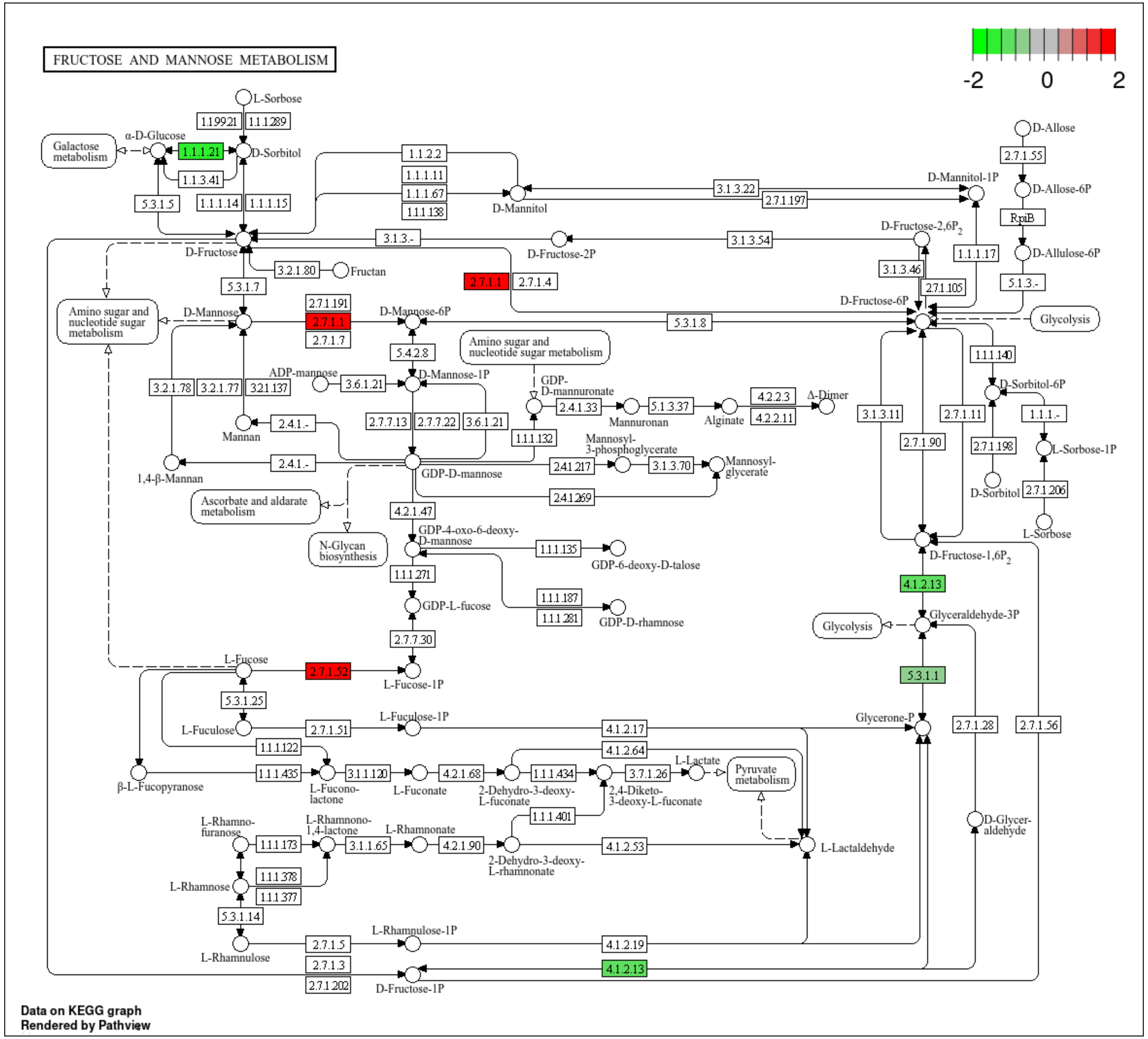

Figure S54. Fructose and mannose metabolism pathway in SYNr group in F3 (Cecal tonsils).

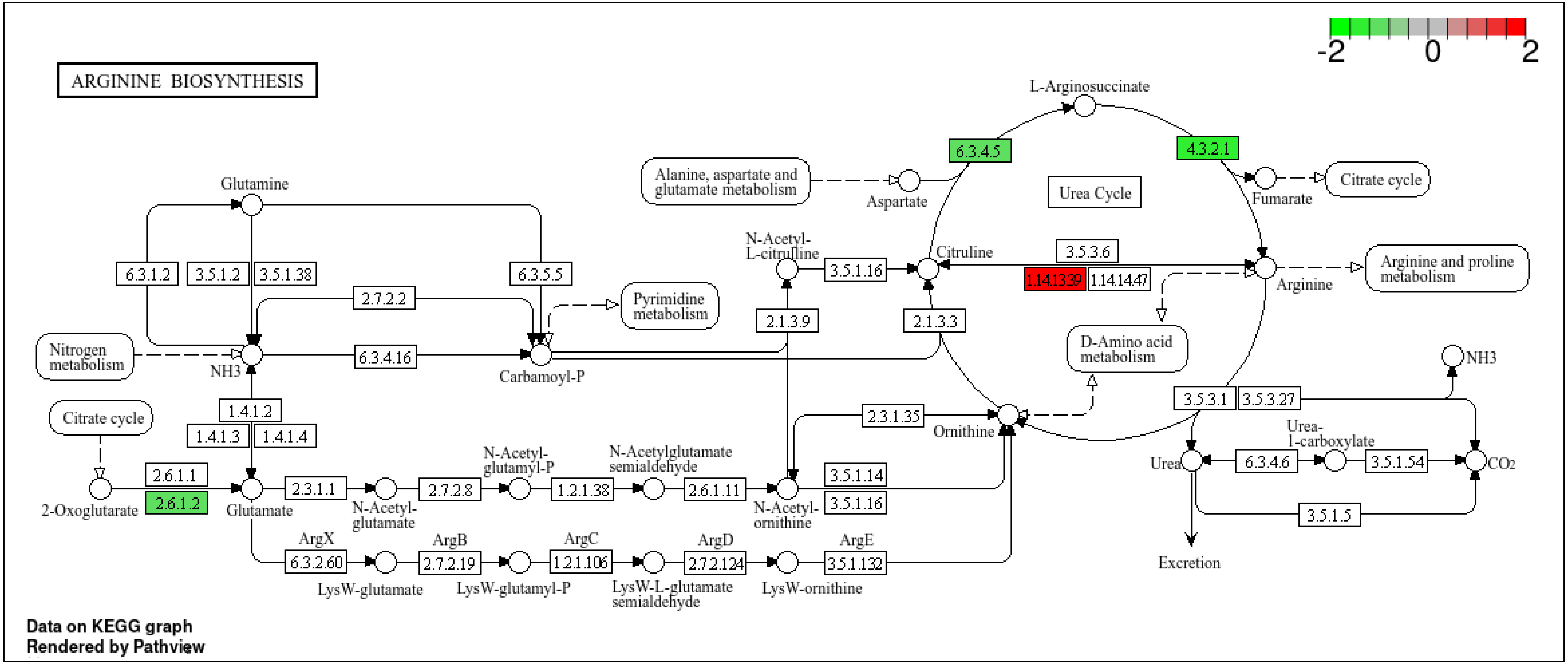

Figure S55. Arginine biosynthesis pathway in SYNr group in F3 (Cecal tonsils).

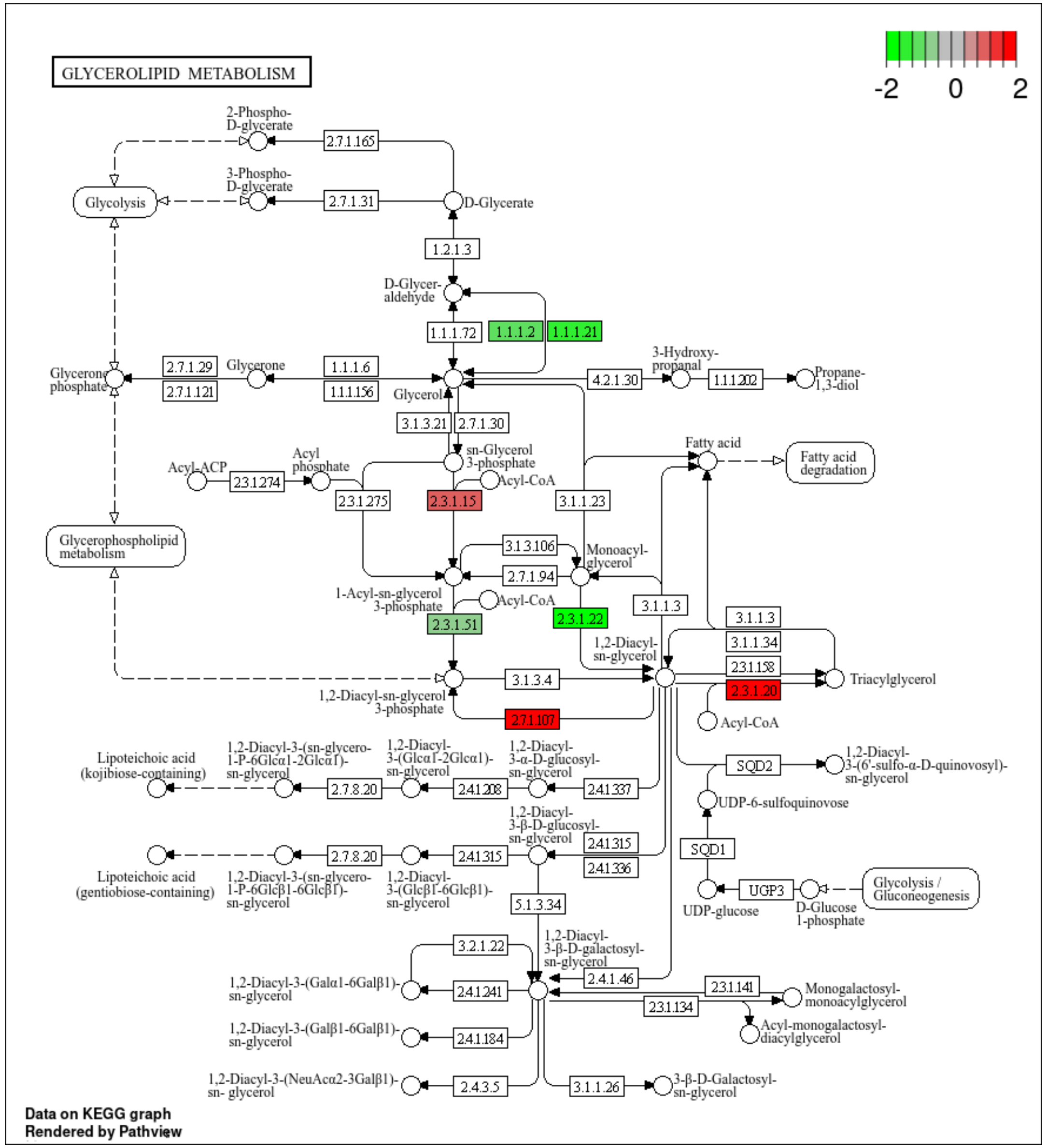

Figure S56. Glycerolipid metabolism pathway in SYNr group in F3 (Cecal tonsils).

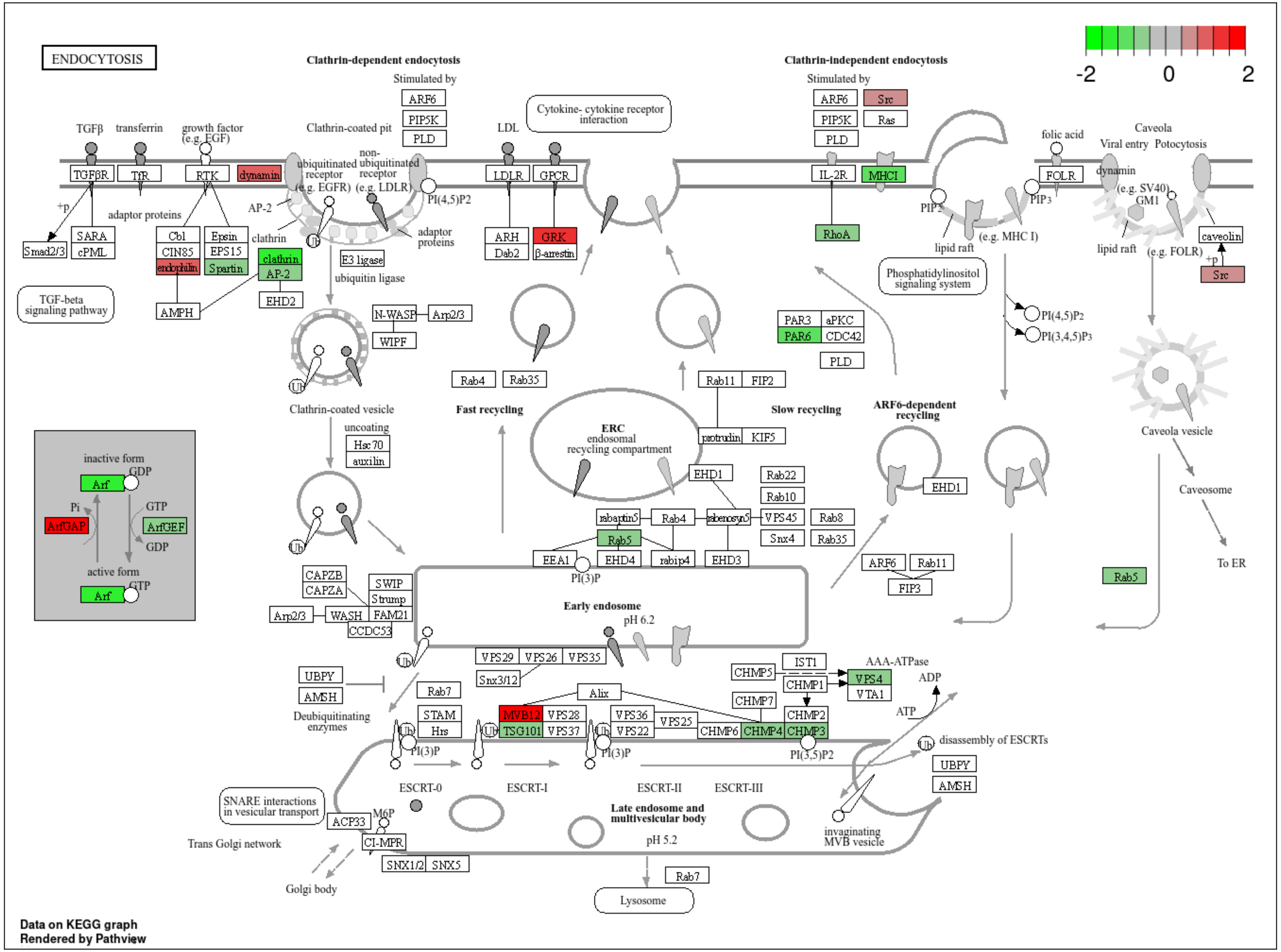

Figure S57. Endocytosis pathway in SYNr group in F3 (Cecal tonsils).

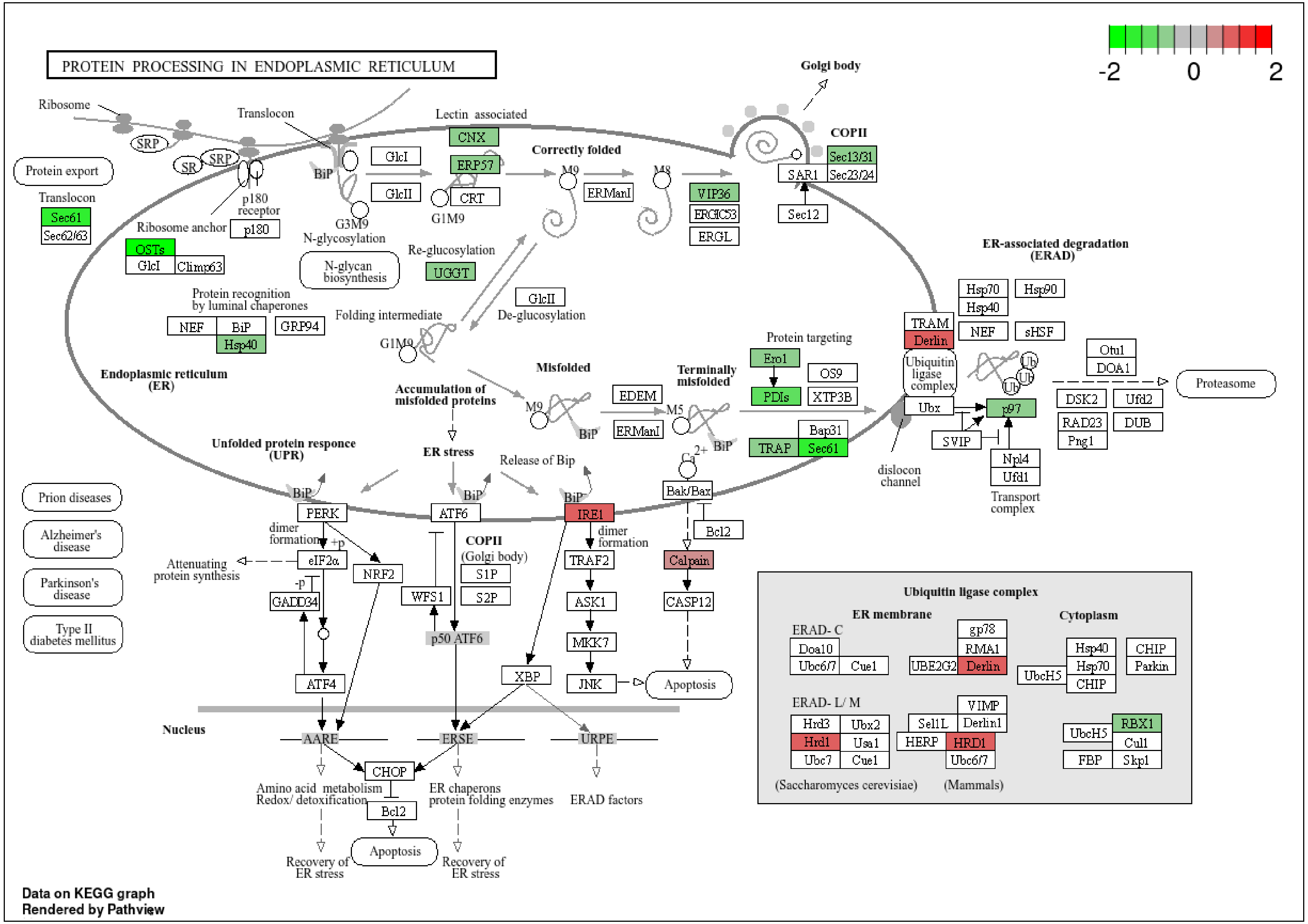

Figure S58. Proton processing in endoplasmic reticulum pathway in SYNr group in F3 (Cecal tonsils).

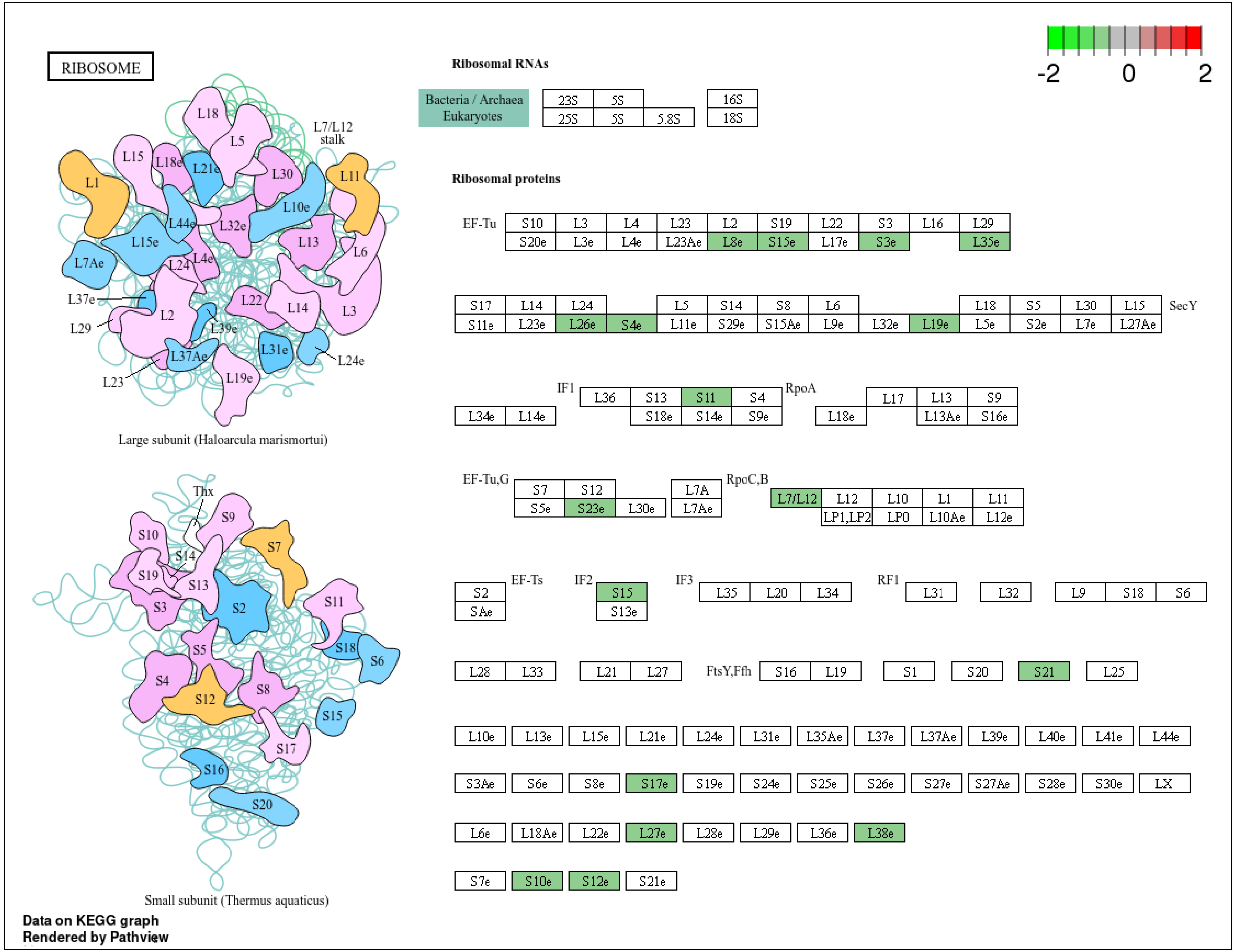

Figure S59. Ribosome pathway in SYNr group in F3 (Cecal tonsils).

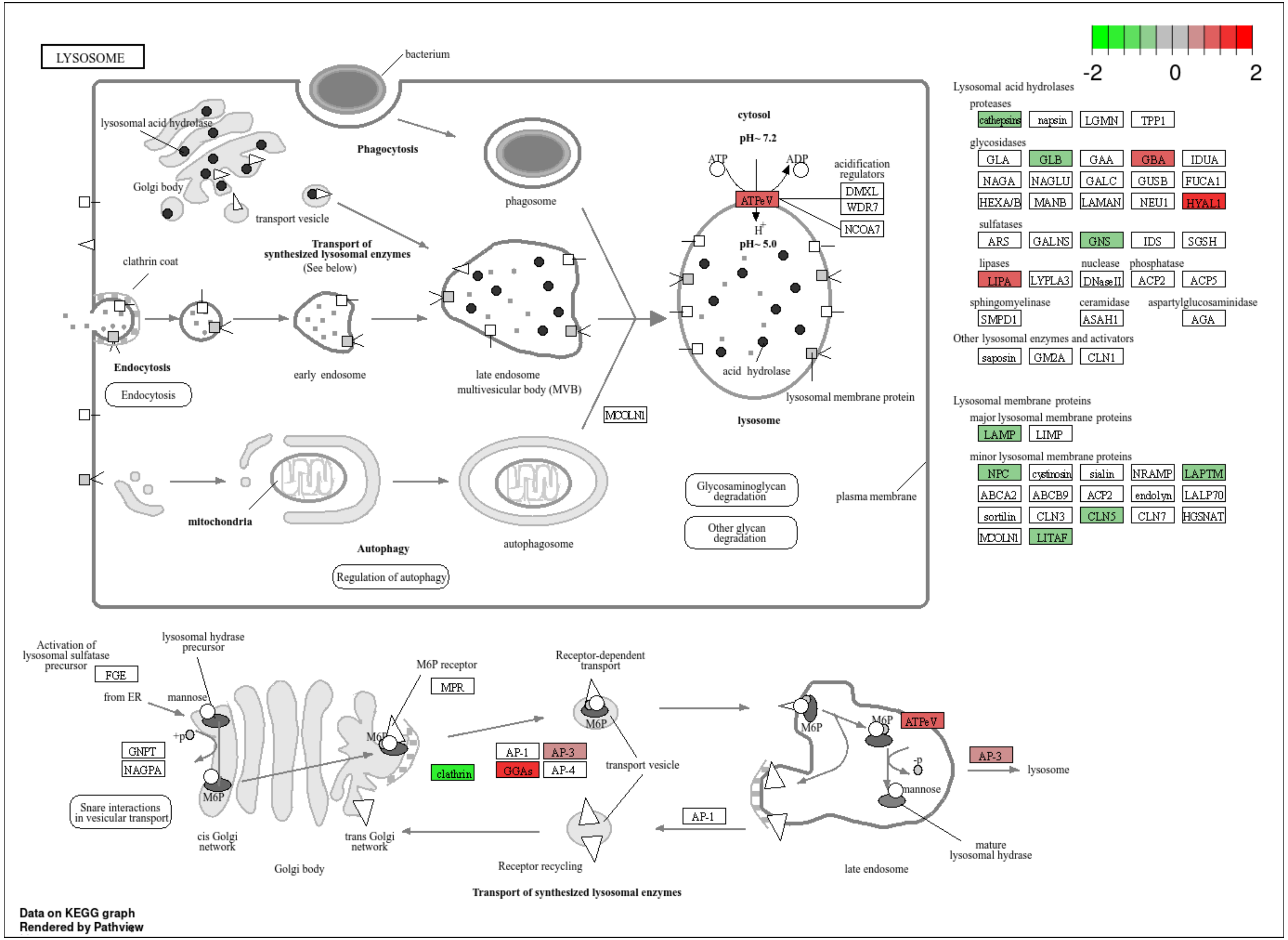

Figure S60. lysosome pathway in SYNr group in F3 (Cecal tonsils).

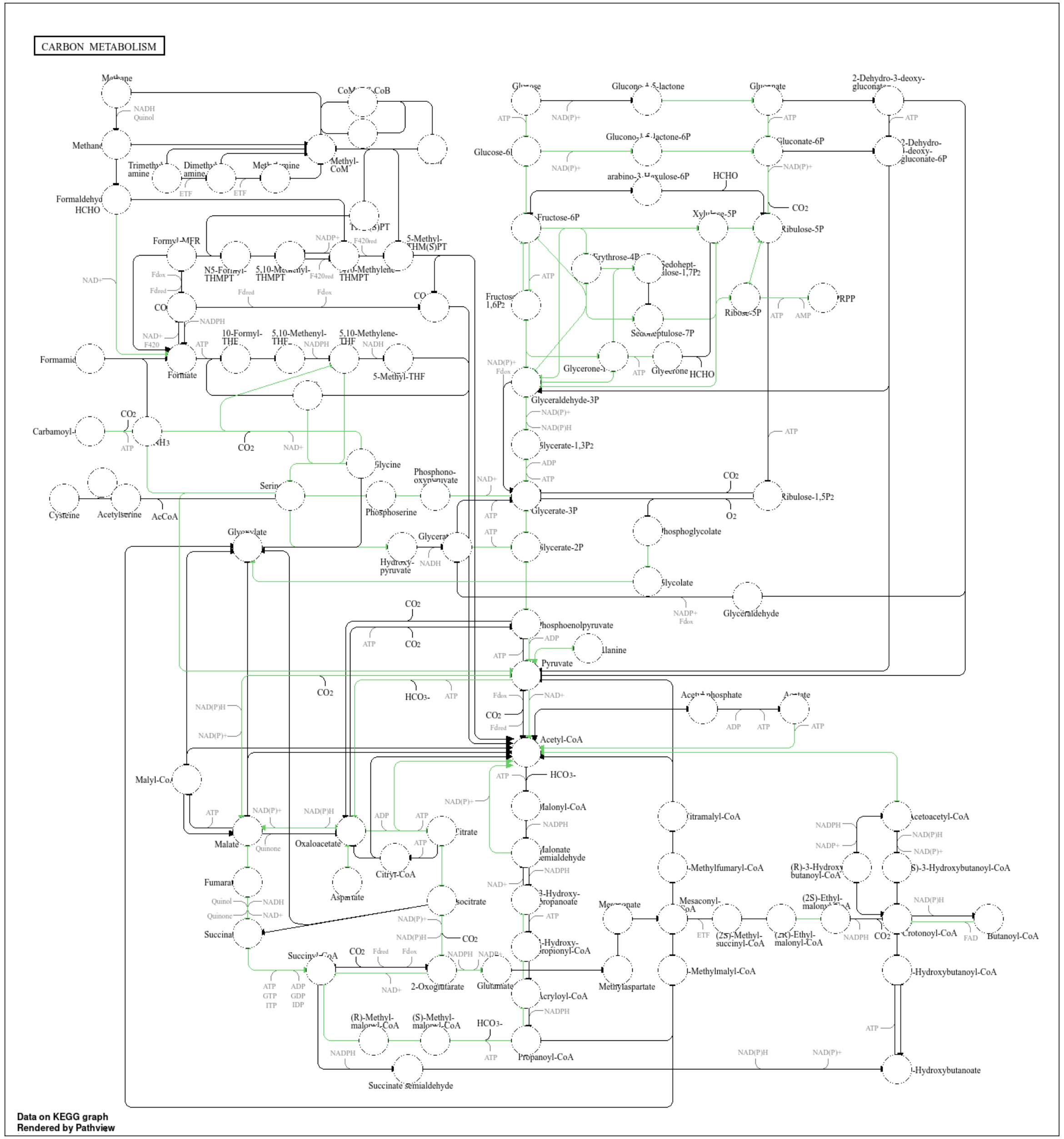

Figure S61. Carbon metabolism pathway in SYNr group in F3 (Cecal tonsils).

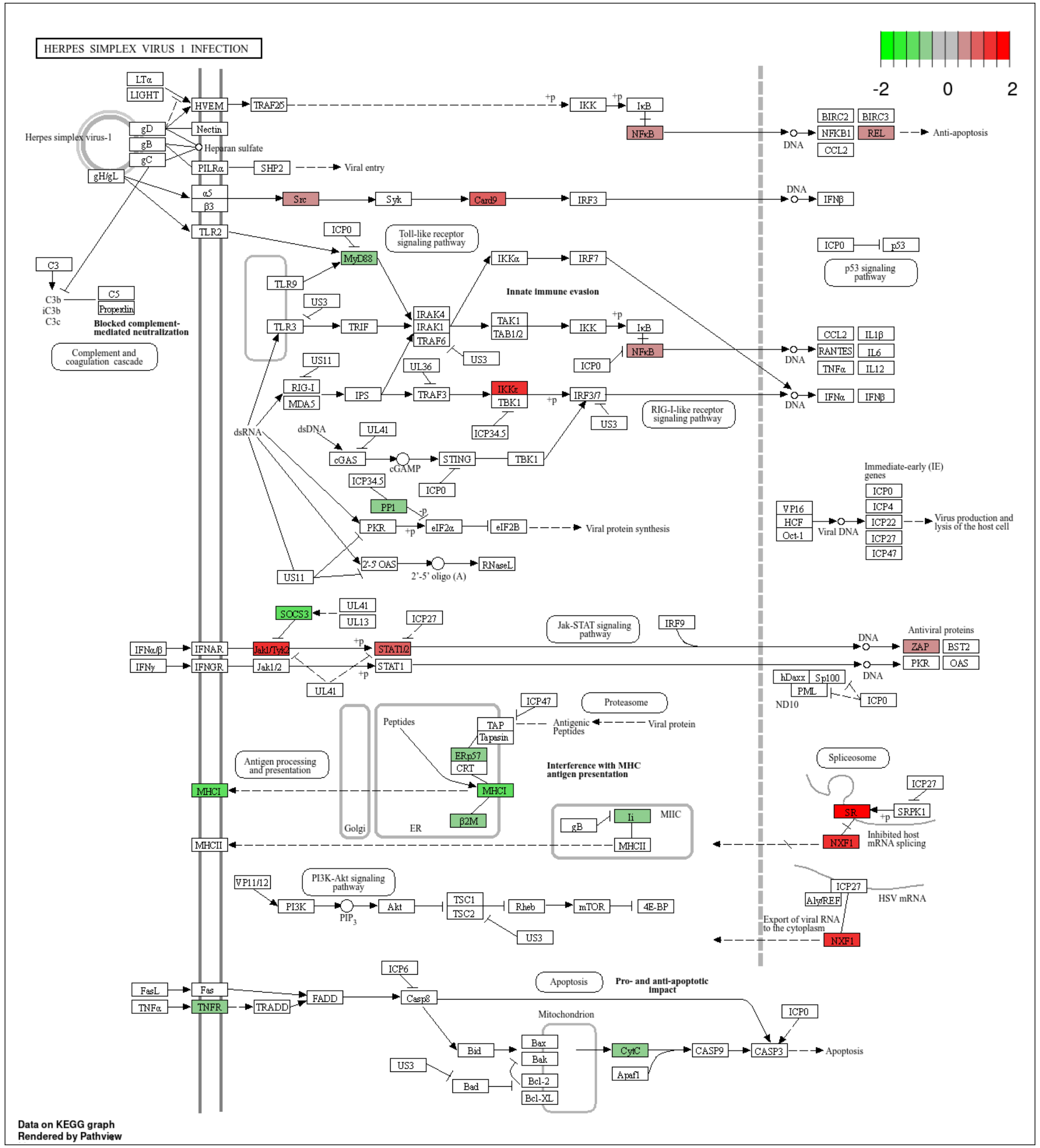

Figure S62. Herpes simplex virus 1 infection pathway in SYNr group in F3 (Cecal tonsils).

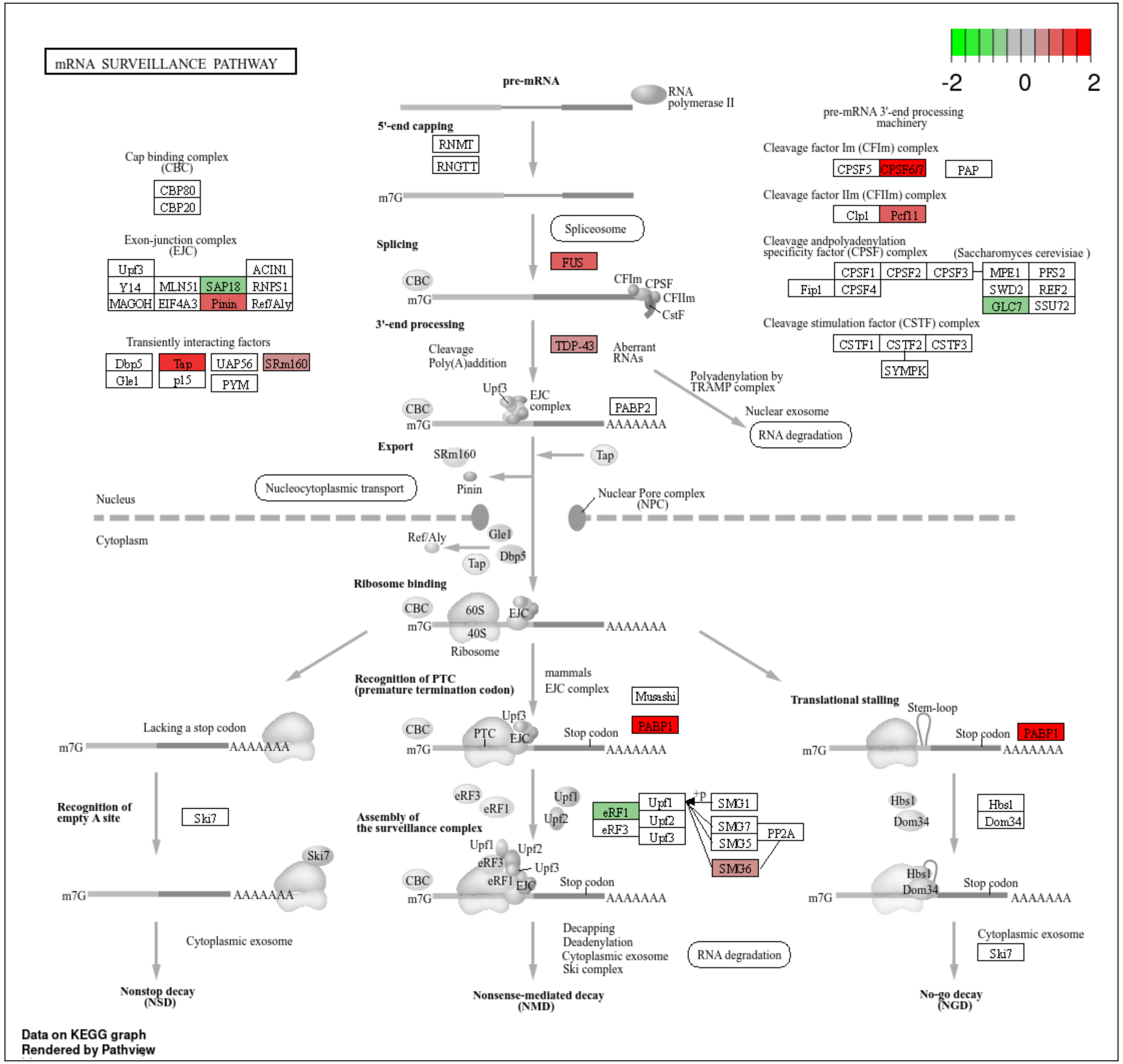

Figure S63. mRNA surveillance pathway in SYNr group in F3 (Cecal tonsils).

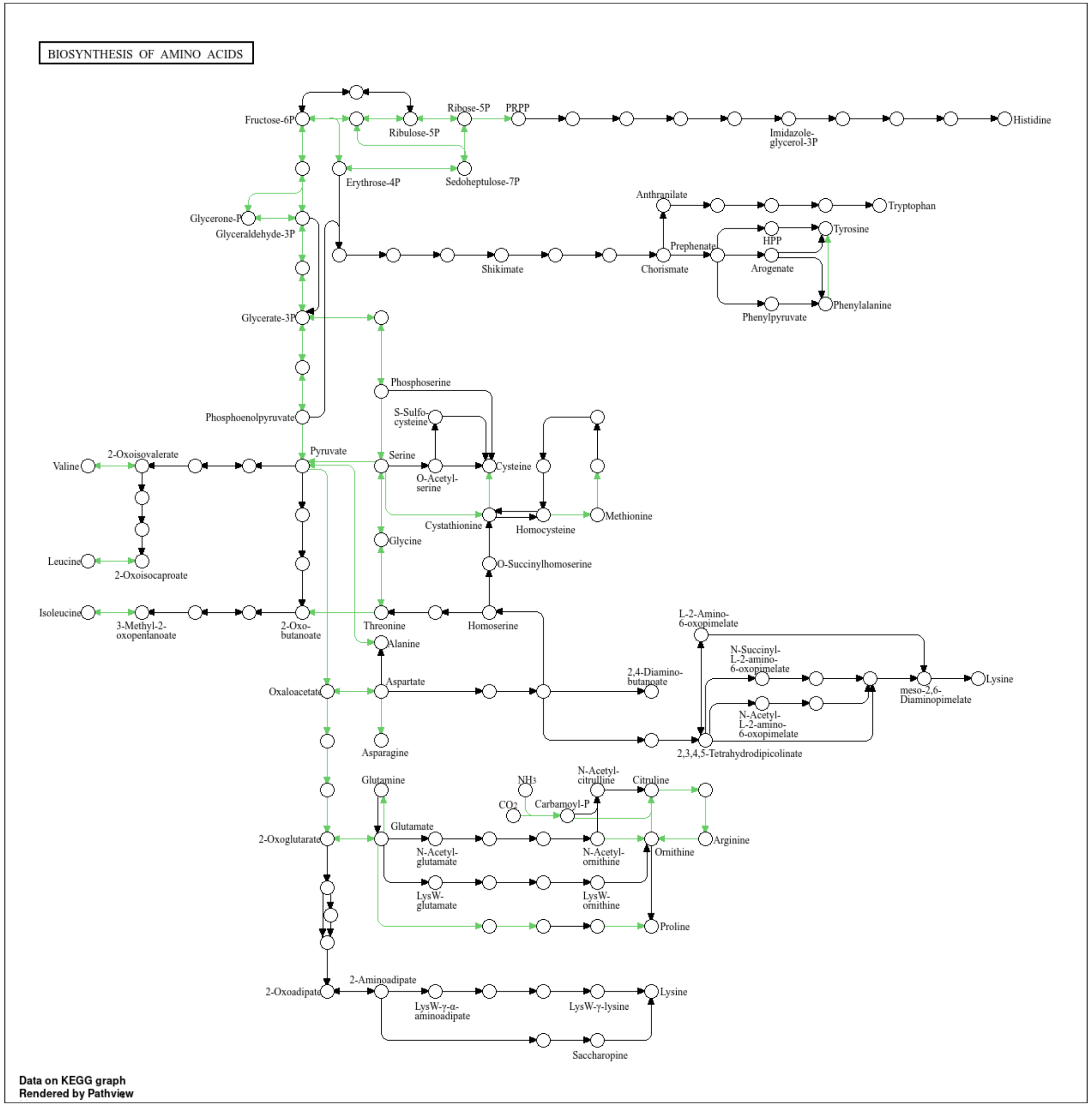

Figure S64. Biosynthesis of amino acids pathway in SYNr group in F3 (Cecal tonsils).

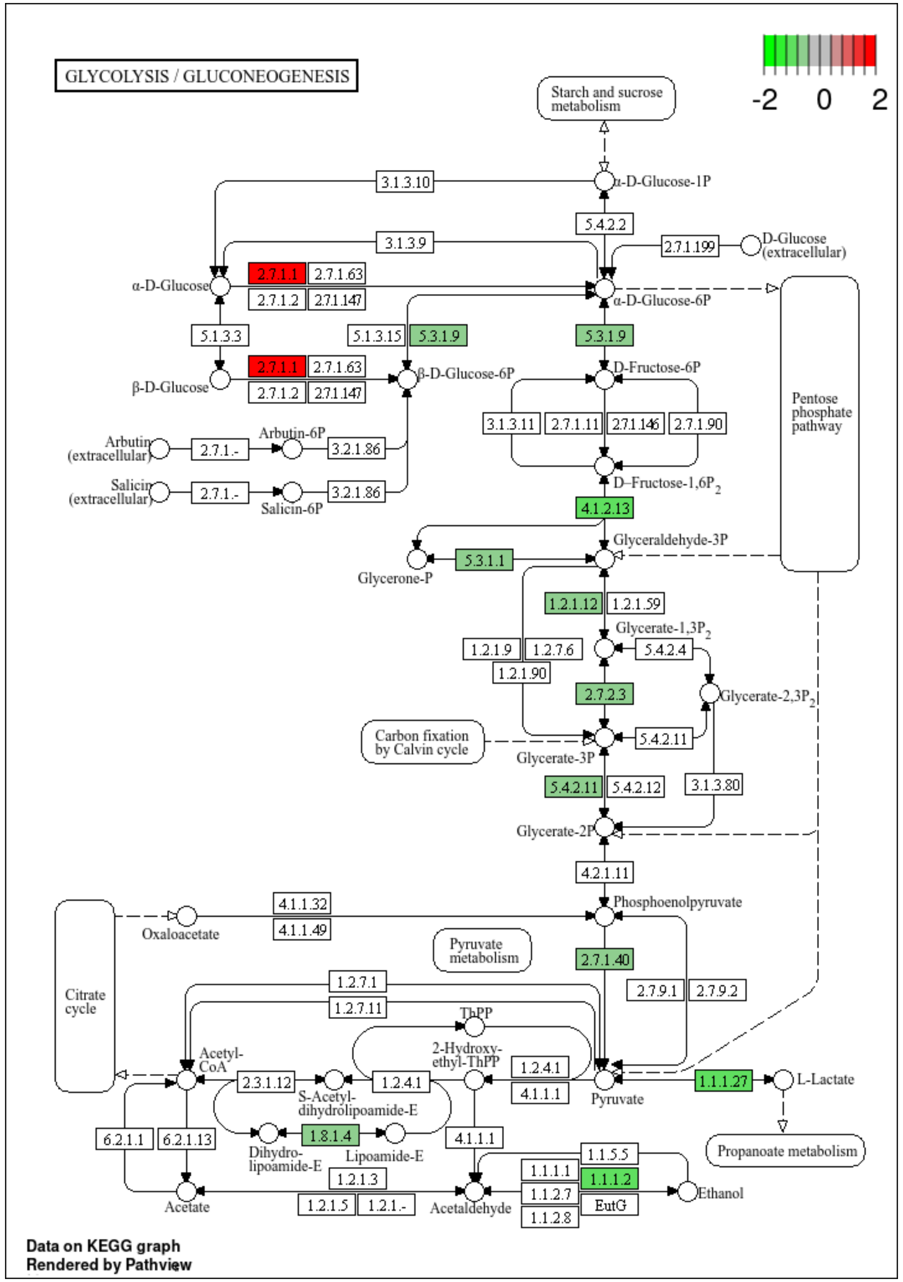

Figure S65. Glycolysis/ gluconeogenesis pathway in SYNr group in F3 (Cecal tonsils).

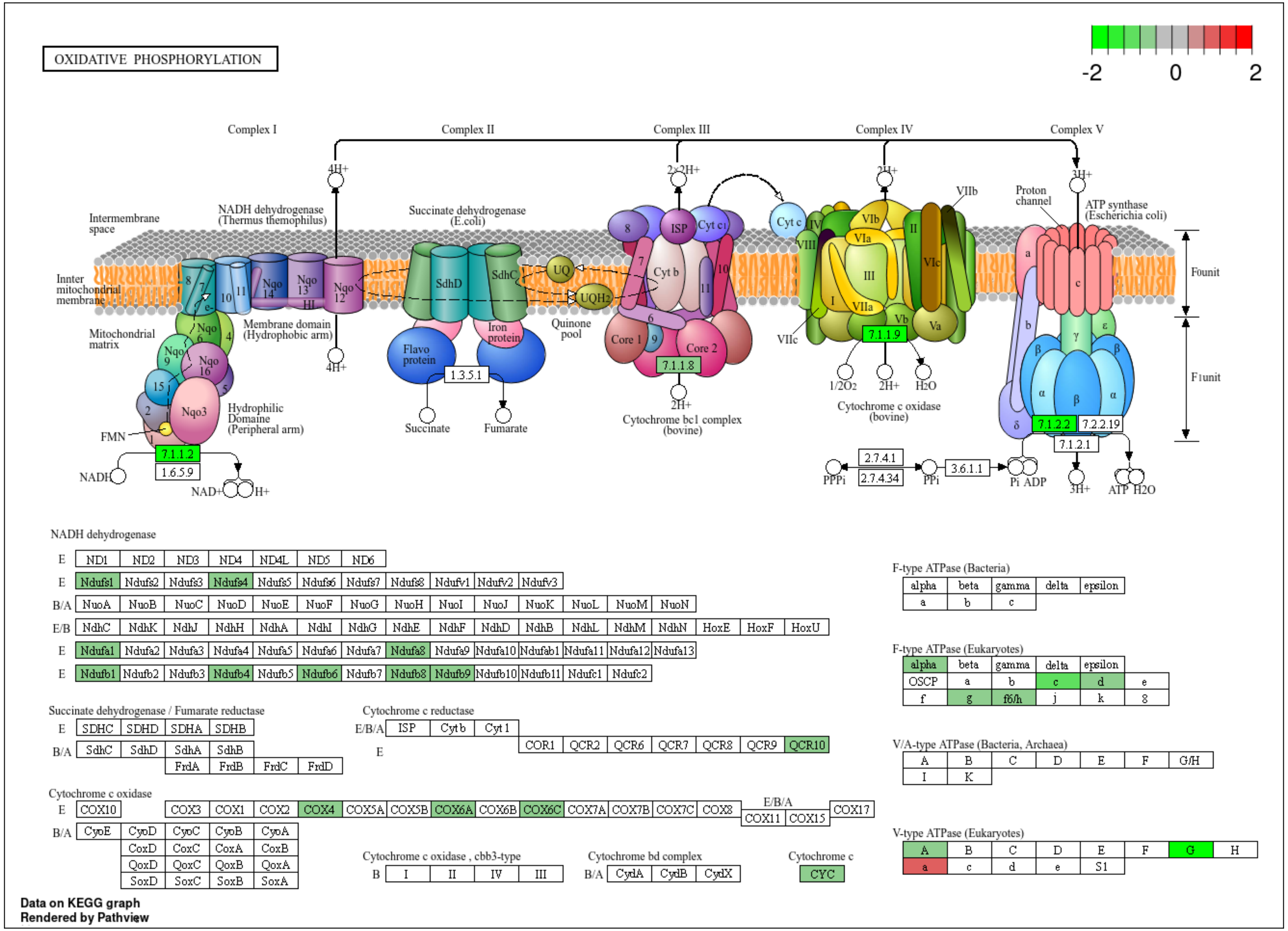

Figure S66. Oxidative phosphorylation pathway in SYNr group in F3 (Cecal tonsils).

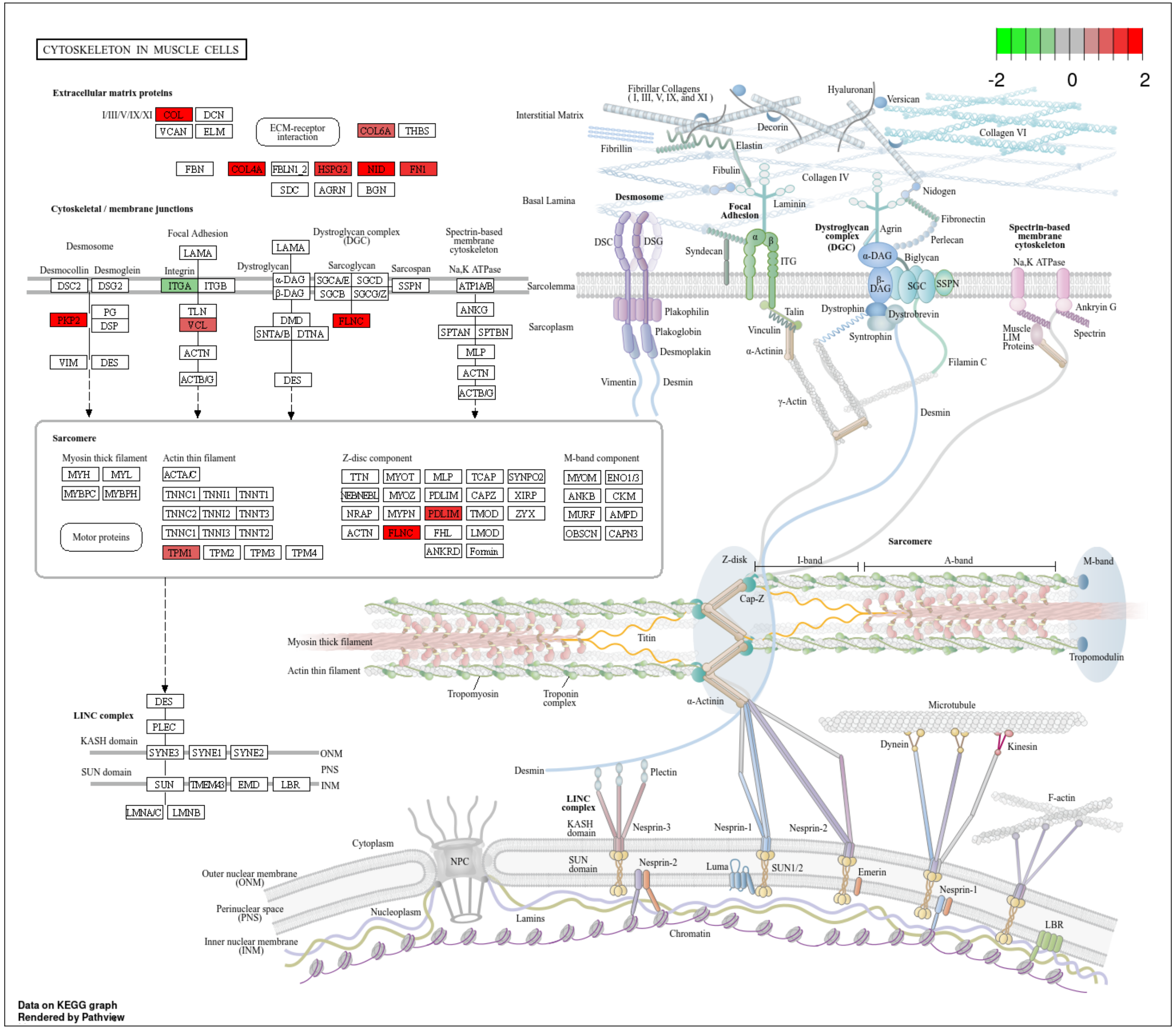

Figure S67. Cytoskeleton in muscle cells pathway in SYNCHs group in F3 (Cecal tonsils).

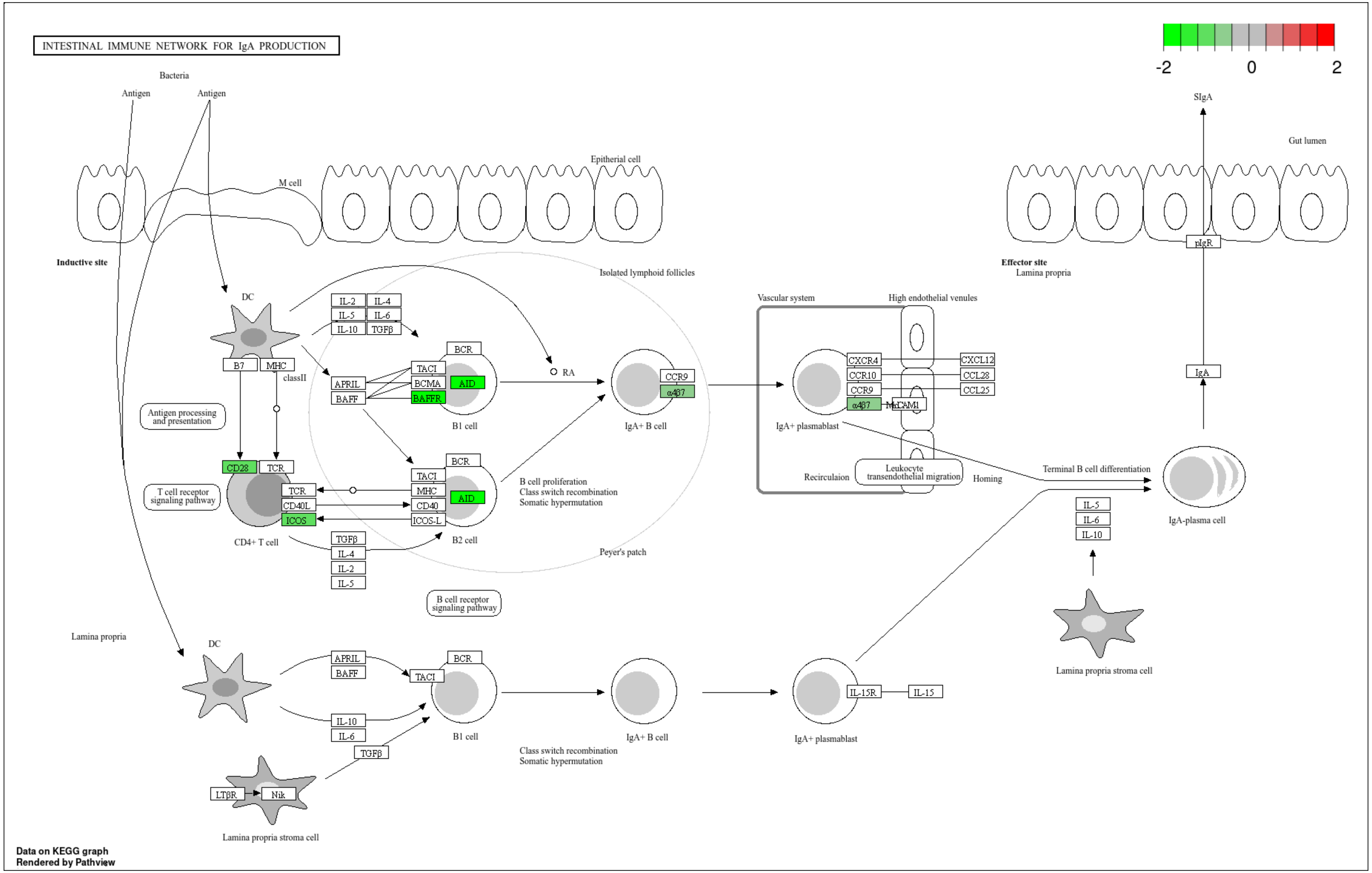

Figure S68. Intestinal immune network for IgA production pathway in SYNCHs group in F3 (Cecal tonsils).

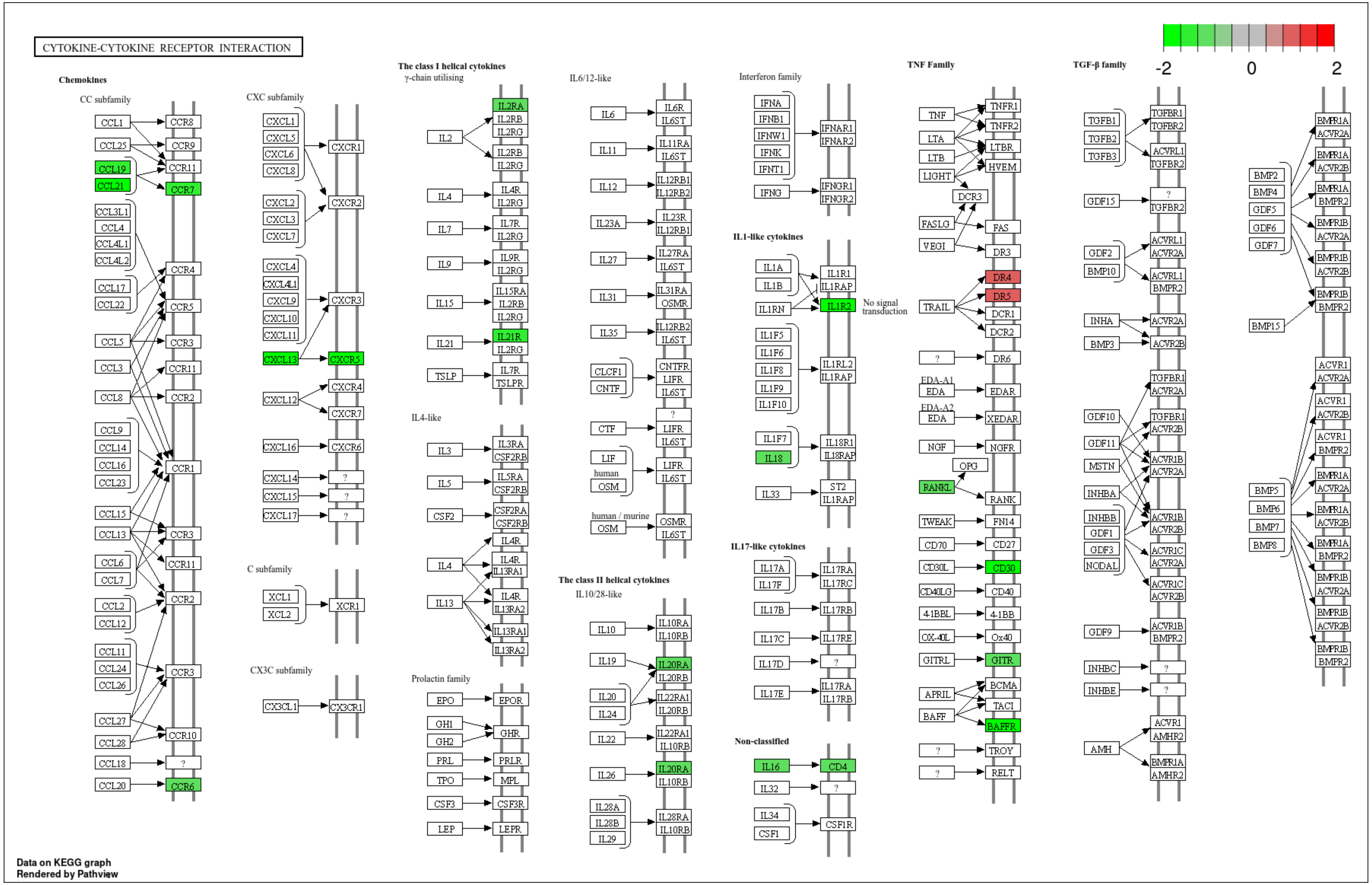

Figure S69. Cytokine – cytokine receptor interaction pathway in SYNCHs group in F3 (Cecal tonsils).

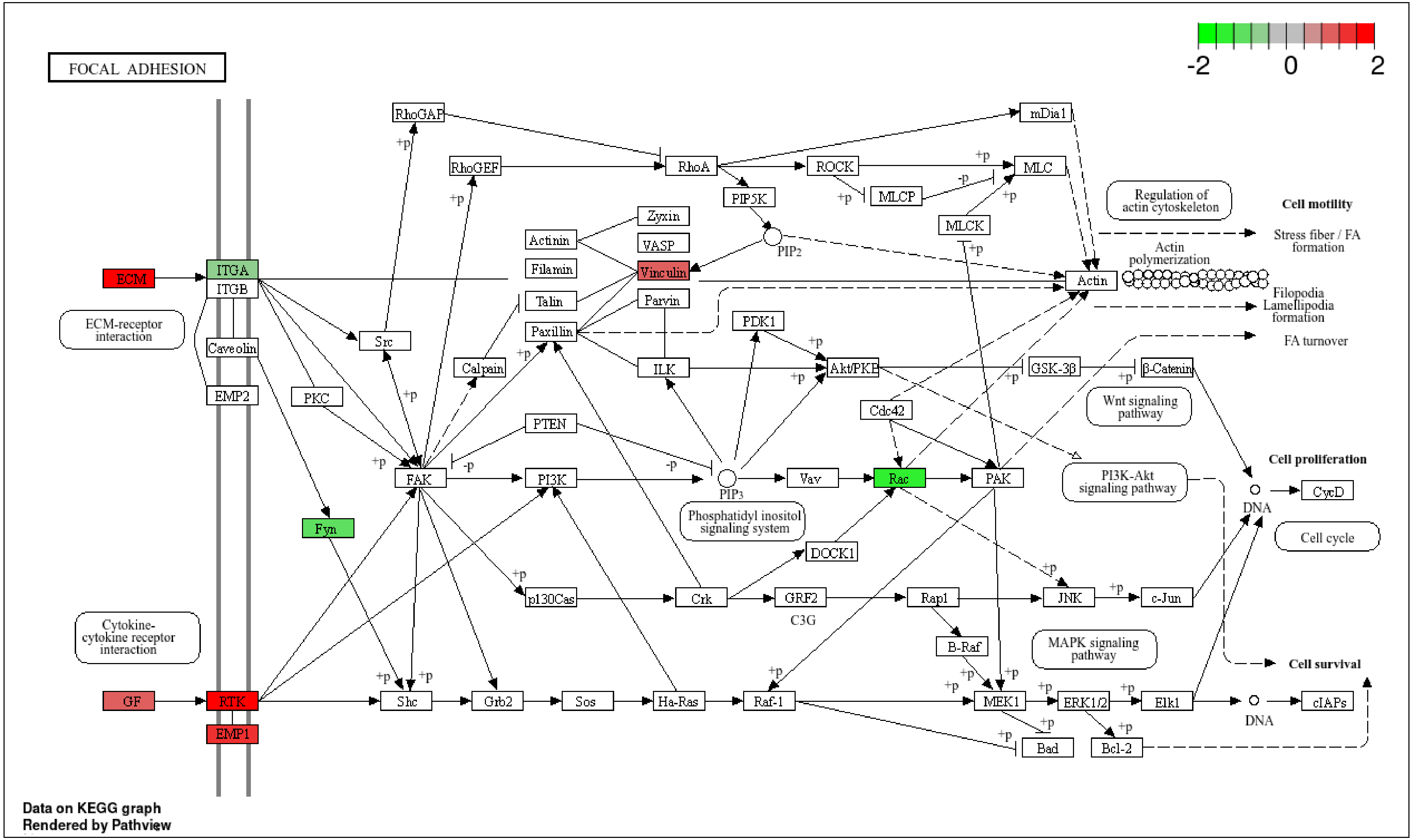

Figure S70. Focal adhesion pathway in SYNCHs group in F3 (Cecal tonsils).

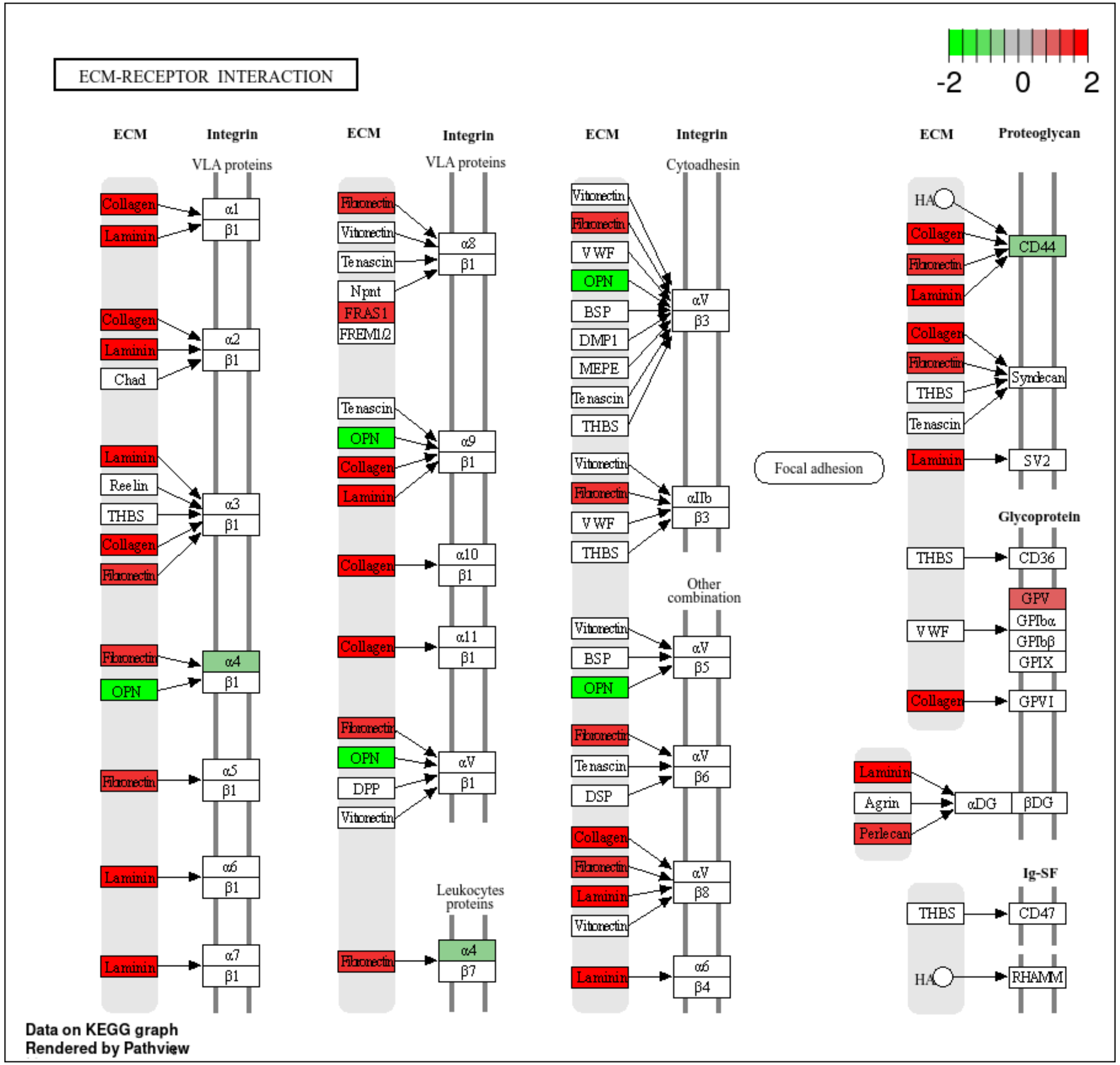

Figure S71. ECM-Receptor interaction pathway in SYNCHs group in F3 (Cecal tonsils).

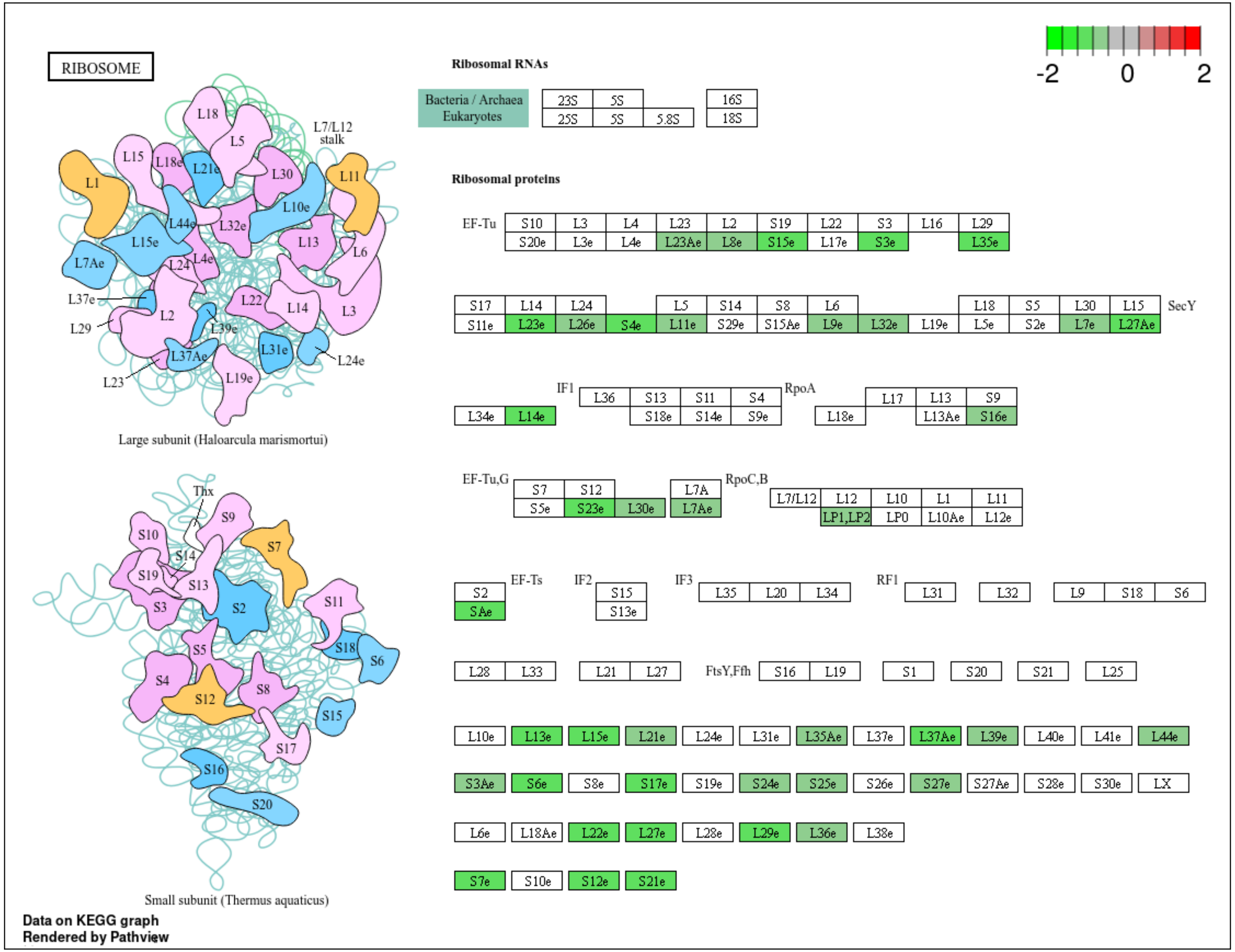

Figure S72. Ribosome pathway in SYNCHs group in F3 (Cecal tonsils).

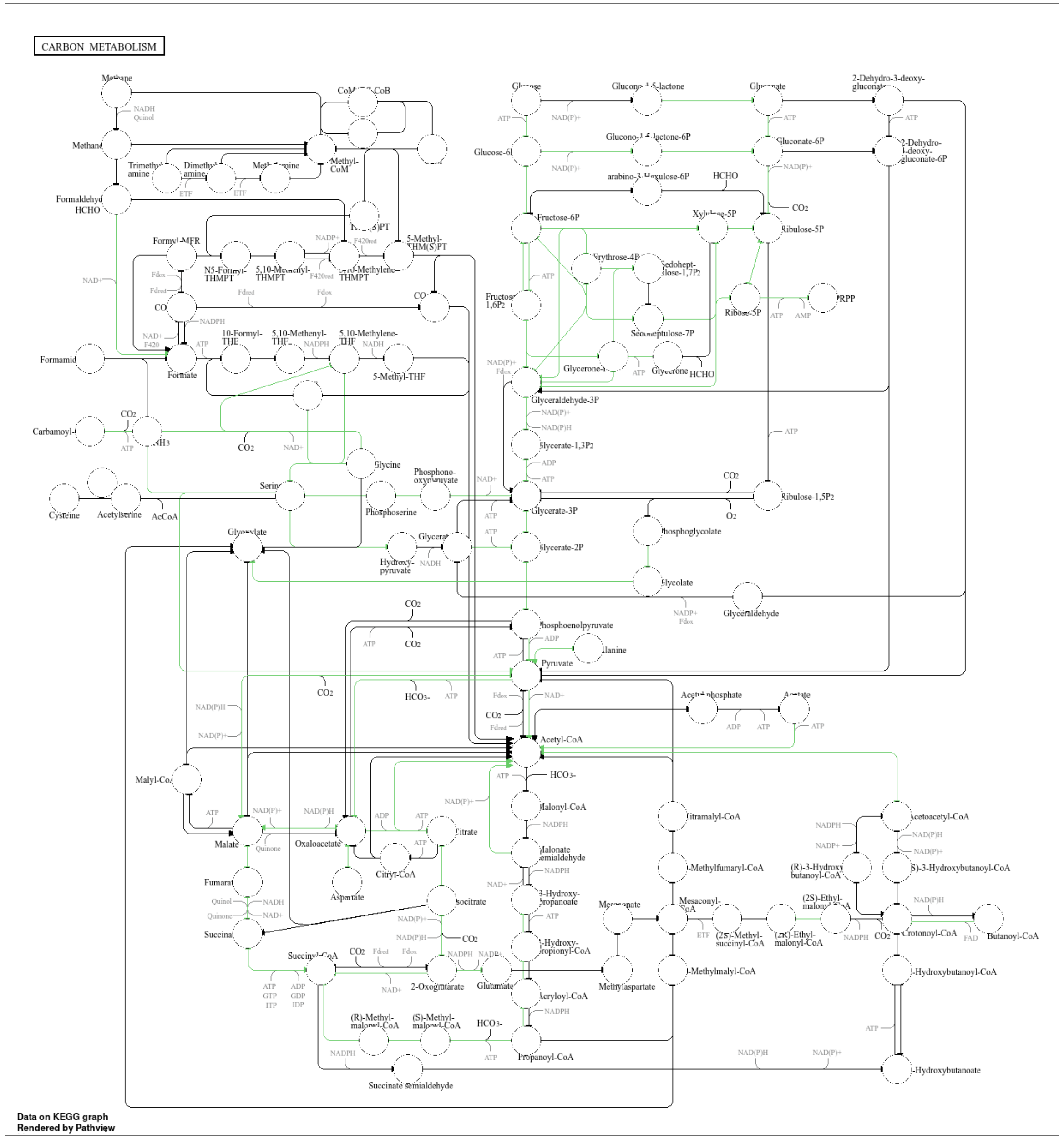

Figure S73. Carbon metabolism pathway in SYNCHr group in F3 (Cecal tonsils).

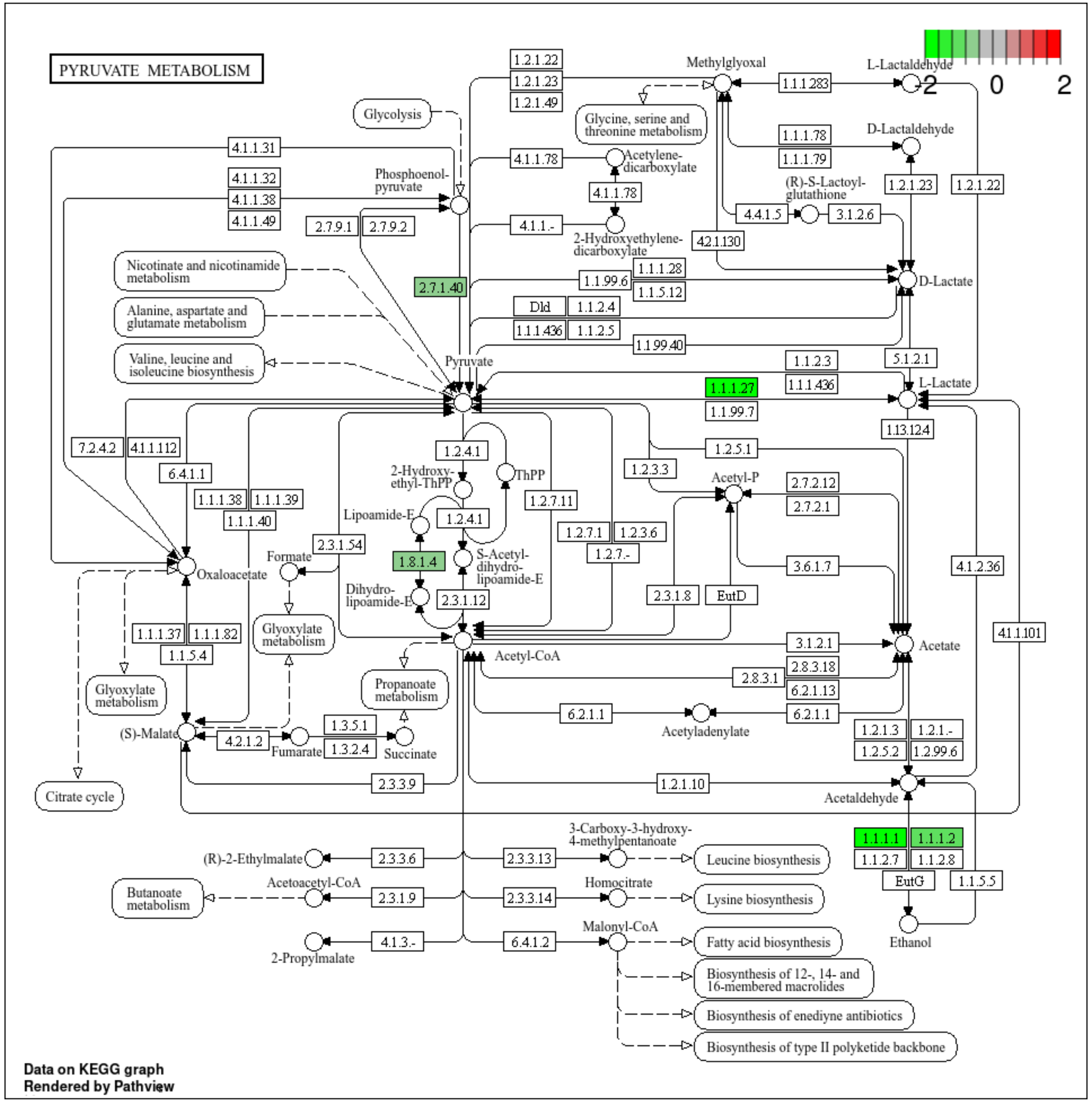

Figure S74. Pyruvate metabolism pathway in SYNCHr group in F3 (Cecal tonsils).

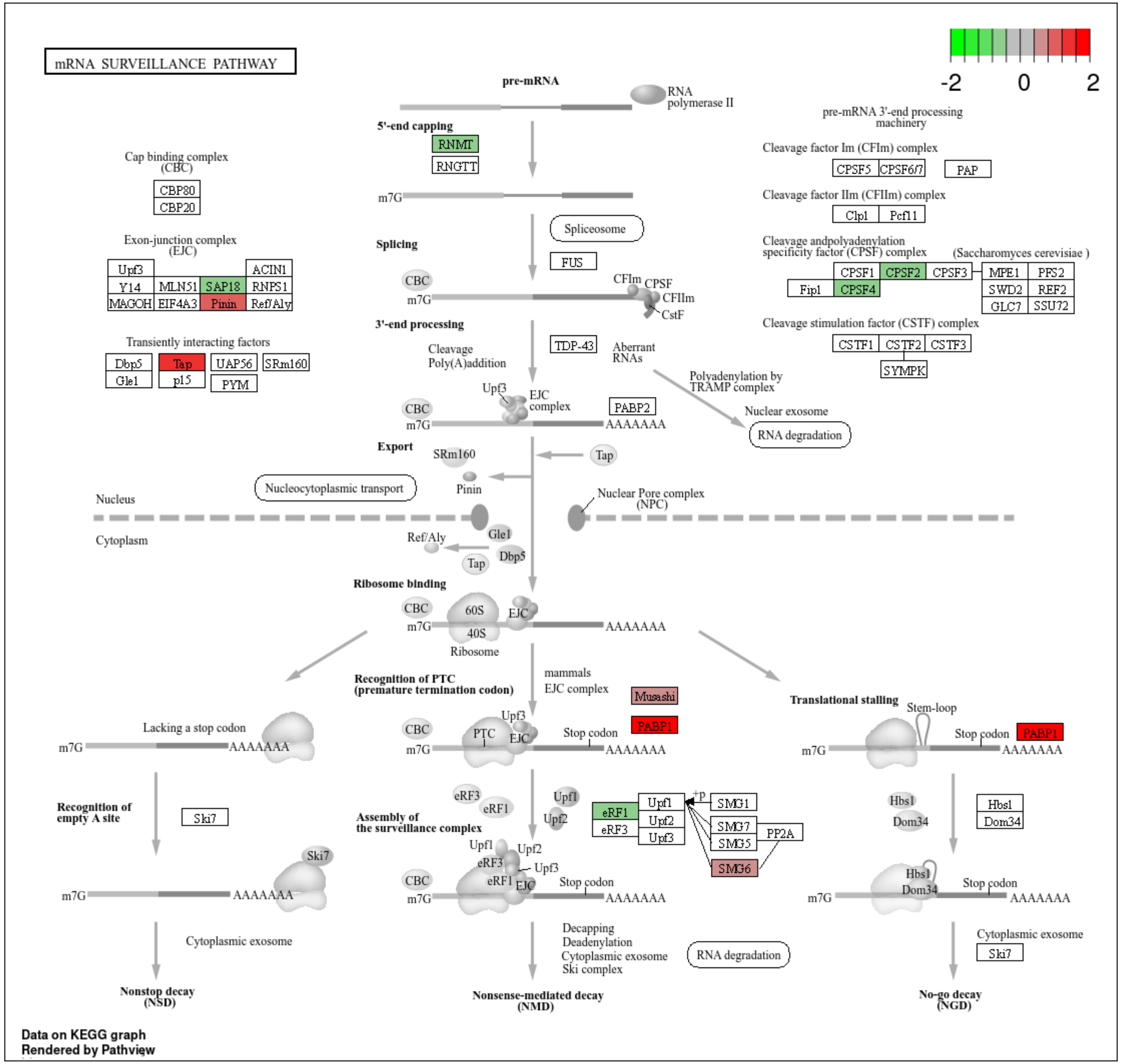

Figure S75. mRNA surveillance pathway in SYNCHr group in F3 (Cecal tonsils).

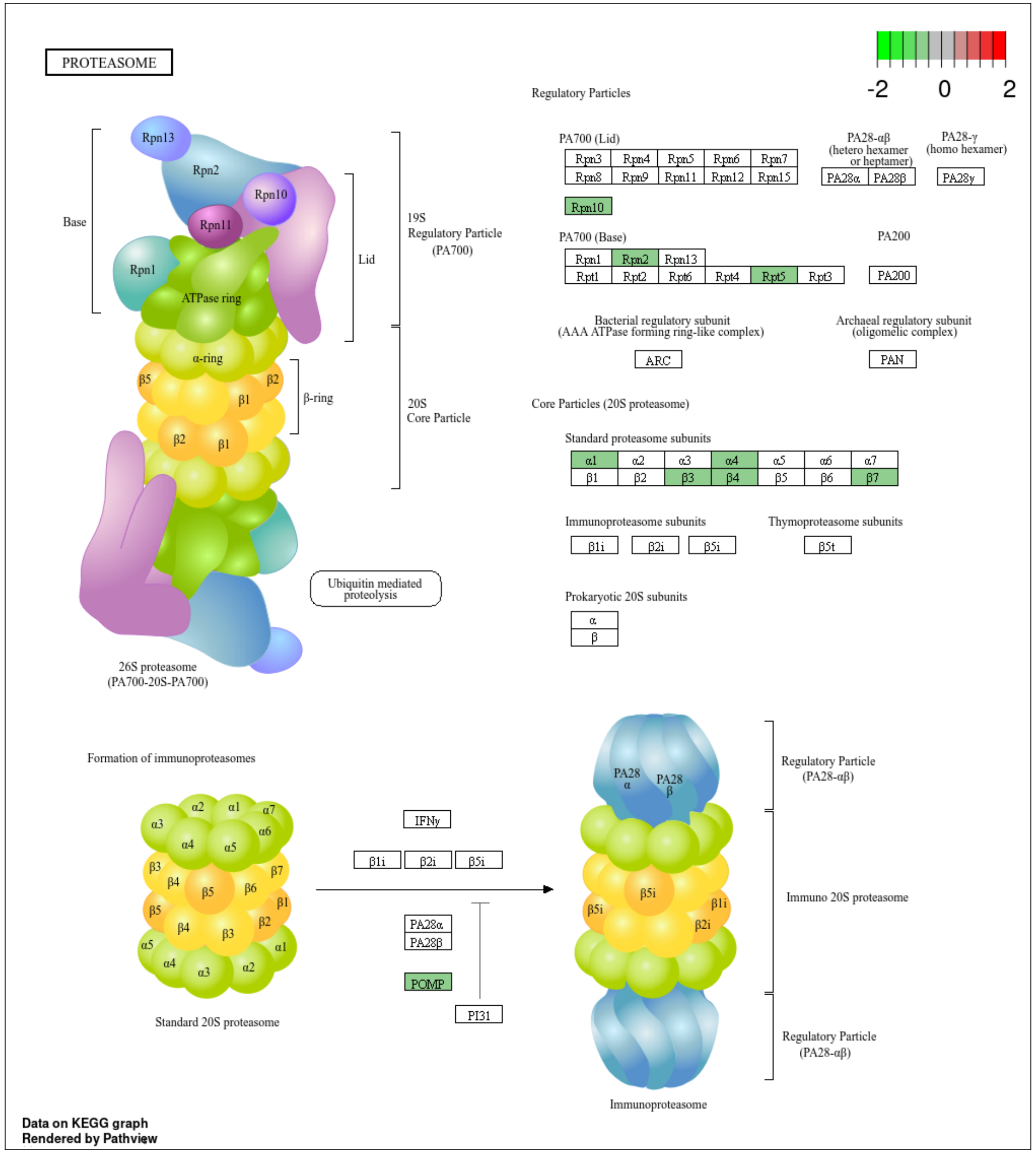

Figure S76. Proteasome pathway in SYNCHr group in F3 (Cecal tonsils).

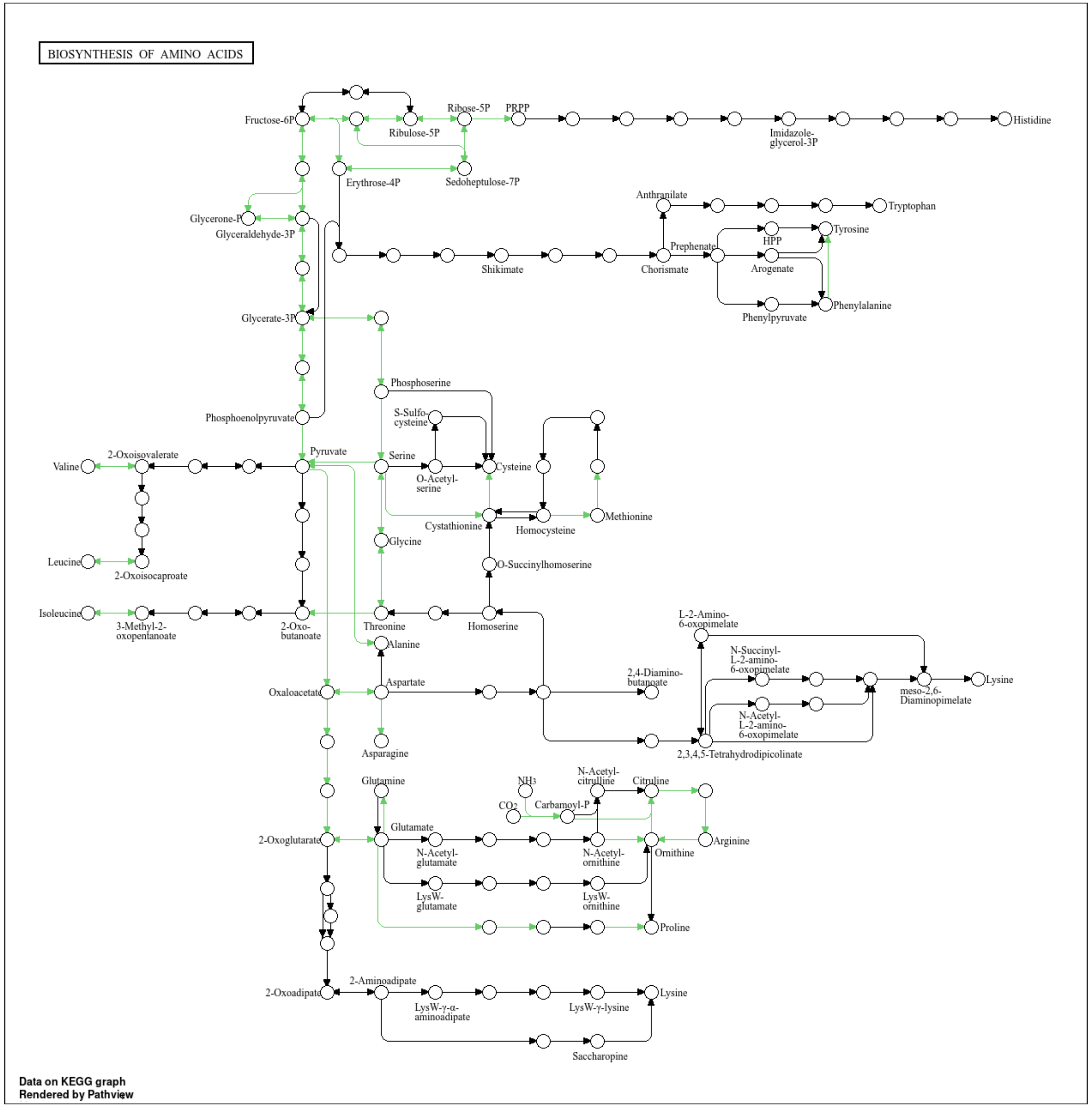

Figure S77. Biosynthesis of amino acids pathway in SYNCHr group in F3 (Cecal tonsils).

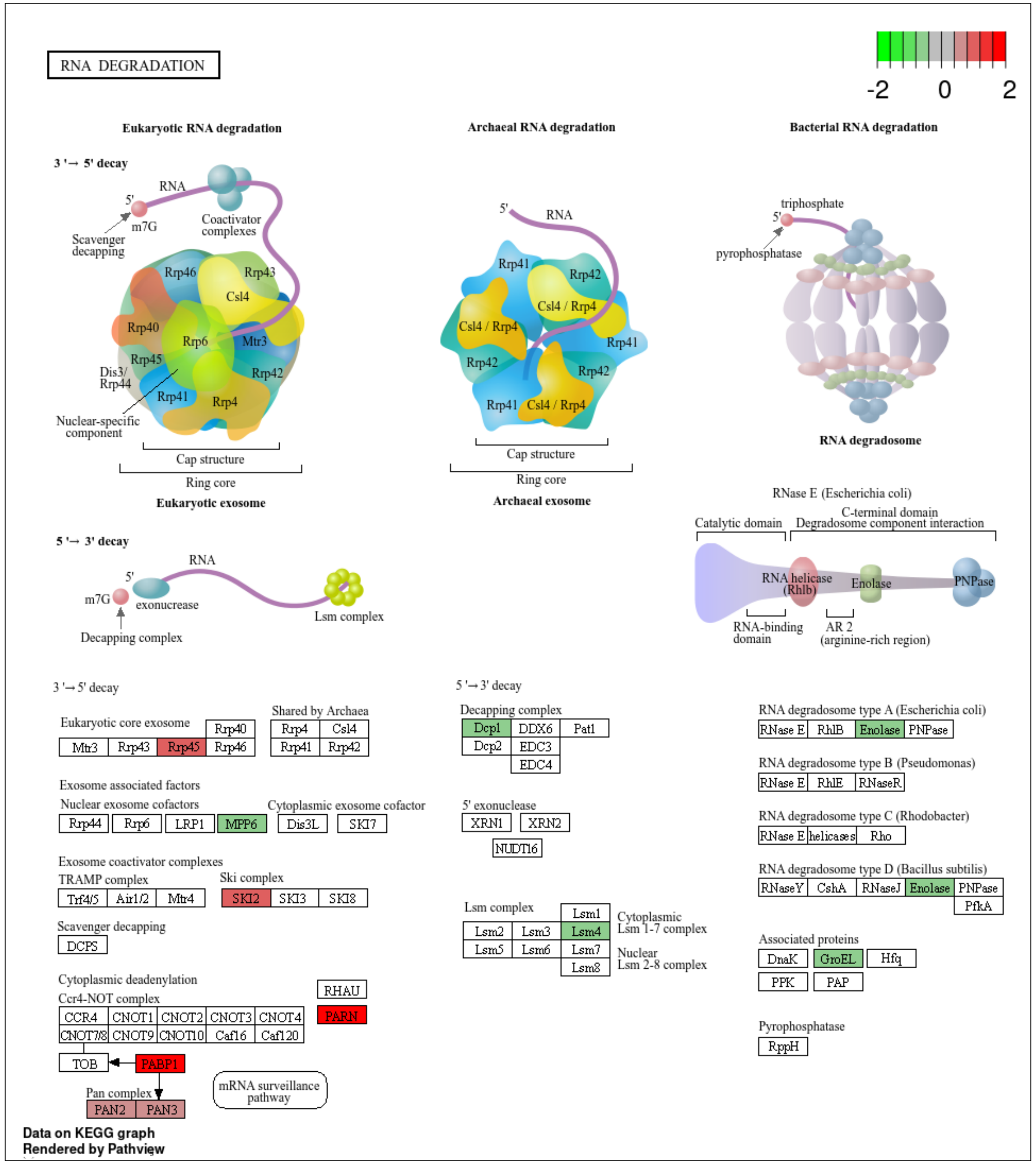

Figure S78. RNA degradation pathway in SYNCHr group in F3 (Cecal tonsils).

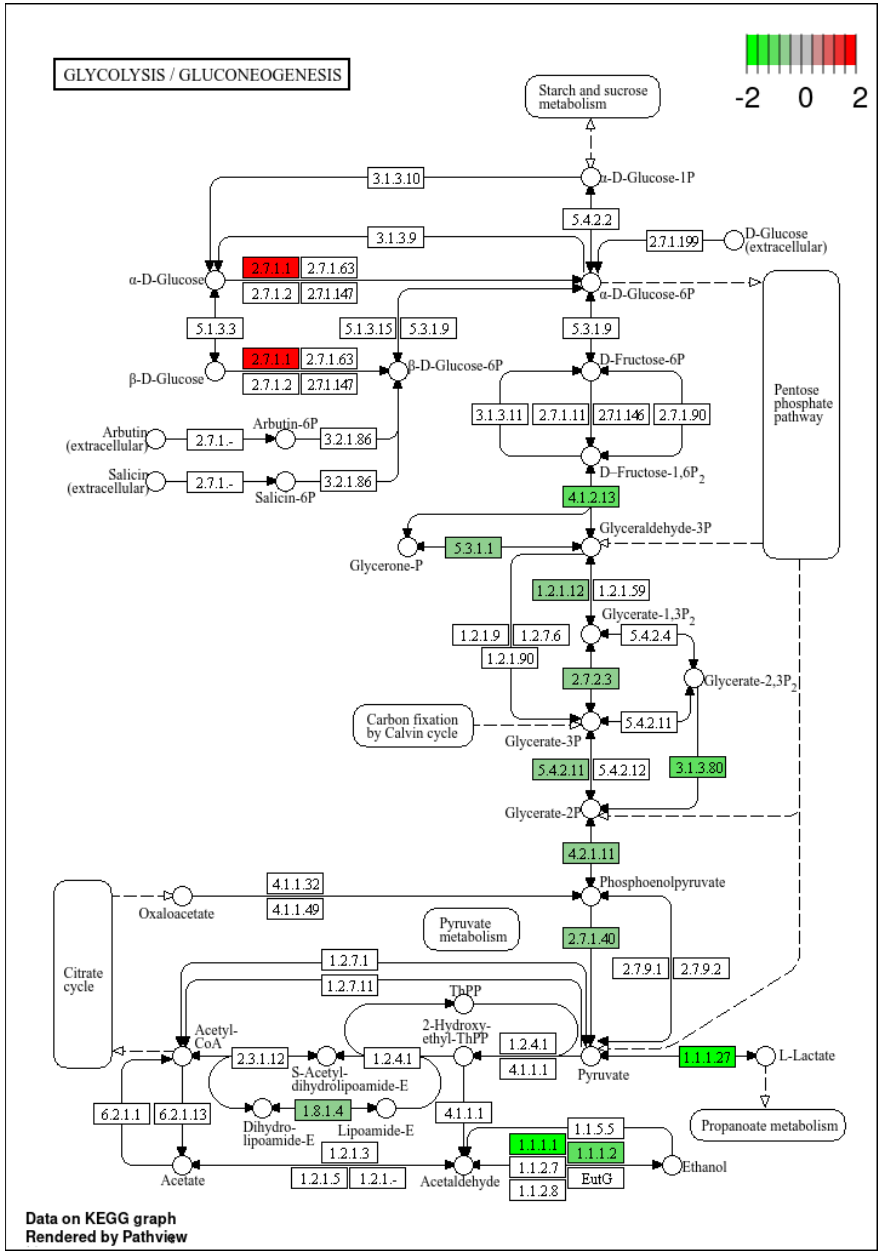

Figure S79. Glycolysis / gluconeogenesis pathway in SYNCHr group in F3 (Cecal tonsils).

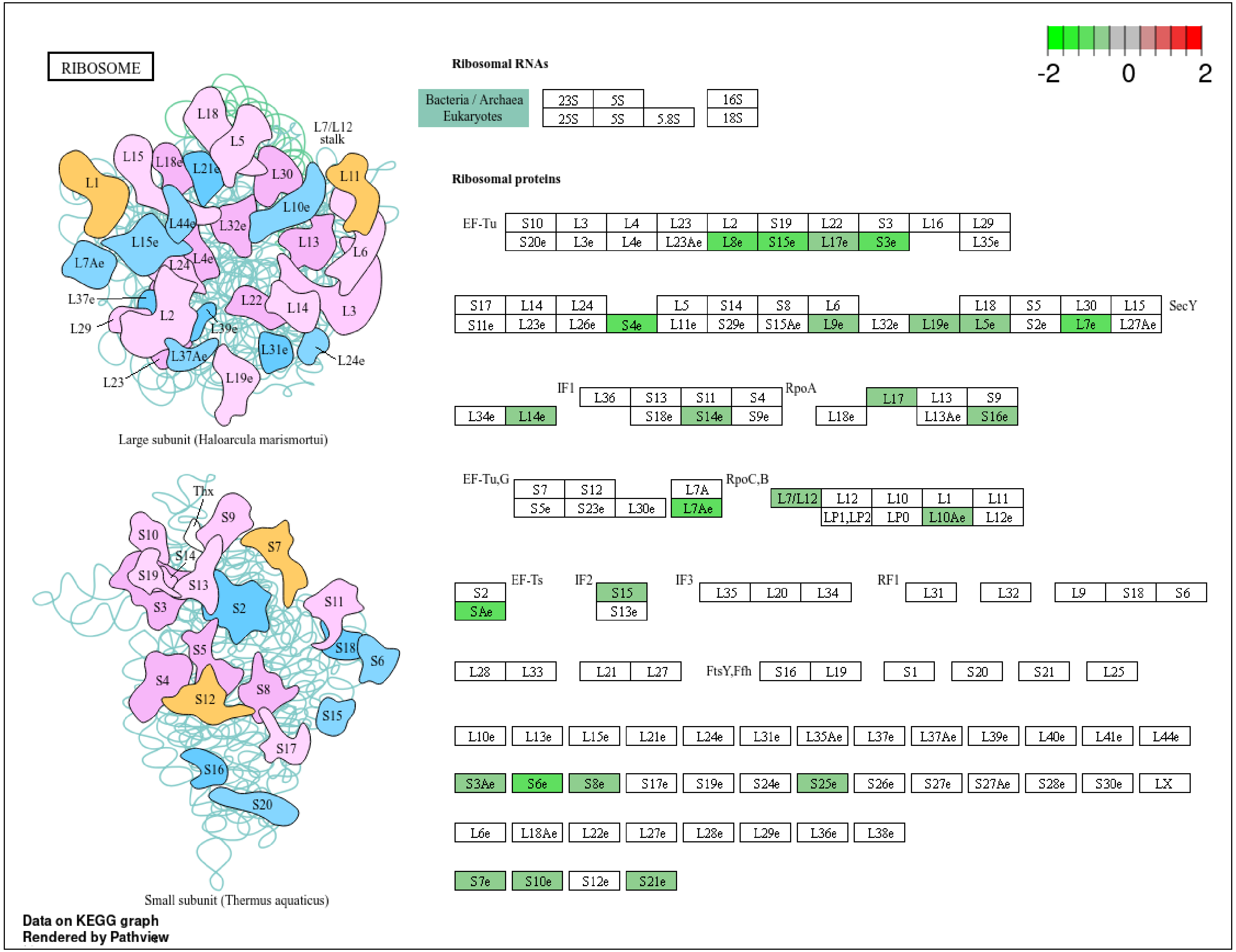

Figure S80. Ribosome pathway in SYNCHr group in F3 (Cecal tonsils).
