# Supplementary material for: Inter- and Transgenerational Effects of In Ovo Stimulation with Bioactive Compounds on Cecal Tonsils and Cecal Mucosa Transcriptomes in a Chicken Model
Source: Int J Mol Sci. 2025 Jan 29;26(3):1174. doi: 10.3390/ijms26031174 (PMC11817890; doi:10.3390/ijms26031174)
Supplement: Supplementary file 1 [file ijms-26-01174-s001.zip › Supplementary file S15.pdf]

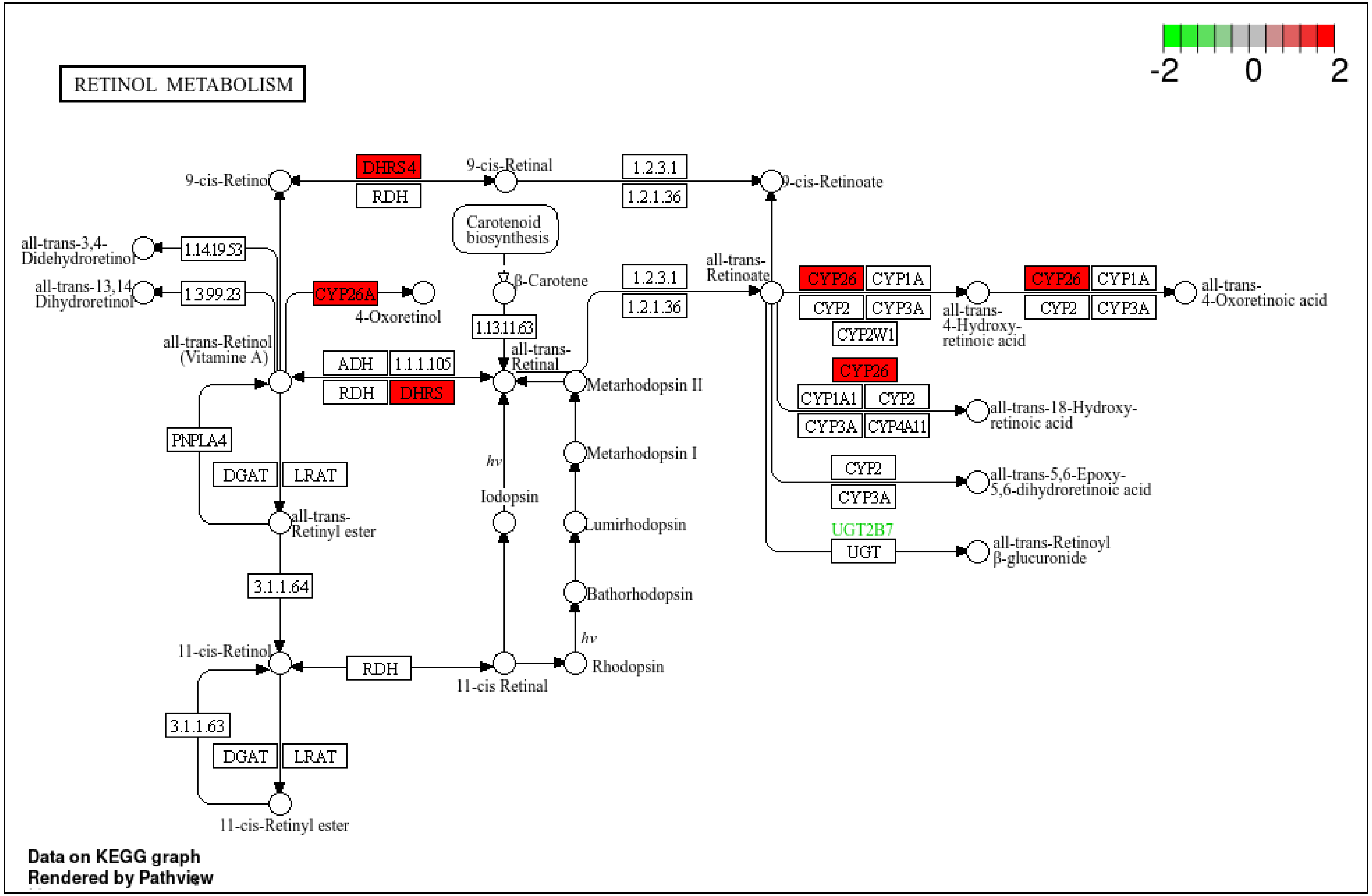

Figure S1. Retinol metabolism pathway in SYN group in F1 (Cecal mucosa).

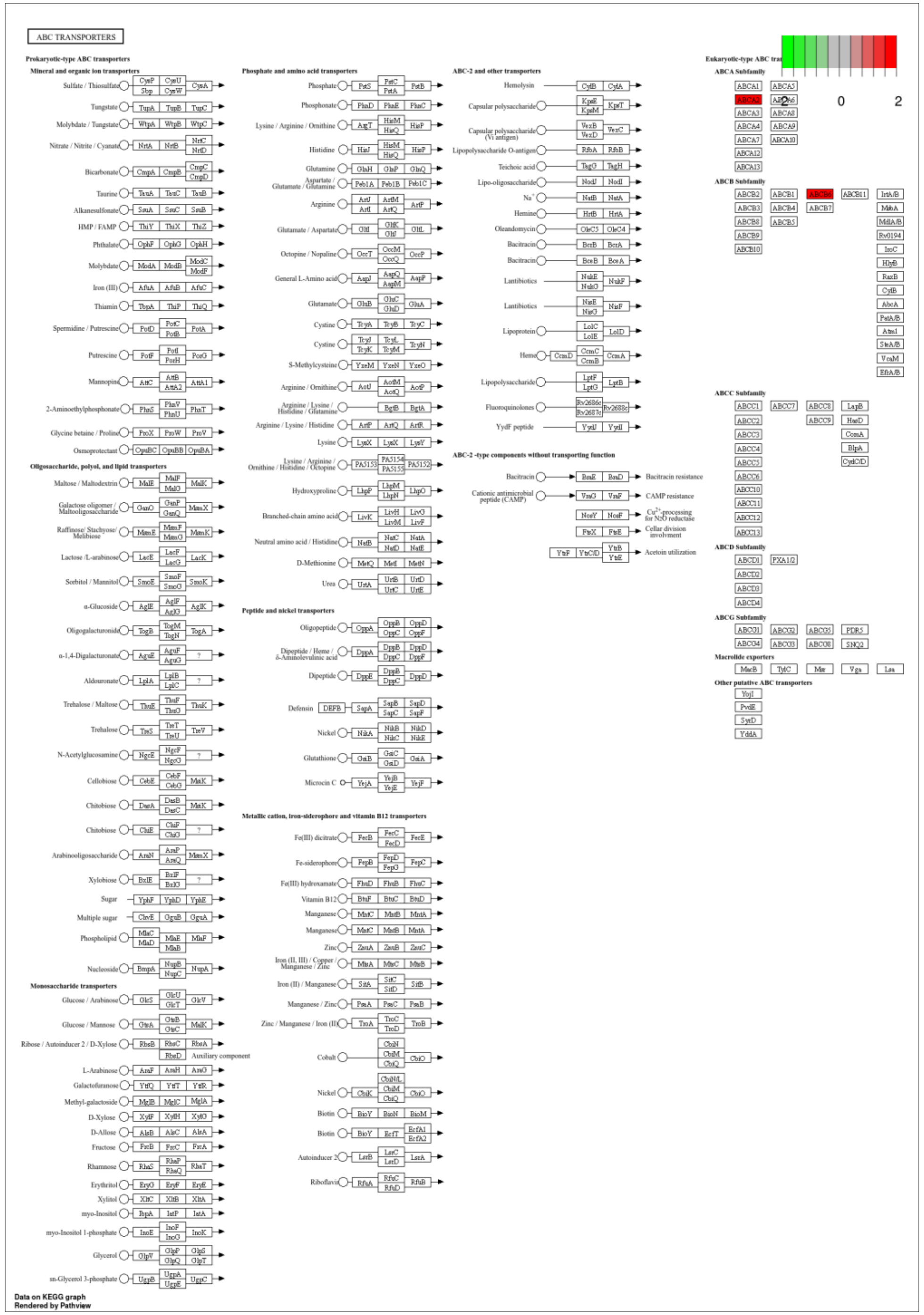

Figure S2. ABC transporter pathway in SYN group in F1 (Cecal mucosa)

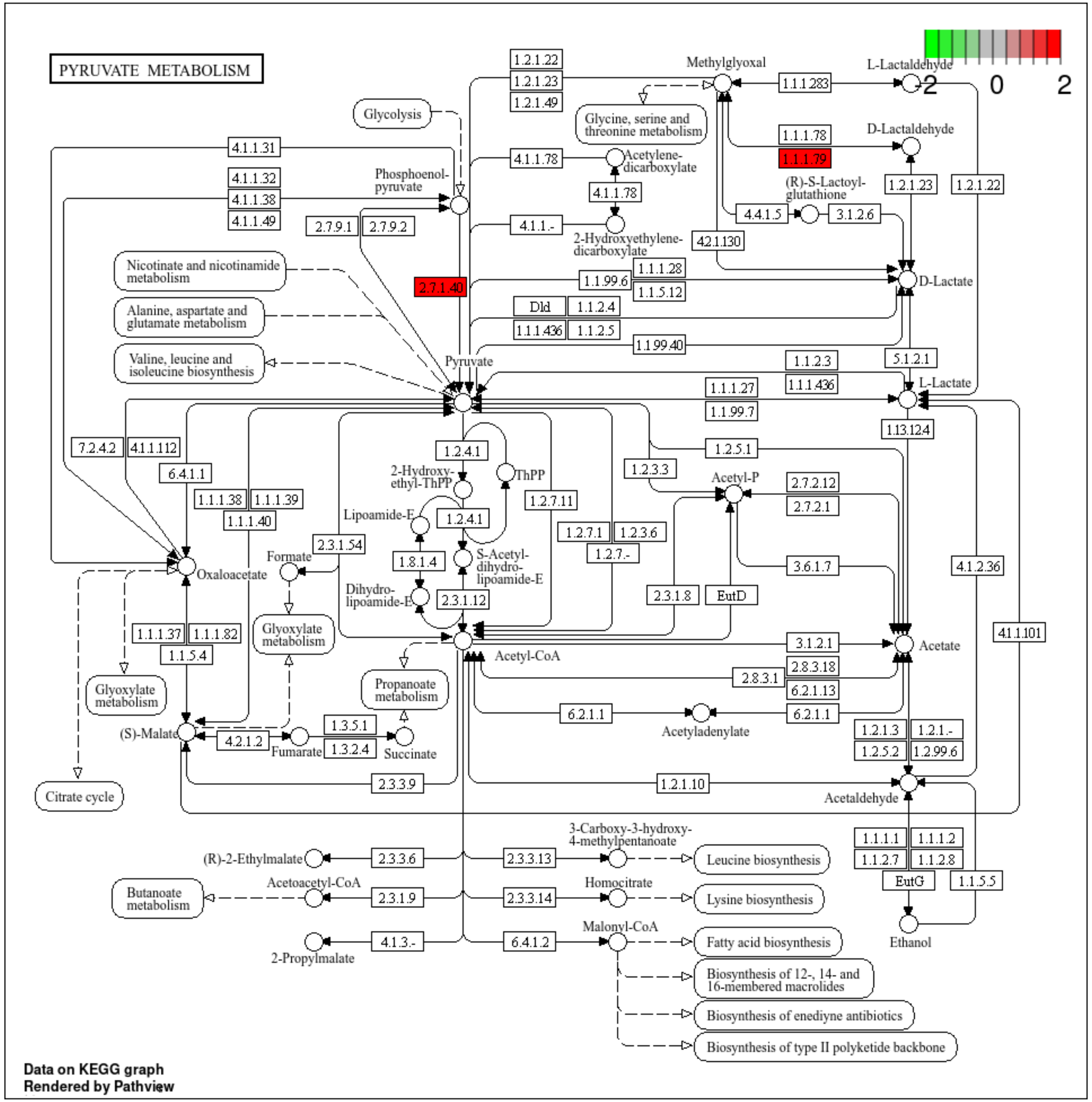

Figure S3. Pyruvate metabolism pathway in SYN group in F1 (Cecal mucosa).

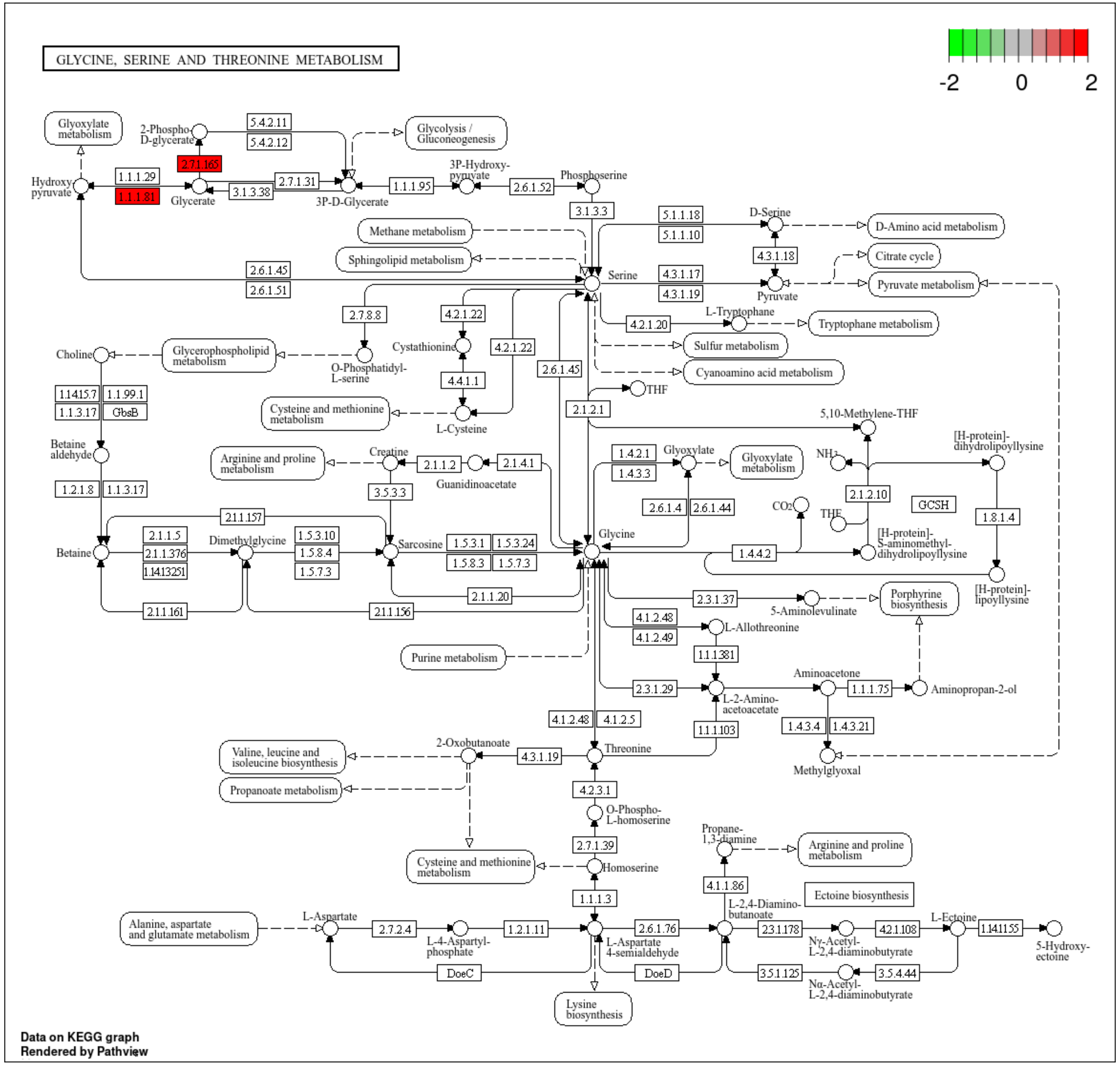

Figure S4. Glycine, serine and threonine metabolism pathway in SYN group in F1 (Cecal mucosa).

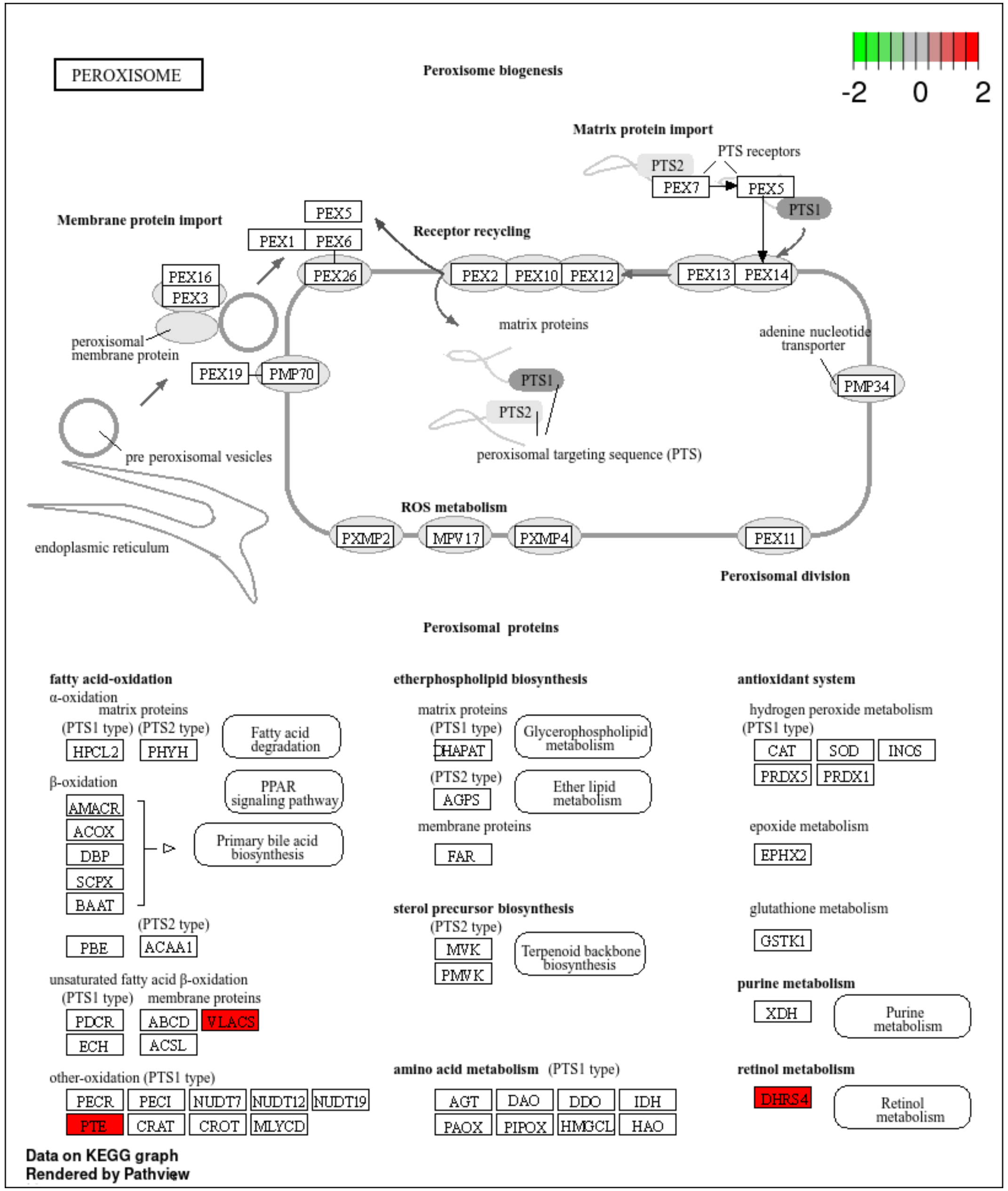

Figure S5. Peroxisome pathway in SYN group in F1 (Cecal mucosa).

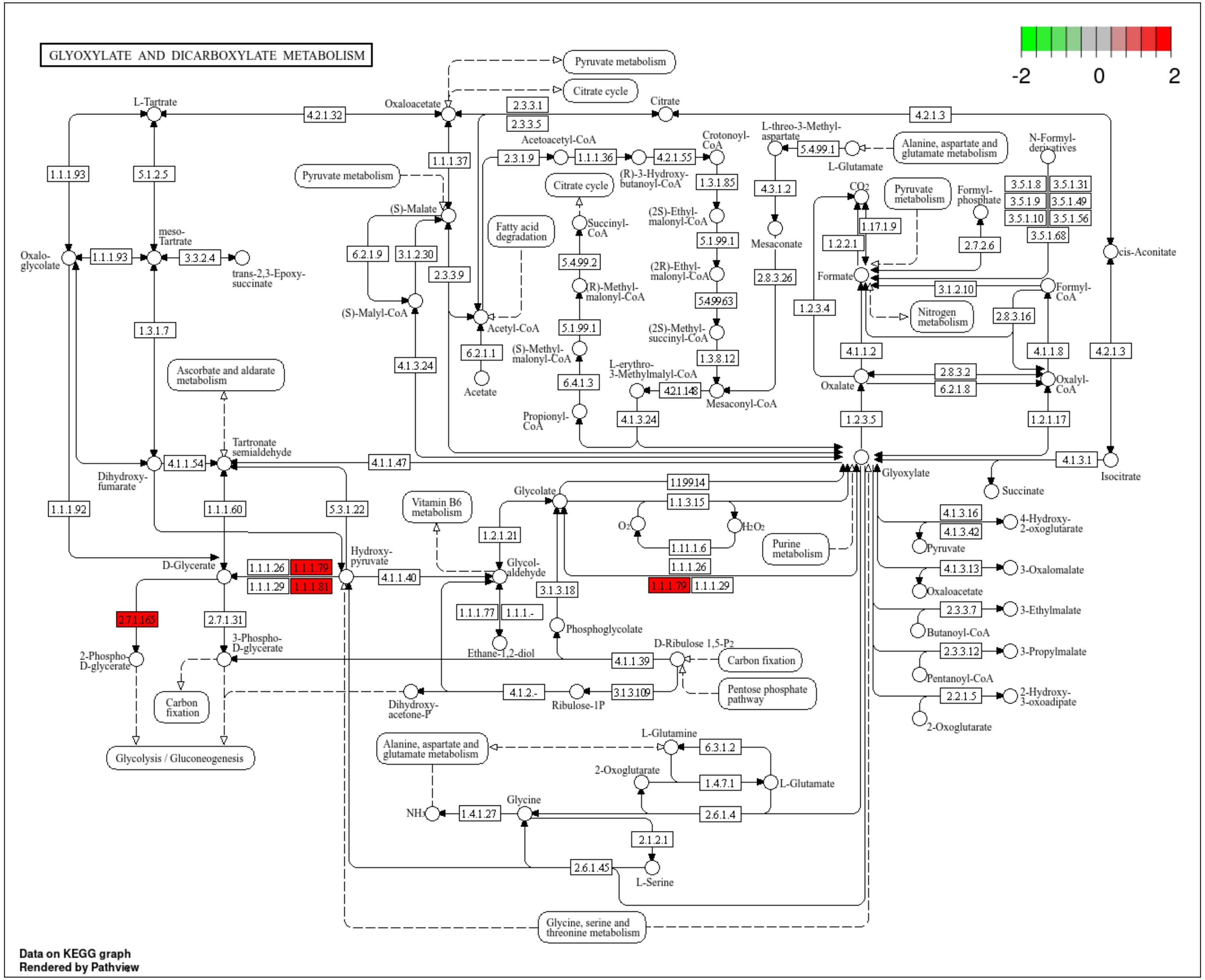

Figure S6. Glyoxylate and carboxylate pathway in SYN group in F1 (Cecal mucosa).

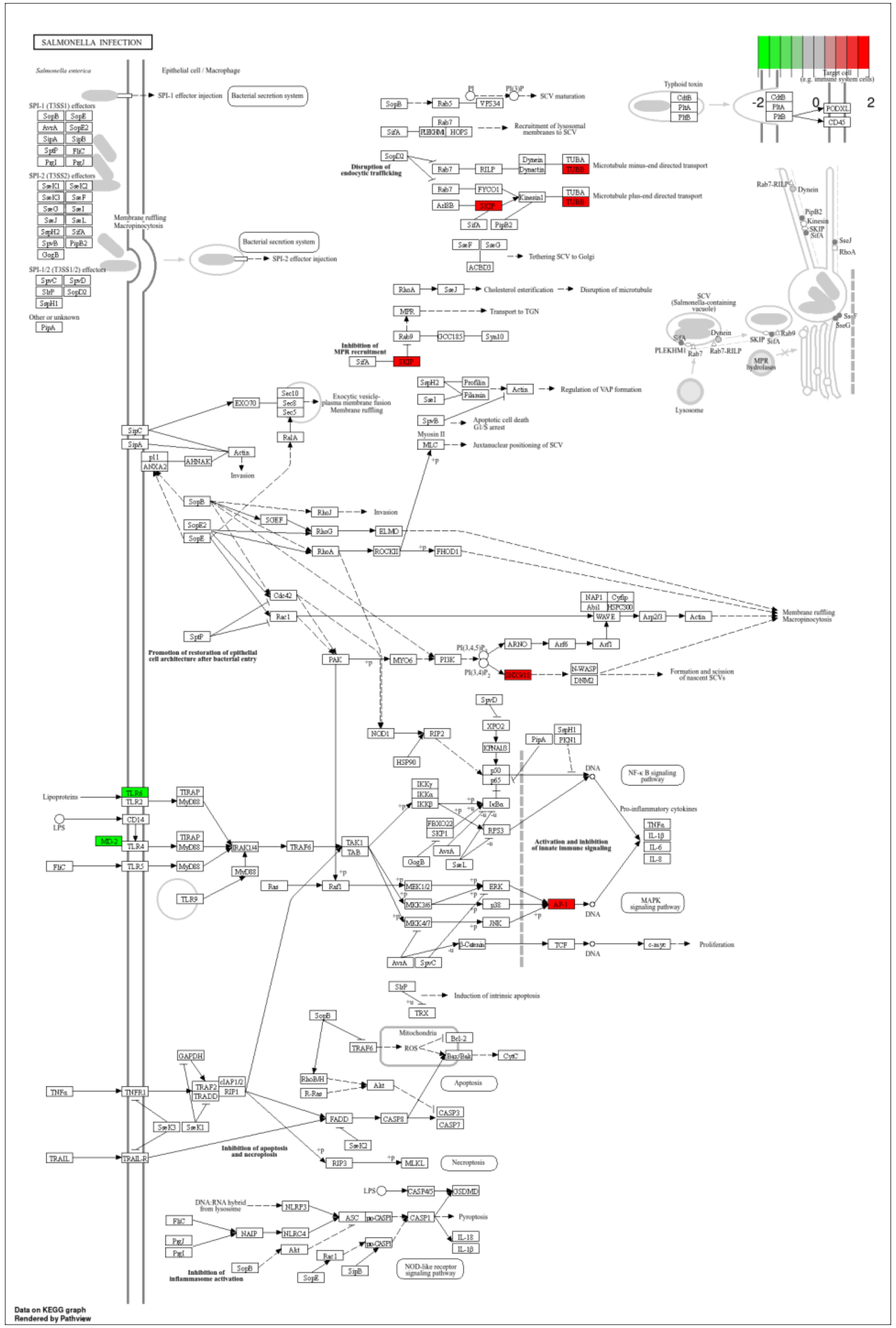

Figure S7. Salmonella infection pathway in SYN group in F1 (Cecal mucosa).

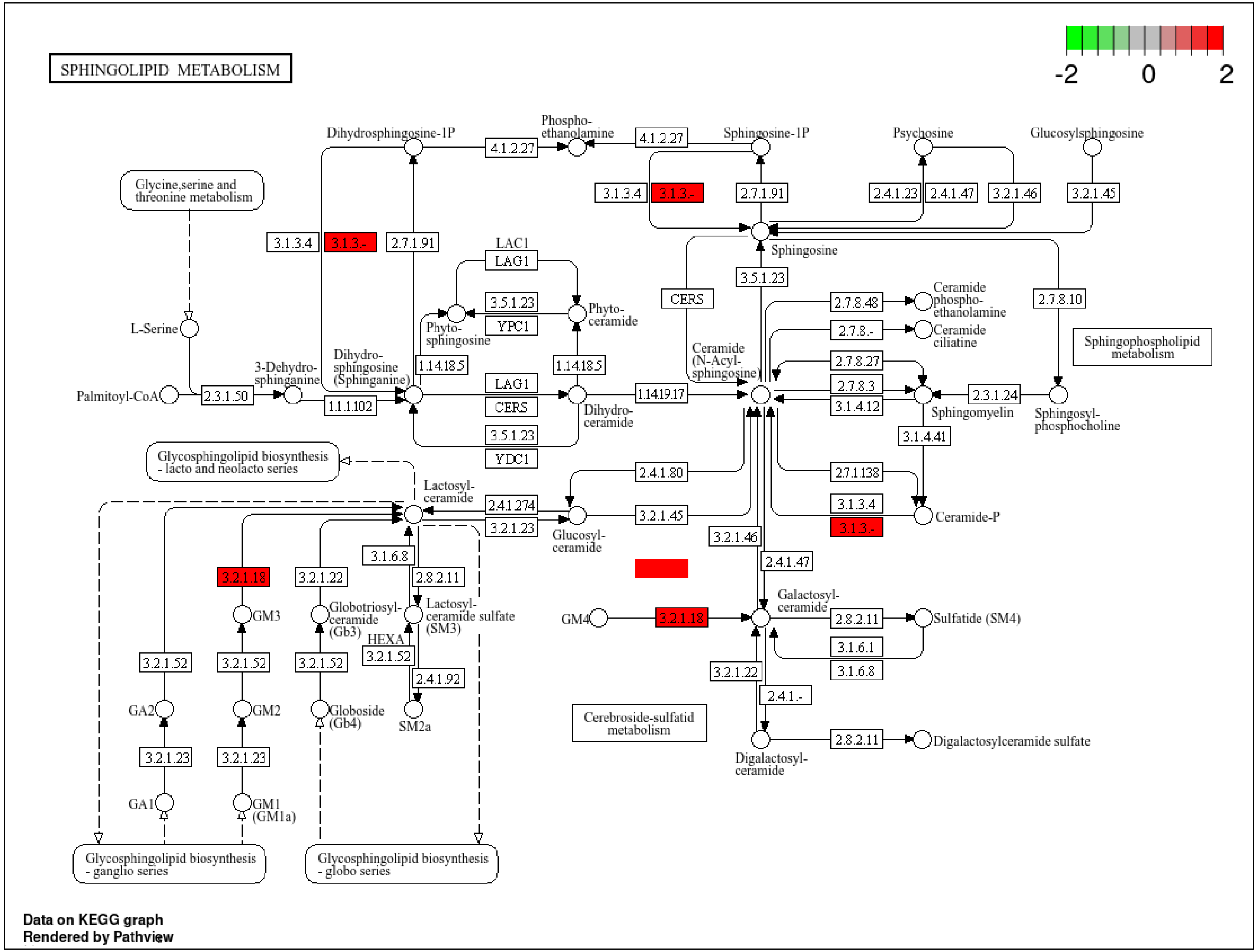

Figure S8. Sphingolipid metabolism pathway in SYN group in F1 (Cecal mucosa).

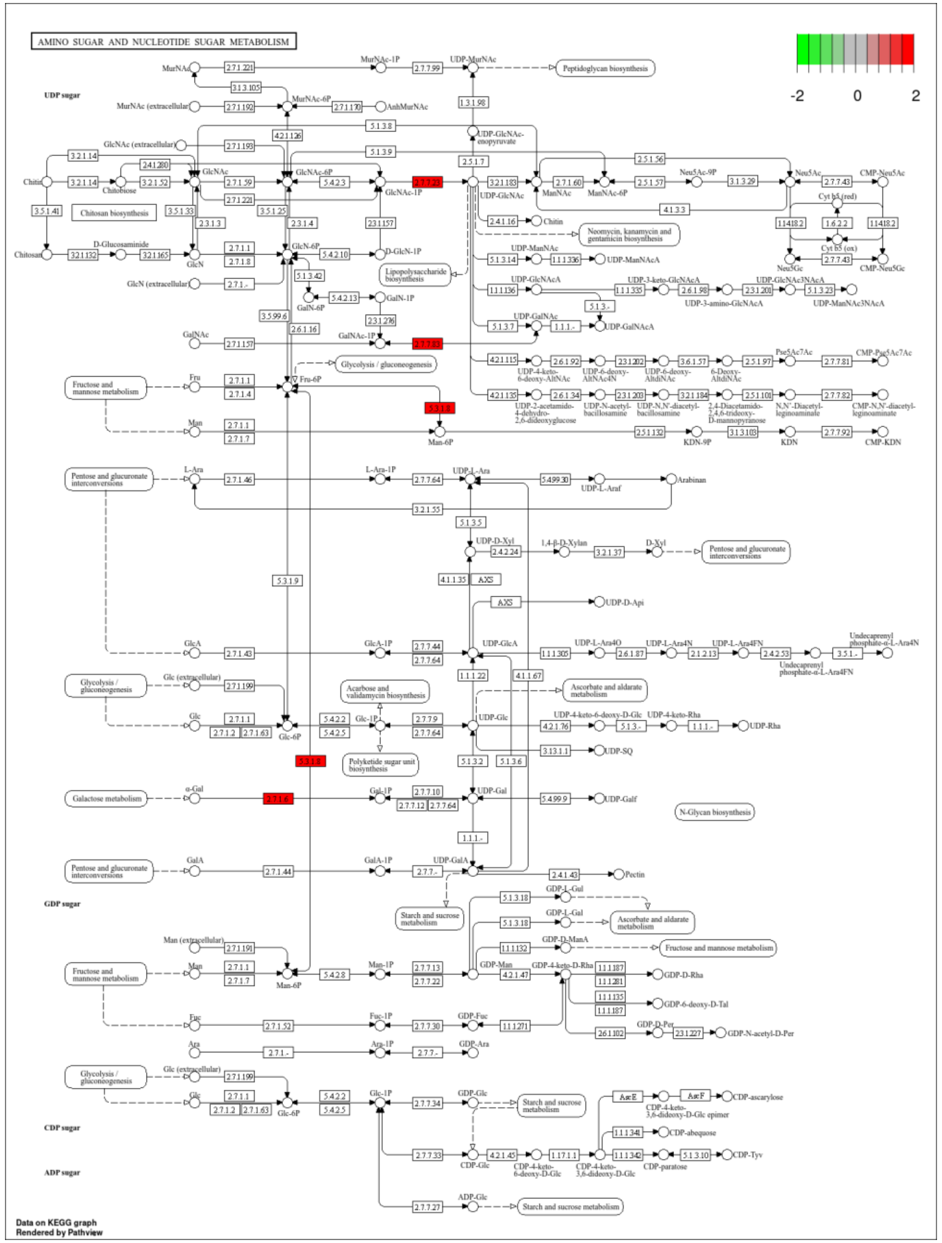

Figure S9. Amino sugar and nucleotide sugar metabolism pathway in SYN group in F1 (Cecal mucosa).

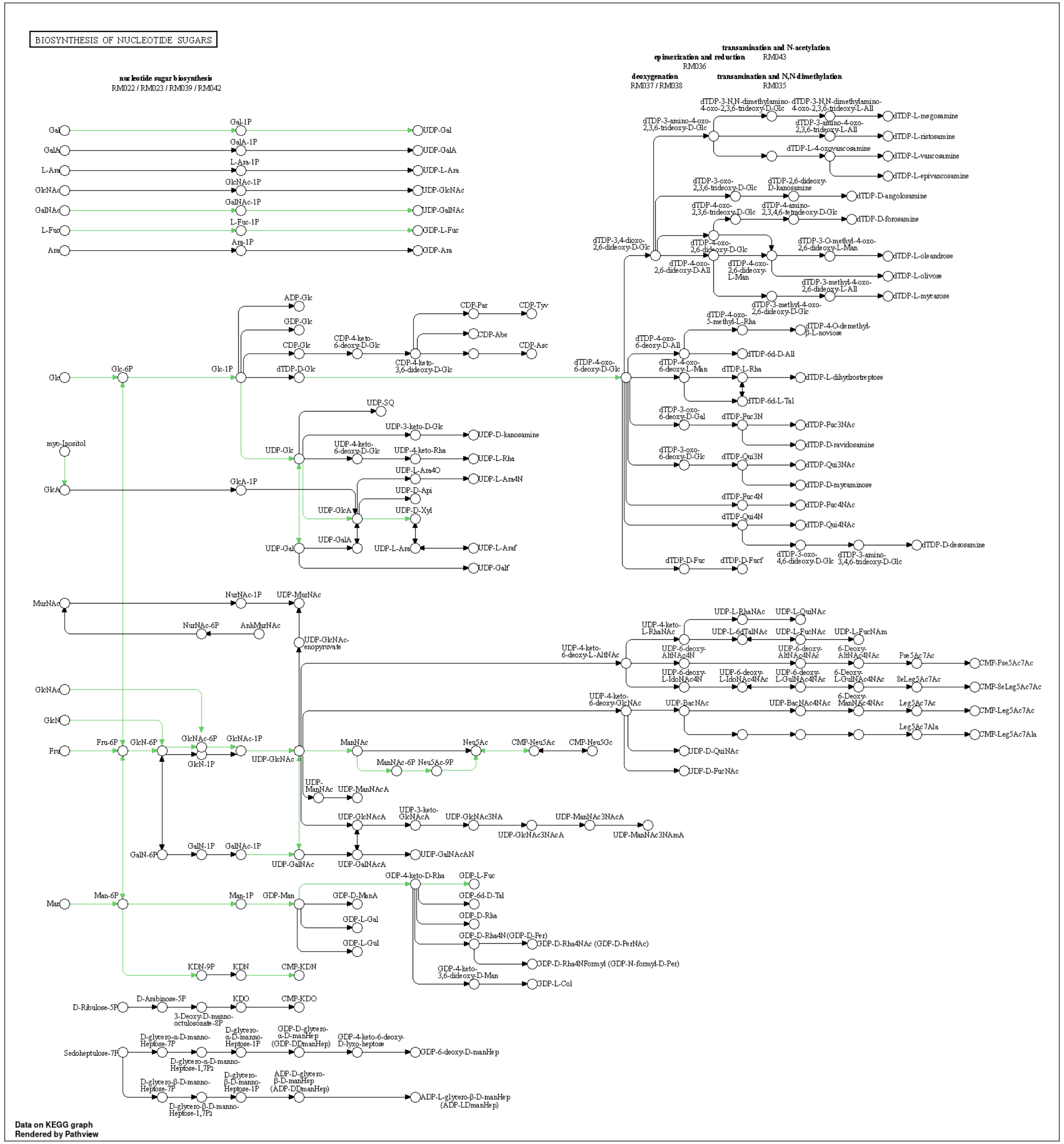

Figure S10. Biosynthesis and nucleotide sugars pathway in SYN group in F1 (Cecal mucosa).

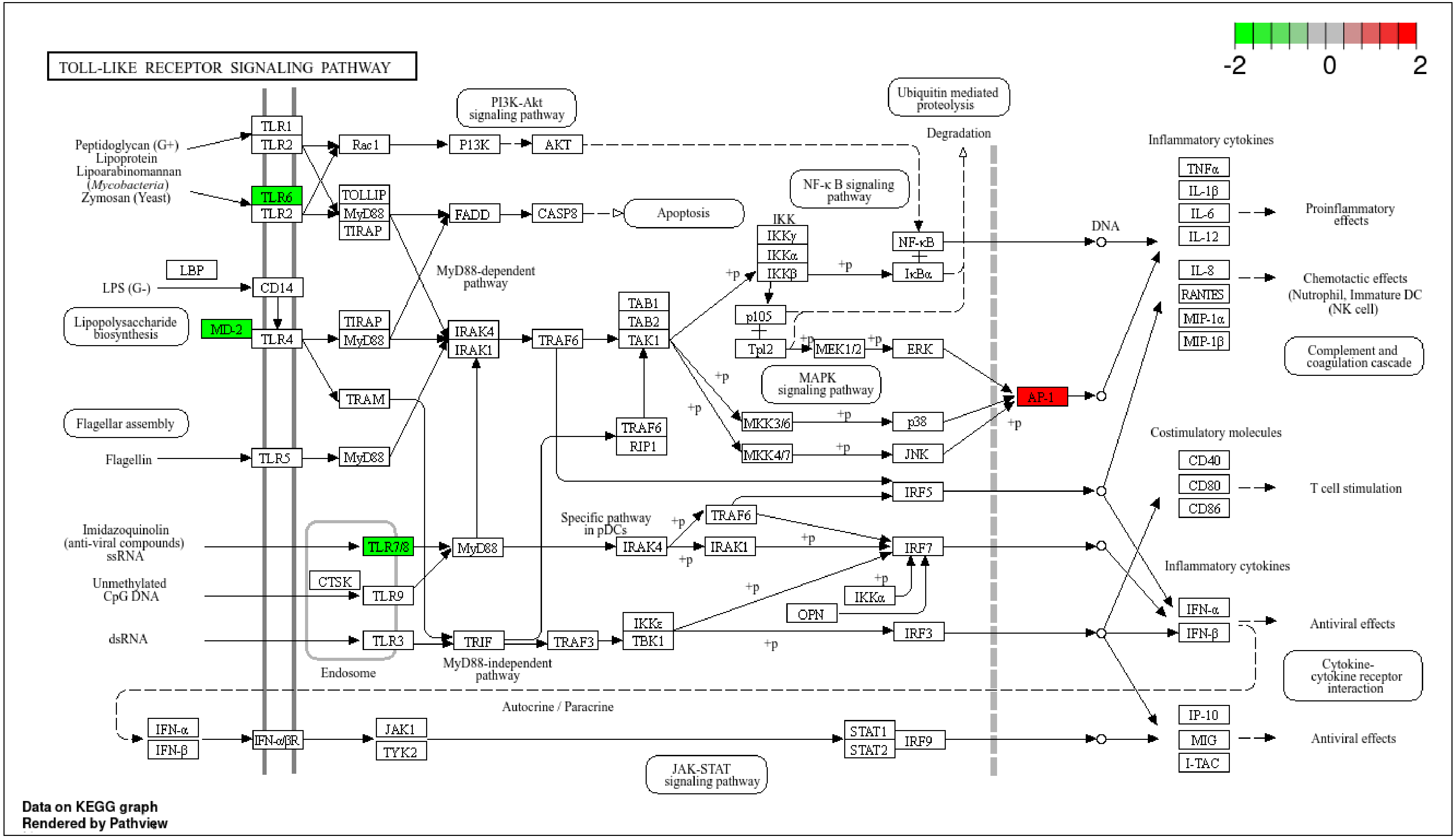

Figure S11. TOLL-like receptor signaling pathway in SYN group in F1 (Cecal mucosa).

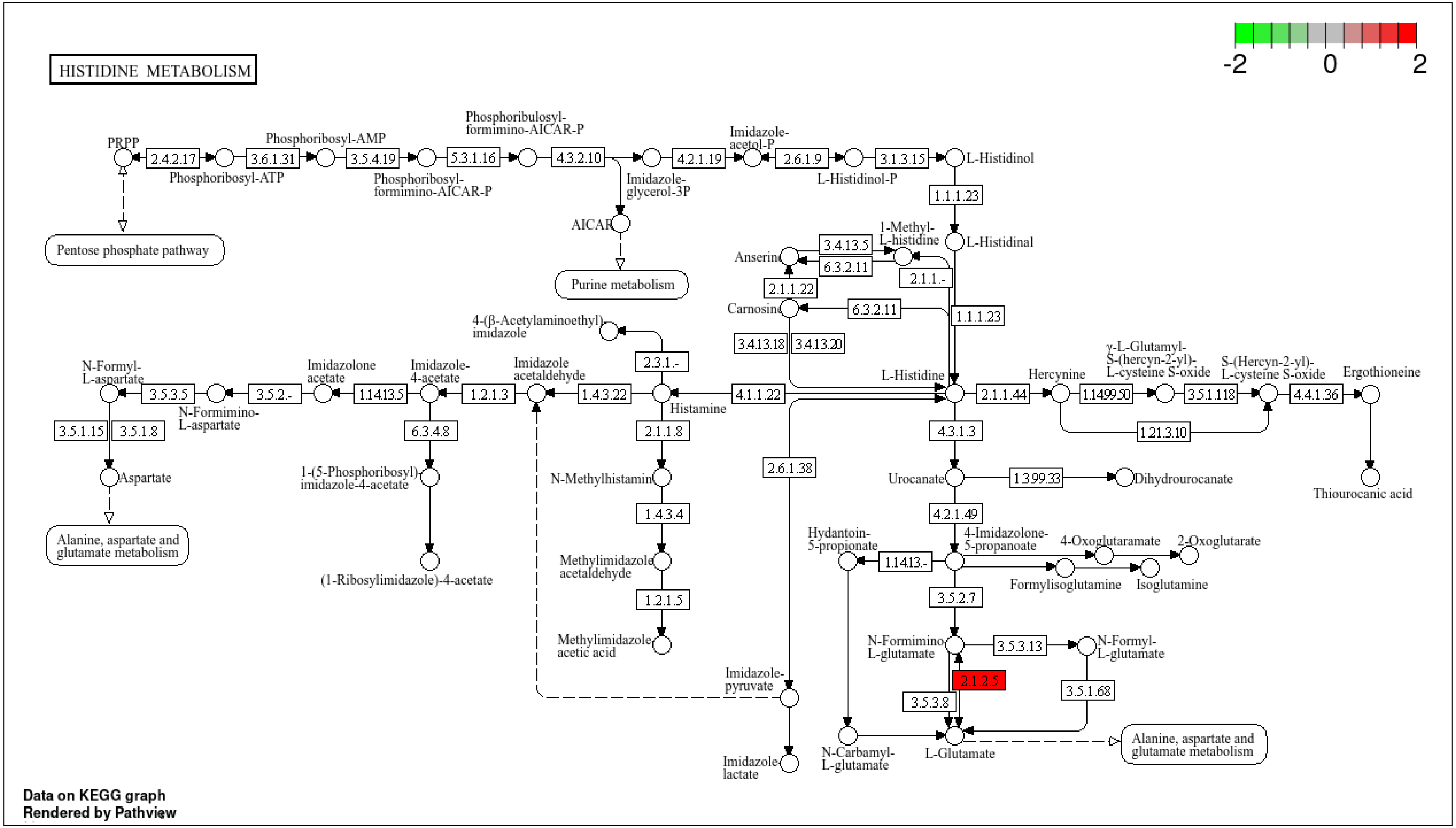

Figure S12. Histidine metabolism pathway in SYNCH group in F1 (Cecal mucosa).

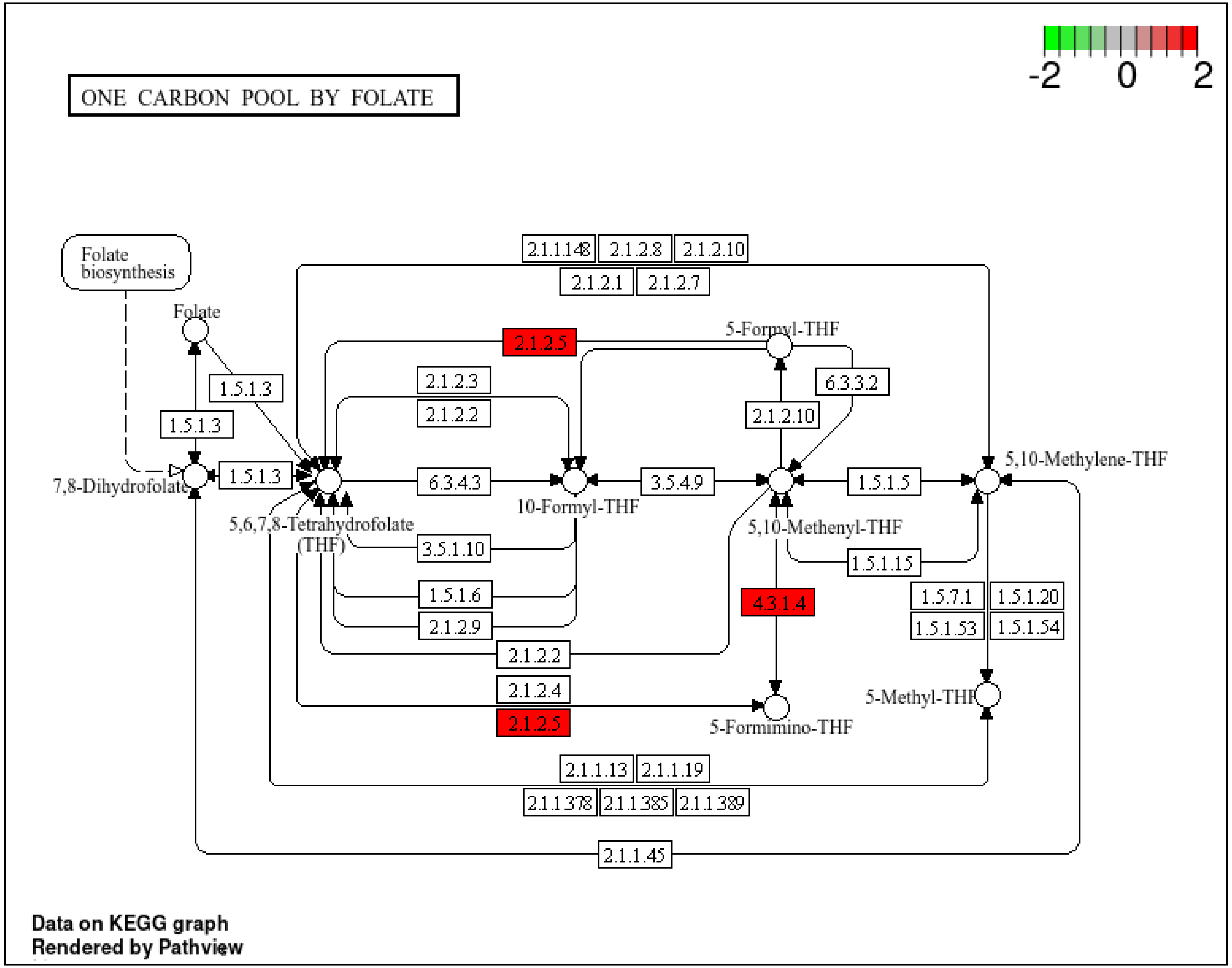

Figure S13. One Carbon pool by folate pathway in SYNCH group in F1 (Cecal mucosa).

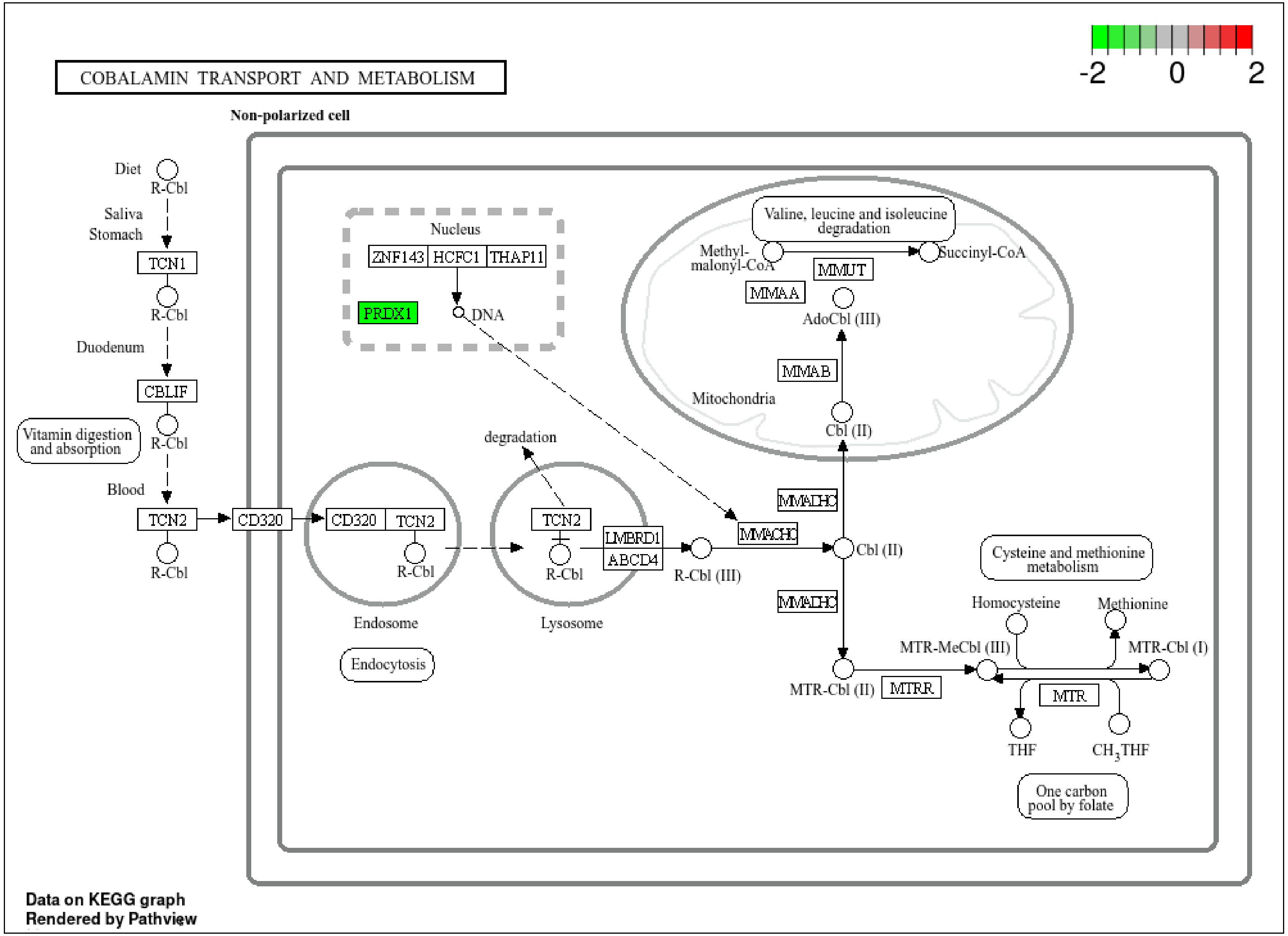

Figure S14. Cobalamin transport and metabolism pathway in SYNCH group in F1 (Cecal mucosa).

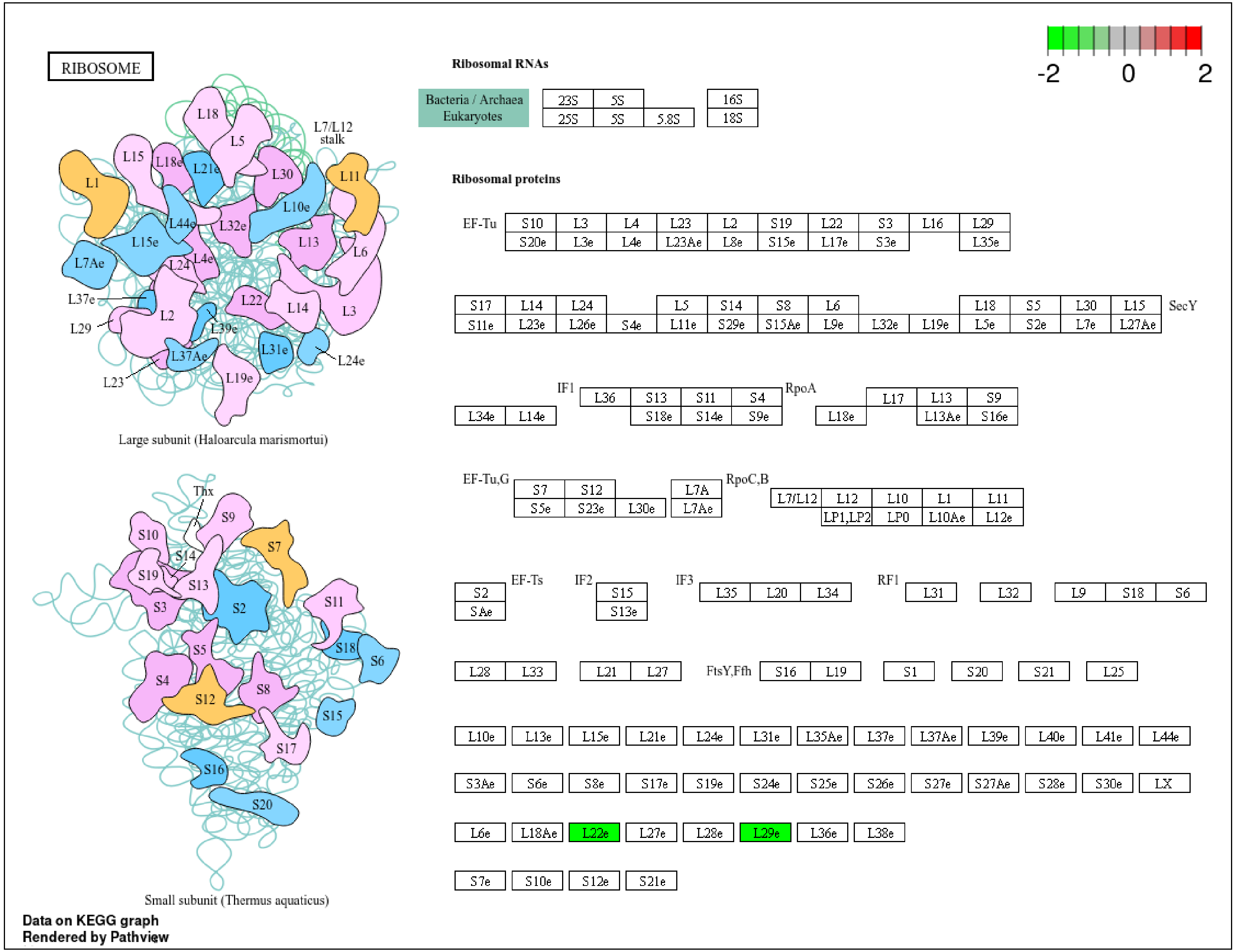

Figure S15. Ribosome pathway in SYNCH group in F1 (Cecal mucosa).

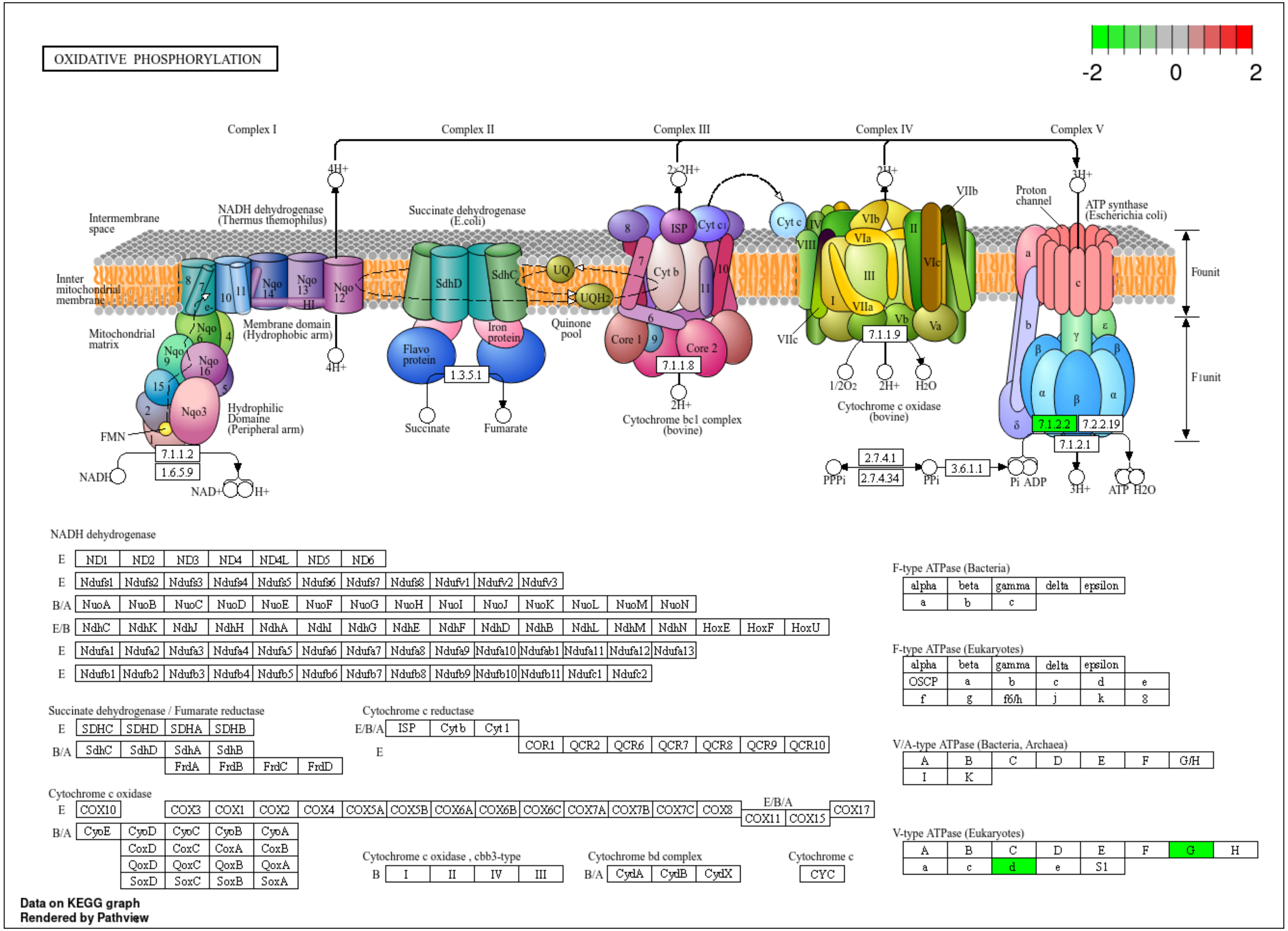

Figure S16. Oxidative phosphorylation pathway in SYNCH group in F1 (Cecal mucosa).

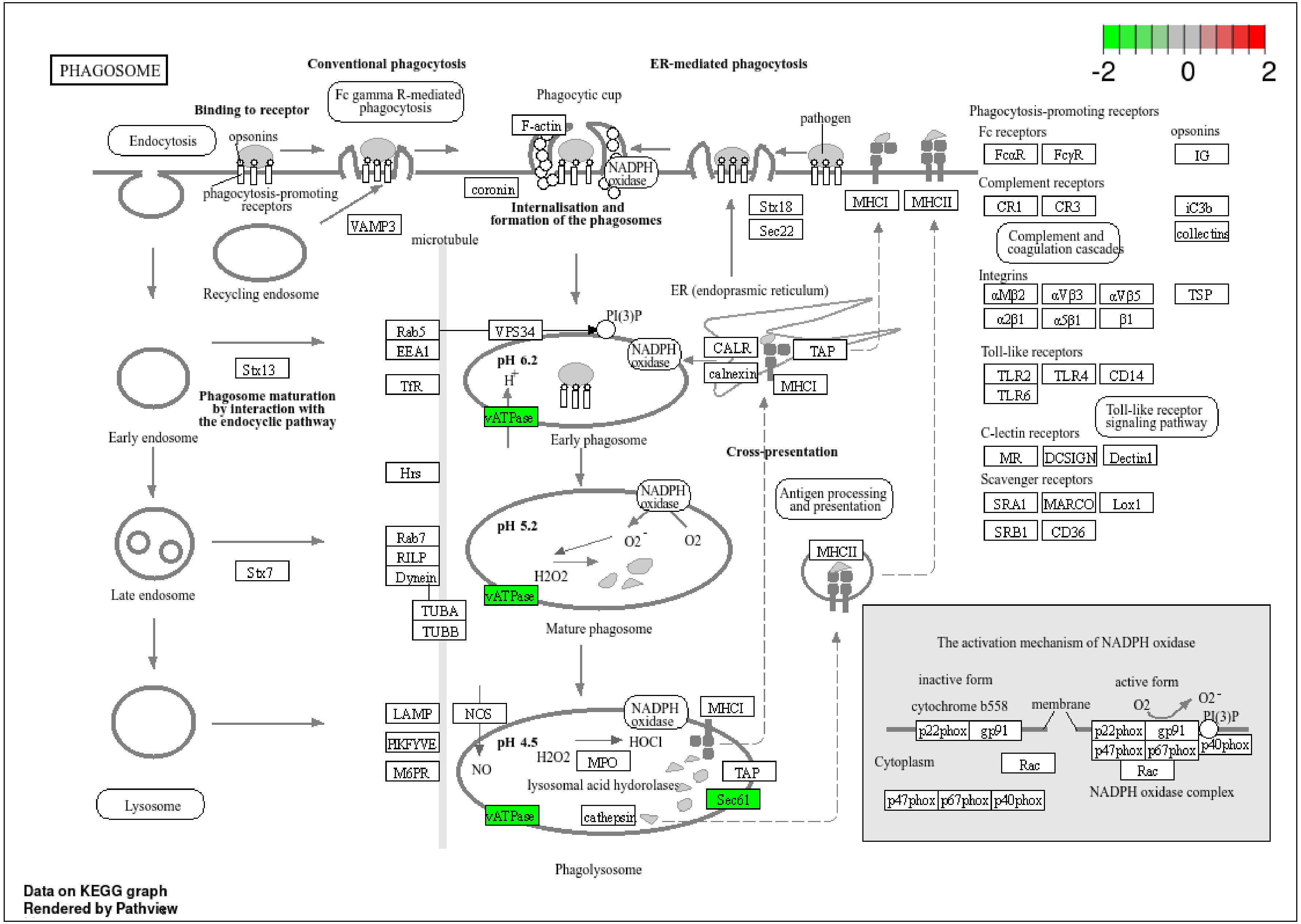

Figure S17. Phagosome pathway in SYNCH group in F1 (Cecal mucosa).

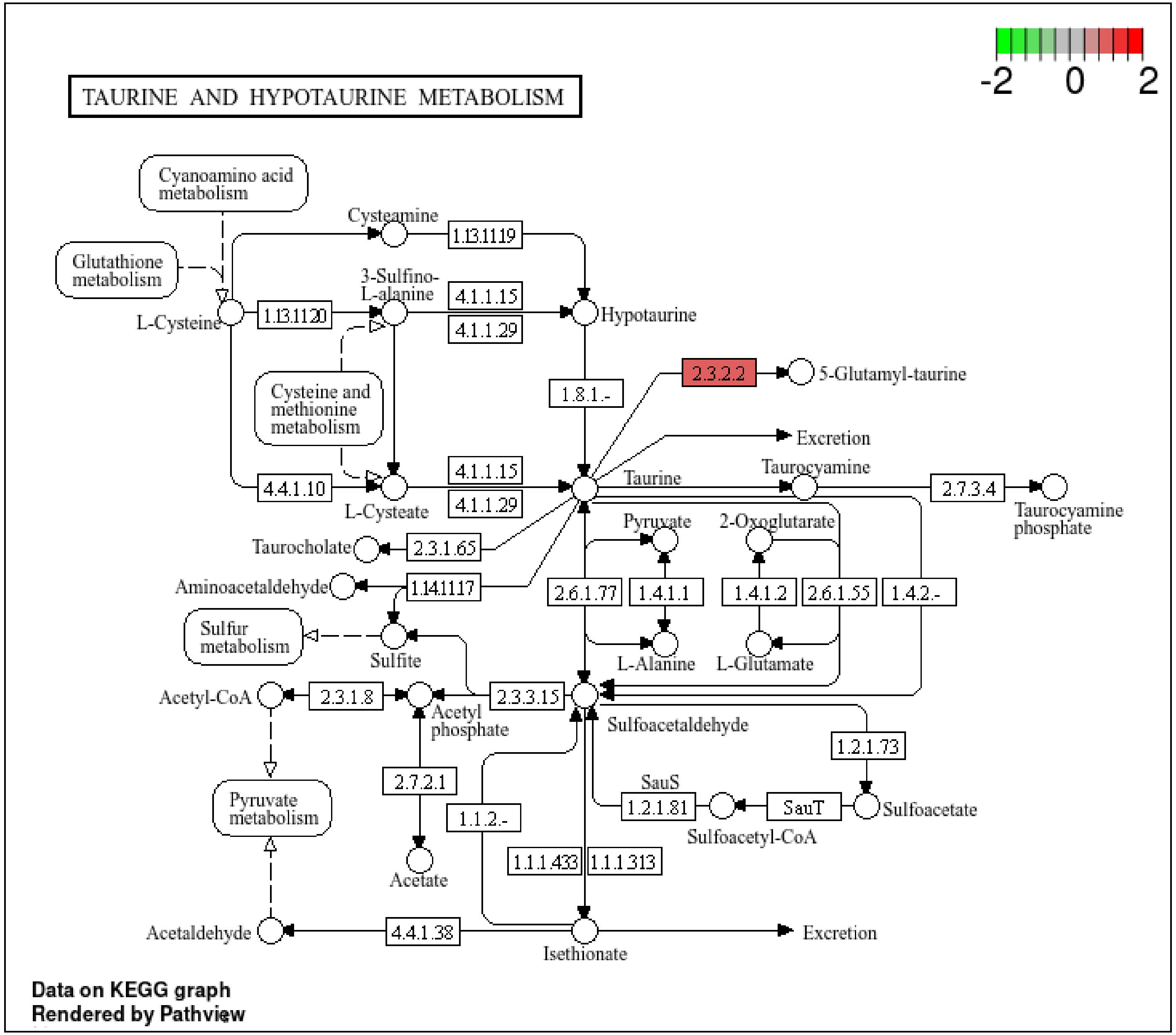

Figure S18. Taurine and hypotaurine metabolism pathway in SYNs group in F2 (Cecal mucosa).

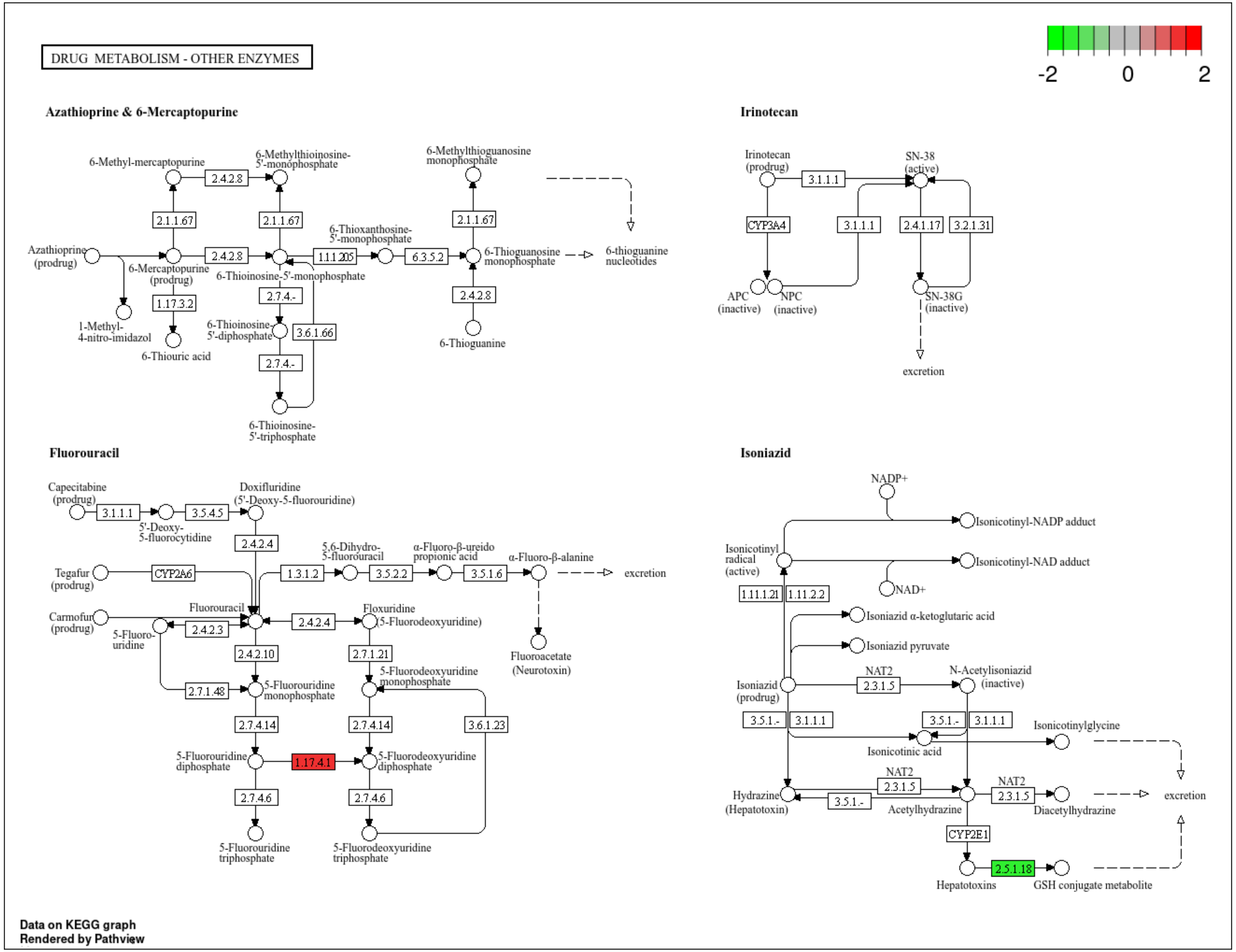

Figure S19. Drug metabolism – other enzymes pathway in SYNs group in F2 (Cecal mucosa).

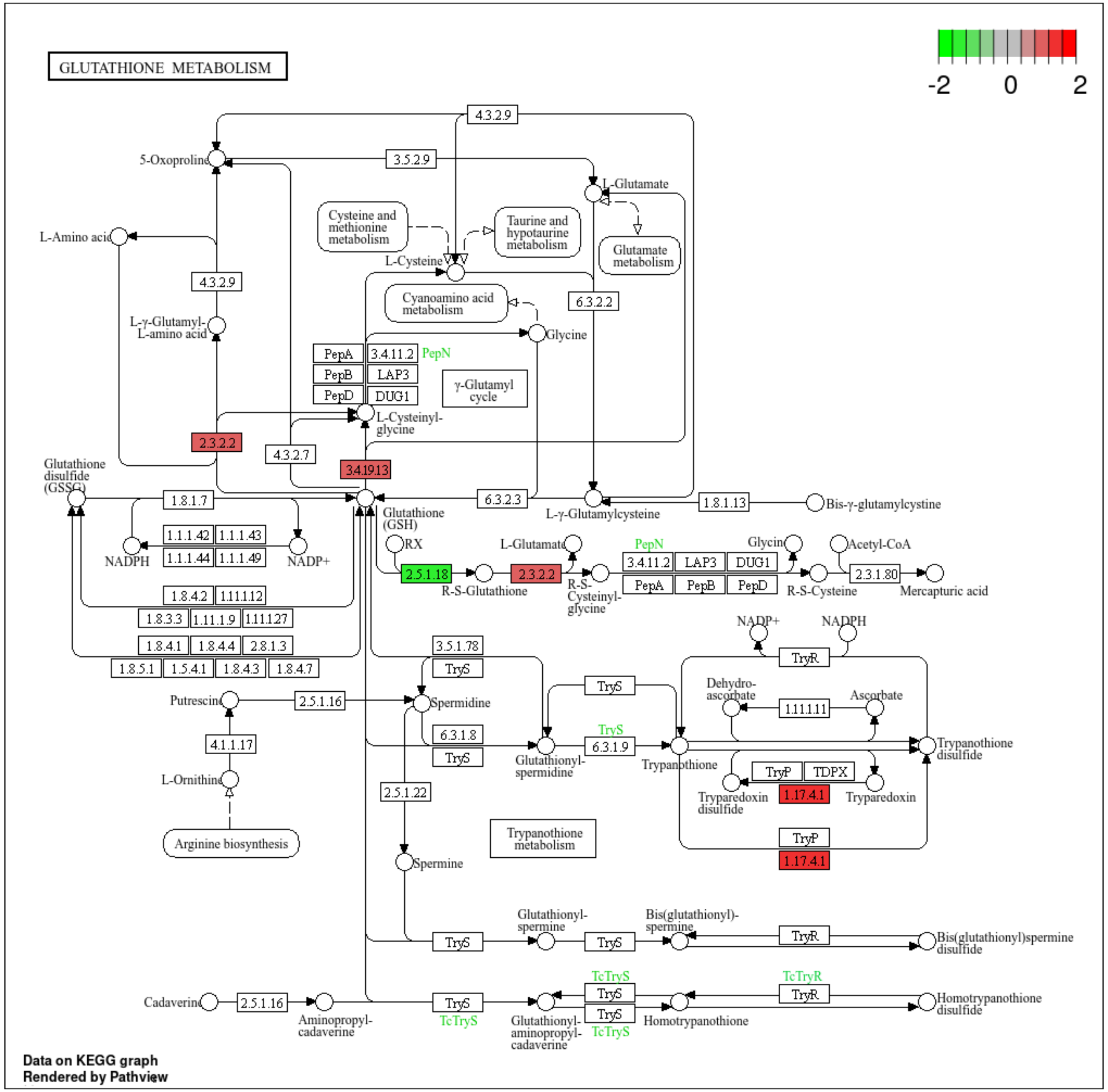

Figure S20. Glutathione metabolism pathway in SYNs group in F2 (Cecal mucosa).

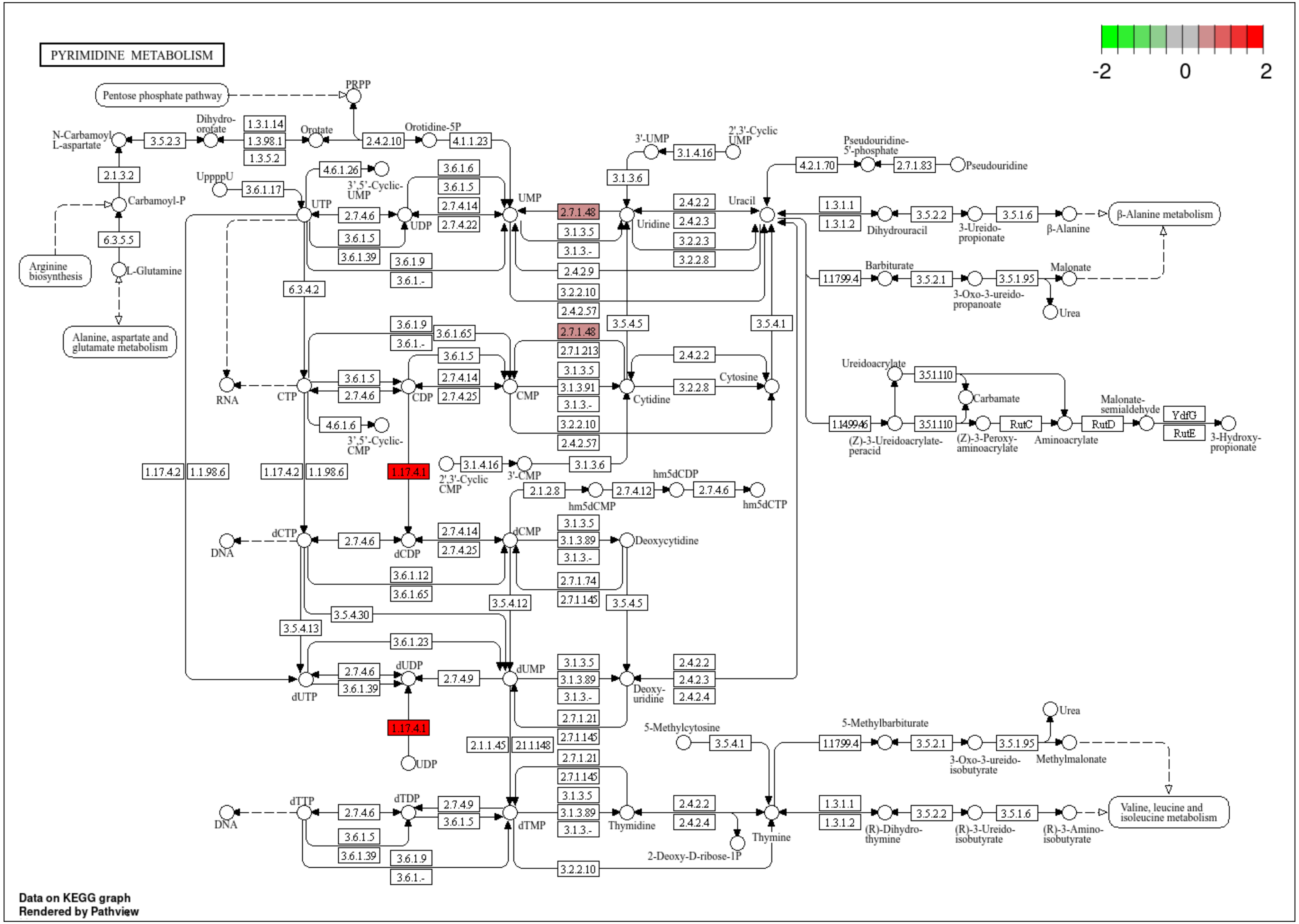

Figure S21. Pyrimidine metabolism pathway in SYNr group in F2 (Cecal mucosa).

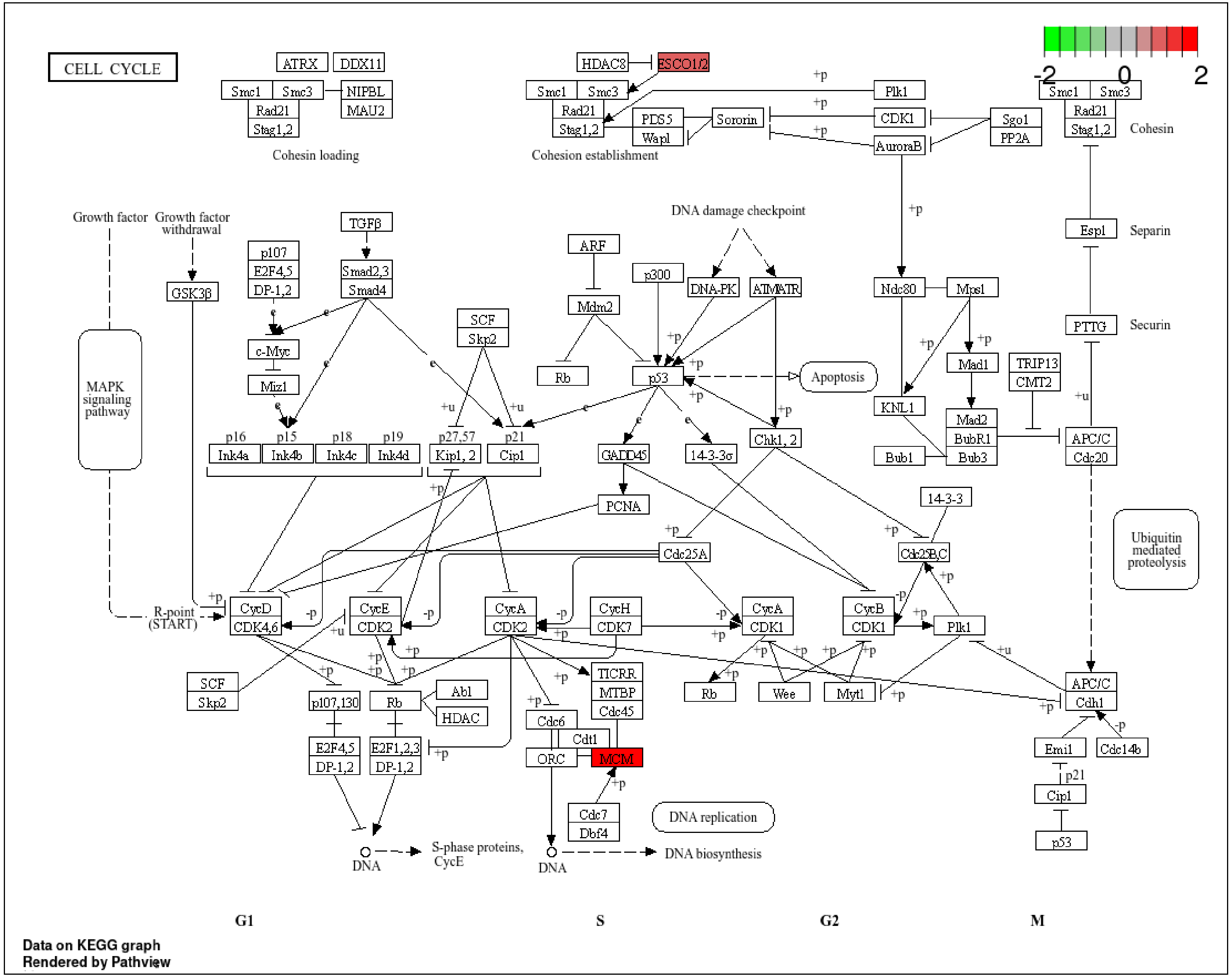

Figure S22. Cell cycle pathway in SYNr group in F2 (Cecal mucosa).

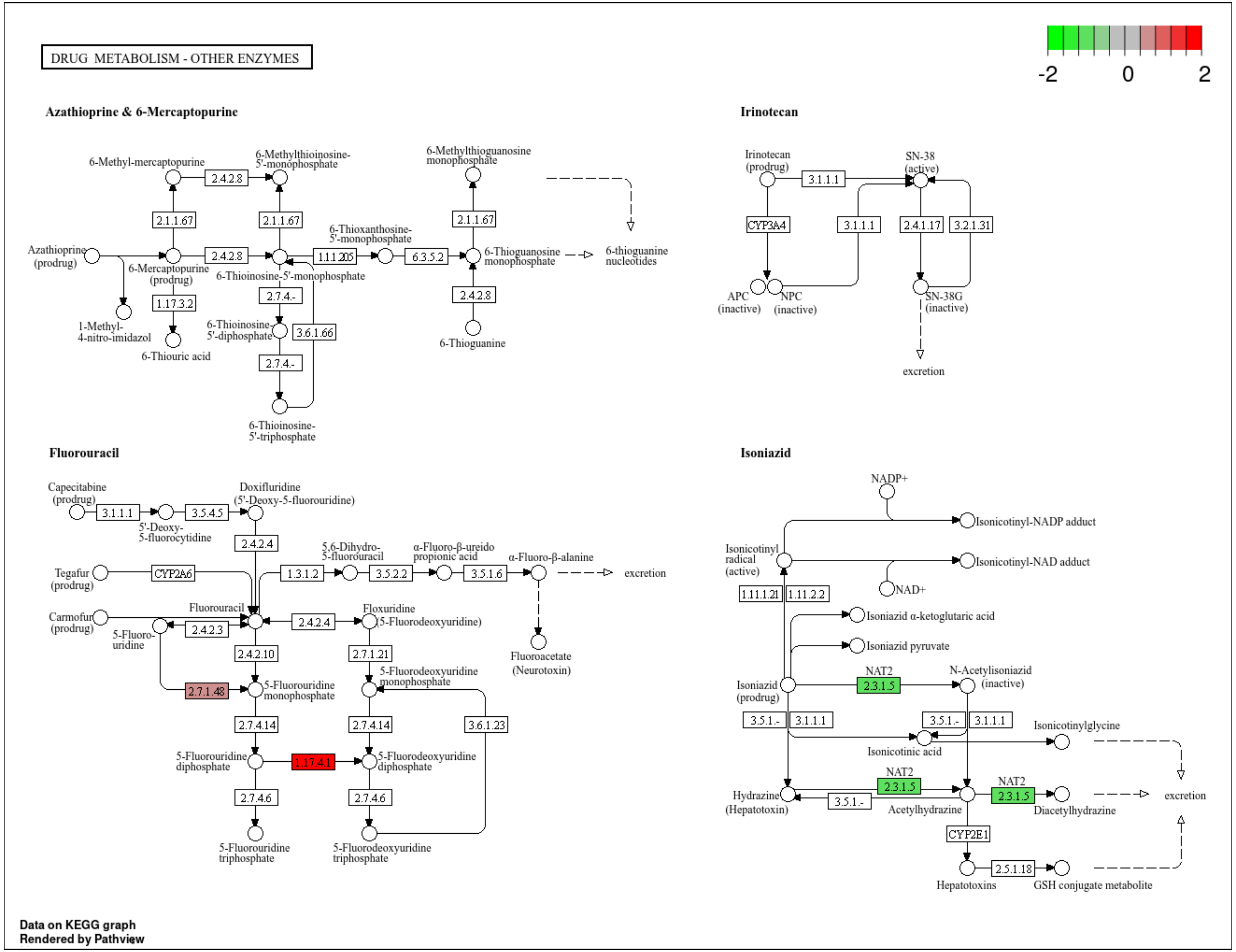

Figure S23. Drug metabolism – other enzymes pathway in SYNr group in F2 (Cecal mucosa).

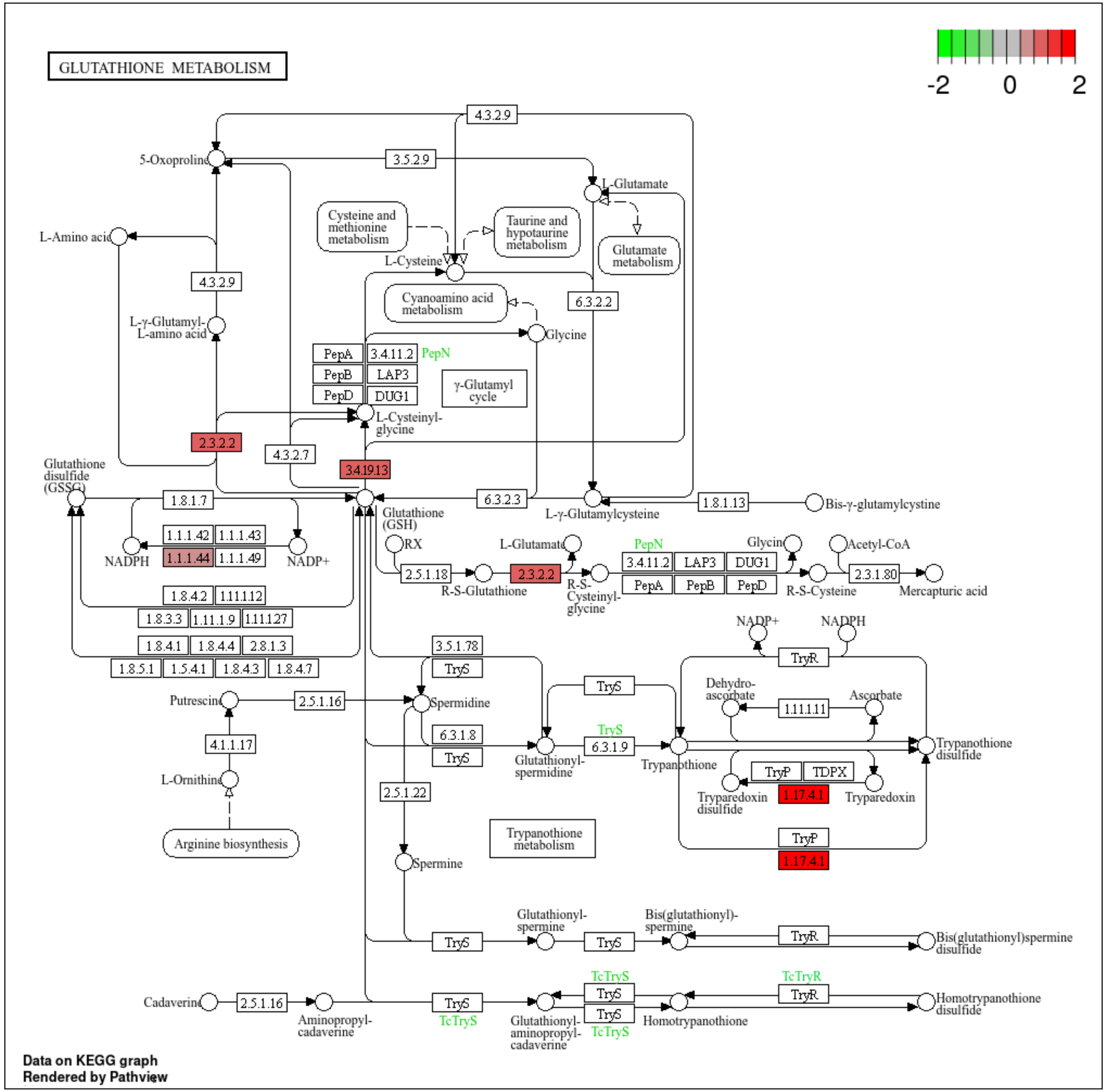

Figure S24. Glutathione metabolism pathway in SYNr group in F2 (Cecal mucosa).

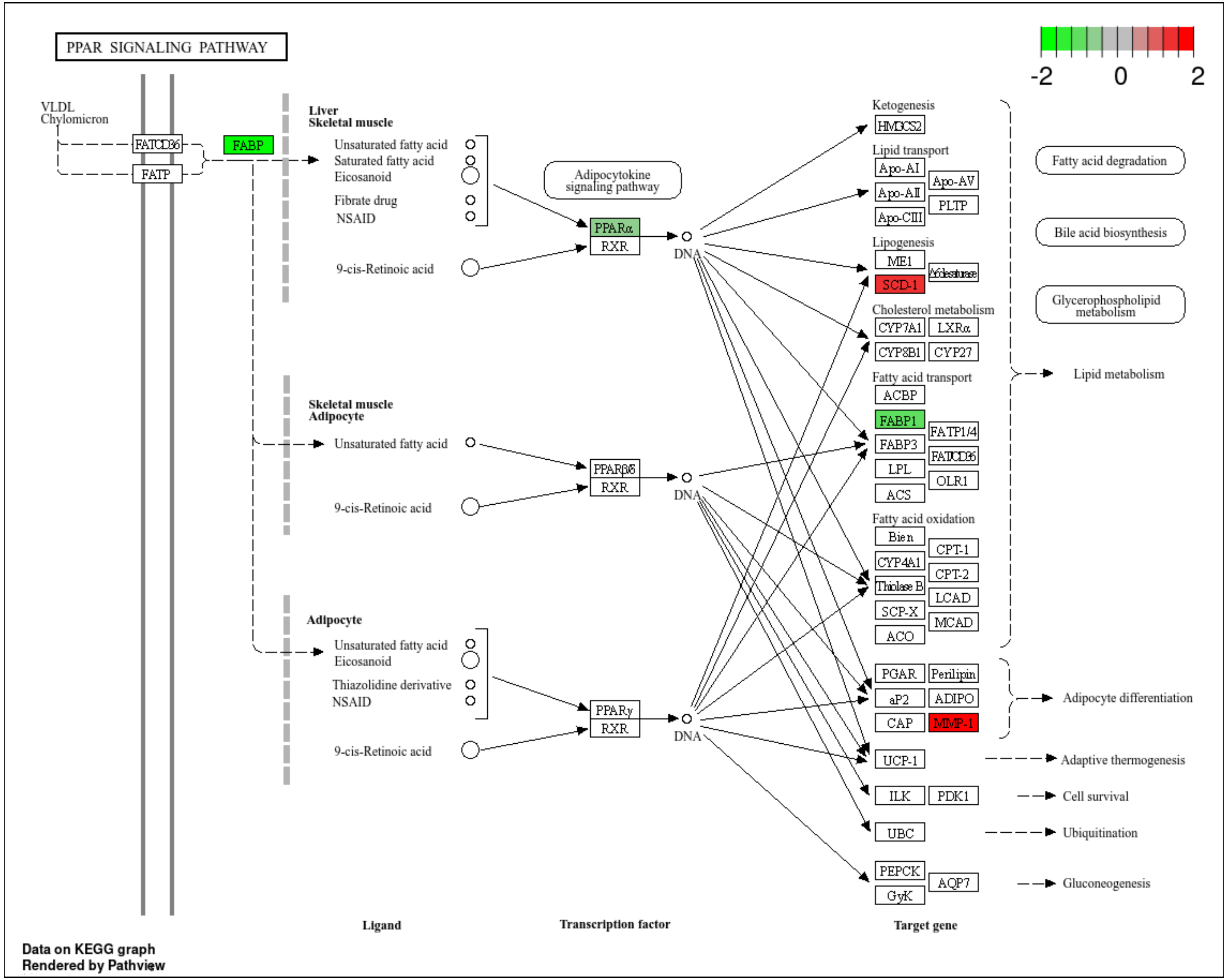

Figure S25. PPAR signaling pathway in SYNr group in F2 (Cecal mucosa)

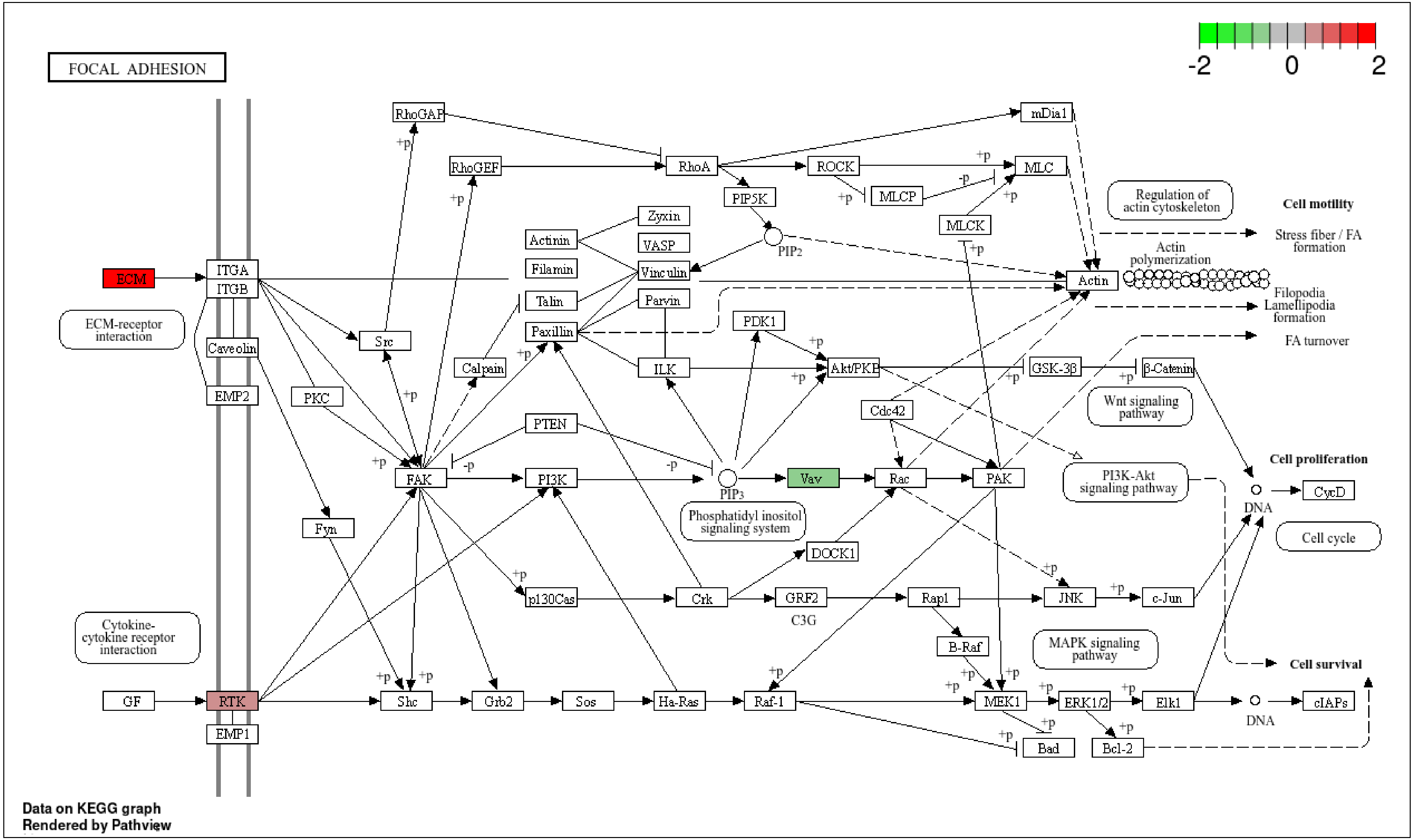

Figure S26. Focal adhesion pathway in SYNr group in F2 (Cecal mucosa).

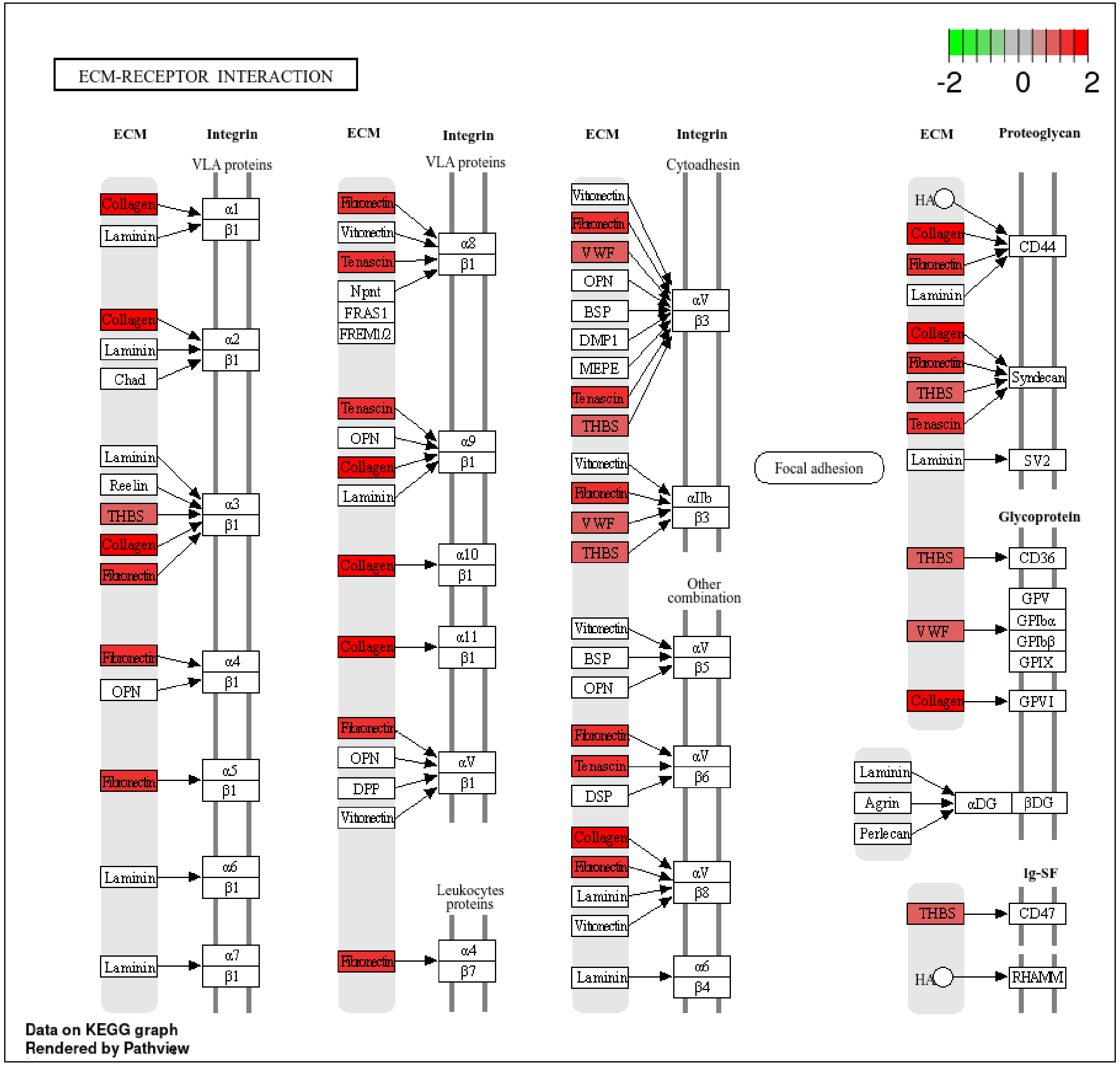

Figure S27. ECM-Receptor interaction pathway in SYNr group in F2 (Cecal mucosa).

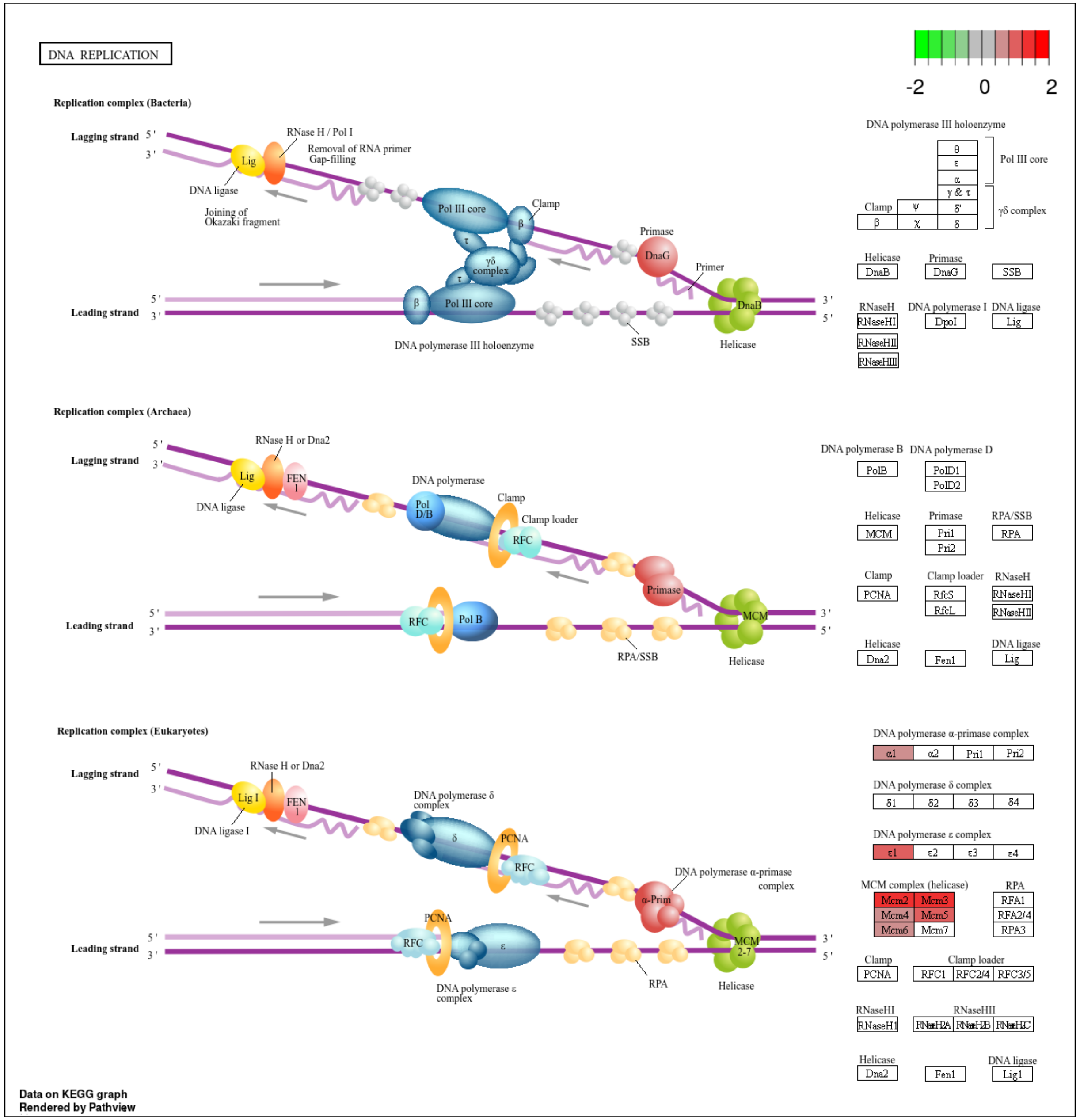

Figure S28. DNA replication pathway in SYNr group in F2 (Cecal mucosa).

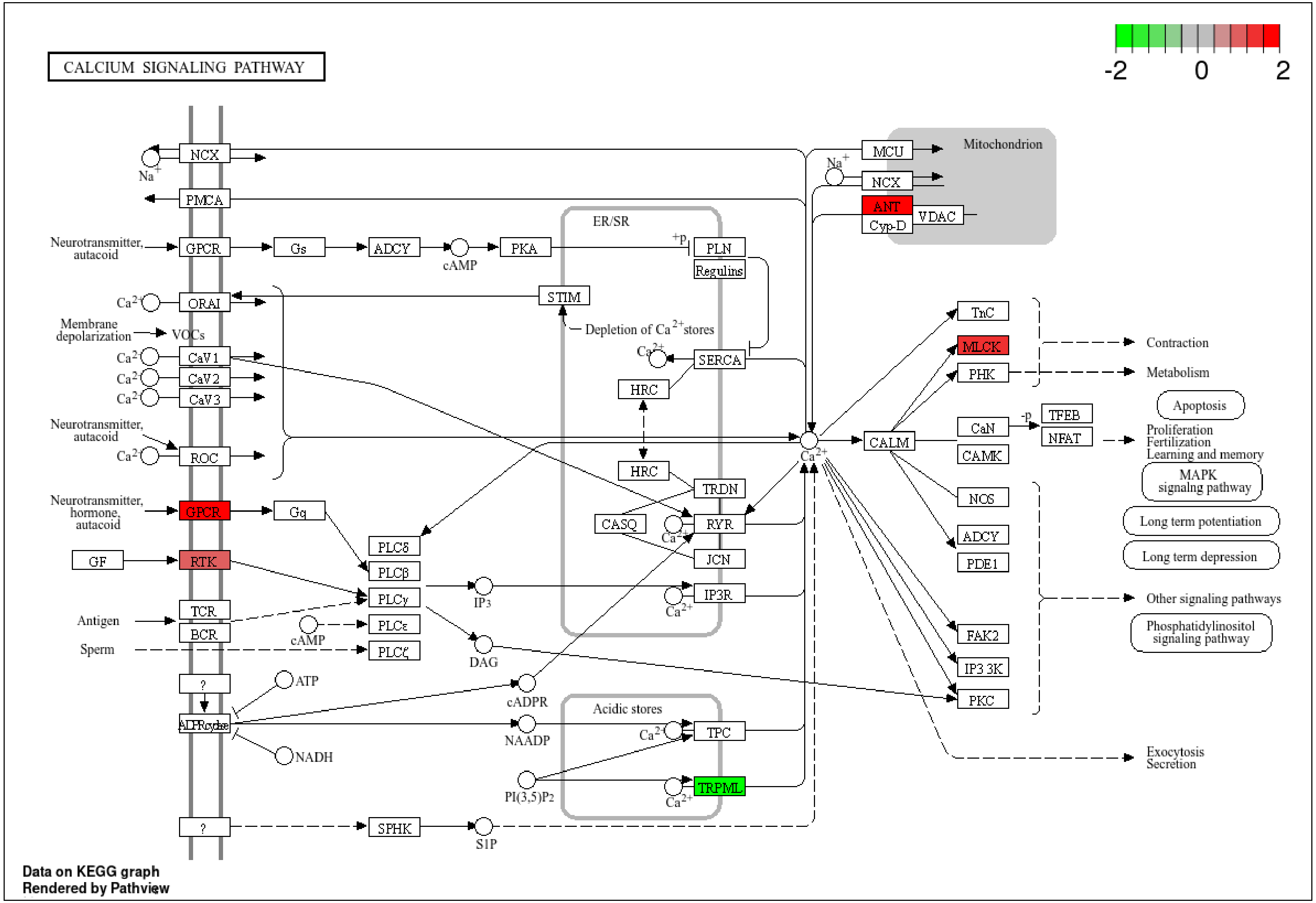

Figure S29. Calcium signaling pathway in SYNCHs group in F2 (Cecal mucosa).

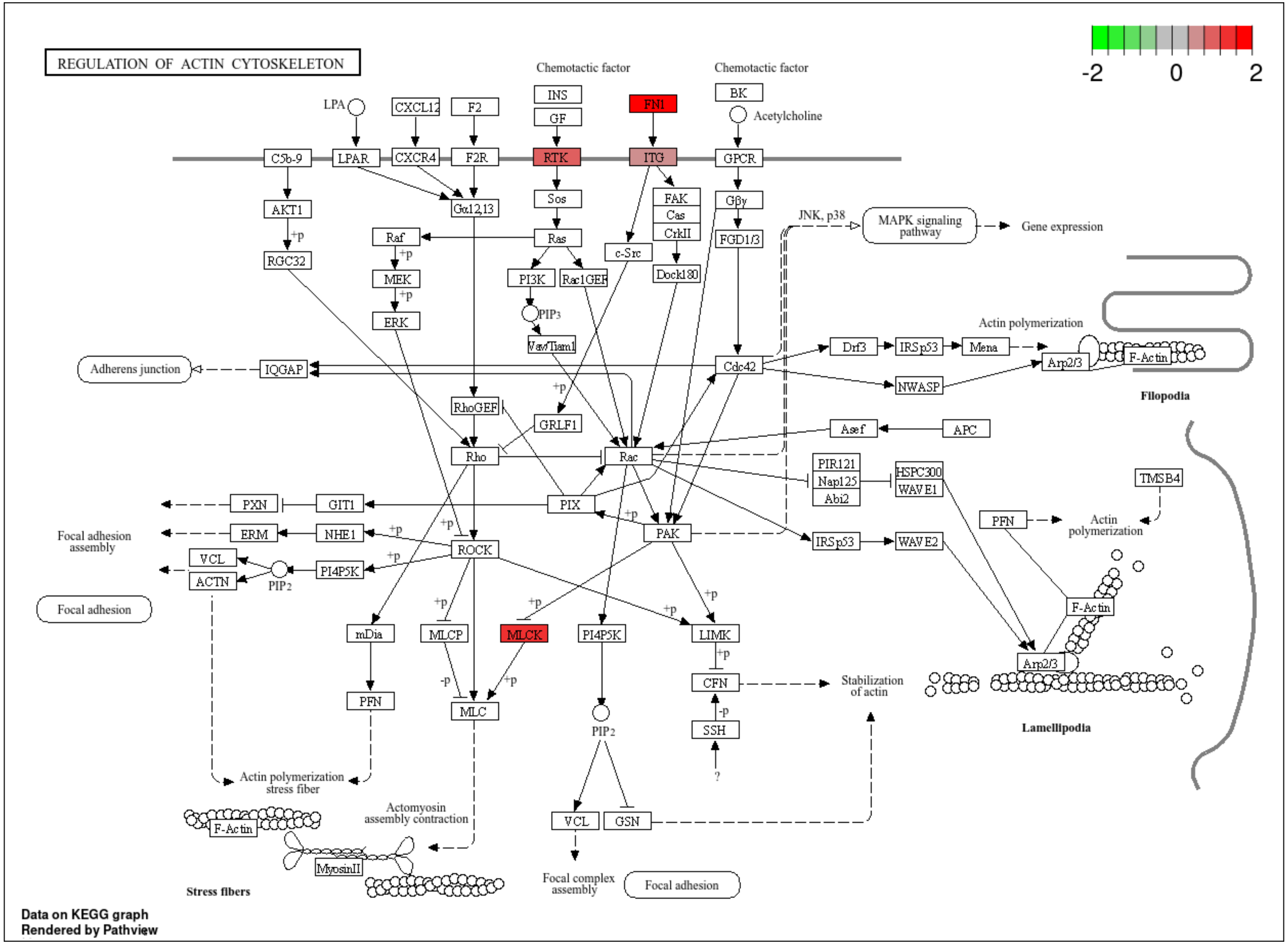

Figure S30. Regulation of actin cytoskeleton pathway in SYNCHs group in F2 (Cecal mucosa).

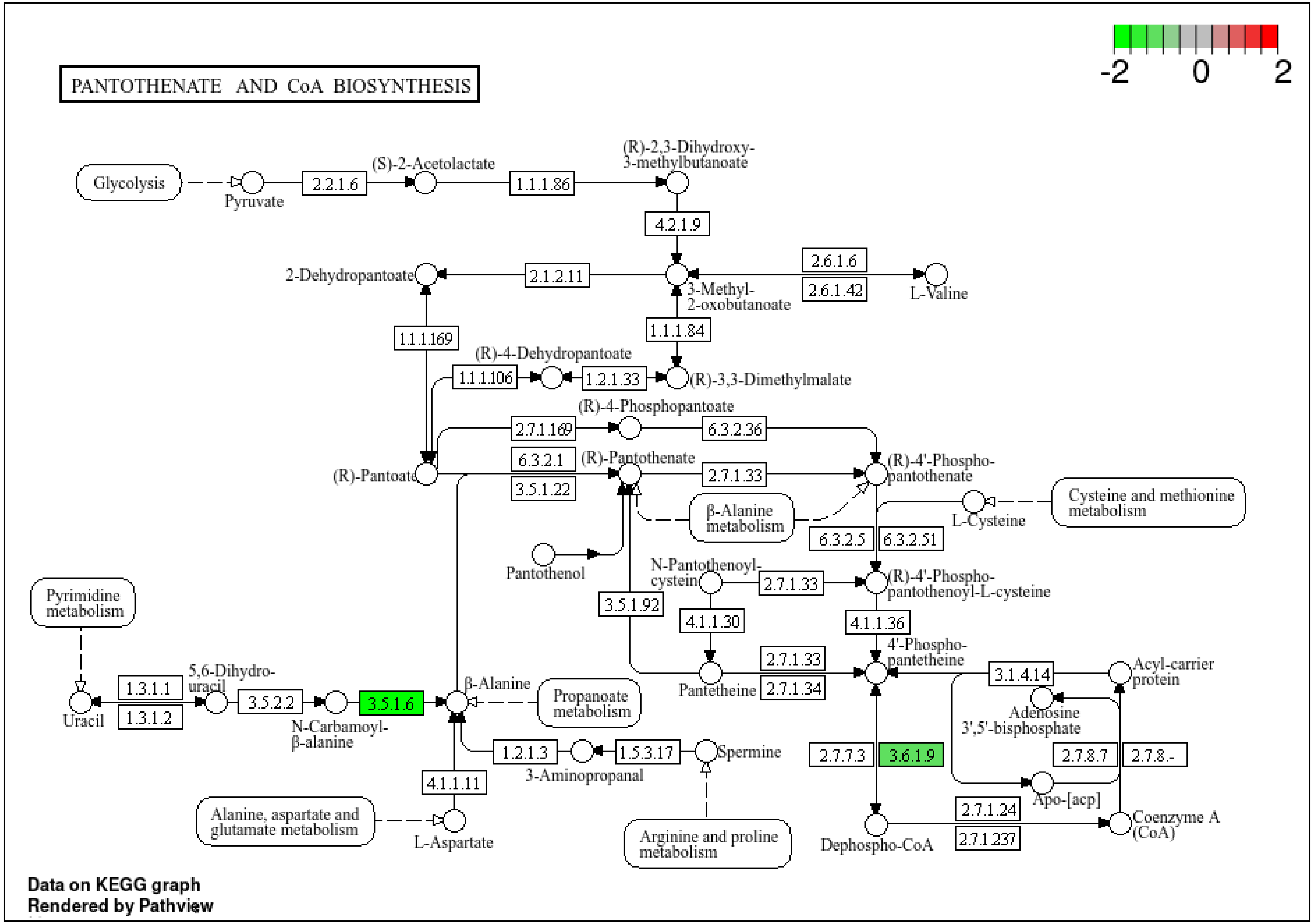

Figure S31. Pantothenate and CoA Biosynthesis pathway in SYNCHs group in F2 (Cecal mucosa).

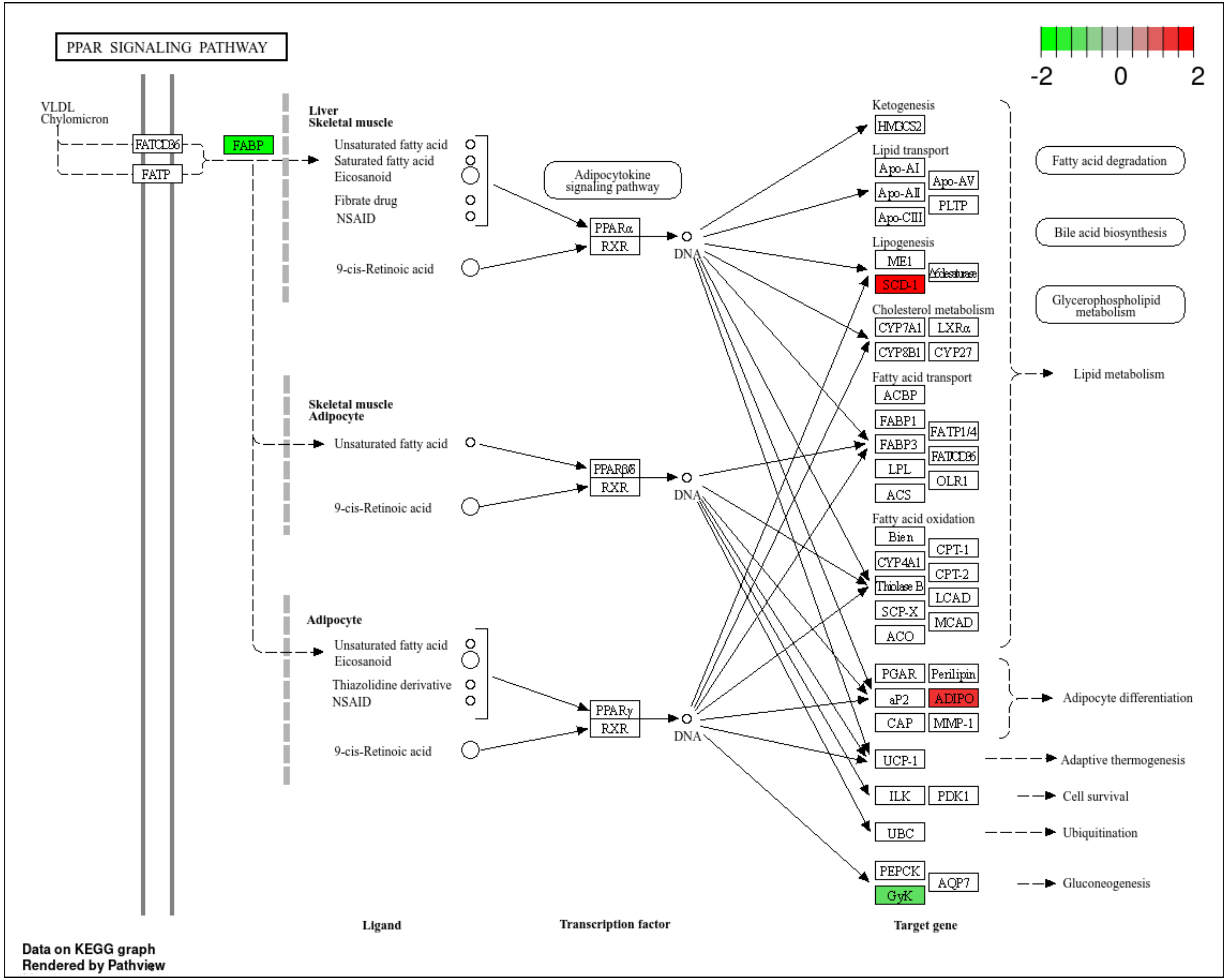

Figure S32. PPAR signaling pathway in SYNCHs group in F2 (Cecal mucosa).

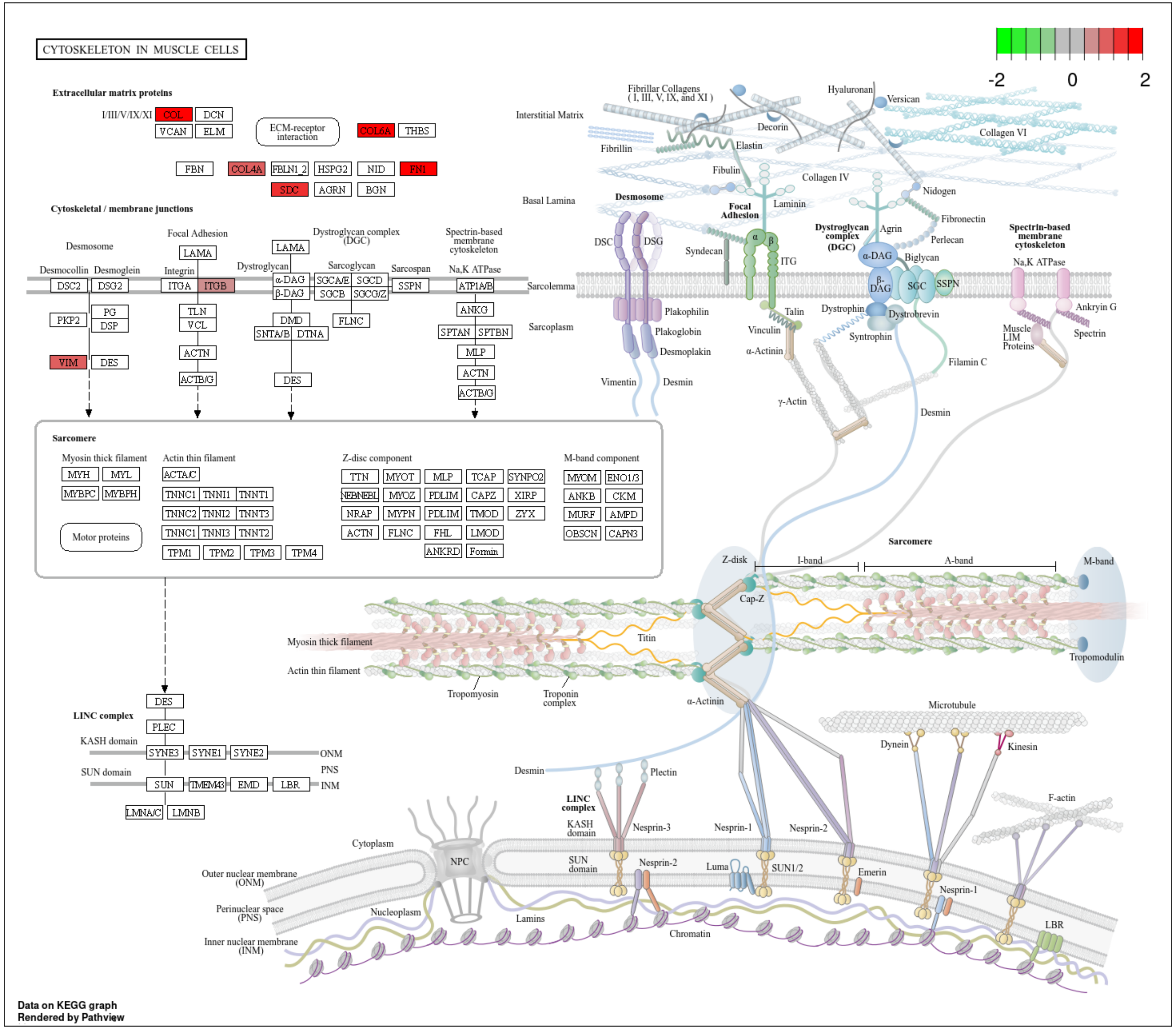

Figure S33. Cytoskeleton in muscle cells pathway in SYNCHs group in F2 (Cecal mucosa).

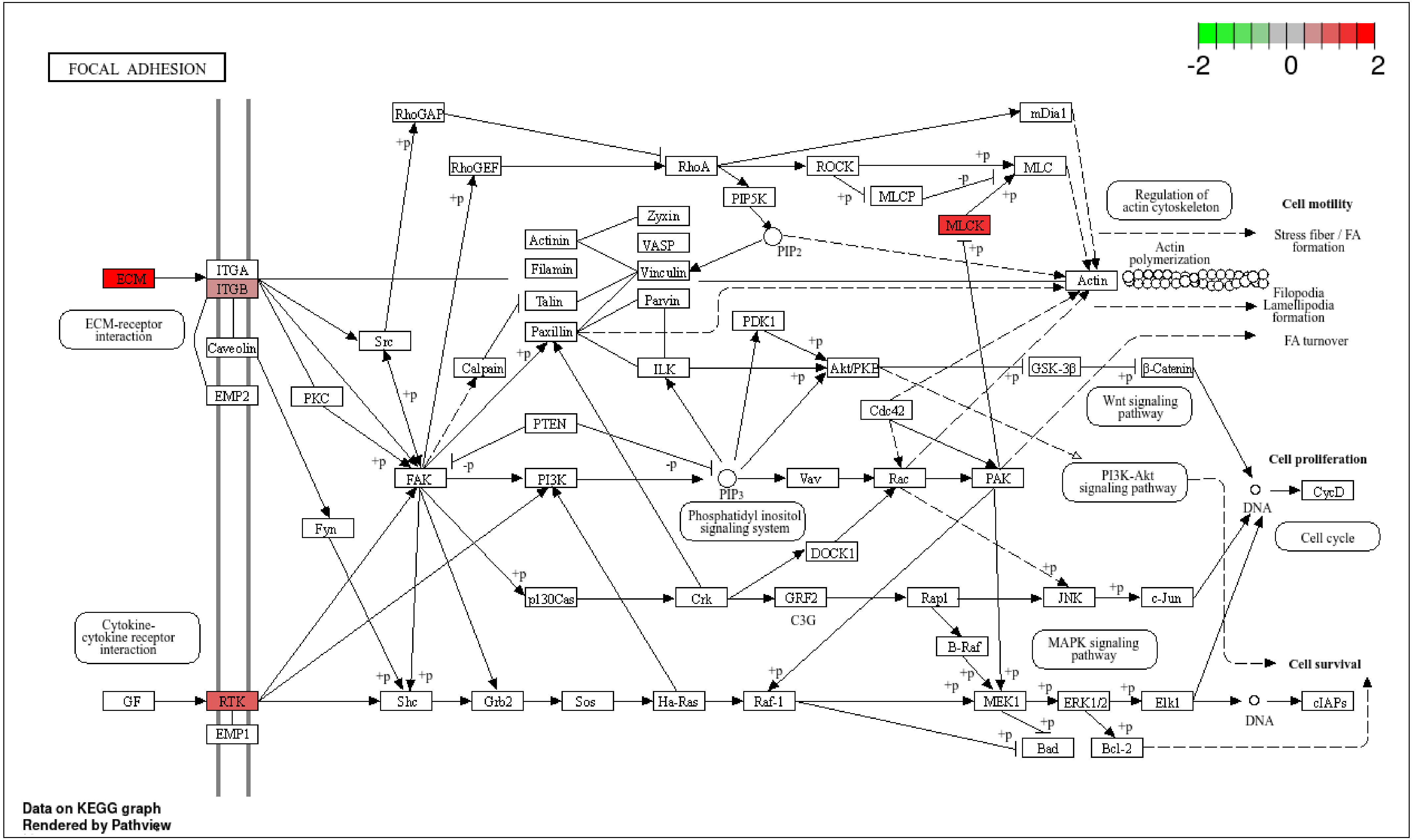

Figure S34. Focal adhesion pathway in SYNCHs group in F2 (Cecal mucosa).

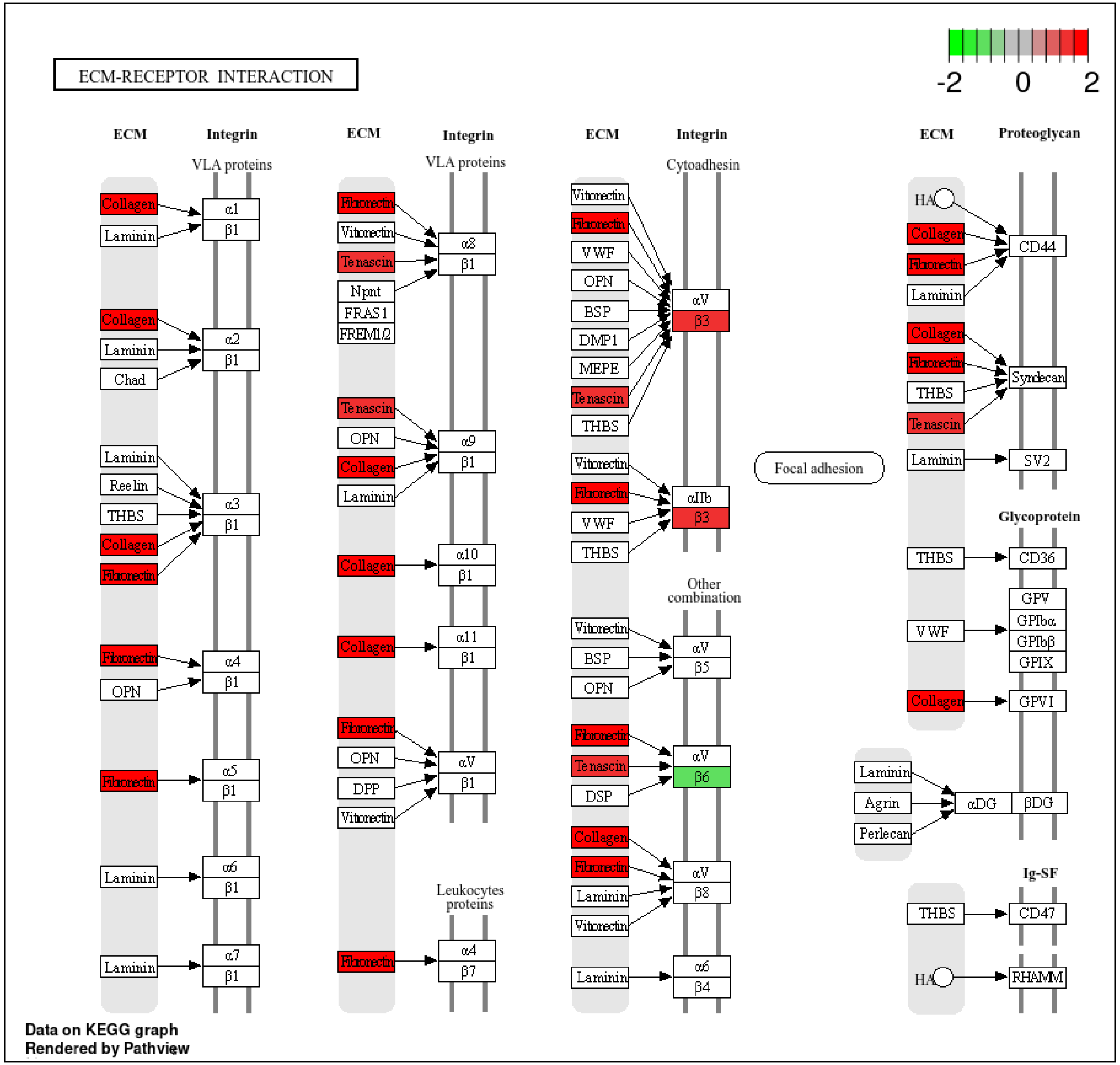

Figure S35. ECM- Receptor interaction pathway in SYNCHs group in F2 (Cecal mucosa).

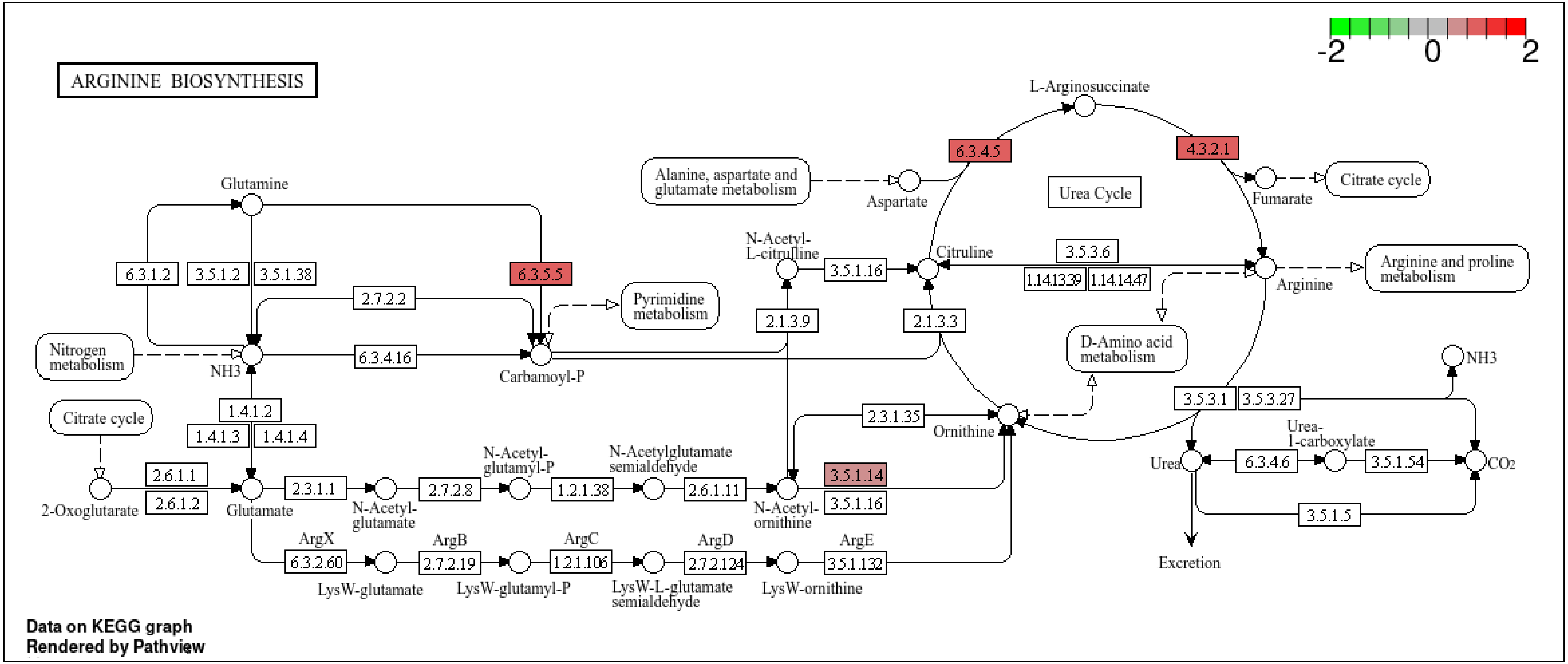

Figure S36. Arginine Biosynthesis pathway in SYNCHr group in F2 (Cecal mucosa).

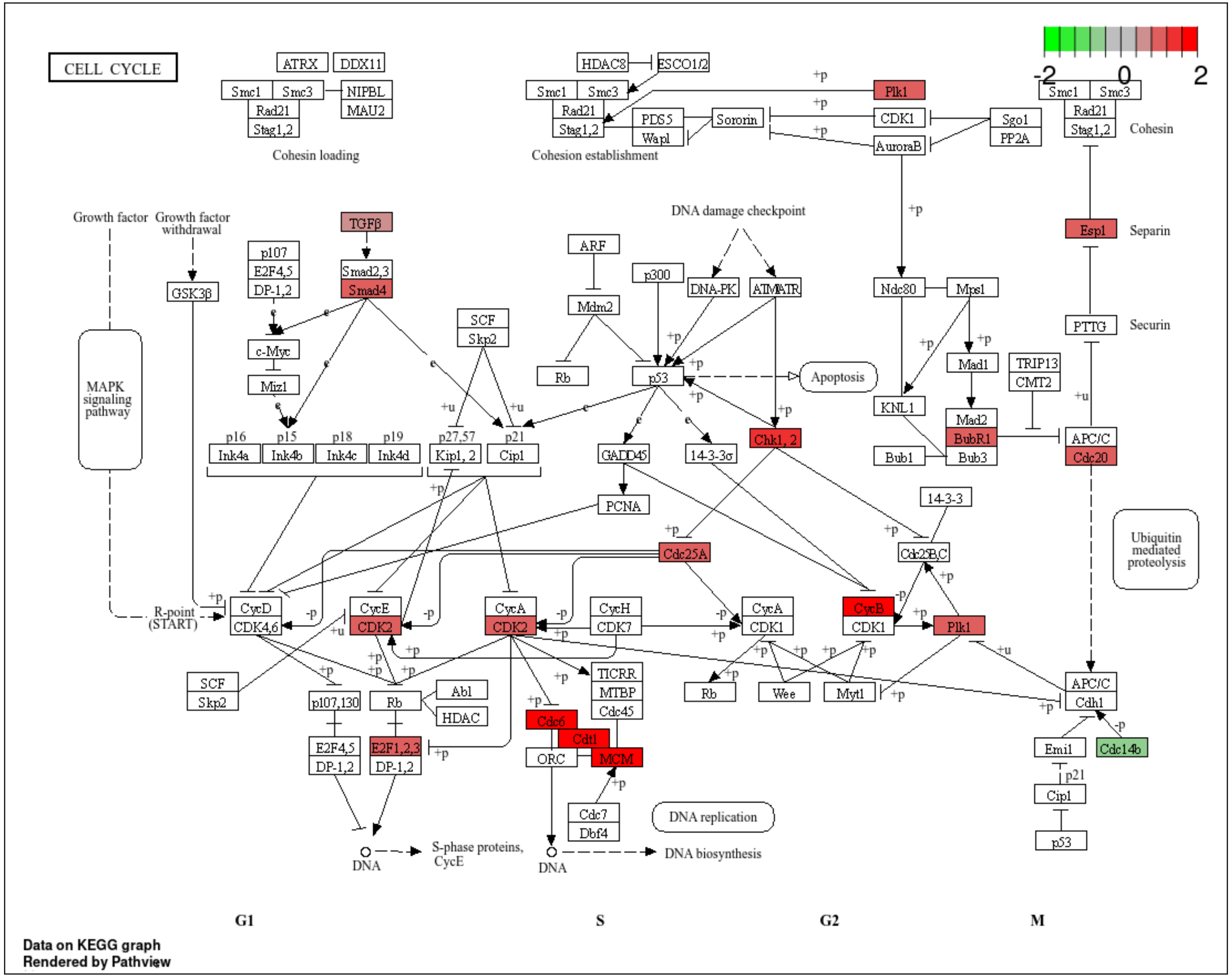

Figure S37. Cell cycle pathway in SYNCHr group in F2 (Cecal mucosa).

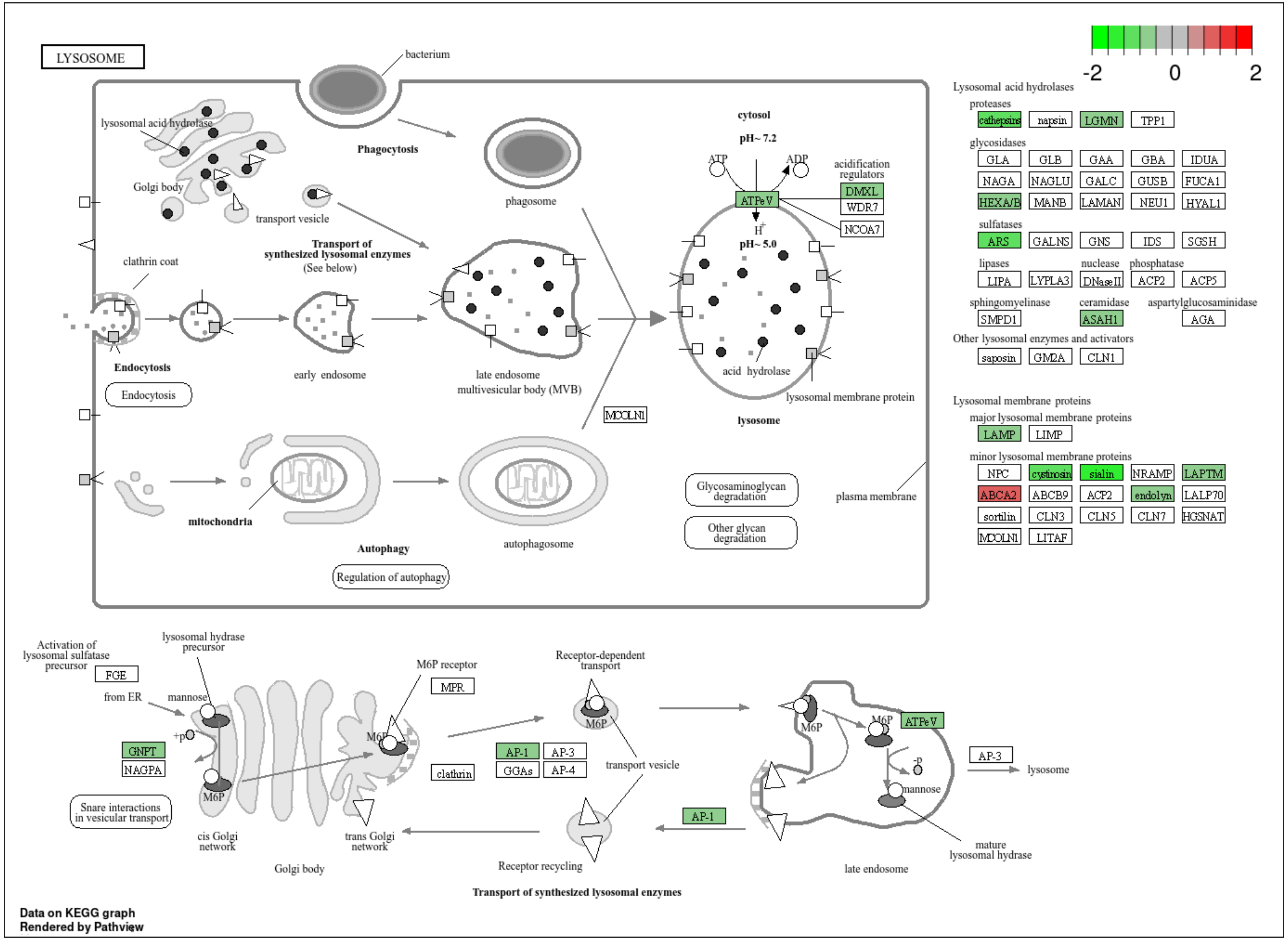

Figure S38. Lysosome pathway in SYNCHr group in F2 (Cecal mucosa).

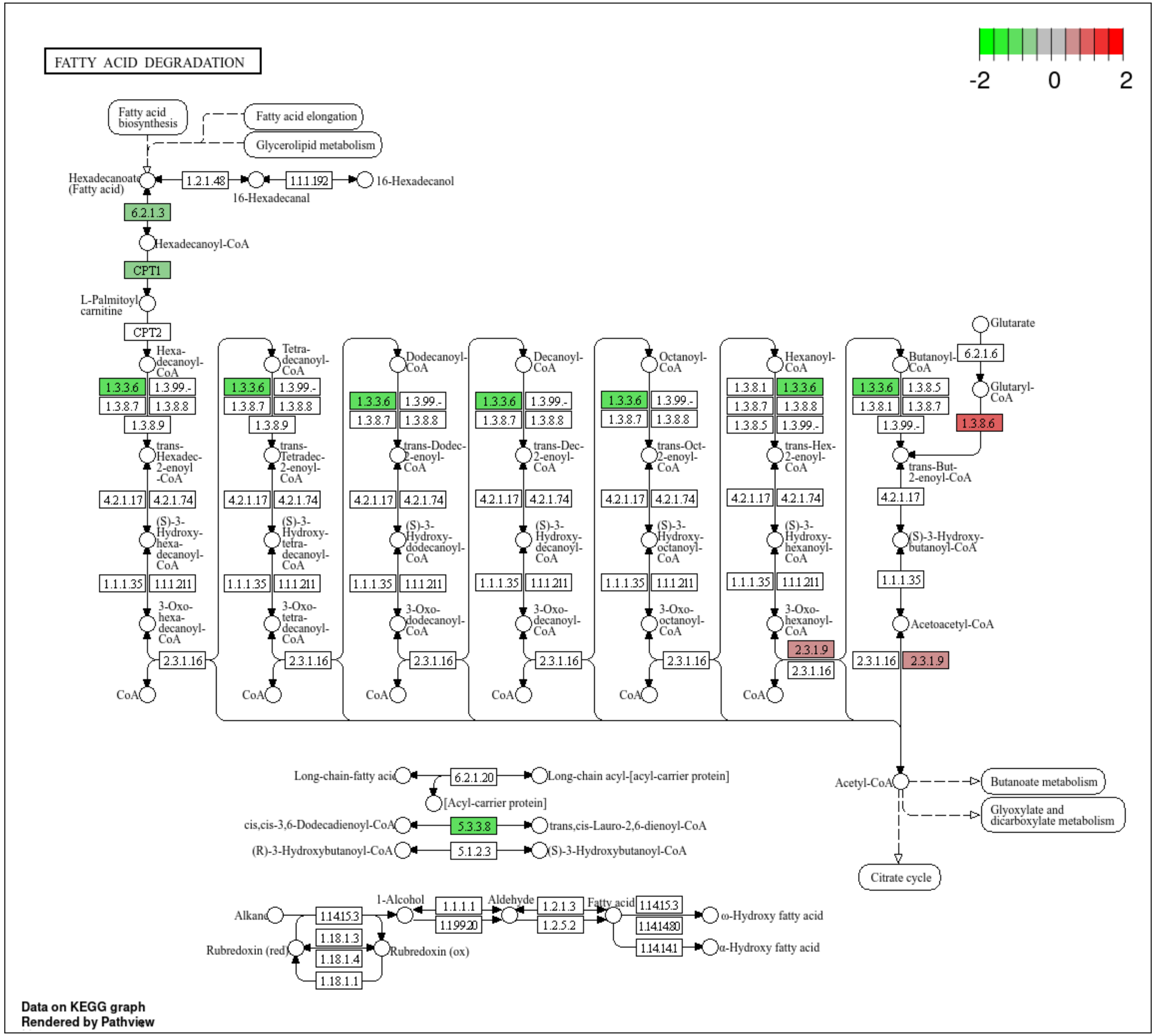

Figure S39. Fatty acid degradation pathway in SYNCHr group in F2 (Cecal mucosa).

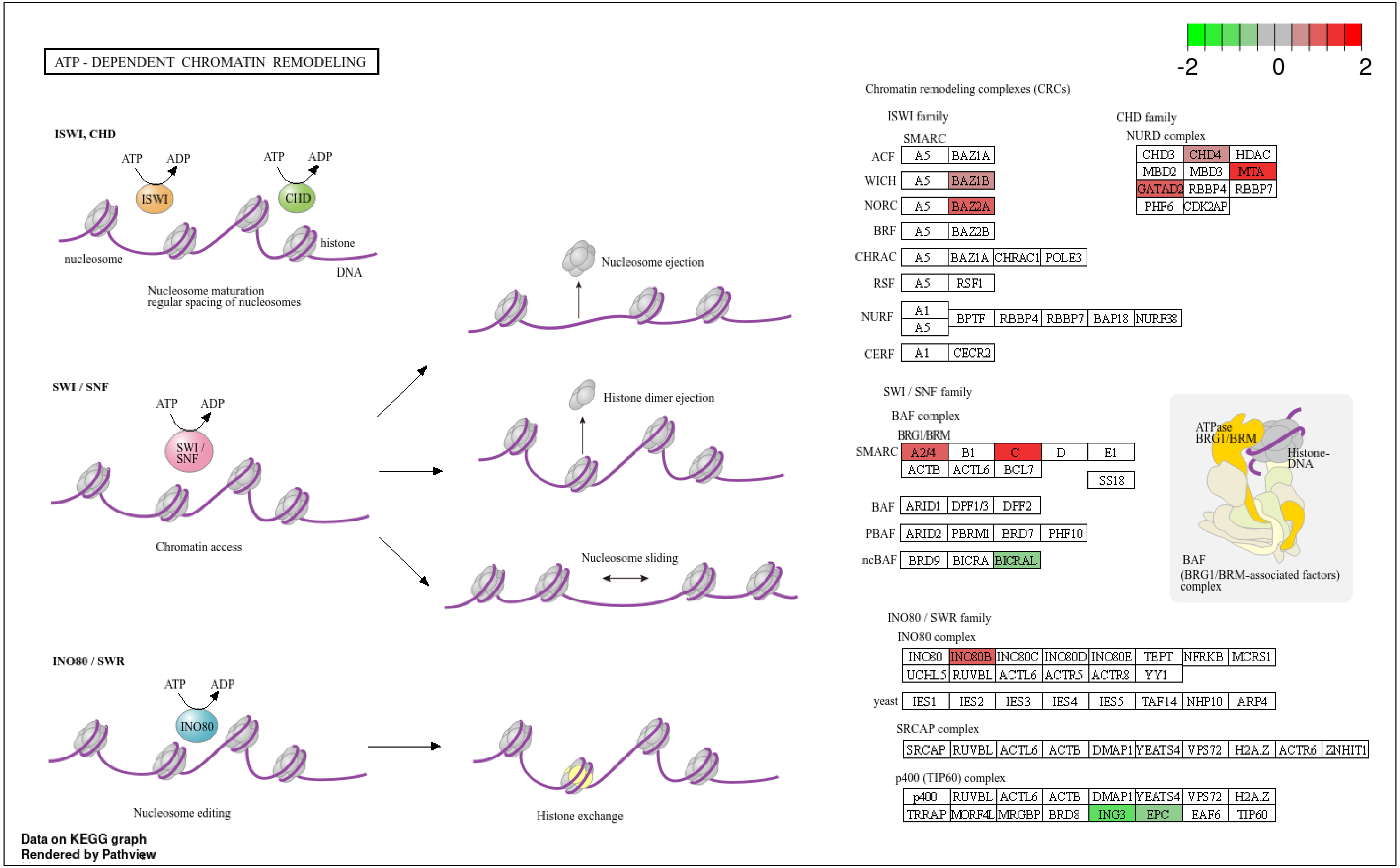

Figure S40. ATP – Dependent chromatin remodeling pathway in SYNCHr group in F2 (Cecal mucosa).

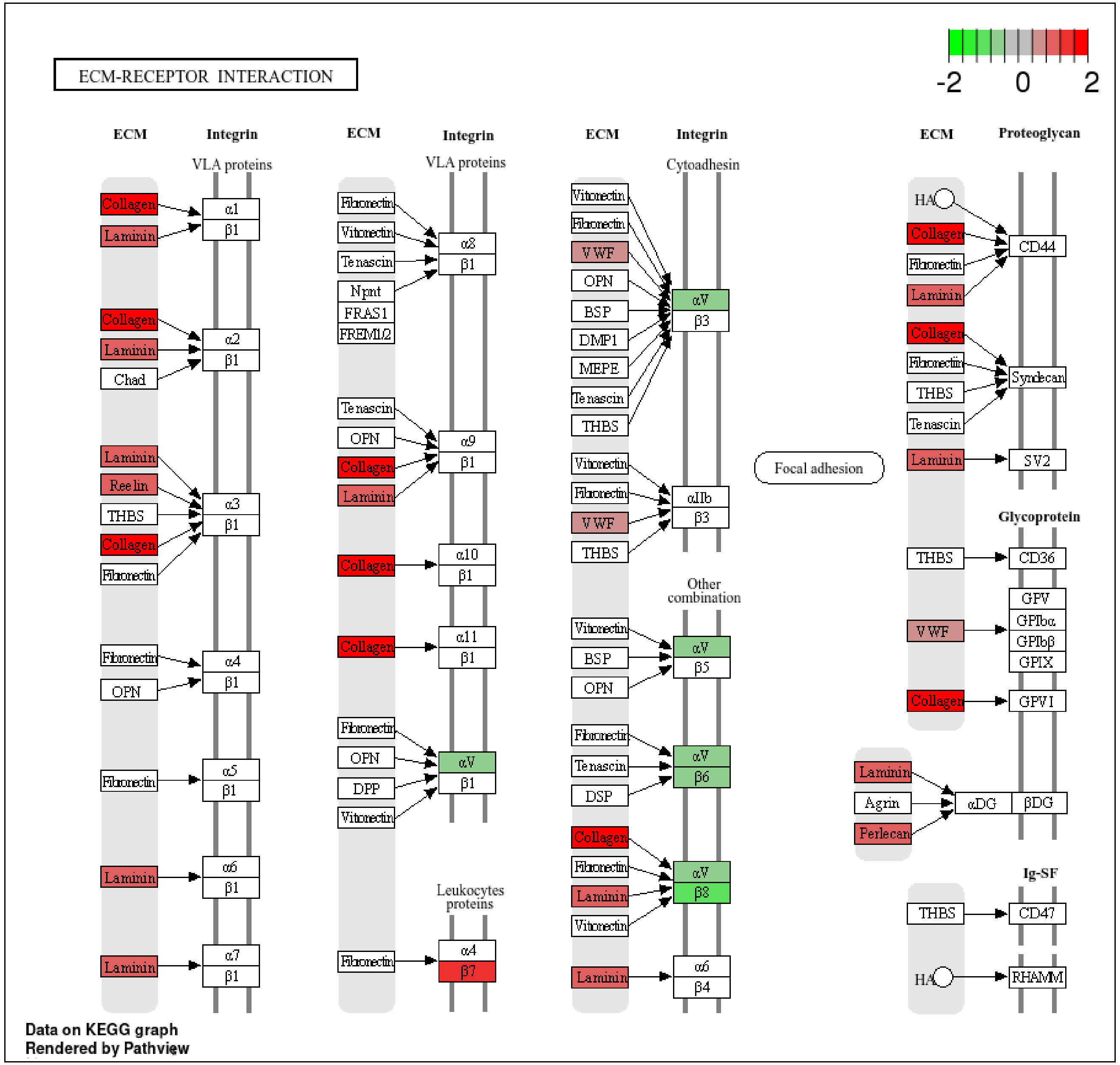

Figure S41. ECM – Receptor interaction pathway in SYNCHr group in F2 (Cecal mucosa).

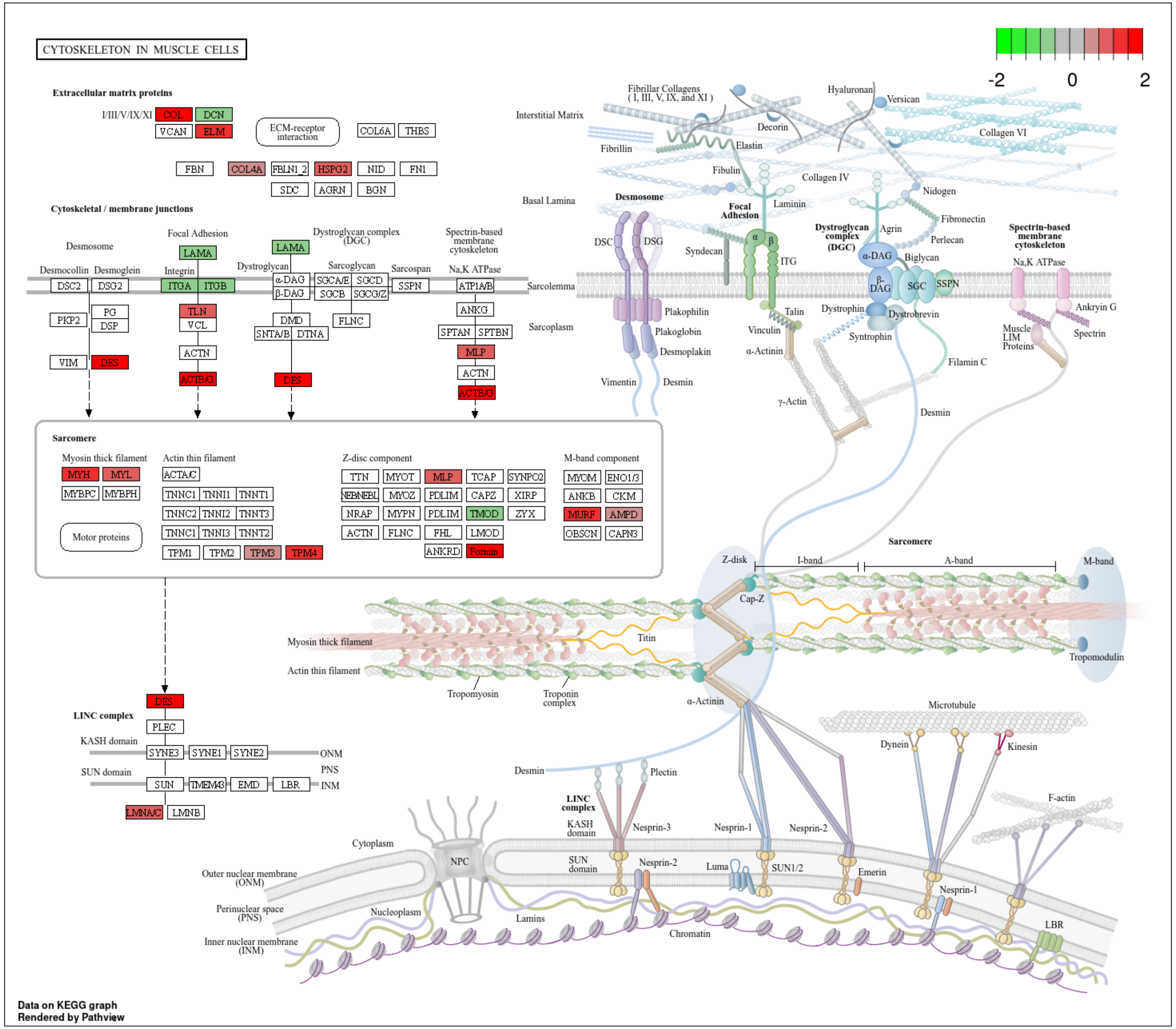

Figure S42. Cytoskeleton in muscle cells pathway in SYNCHr group in F2 (Cecal mucosa).

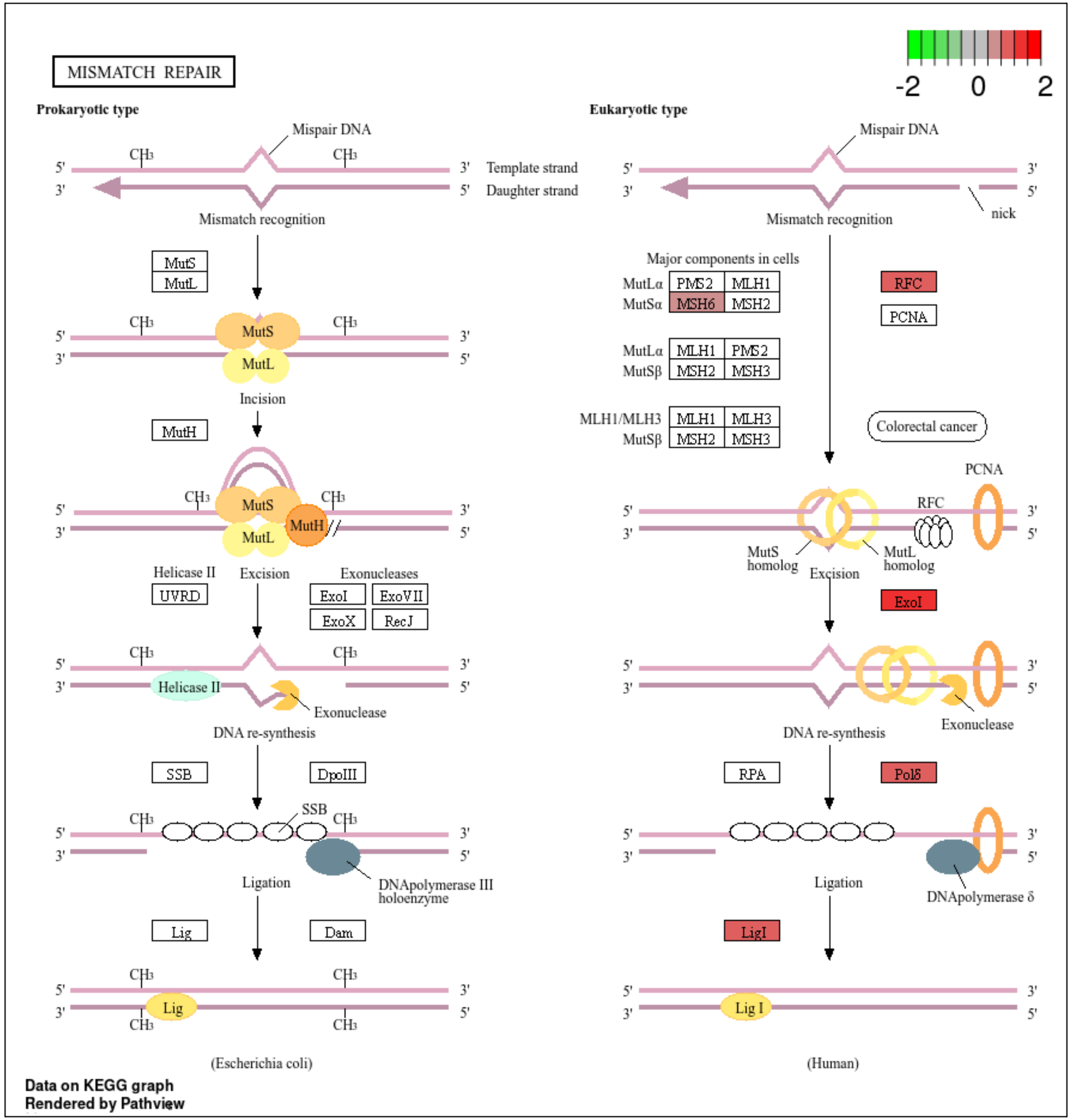

Figure S43. Mismatch repair pathway in SYNCHr group in F2 (Cecal mucosa).

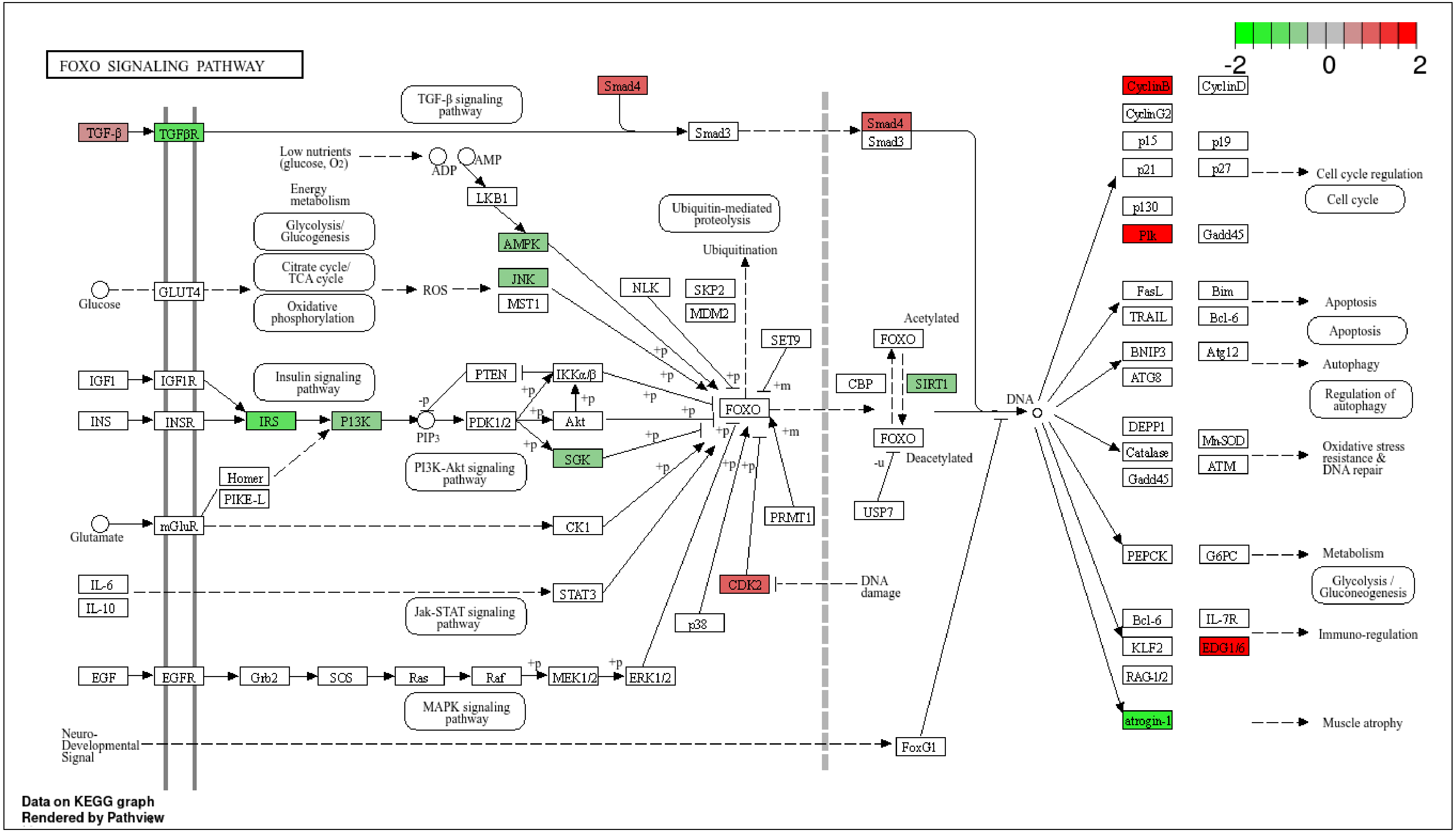

Figure S44. FOXO signaling pathway in SYNCHr group in F2 (Cecal mucosa).

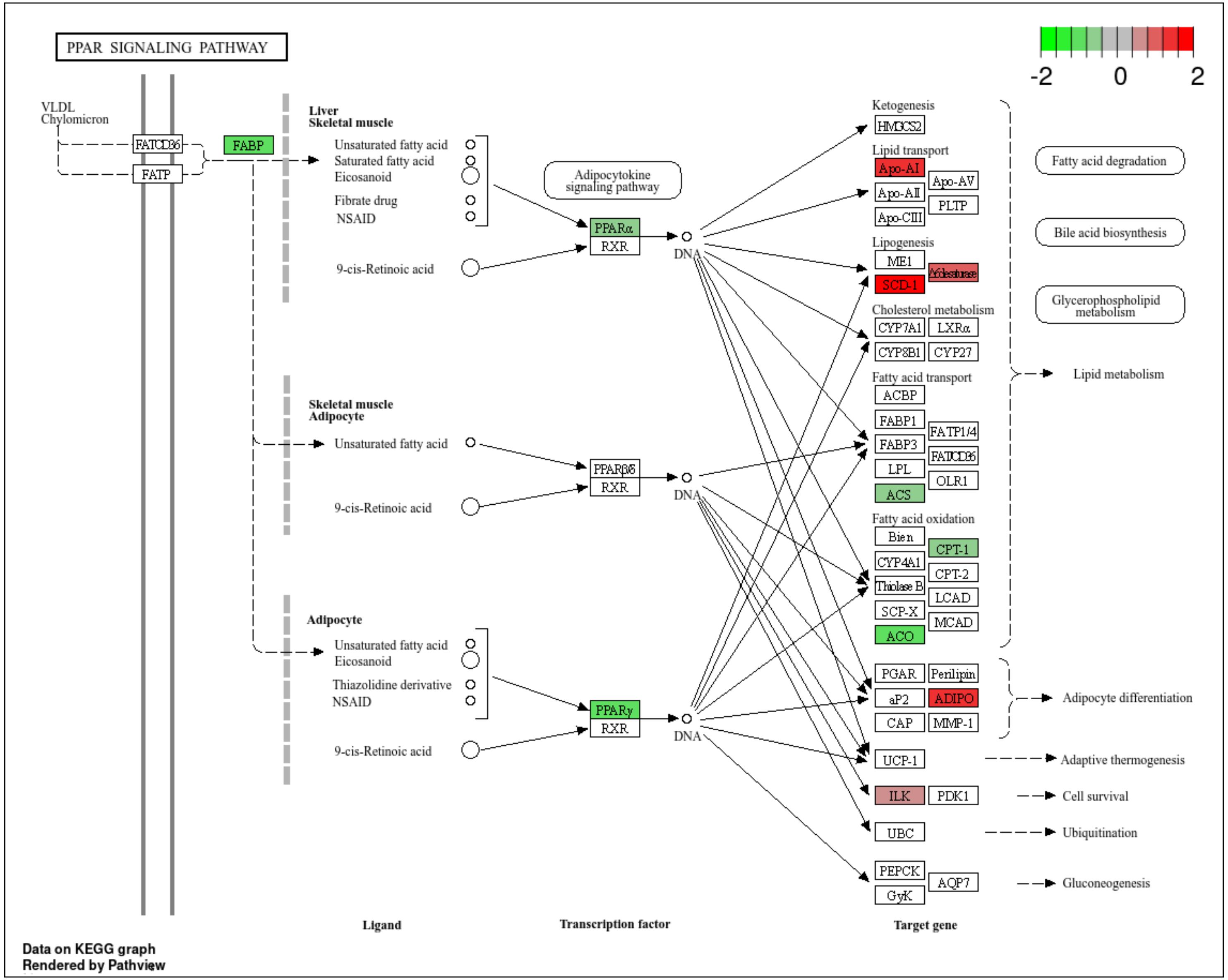

Figure S45. PPAR signaling pathway in SYNCHr group in F2 (Cecal mucosa).

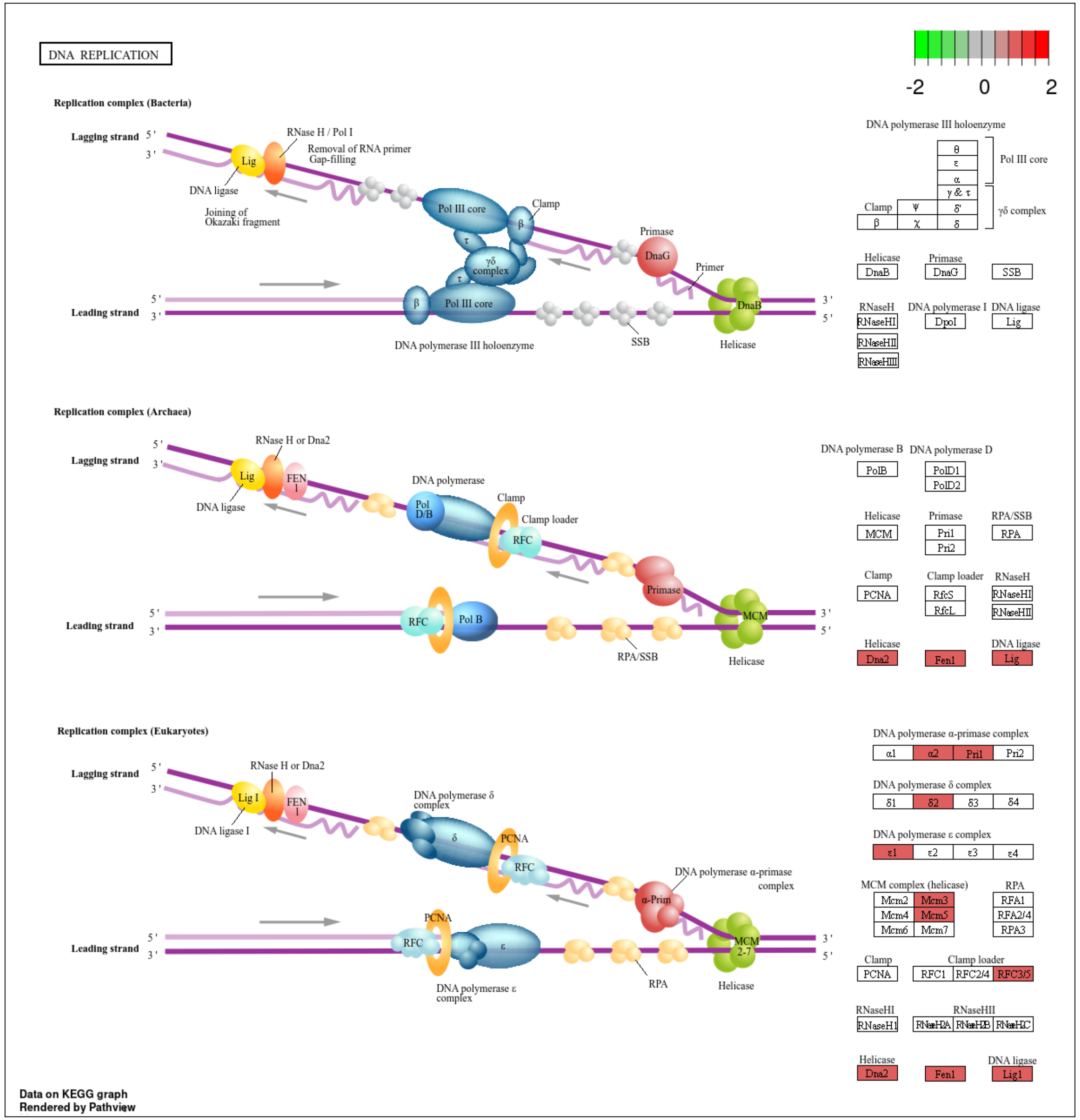

Figure S46. DNA replication pathway in SYNCHr group in F2 (Cecal mucosa).

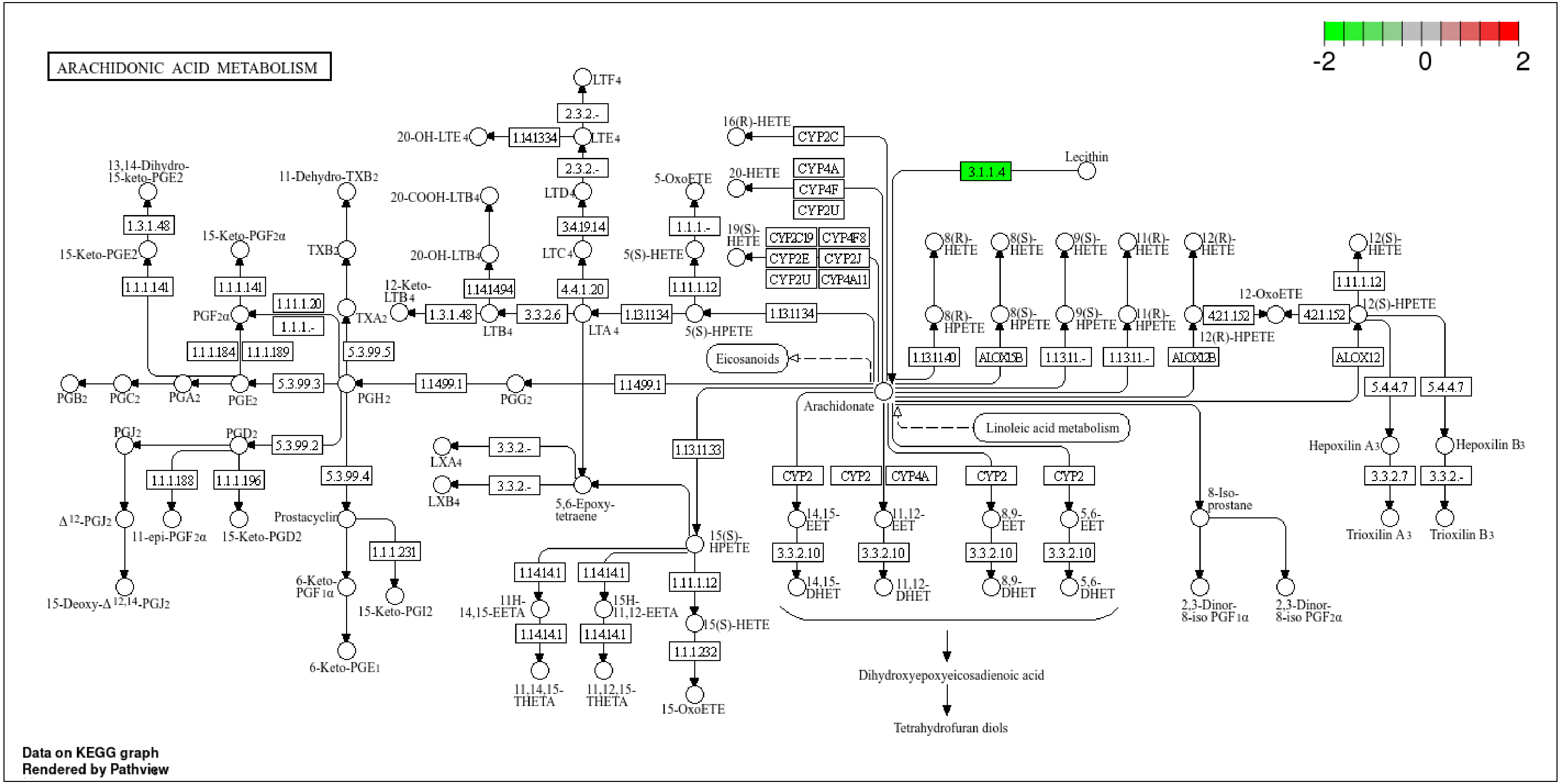

Figure S47. Arachidonic acid metabolism pathway in SYNs group in F3 (Cecal mucosa).

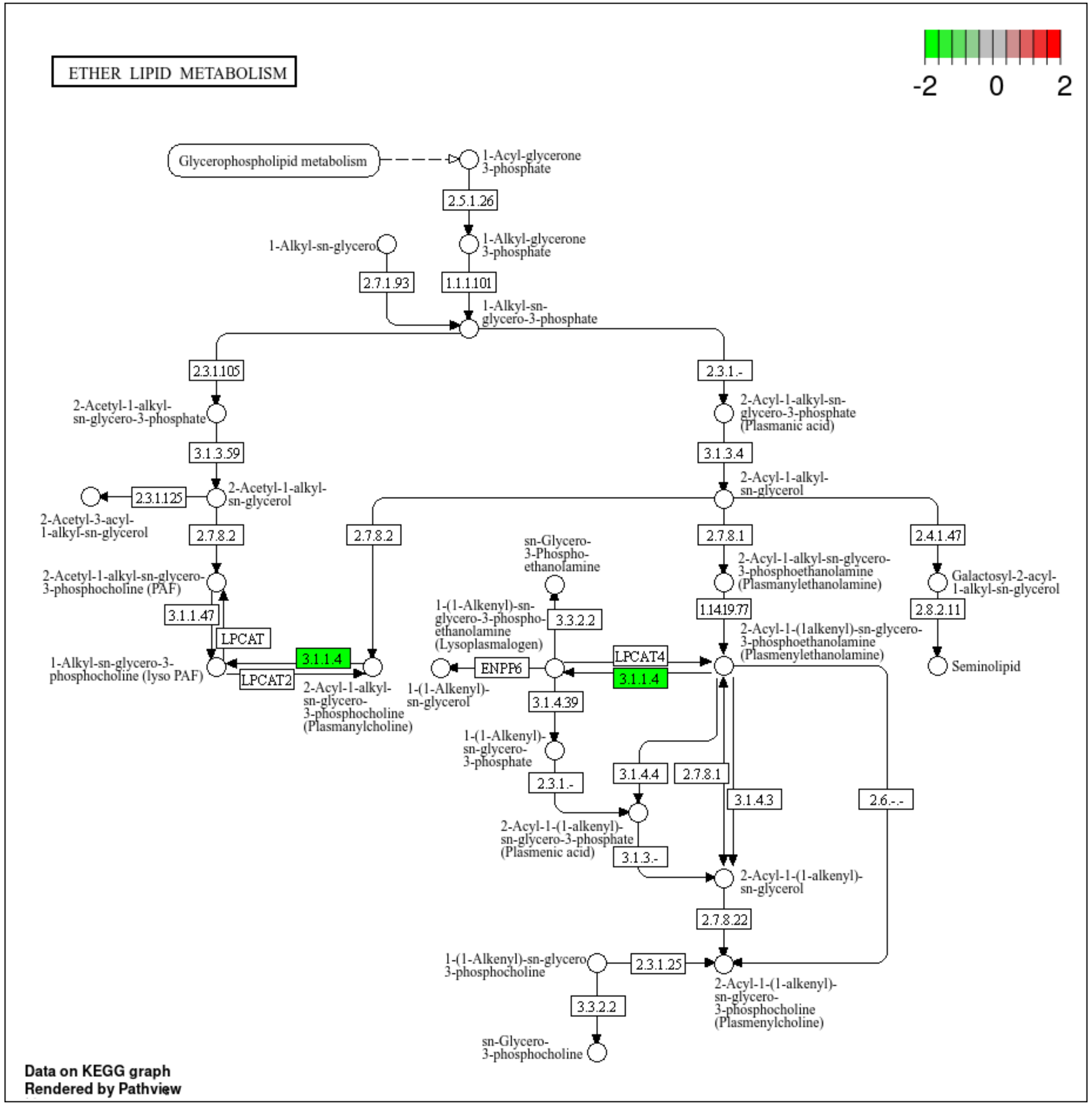

Figure S48. Ether lipid metabolism pathway in SYNs group in F3 (Cecal mucosa).

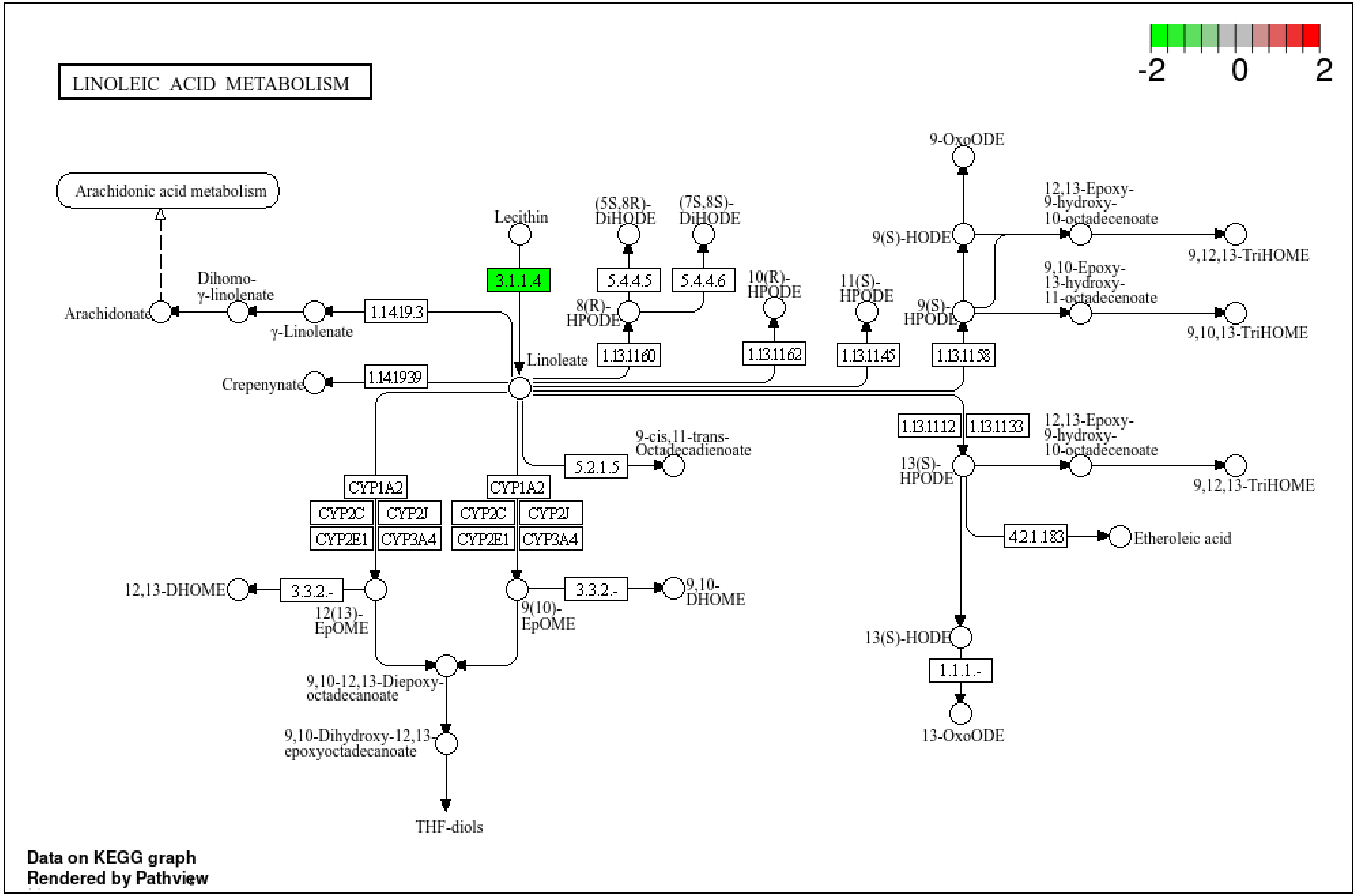

Figure S49. Linoleic acid metabolism pathway in SYNs group in F3 (Cecal mucosa).

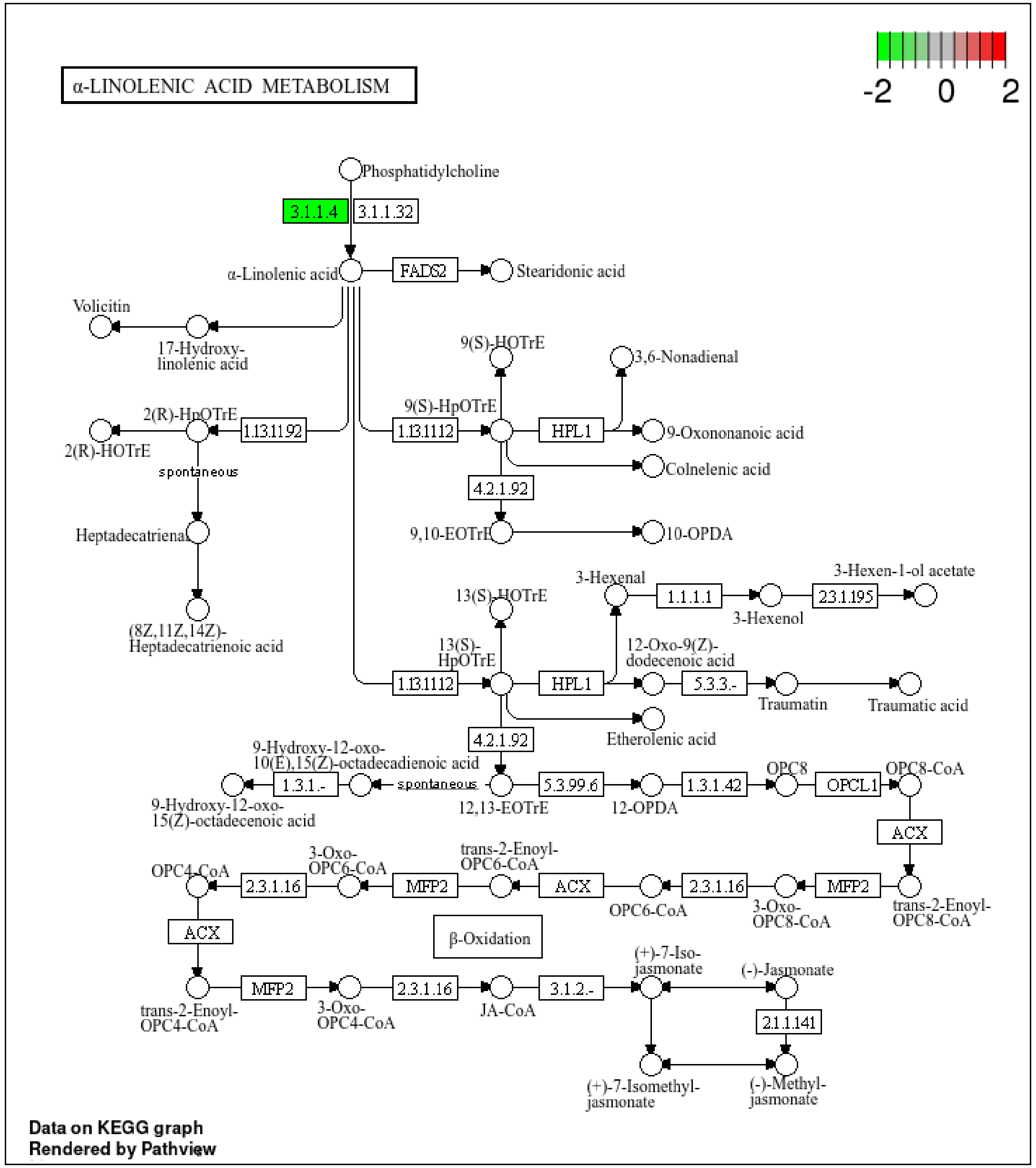

Figure S50. Alfa- Linoleic acid metabolism pathway in SYNs group in F3 (Cecal mucosa).

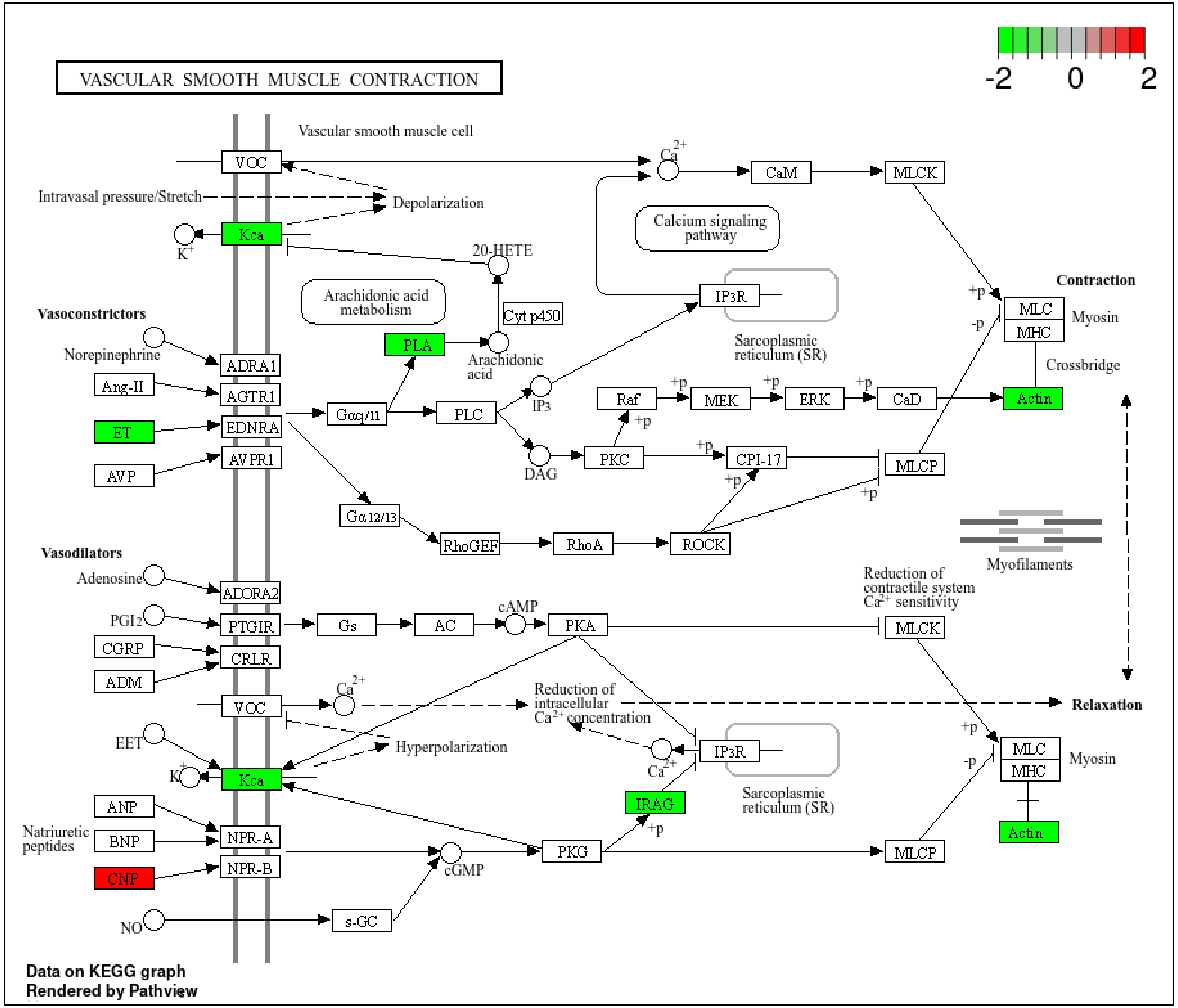

Figure S51. Vascular smooth muscle contraction pathway in SYNs group in F3 (Cecal mucosa).

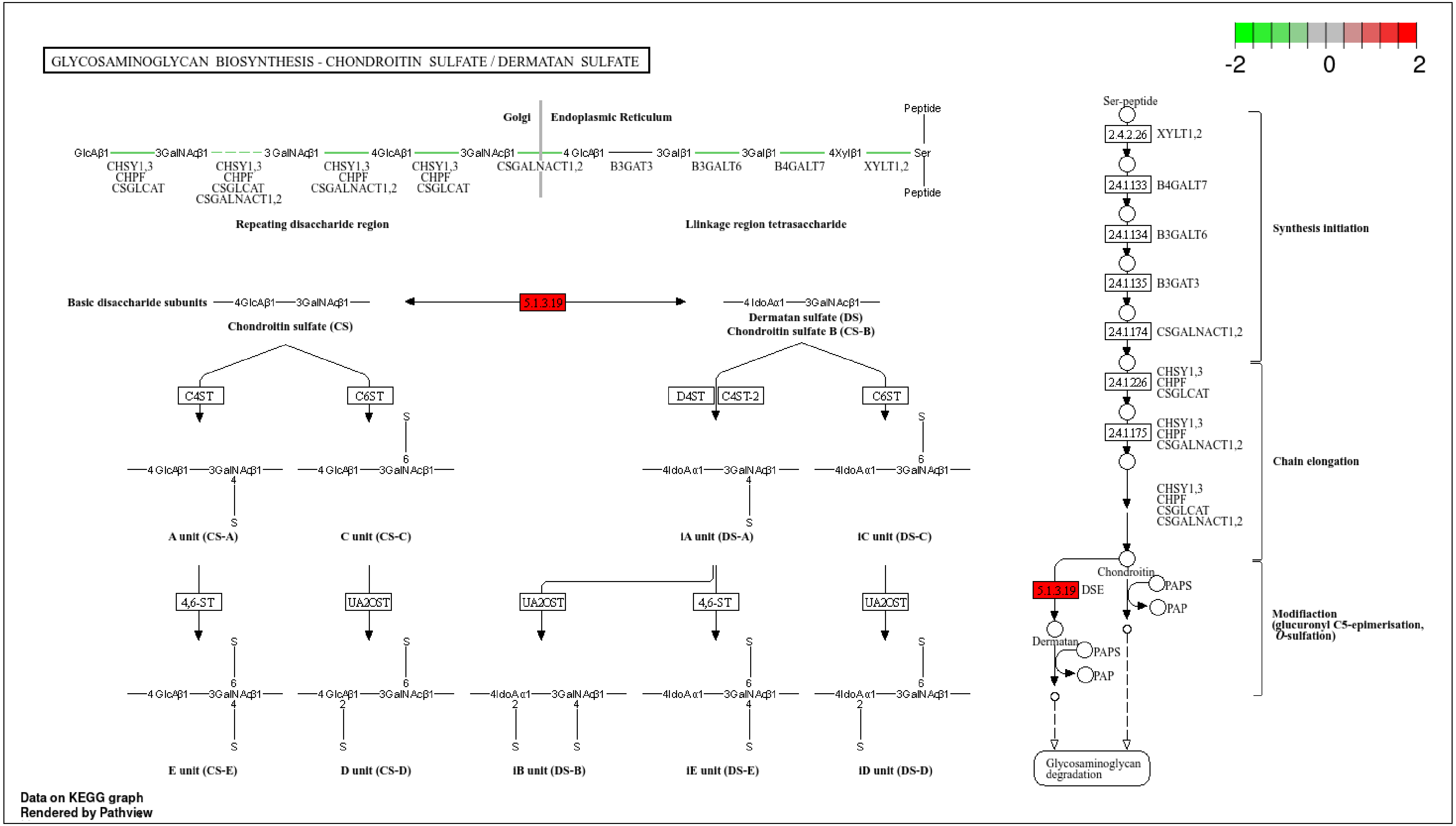

Figure S52. Glycosaminoglycan biosynthesis – chondroitin sulfate / dermatan sulfate pathway in SYNCHs group in F3 (Cecal mucosa).

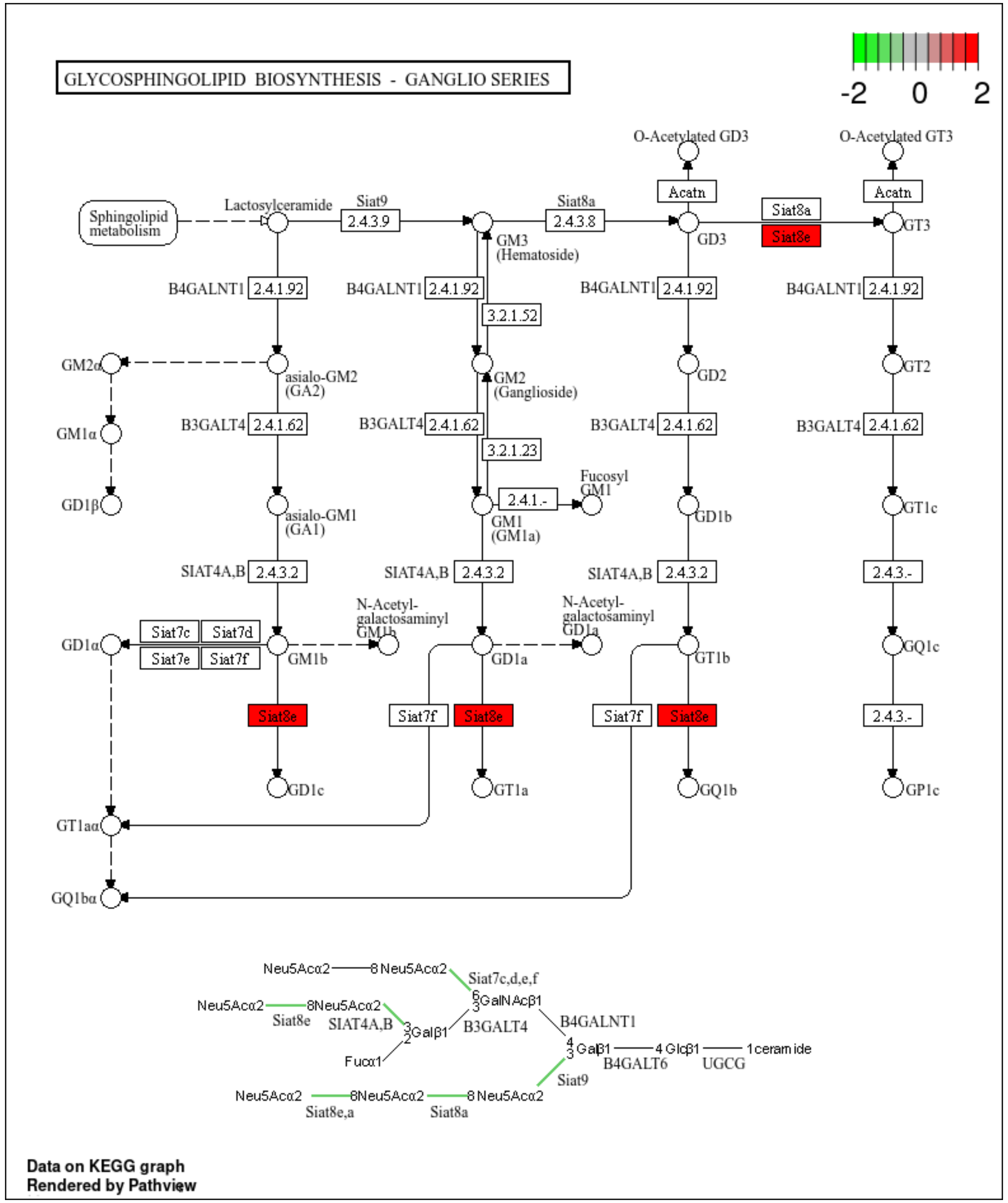

Figure S53. Glycosphingolipid biosynthesis – ganglio series pathway in SYNCHs group in F3 (Cecal mucosa).

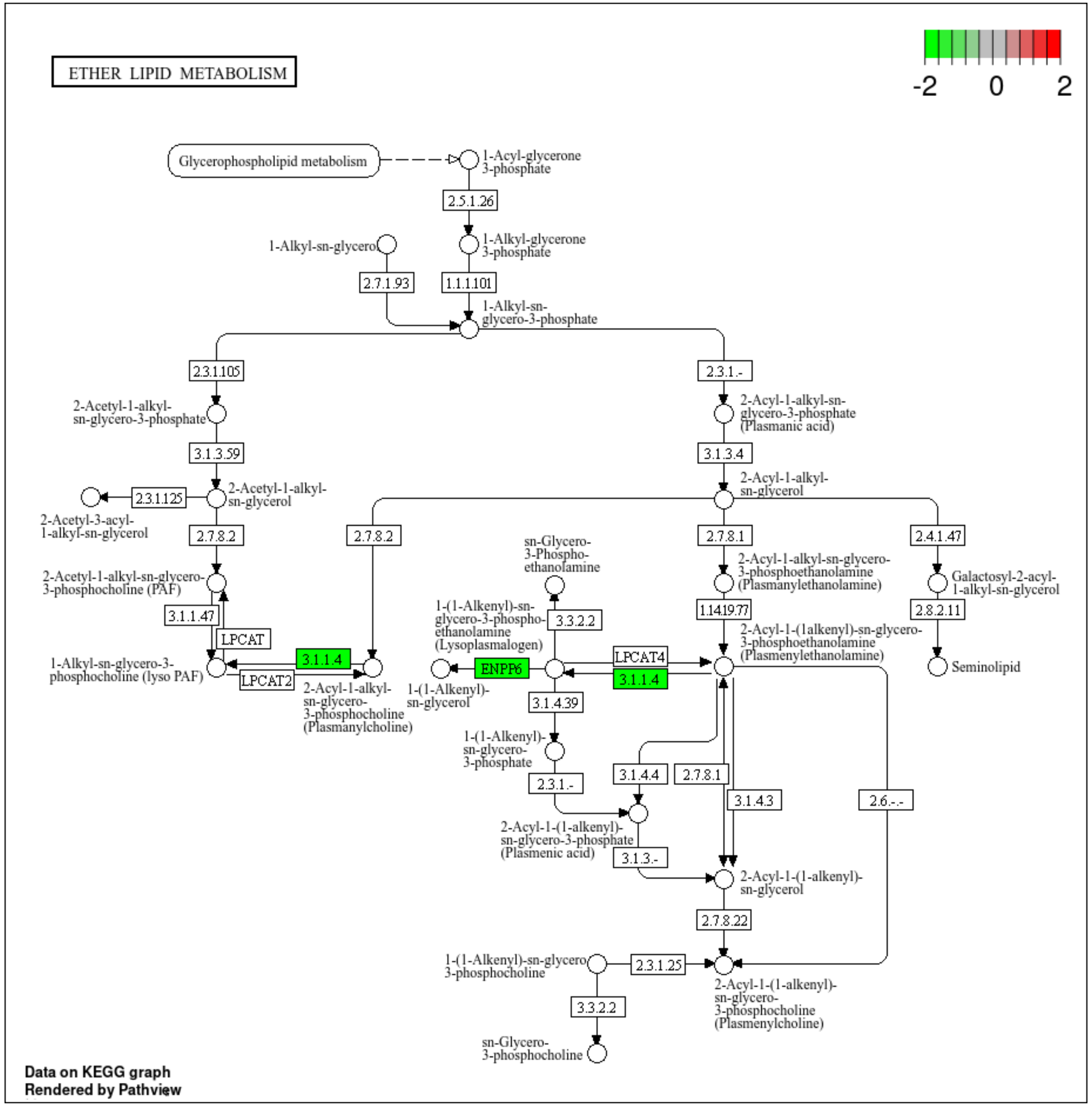

Figure S54. Ether lipid metabolism pathway in SYNCHs group in F3 (Cecal mucosa).

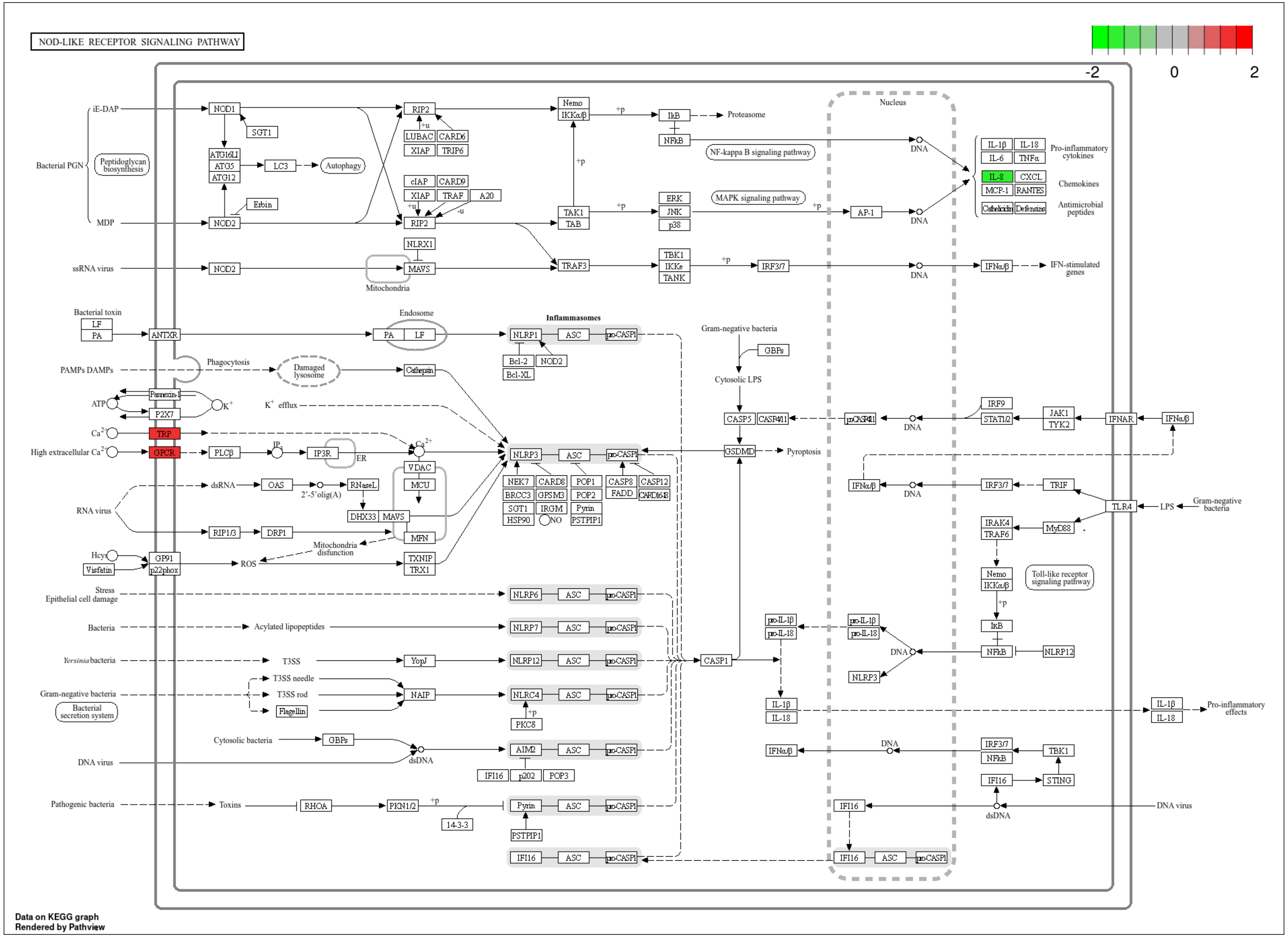

Figure S55. NOD-like receptor signaling pathway in SYNCHs group in F3 (Cecal mucosa).

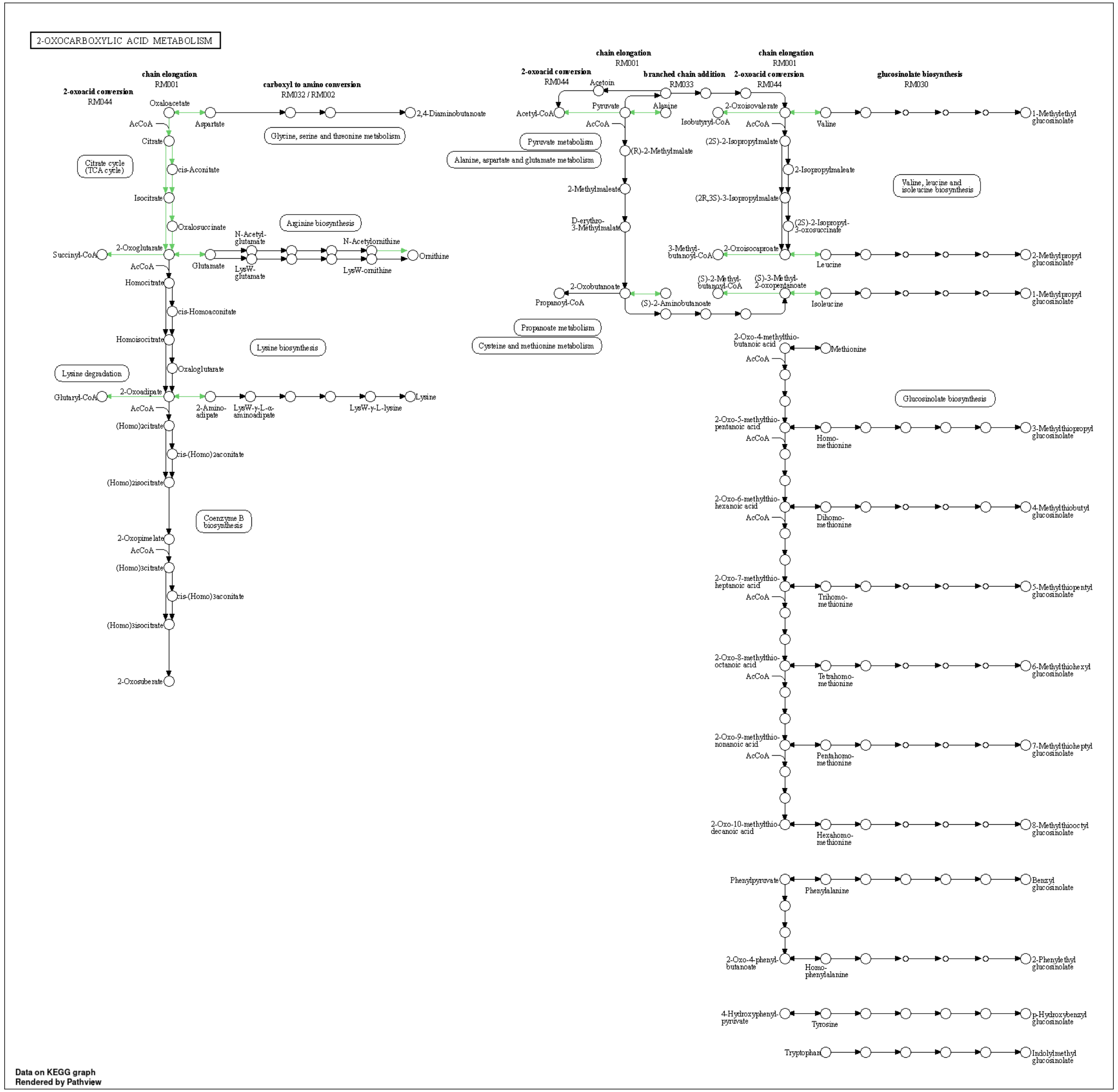

Figure S56. 2- Oxocarboxylic acid metabolism pathway in SYNCHr group in F3 (Cecal mucosa).

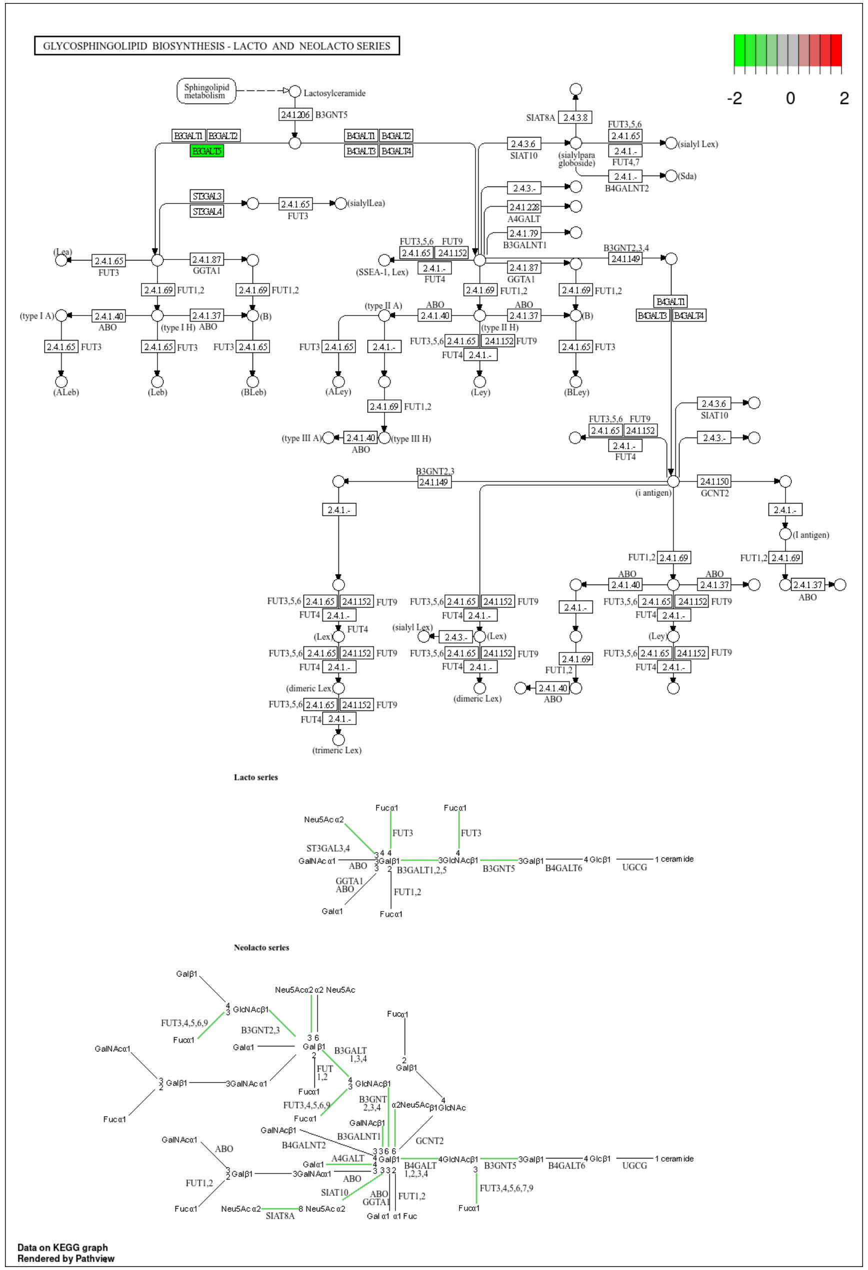

Figure S57. Glycosphingolipid biosynthesis – Lacto and neolacto series pathway in SYNCHr group in F3 (Cecal mucosa).

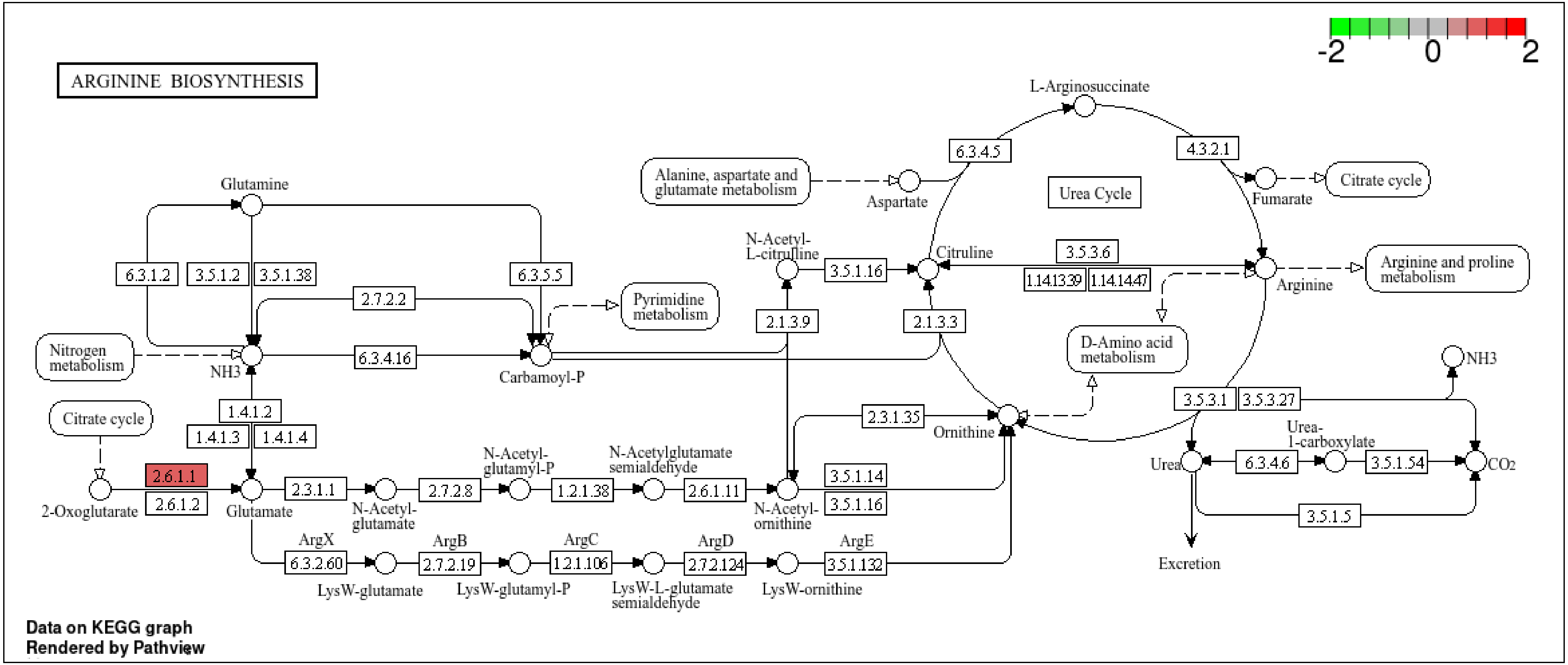

Figure S58. Arginine biosynthesis pathway in SYNCHr group in F3 (Cecal mucosa).

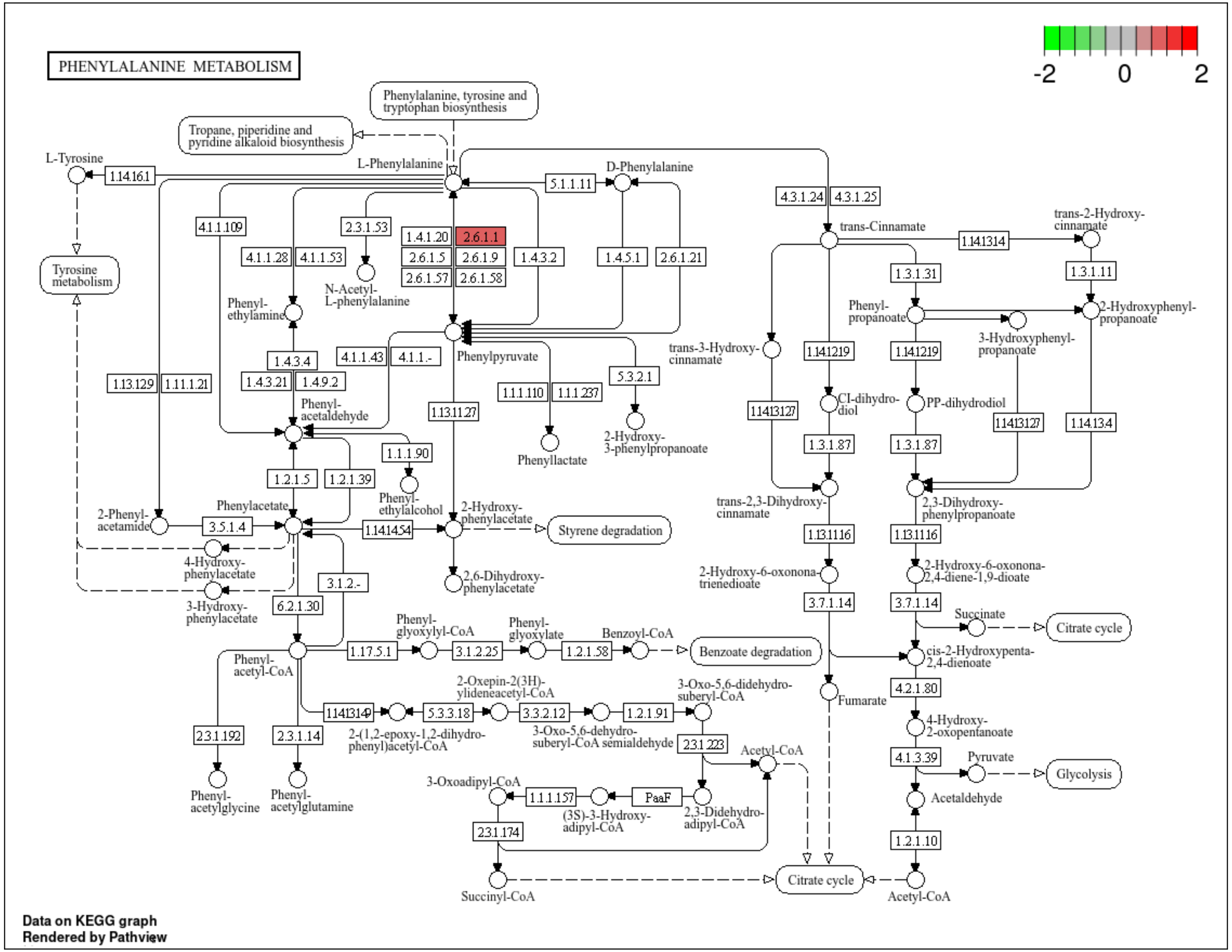

Figure S59. Phenylalanine metabolism pathway in SYNCHr group in F3 (Cecal mucosa).

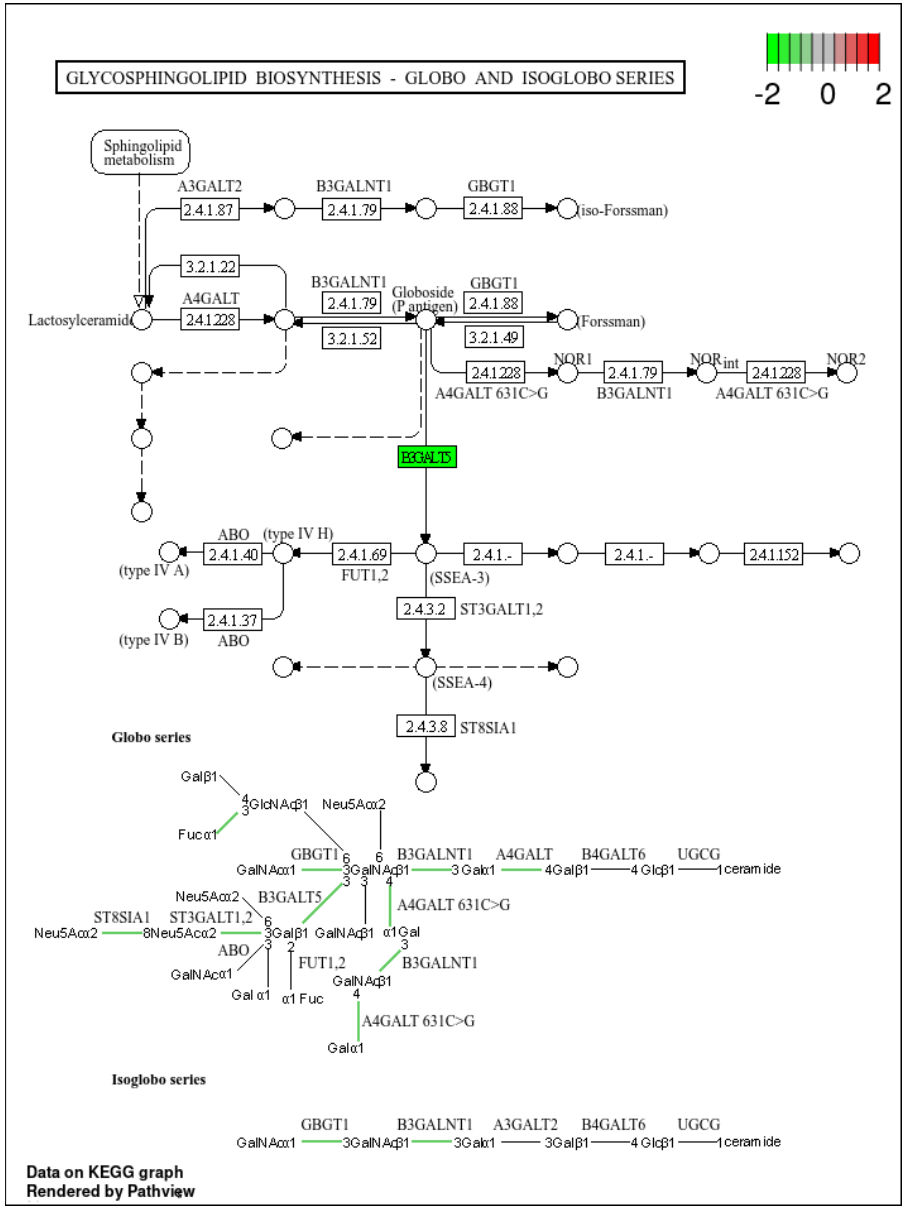

Figure S60. Glycosphingolipid biosynthesis – globo and isoglobo series pathway in SYNCHr group in F3 (Cecal mucosa).

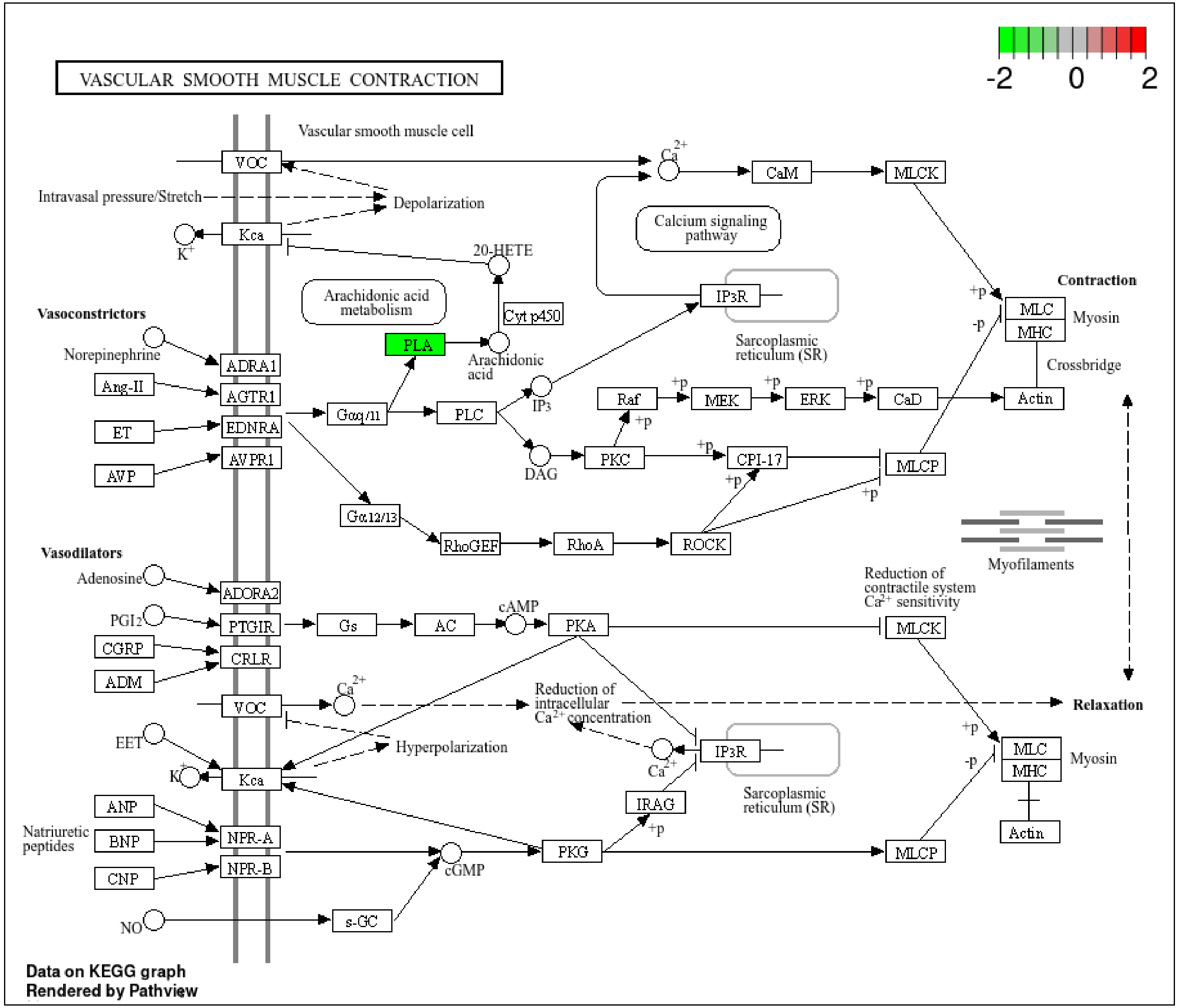

Figure S61. Vacular smooth muscle contraction pathway in SYNCHr group in F3 (Cecal mucosa).

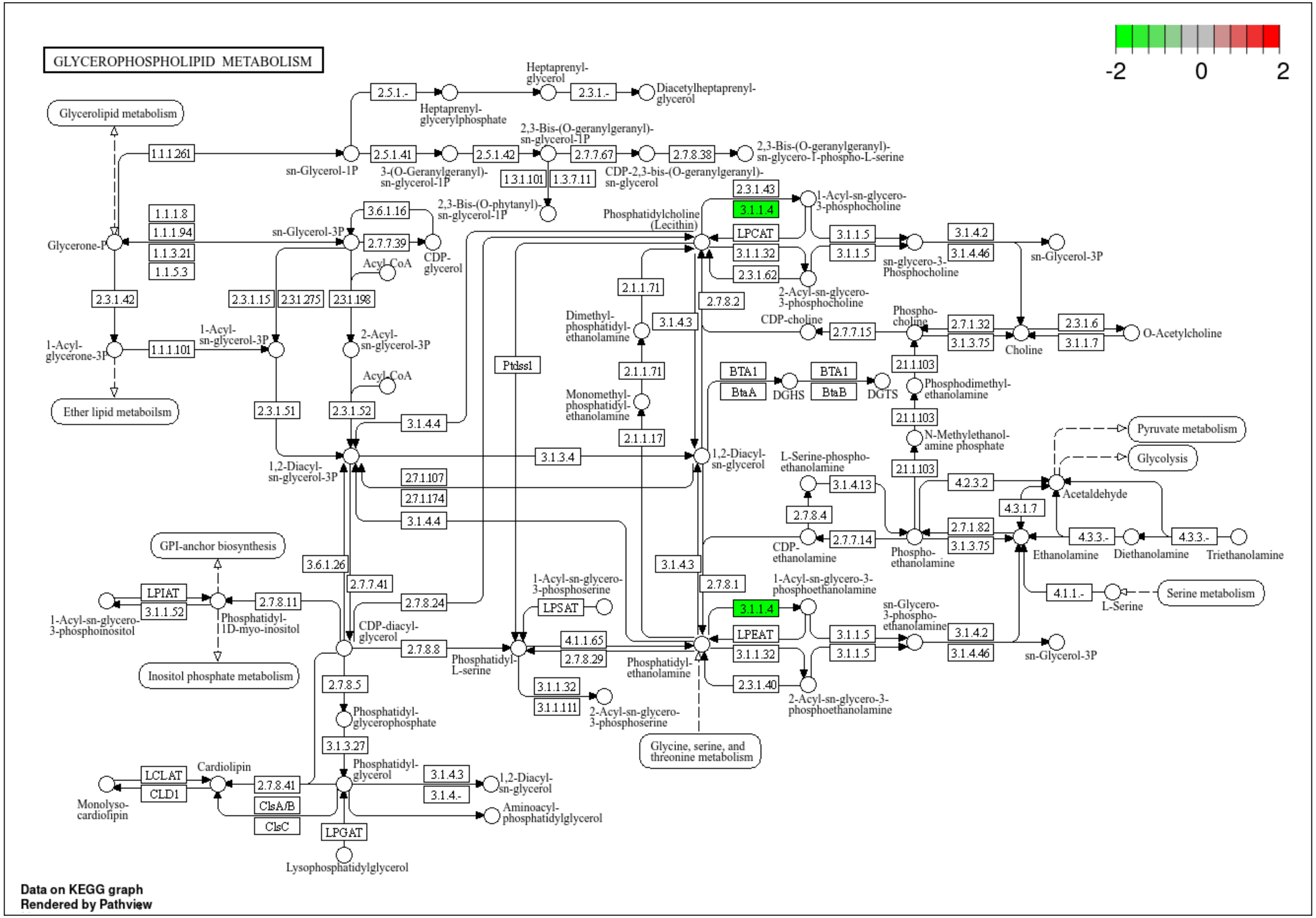

Figure S62. Glycerophospholipid metabolism pathway in SYNCHr group in F3 (Cecal mucosa).

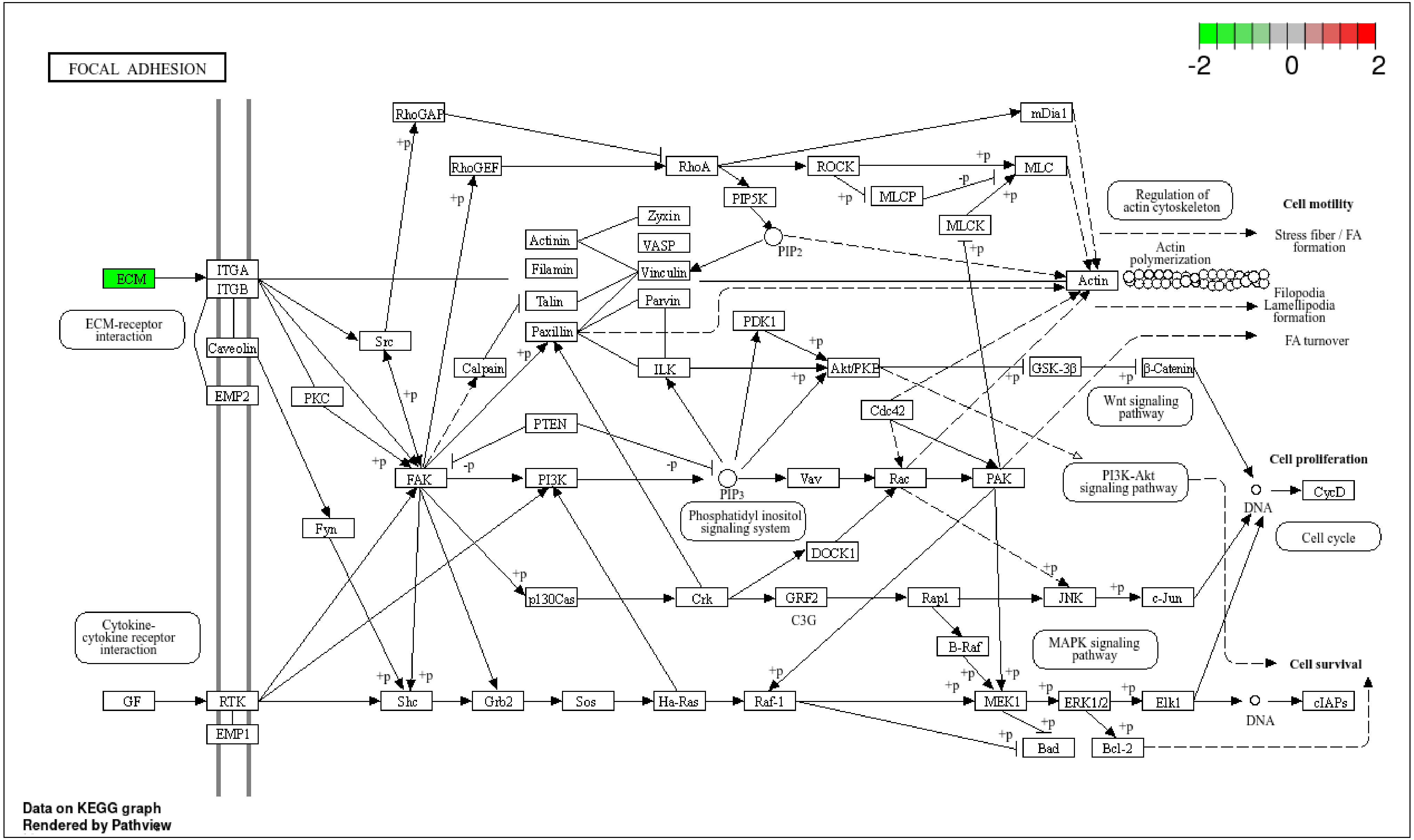

Figure S63. Focal adhesion pathway in SYNCHr group in F3 (Cecal mucosa).

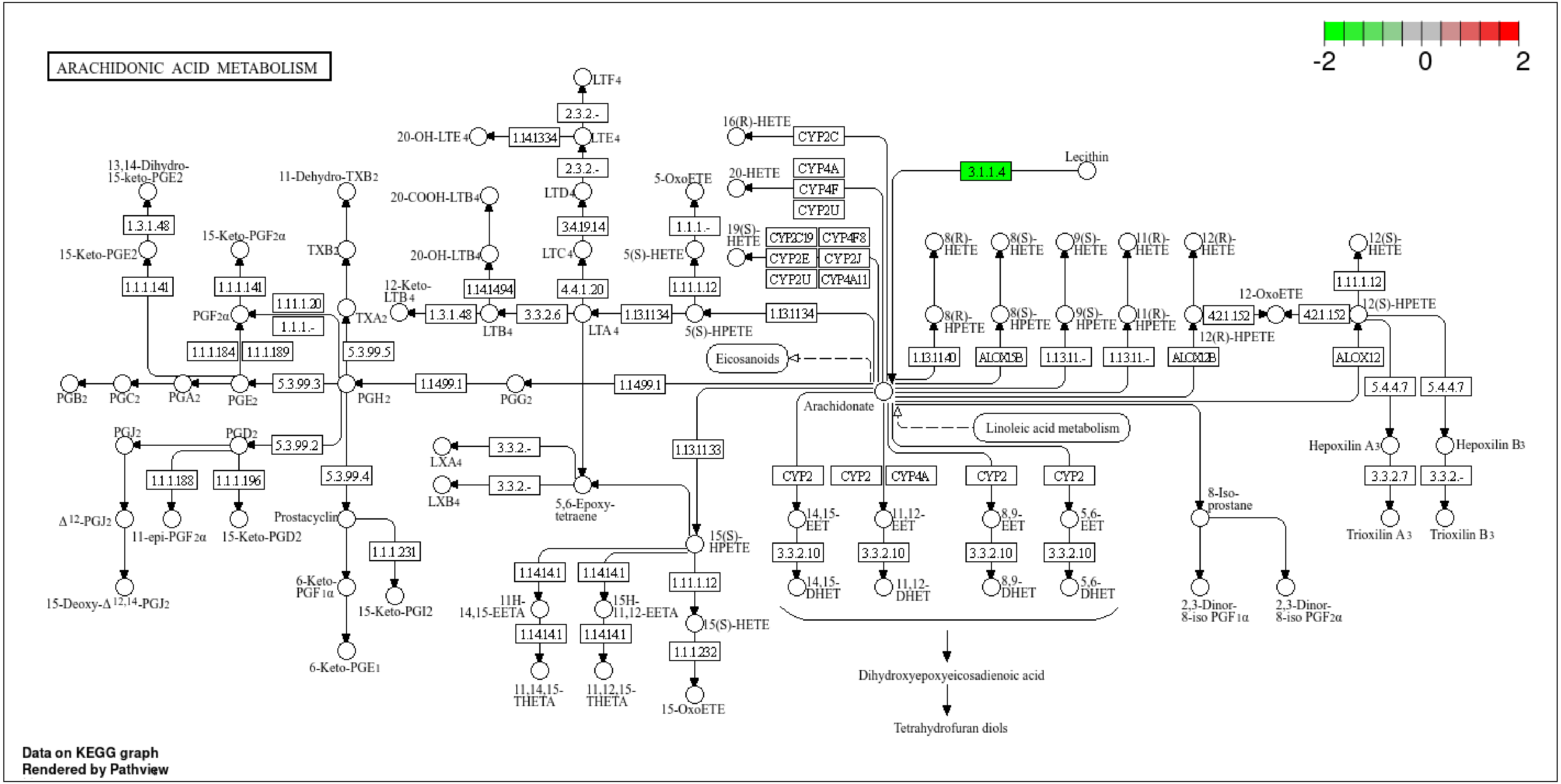

Figure S64. Arachidonic acid metabolism pathway in SYNCHr group in F3 (Cecal mucosa).

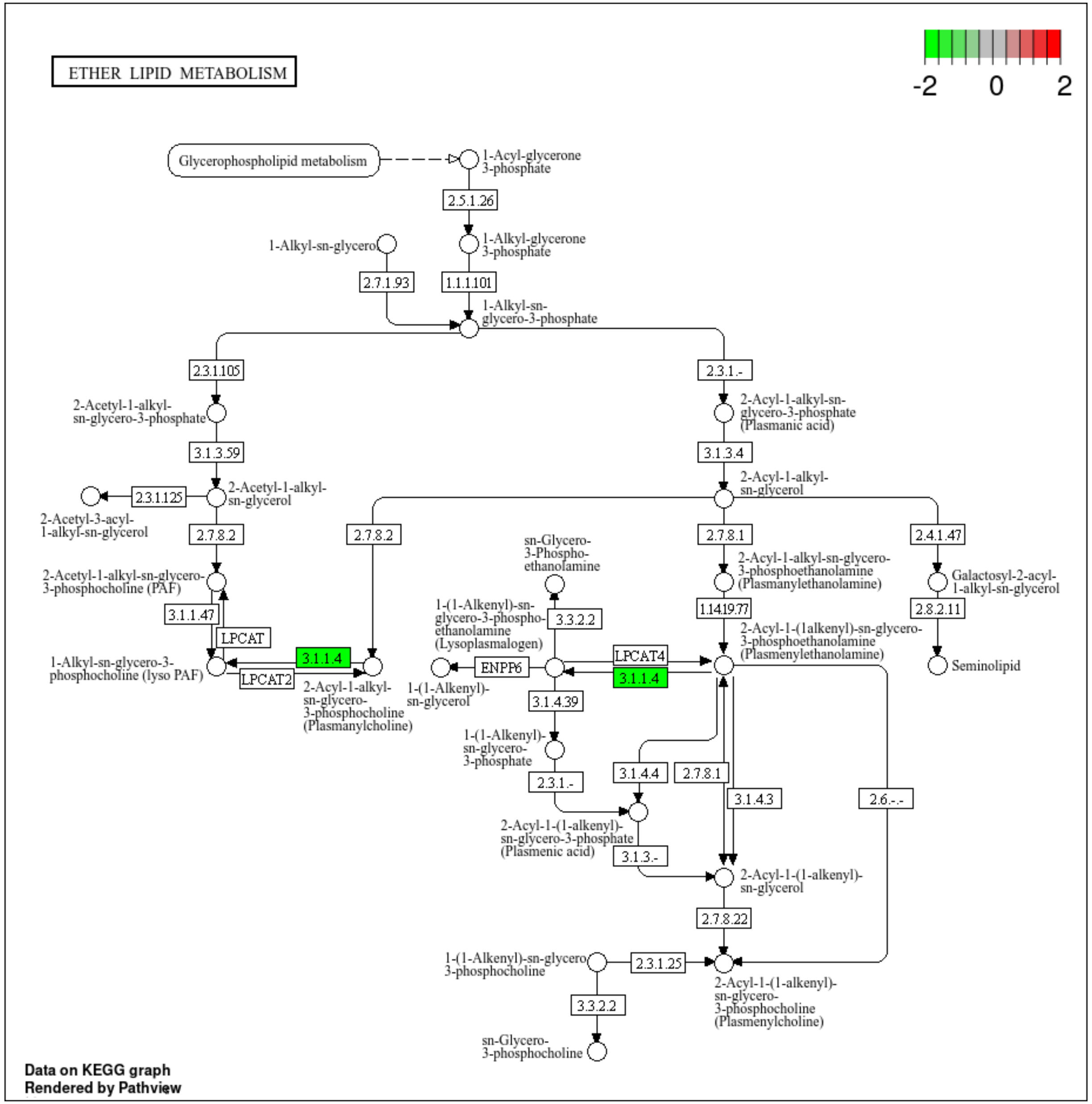

Figure S65. Ether lipid metabolism pathway in SYNCHr group in F3 (Cecal mucosa).

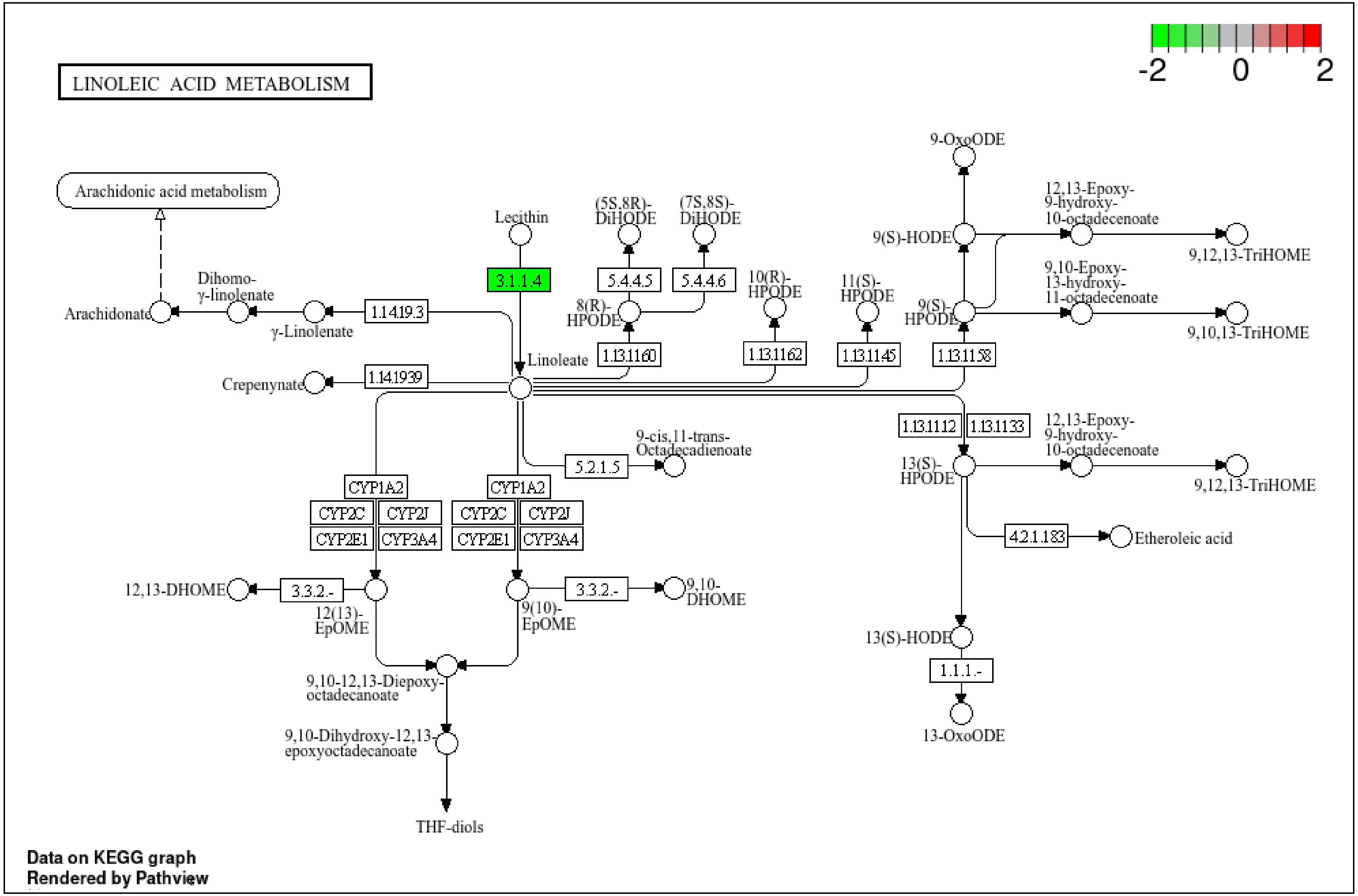

Figure S66. Linoleic acid metabolism pathway in SYNCHr group in F3 (Cecal mucosa).

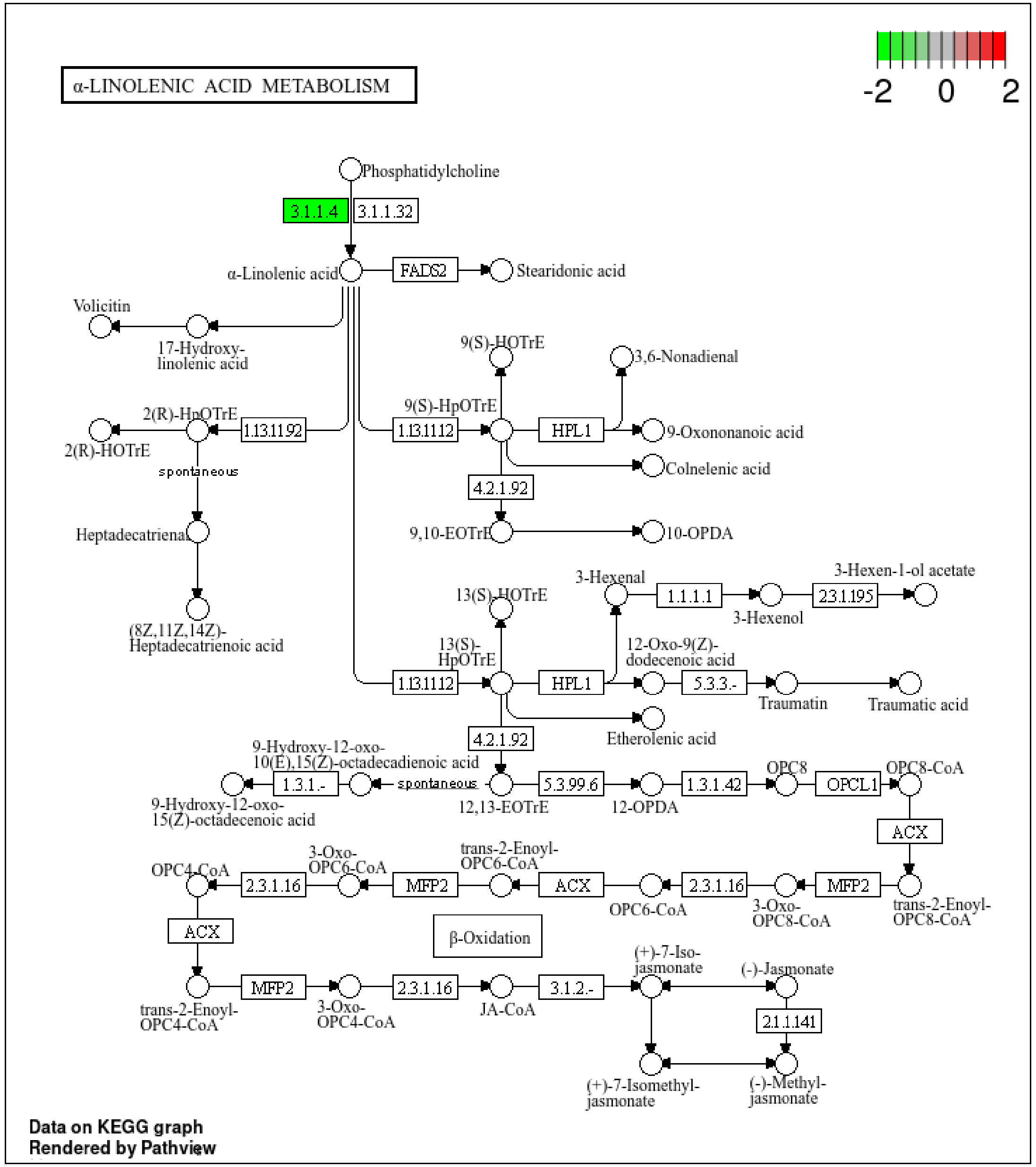

Figure S67. Alfa-Linoleic acid metabolism pathway in SYNCHr group in F3 (Cecal mucosa).

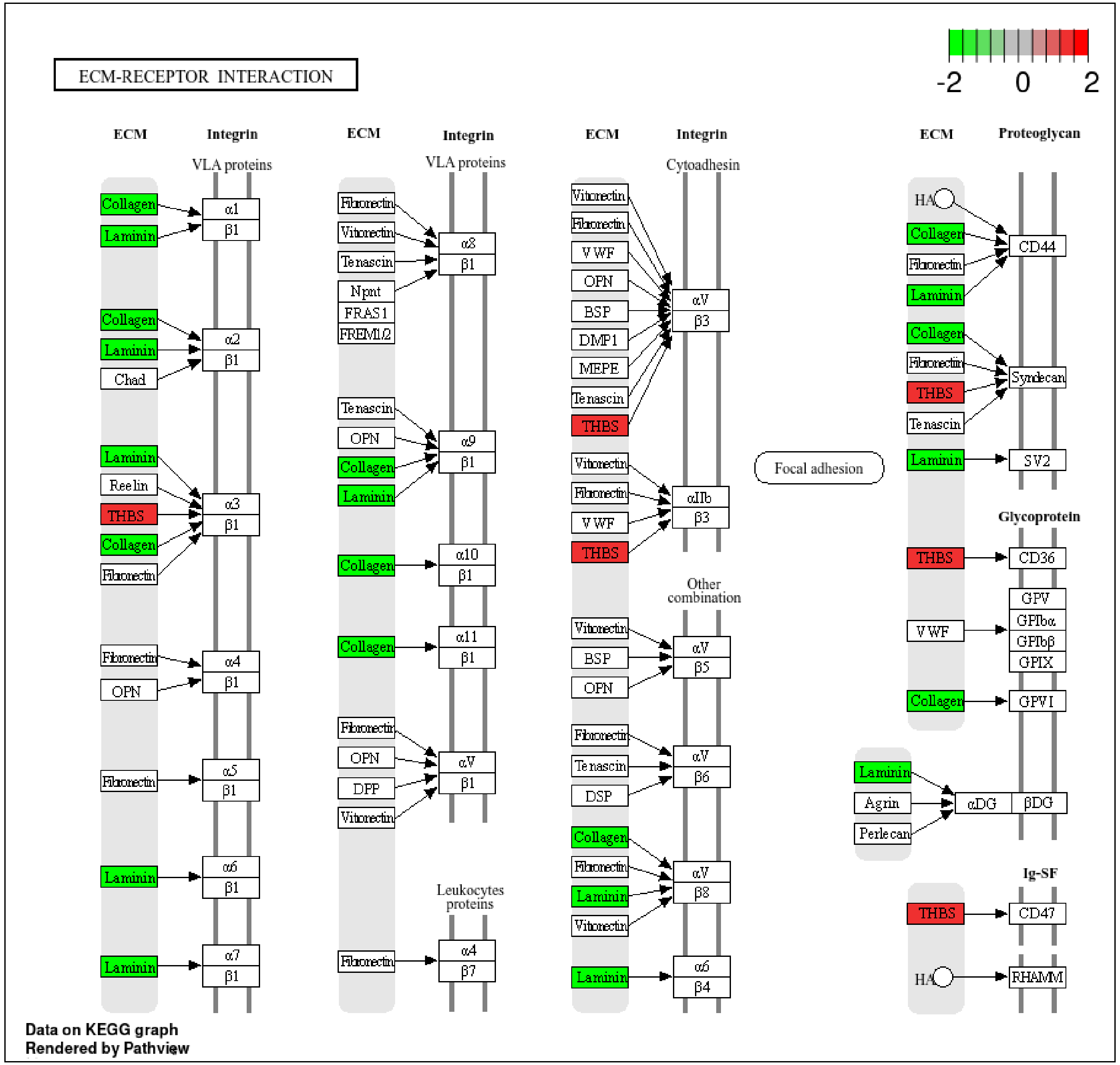

Figure S68. ECM- receptor interaction pathway in SYNCHr group in F3 (Cecal mucosa).
