# Supplementary material for: Inter- and Transgenerational Effects of In Ovo Stimulation with Bioactive Compounds on Cecal Tonsils and Cecal Mucosa Transcriptomes in a Chicken Model
Source: Int J Mol Sci. 2025 Jan 29;26(3):1174. doi: 10.3390/ijms26031174 (PMC11817890; doi:10.3390/ijms26031174)
Supplement: Supplementary file 1 [file ijms-26-01174-s001.zip › Supplementary file S3.pdf]

Analysis for Experiment 1:

Table S1. Two-way ANOVA results for hatchability based on choline source and dose.

ANOVA - Hatchability

Factor	Sum of Squares	df	Mean Square	F	p	η^2_p
Choline source	526.826	3	175.609	0.856	0.484	0.138
Choline dose	37.475	1	37.475	0.183	0.675	0.011
Choline source × choline dose	74.521	3	24.840	0.121	0.946	0.022
Residuals	3281.596	16	205.100			

Note. Type III Sum of Squares

Analysis for Experiment 2:

Table S2. Three-way ANOVA results for hatchability based on choline source, choline dose and synbiotic dose.

ANOVA - Hatchability

Factor	Sum of Squares	df	Mean Square	F	p	η^2_p
Choline source	1.815	1	1.815	0.094	0.763	0.006
Choline dose	39.015	1	39.015	2.022	0.174	0.112
Synbiotic dose	0.282	1	0.282	0.015	0.905	9.114×10^{-4}
Choline source × choline dose	1.707	1	1.707	0.088	0.770	0.005
Choline source × synbiotic dose	179.307	1	179.307	9.291	0.008	0.367
Choline dose × synbiotic dose	164.327	1	164.327	8.515	0.010	0.347
Choline source × choline dose × synbiotic dose	29.482	1	29.482	1.528	0.234	0.087
Residuals	308.780	16	19.299			

Note. Type III Sum of Squares

Table S3. Post hoc comparisons for the interaction between choline source and synbiotic dose on hatchability.

Post hoc comparisons - choline source × synbiotic dose

Comparison		Mean Difference	95% CI for Mean Difference		SE	df	t	p_{tukey}
			Lower	Upper				
Miavit 1 mg	Sigma Alrich 1 mg	6.017	-1.240	13.273	2.536	16	2.372	0.123
	Miavit 2 mg	5.250	-2.006	12.506	2.536	16	2.070	0.205
	Sigma Alrich 2 mg	0.333	-6.923	7.590	2.536	16	0.131	0.999
Sigma Alrich 1 mg	Miavit 2 mg	-0.767	-8.023	6.490	2.536	16	-0.302	0.990
	Sigma Alrich 2 mg	-5.683	-12.940	1.573	2.536	16	-2.241	0.154
Miavit 2 mg	Sigma Alrich 2 mg	-4.917	-12.173	2.340	2.536	16	-1.939	0.252

Note. P-value and confidence intervals adjusted for comparing a family of 4 estimates (confidence intervals corrected using the Tukey method).

Note. Results are averaged over the levels of: choline dose

The choline chloride from Sigma Aldrich used here is the one with cat. no. (C7527)

Table S4. Post hoc comparisons for the interaction between choline dose and synbiotic dose on hatchability.

Post hoc comparisons - choline dose × synbiotic dose

Comparison		Mean Difference	95% CI for Mean Difference Lower	95% CI for Mean Difference Upper	SE	df	t	p_{tukey}
0.25 mg choline 1 mg synbiotic	0.5 mg choline 1 mg synbiotic	-2.683	-9.940	4.573	2.536	16	-1.058	0.719
	0.25 mg choline 2 mg synbiotic	-5.450	-12.706	1.806	2.536	16	-2.149	0.180
	0.5 mg choline 2 mg synbiotic	2.333	-4.923	9.590	2.536	16	0.920	0.795
0.5 mg choline 1 mg synbiotic	0.25 mg choline 2 mg synbiotic	-2.767	-10.023	4.490	2.536	16	-1.091	0.700
	0.5 mg choline 2 mg synbiotic	5.017	-2.240	12.273	2.536	16	1.978	0.237
0.25 mg choline 2 mg synbiotic	0.5 mg choline 2 mg synbiotic	7.783	0.527	15.040	2.536	16	3.069	0.033

Note. P-value and confidence intervals adjusted for comparing a family of 4 estimates (confidence intervals corrected using the Tukey method).

Note. Results are averaged over the levels of: Choline source
