# Supplementary material for: Inter- and Transgenerational Effects of In Ovo Stimulation with Bioactive Compounds on Cecal Tonsils and Cecal Mucosa Transcriptomes in a Chicken Model
Source: Int J Mol Sci. 2025 Jan 29;26(3):1174. doi: 10.3390/ijms26031174 (PMC11817890; doi:10.3390/ijms26031174)
Supplement: Supplementary file 1 [file ijms-26-01174-s001.zip › Supplementary file S5.pdf]

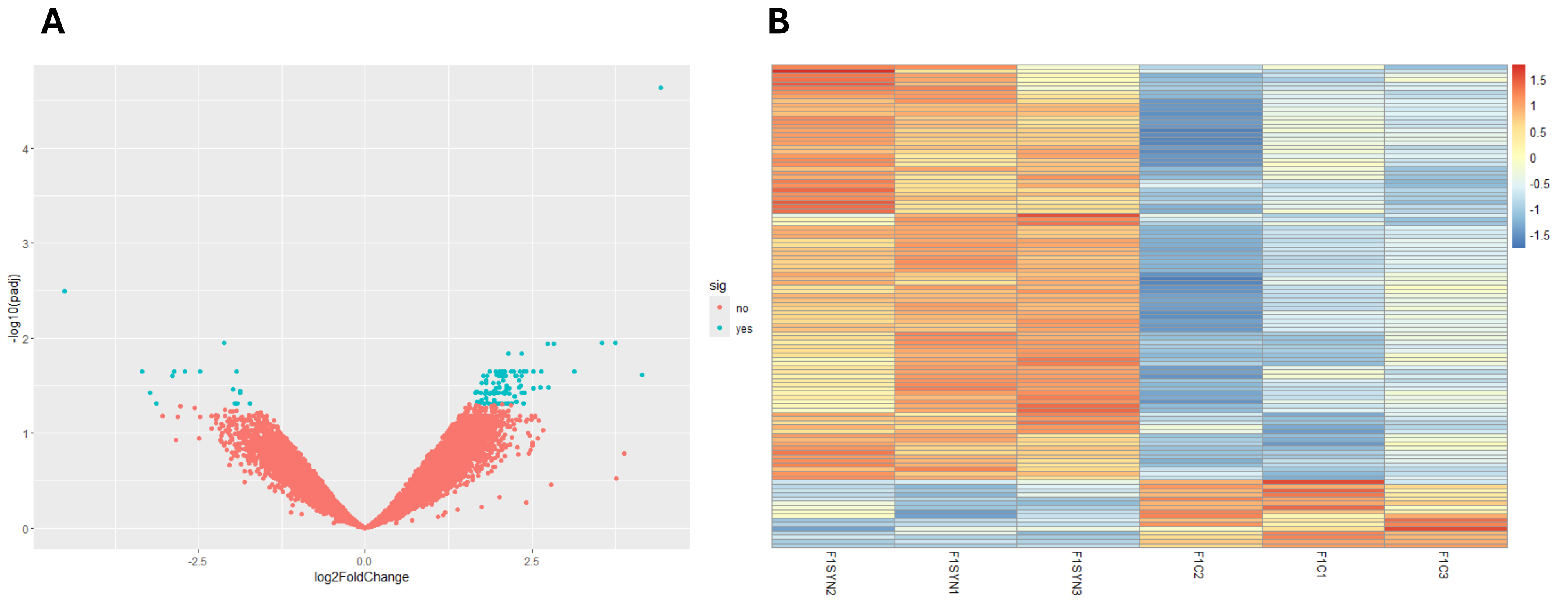

Figure S1: Differential expression gene (DEG) profiles in cecal mucosa of F1: SYN group vs control (F1SYN vs F1C). (A) volcano plots, (B) heatmap.

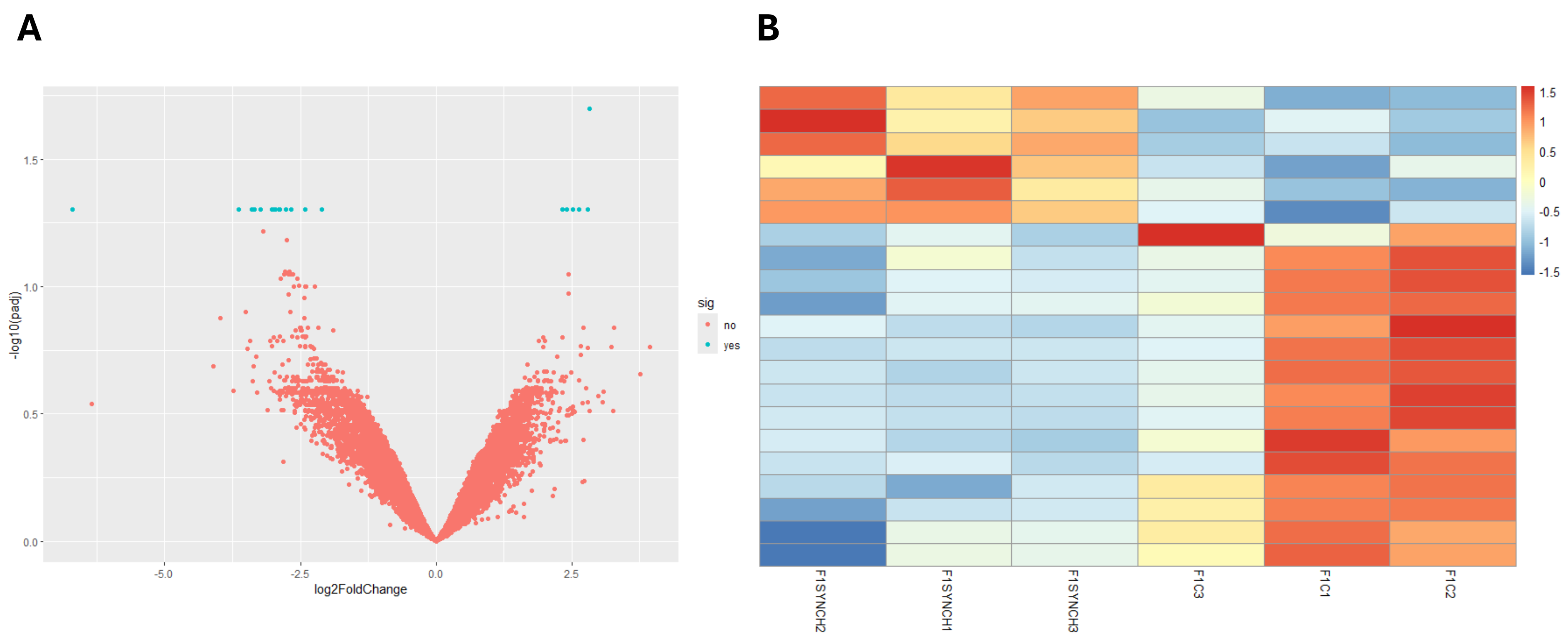

Figure S2: Differential expression gene (DEG) profiles in cecal mucosa of F1: SYNCH group vs control (F1SYNCH vs F1C). (A) volcano plots, (B) heatmap.

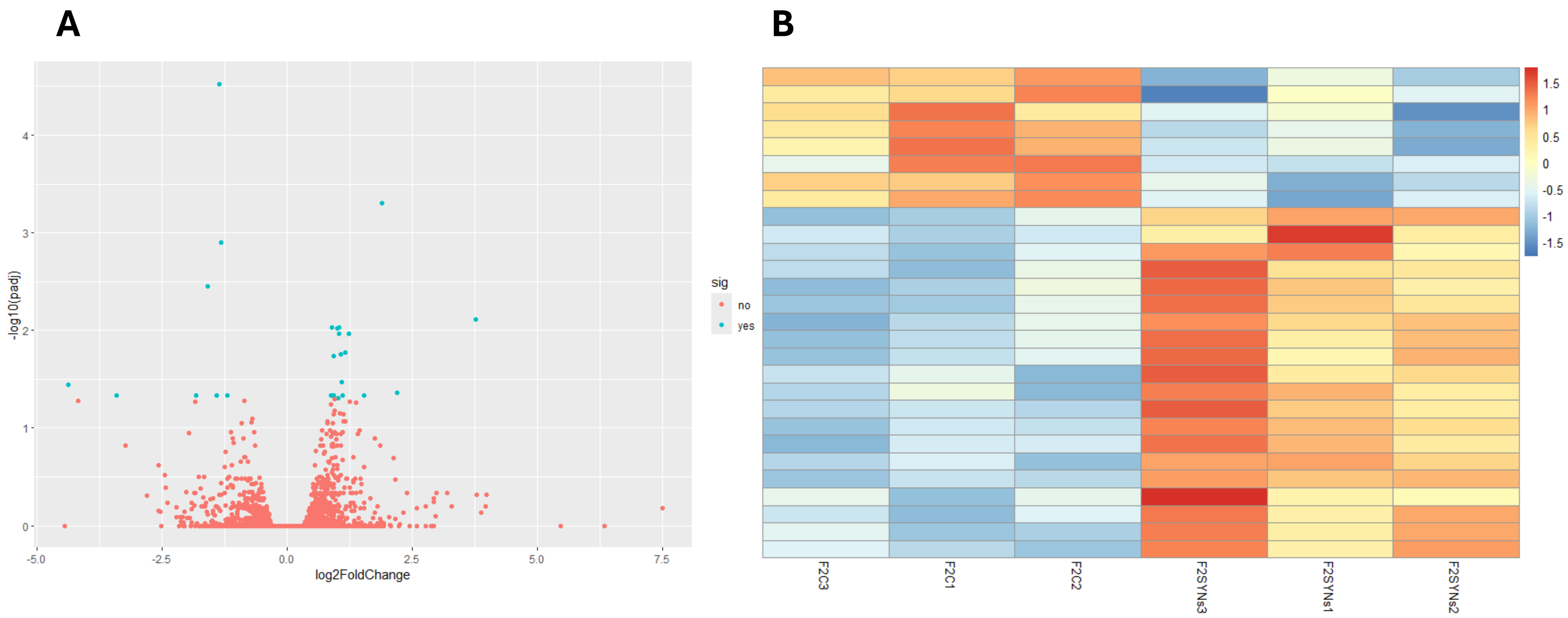

Figure S3: Differential expression gene (DEG) profiles in cecal mucosa of F2: SYNs group vs control (F2SYNs vs F2C). (A) volcano plots, (B) heatmap.

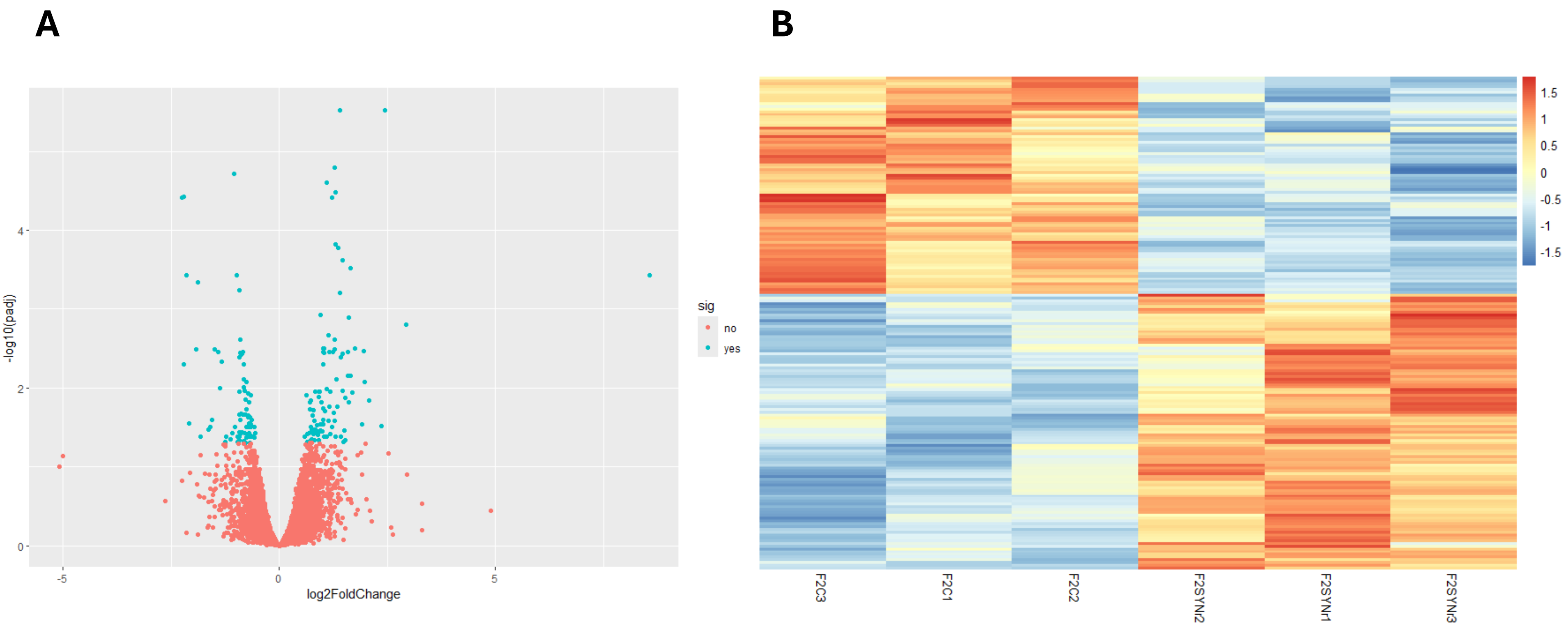

Figure S4: Differential expression gene (DEG) profiles in cecal mucosa of F2: SYNr group vs control (F2SYNr vs F2C). (A) volcano plots, (B) heatmap.

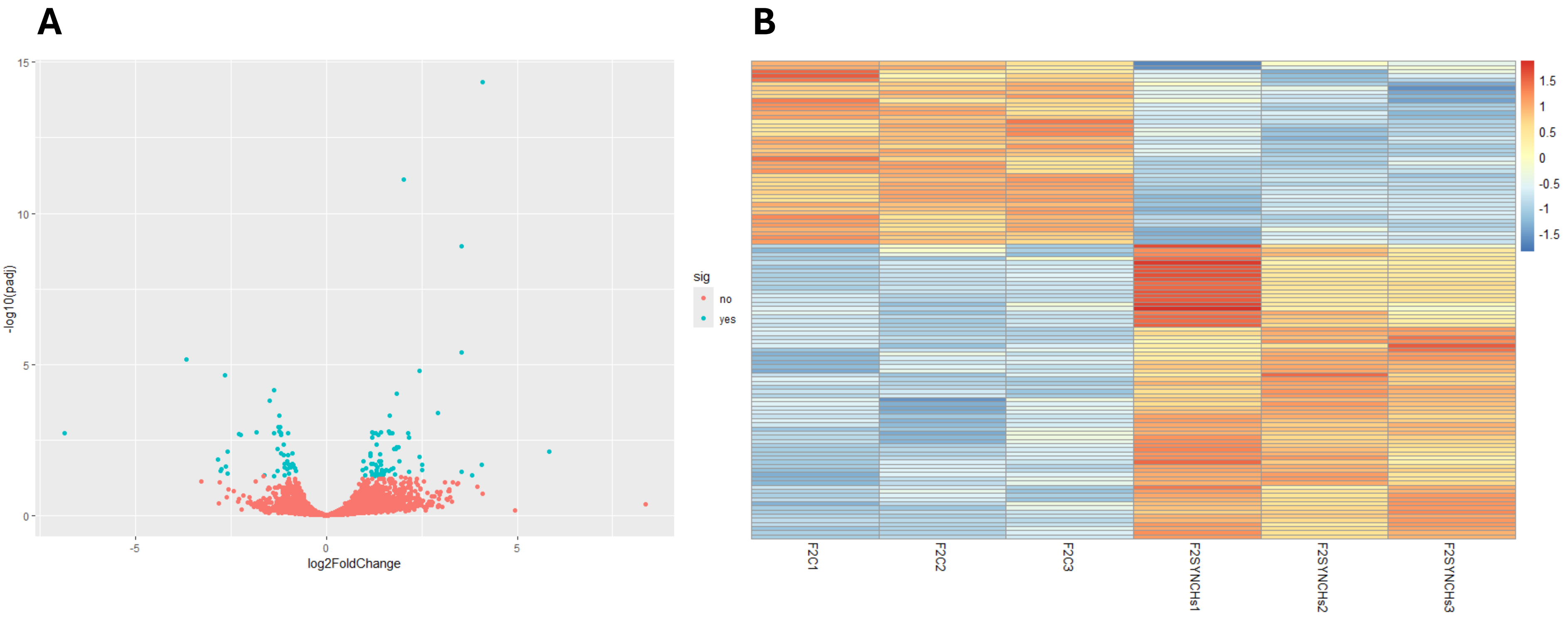

Figure S5: Differential expression gene (DEG) profiles in cecal mucosa of F2: SYNCHs group vs control (F2SYNCHs vs F2C). (A) volcano plots, (B) heatmap.

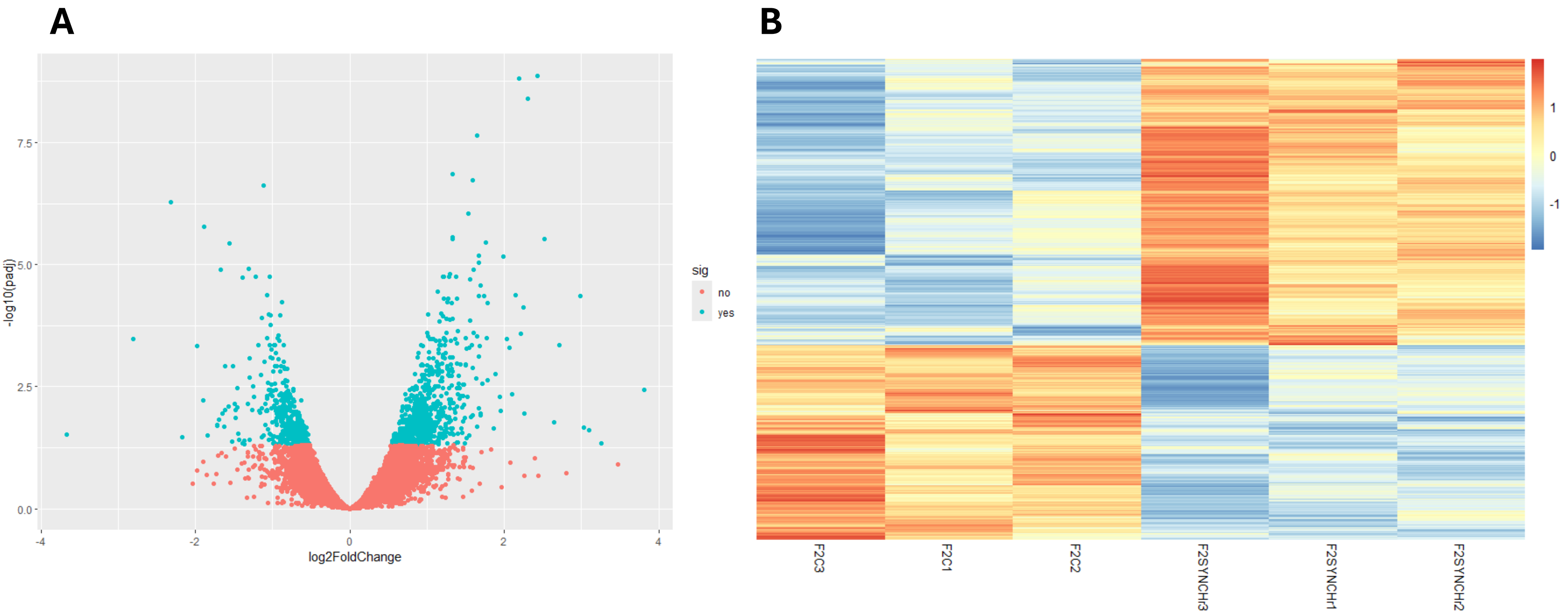

Figure S6: Differential expression gene (DEG) profiles in cecal mucosa of F2: SYNCHr group vs control (F2SYNCHr vs F2C). (A) volcano plots, (B) heatmap.

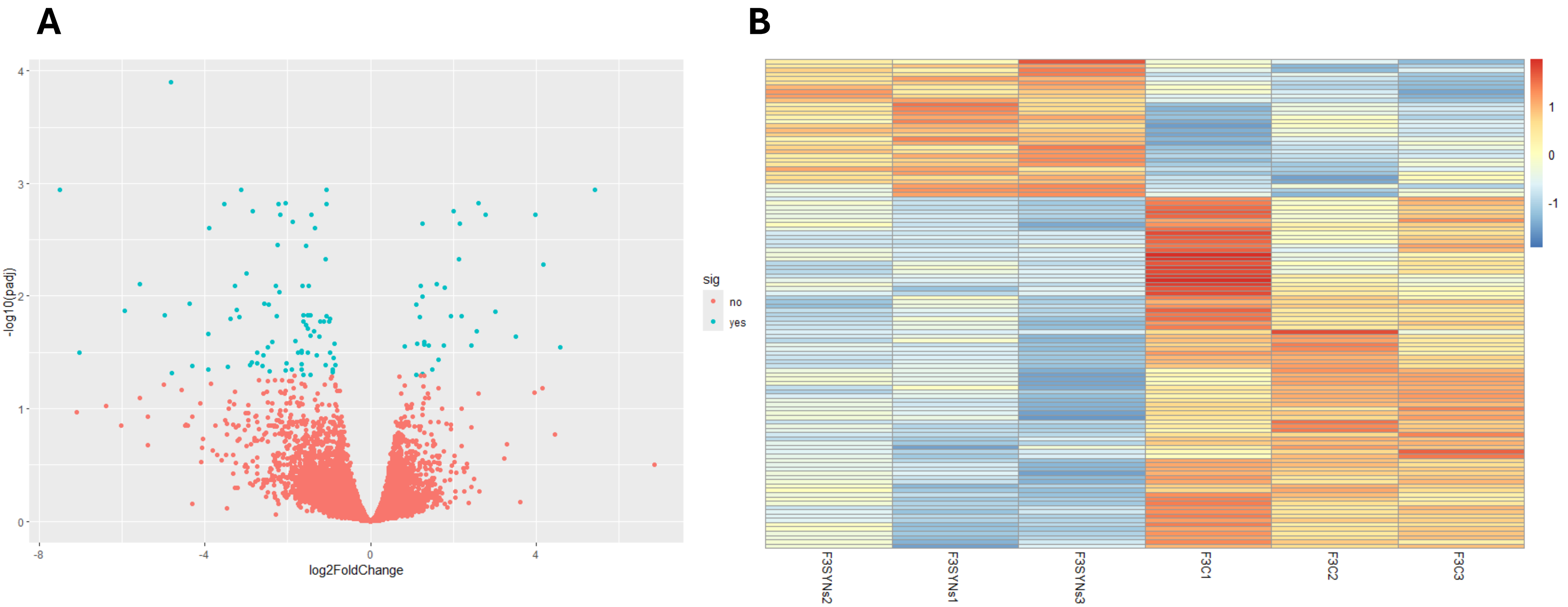

Figure S7: Differential expression gene (DEG) profiles in cecal mucosa of F3: SYNs group vs control (F3SYNs vs F3C). (A) volcano plots, (B) heatmap.

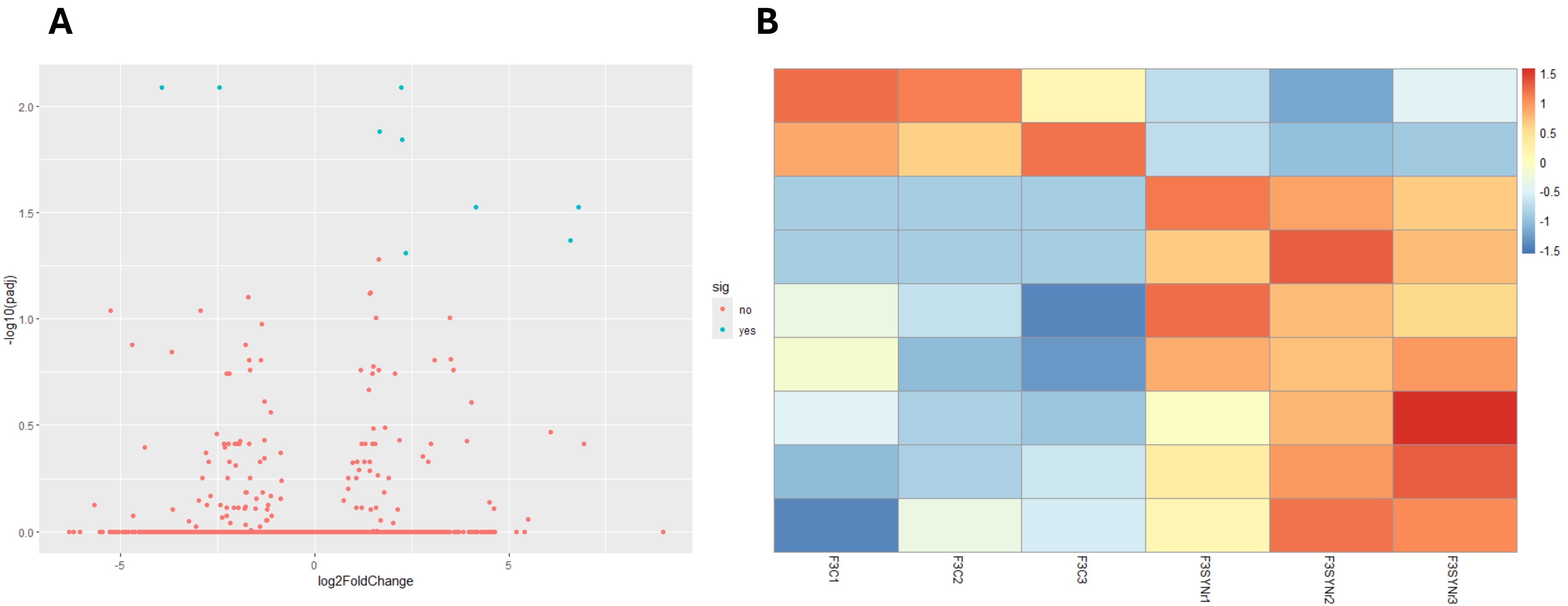

Figure S8: Differential expression gene (DEG) profiles in cecal mucosa of F3: SYNr group vs control (F3SYNr vs F3C). (A) volcano plots, (B) heatmap.

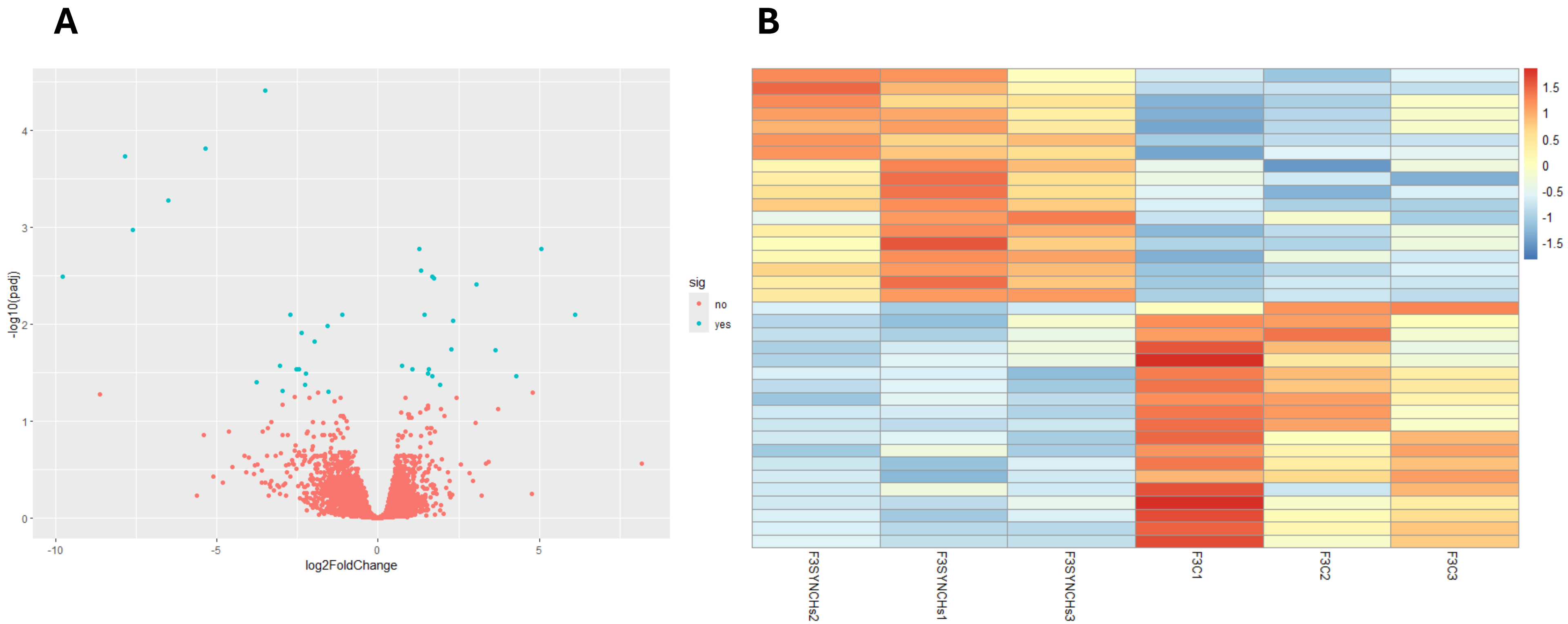

Figure S9: Differential expression gene (DEG) profiles in cecal mucosa of F3: SYNCHs group vs control (F3SYNCHs vs F3C). (A) volcano plots, (B) heatmap.

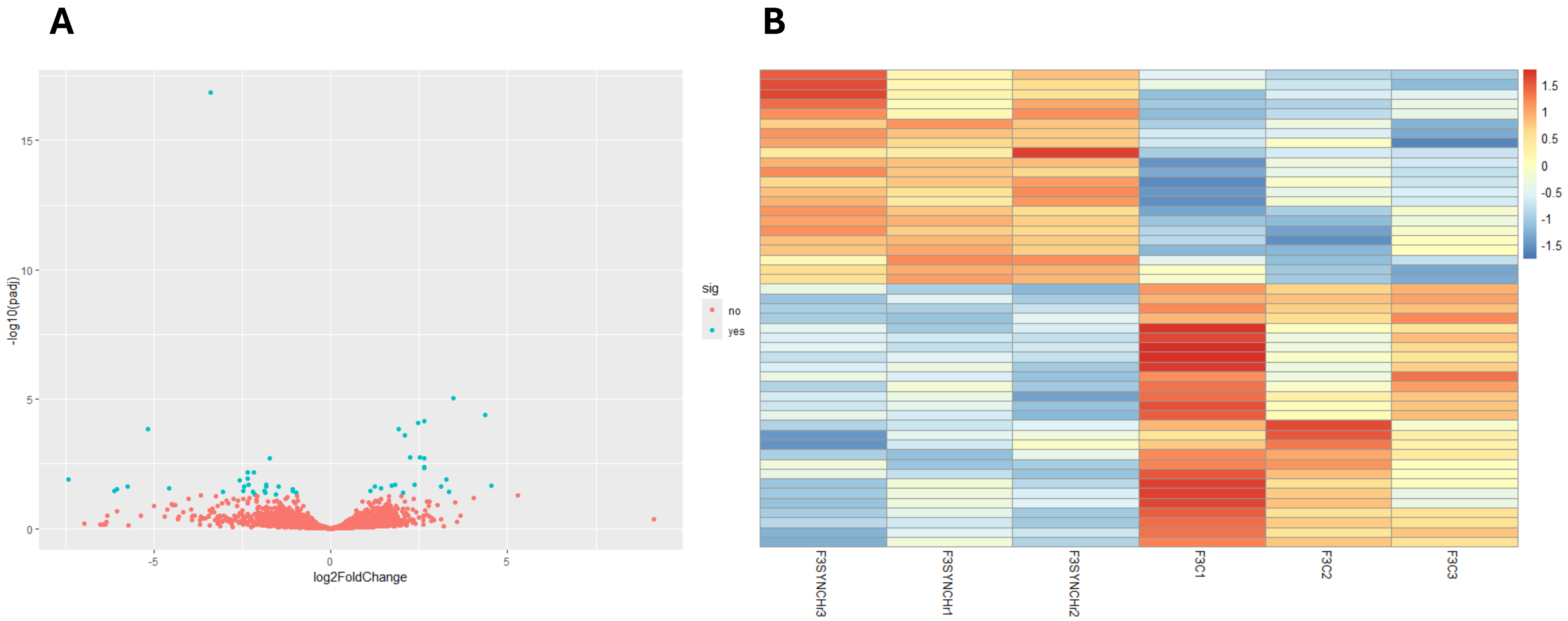

Figure S10: Differential expression gene (DEG) profiles in cecal mucosa of F3: SYNCHr group vs control (F3SYNCHr vs F3C). (A) volcano plots, (B) heatmap.

A

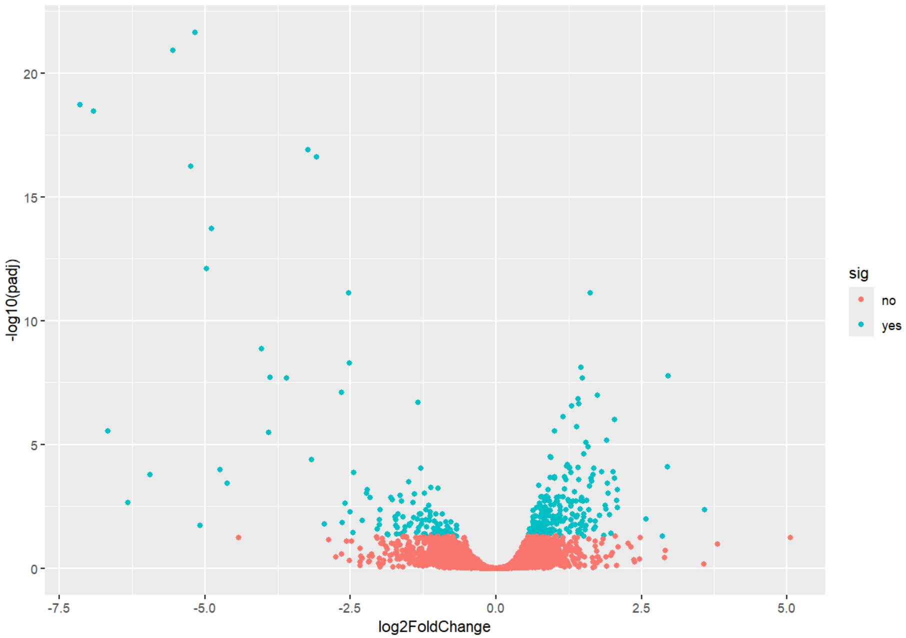

B

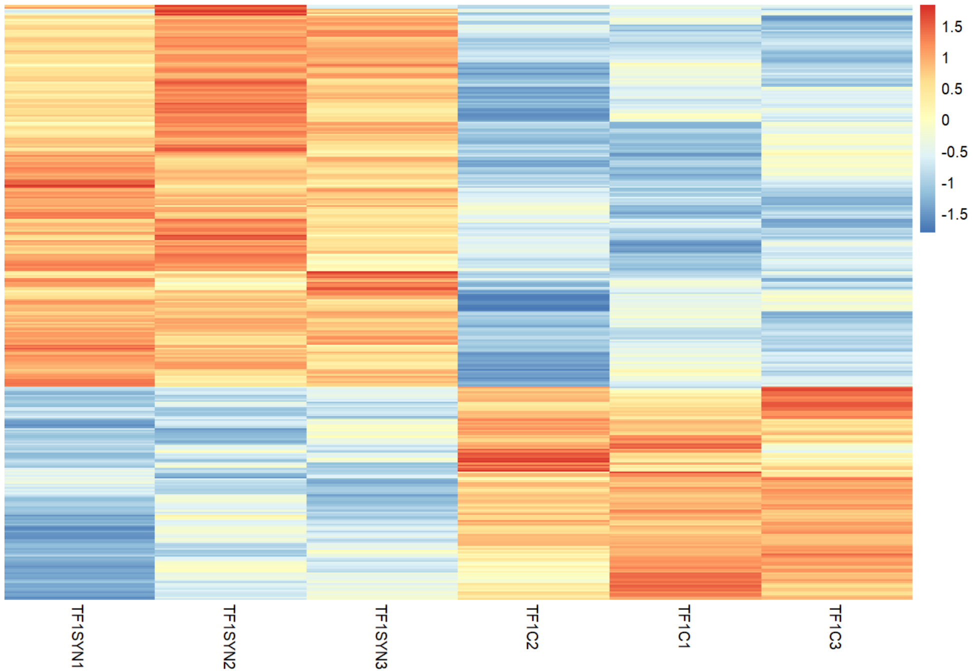

Figure S11: Differential expression gene (DEG) profiles in cecal tonsils of F1: SYN group vs control (TF1SYN vs TF1C). (A) volcano plots, (B) heatmap.

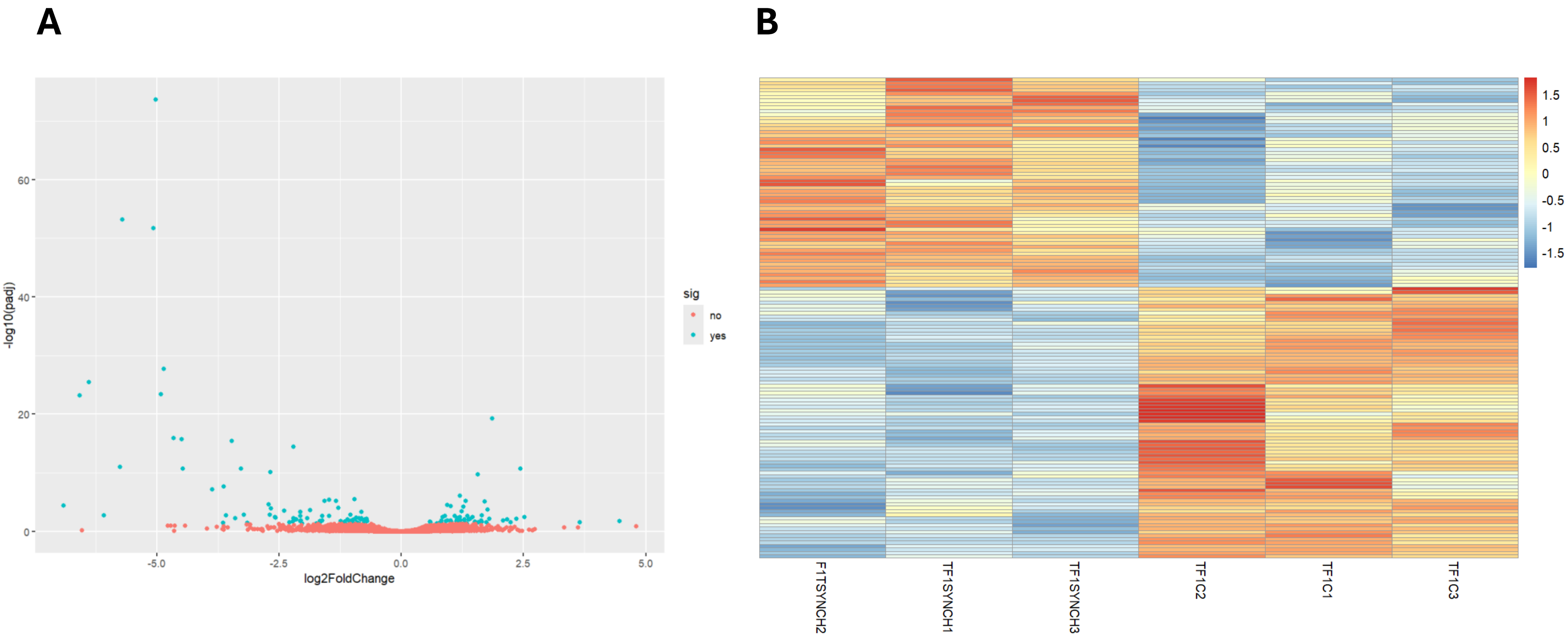

Figure S12: Differential expression gene (DEG) profiles in cecal tonsils of F1: SYNCH group vs control (TF1SYNCH vs TF1C). (A) volcano plots, (B) heatmap.

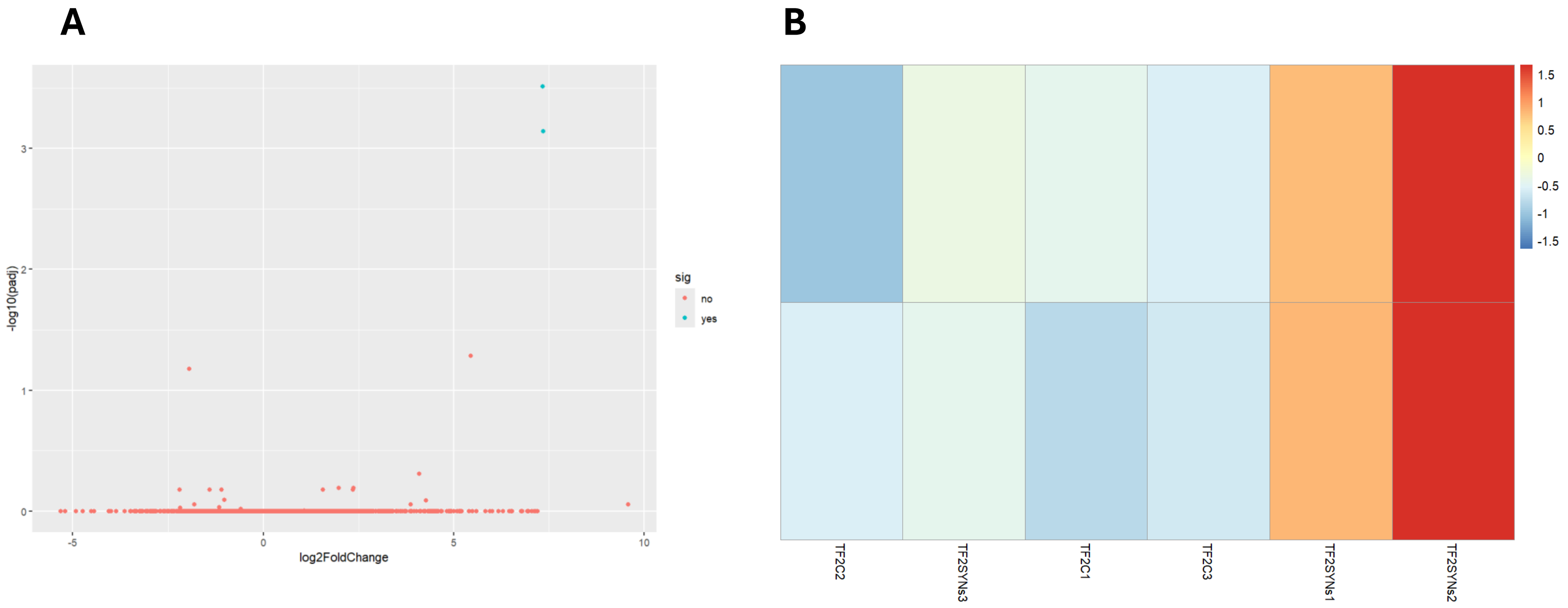

Figure S13: Differential expression gene (DEG) profiles in cecal tonsils of F2: SYNs group vs control (TF2SYNs vs TF2C). (A) volcano plots, (B) heatmap.

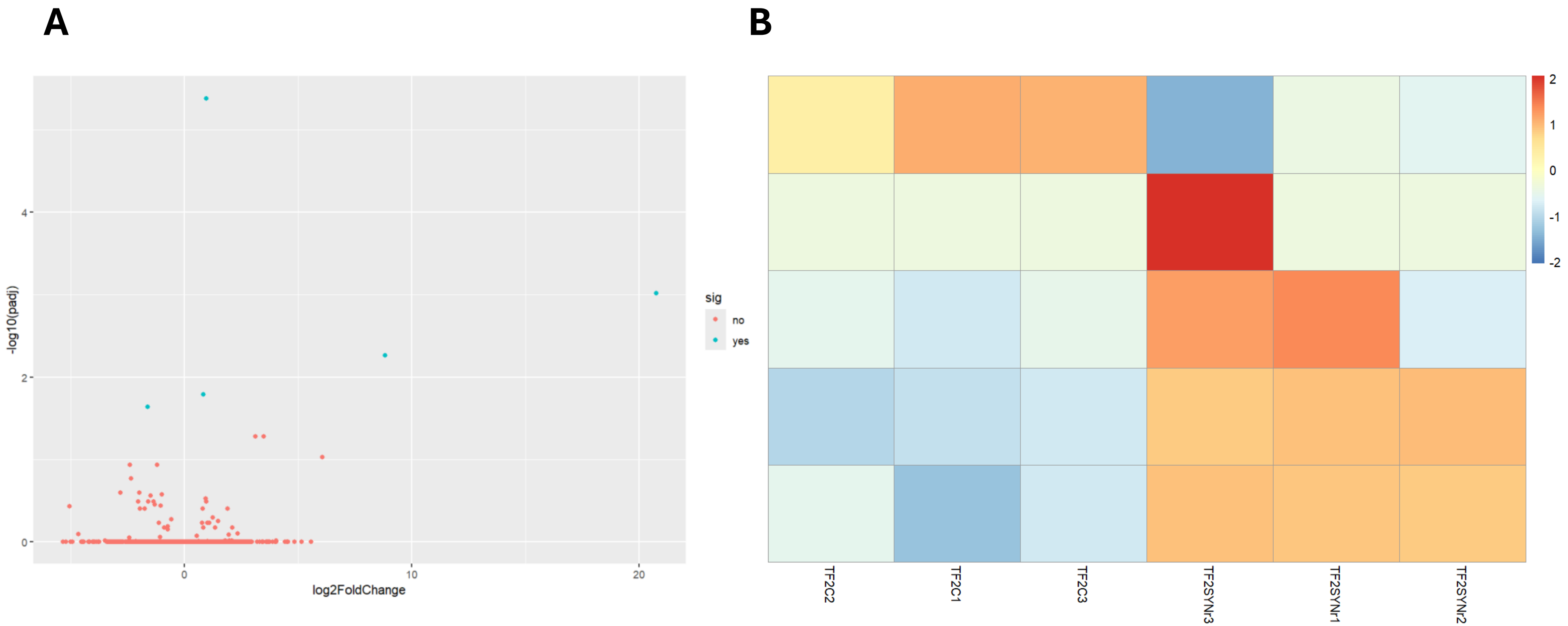

Figure S14: Differential expression gene (DEG) profiles in cecal tonsils of F2: SYNr group vs control (TF2SYNr vs TF2C). (A) volcano plots, (B) heatmap.

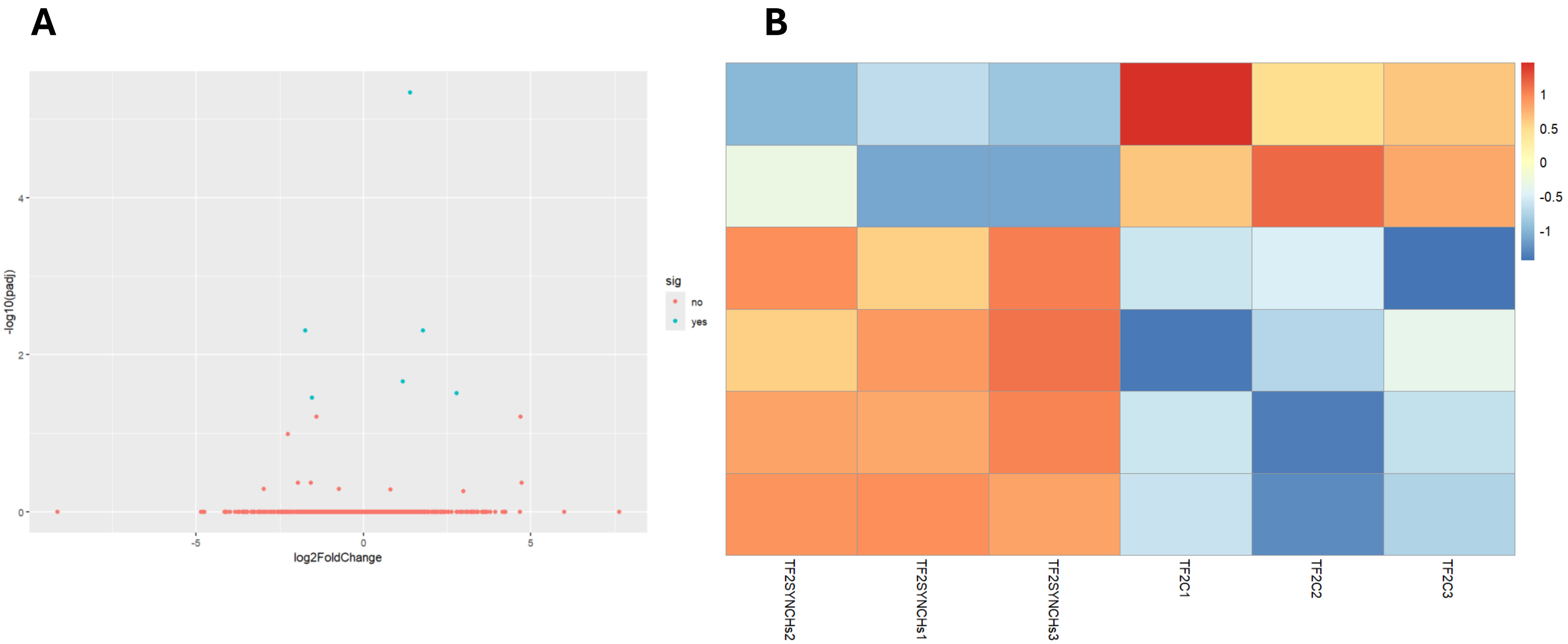

Figure S15: Differential expression gene (DEG) profiles in cecal tonsils of F2: SYNCHs group vs control (TF2SYNCHs vs TF2C). (A) volcano plots, (B) heatmap.

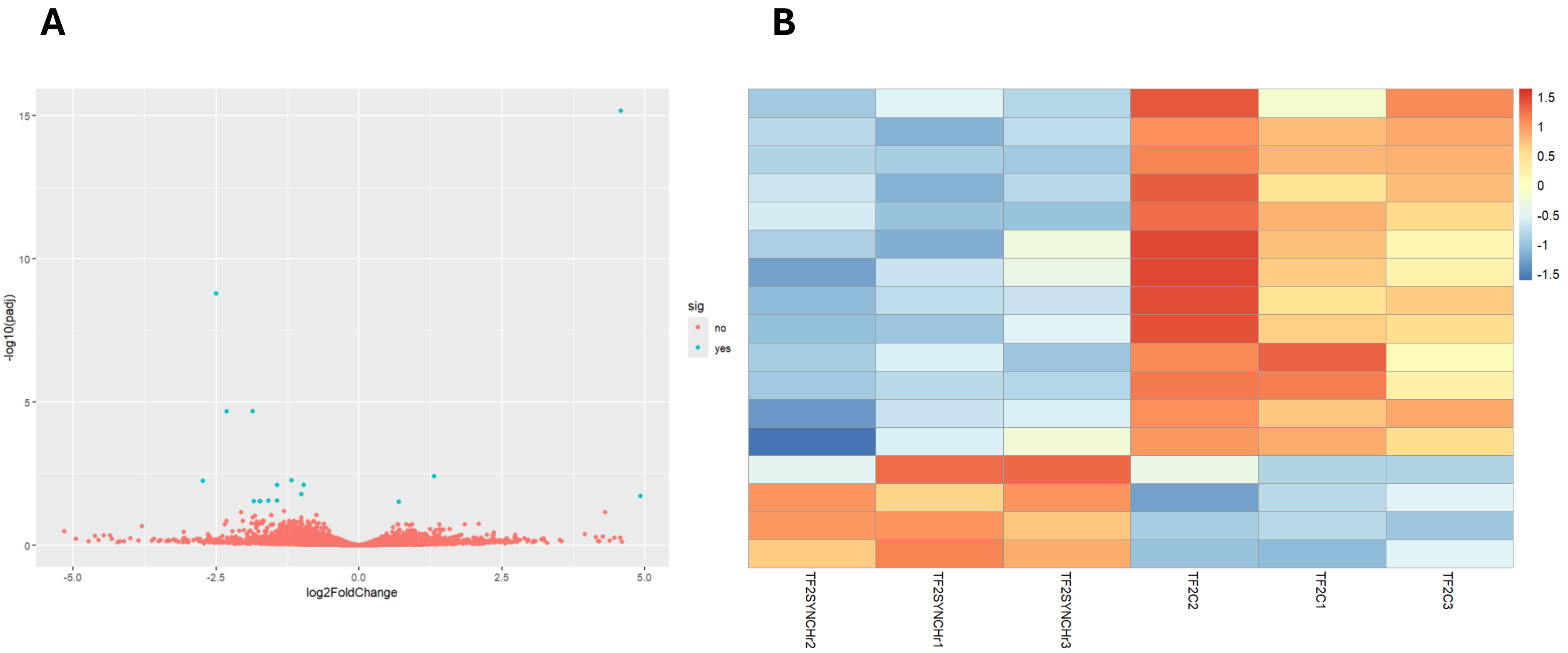

Figure S16: Differential expression gene (DEG) profiles in cecal tonsils of F2: SYNCHr group vs control (TF2SYNCHr vs TF2C). (A) volcano plots, (B) heatmap.

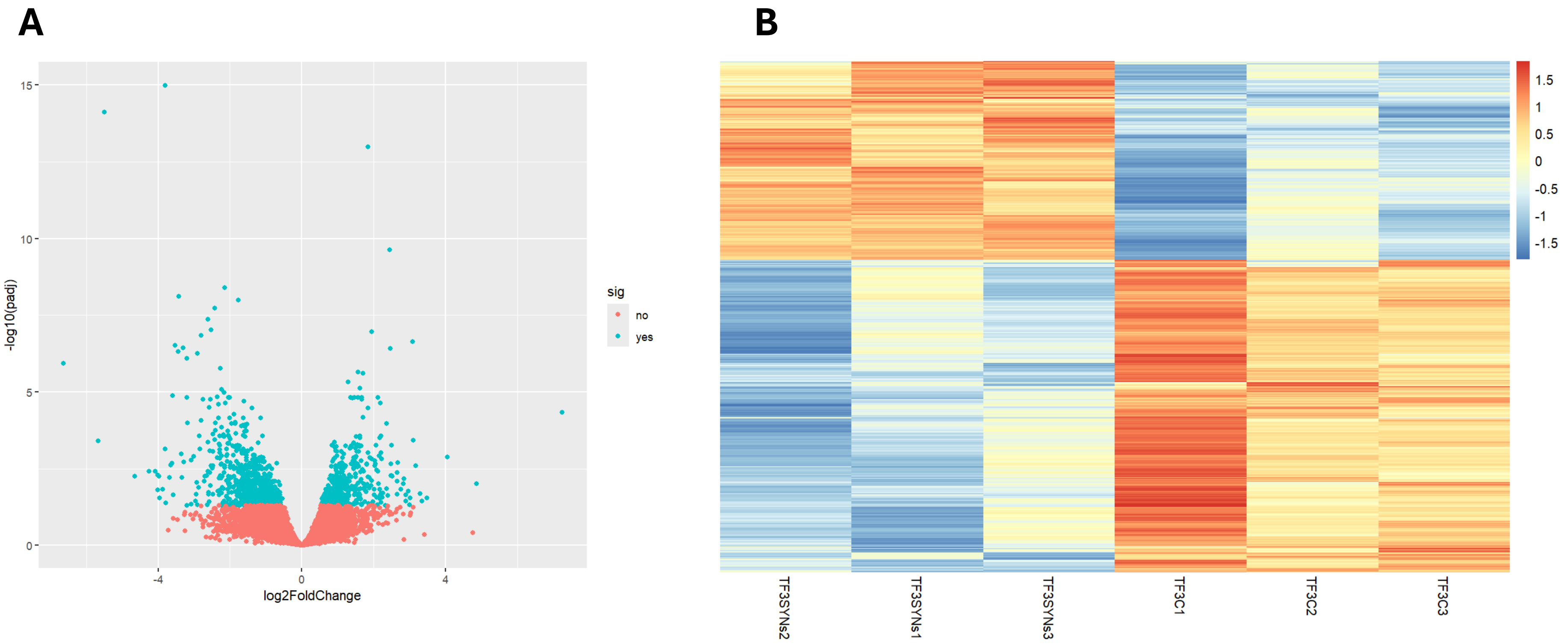

Figure S17: Differential expression gene (DEG) profiles in cecal tonsils of F3: SYNs group vs control (TF3SYNs vs TF3C). (A) volcano plots, (B) heatmap.

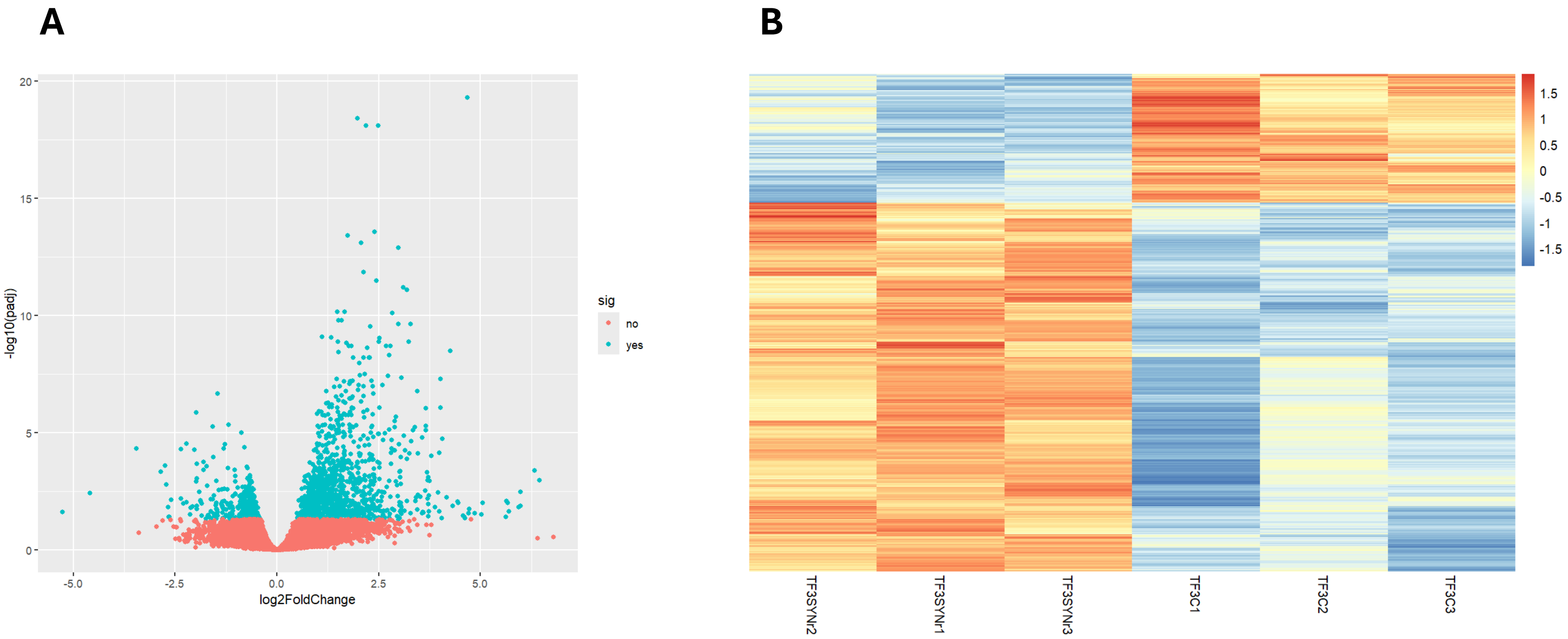

Figure S18: Differential expression gene (DEG) profiles in cecal tonsils of F3: SYNr group vs control (TF3SYNr vs TF3C). (A) volcano plots, (B) heatmap.

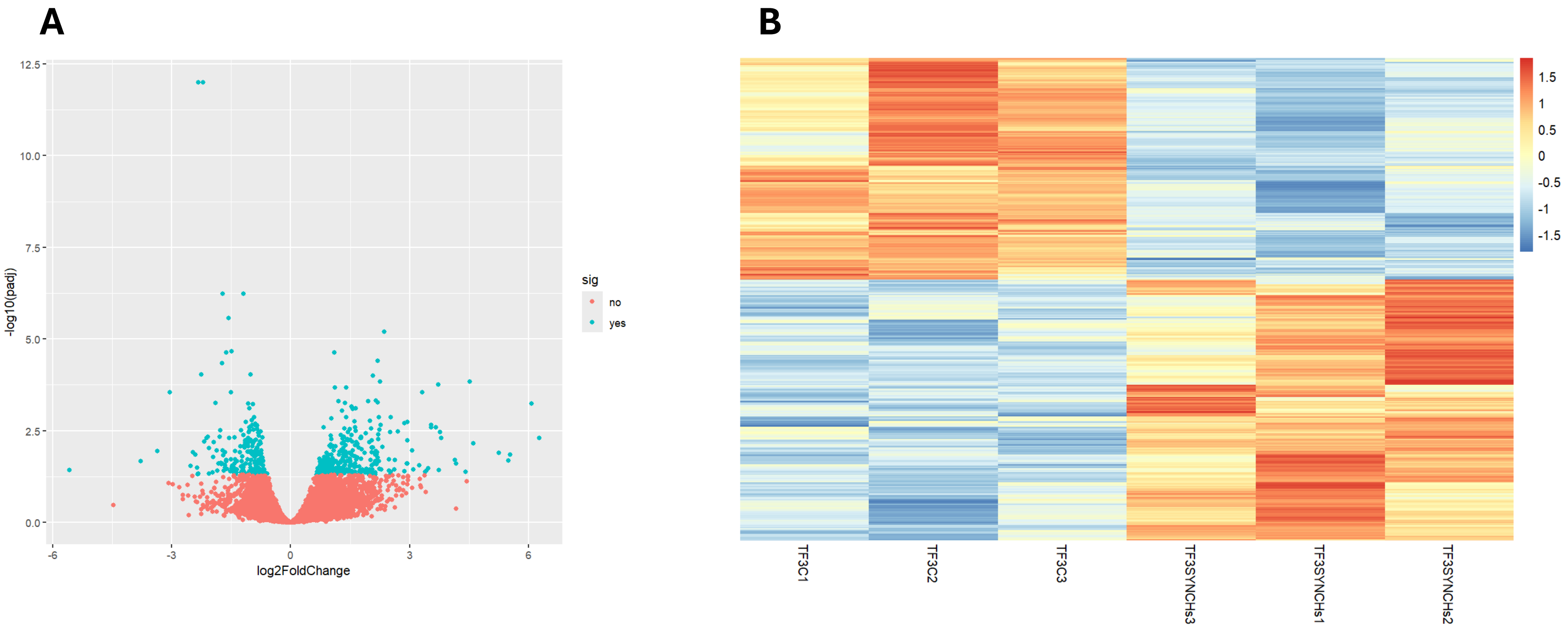

Figure S19: Differential expression gene (DEG) profiles in cecal tonsils of F3: SYNCHs group vs control (TF3SYNCHs vs TF3C). (A) volcano plots, (B) heatmap.

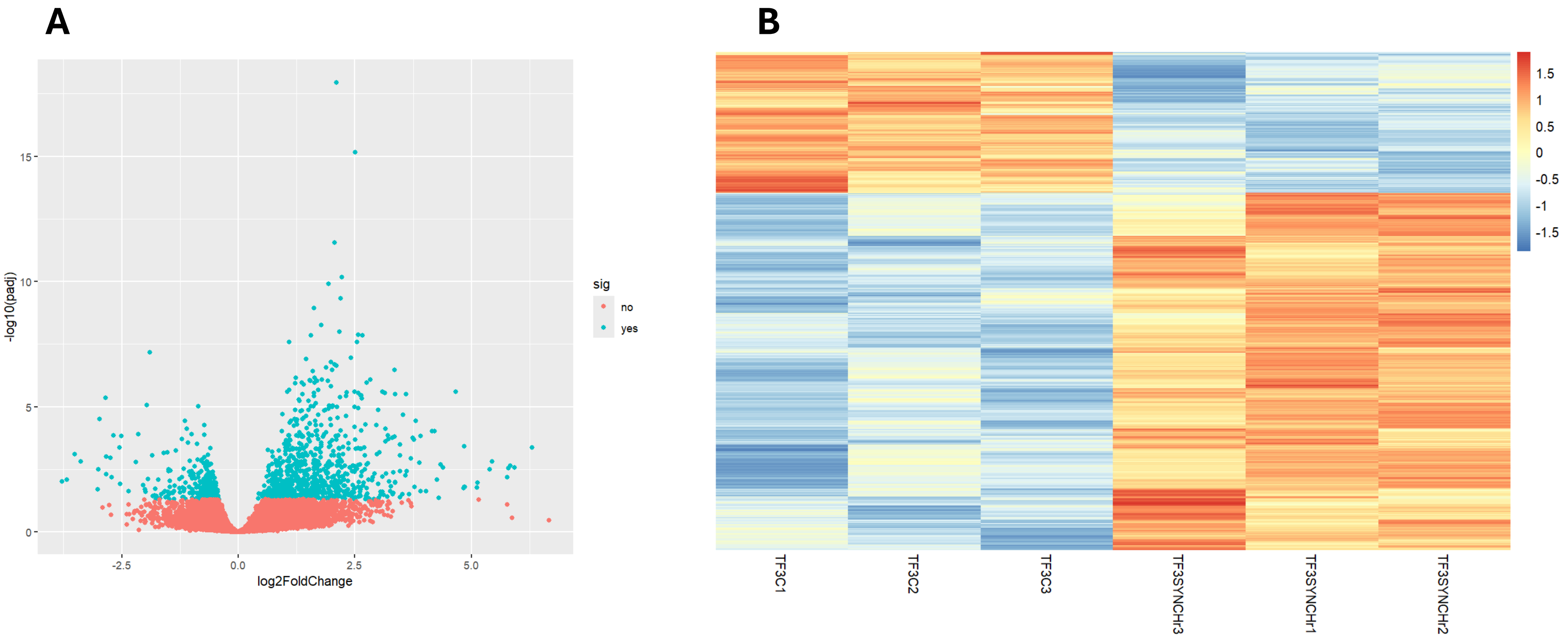

Figure S20: Differential expression gene (DEG) profiles in cecal tonsils of F3: SYNCHr group vs control (TF3SYNCHr vs TF3C). (A) volcano plots, (B) heatmap.
